# Hastat: mining favorable haplotypes of trait-associated genes for breeding improvement in natural populations

**DOI:** 10.1093/hr/uhag018

**Published:** 2026-01-20

**Authors:** Xiaodong Li, Yuhong Wang, Boyu Meng, Zhixuan Qin, Minghao Zhang, Jiaming Song, Kun Lu

**Affiliations:** College of Agronomy and Biotechnology, Southwest University, Chongqing 400715, China; College of Agronomy and Biotechnology, Southwest University, Chongqing 400715, China; College of Agronomy and Biotechnology, Southwest University, Chongqing 400715, China; College of Agronomy and Biotechnology, Southwest University, Chongqing 400715, China; College of Agronomy and Biotechnology, Southwest University, Chongqing 400715, China; College of Agronomy and Biotechnology, Southwest University, Chongqing 400715, China; Engineering Research Center of South Upland Agriculture, Ministry of Education, Chongqing 400715, China; Academy of Agricultural Sciences, Southwest University, Beibei, Chongqing 400715, China; College of Agronomy and Biotechnology, Southwest University, Chongqing 400715, China; Engineering Research Center of South Upland Agriculture, Ministry of Education, Chongqing 400715, China; Academy of Agricultural Sciences, Southwest University, Beibei, Chongqing 400715, China

Dear Editor,

The rapid development of second- and third-generation sequencing technologies, combined with extensive plant germplasm collections, has enabled genome-wide association studies (GWAS) to identify numerous causal variations and trait-associated genes. These advances provide foundational support for modern crop improvement and genomic design breeding. However, the contribution of natural variations in promoter and gene coding sequence to phenotypic change remains poorly characterized. Therefore, the urgent need is how to rapidly mine the favorable haplotypes generated by these variations in natural populations and exploit their potential for future crop improvement. Here, we introduce hastat (https://github.com/swu1019lab/hastat), a comprehensive gene-favorable haplotype mining tool based on the Python language, while an online analysis platform is also provided at https://brassicaibd.cn/analysis/haplotype. Its modular design (view, stat, network, and plot modules) allows users to benefit from a streamlined process (Fig. 1A) and has been successfully implemented in multiple studies [1, 2], which is of great value for modern crop genome design breeding research.

**Figure 1:**
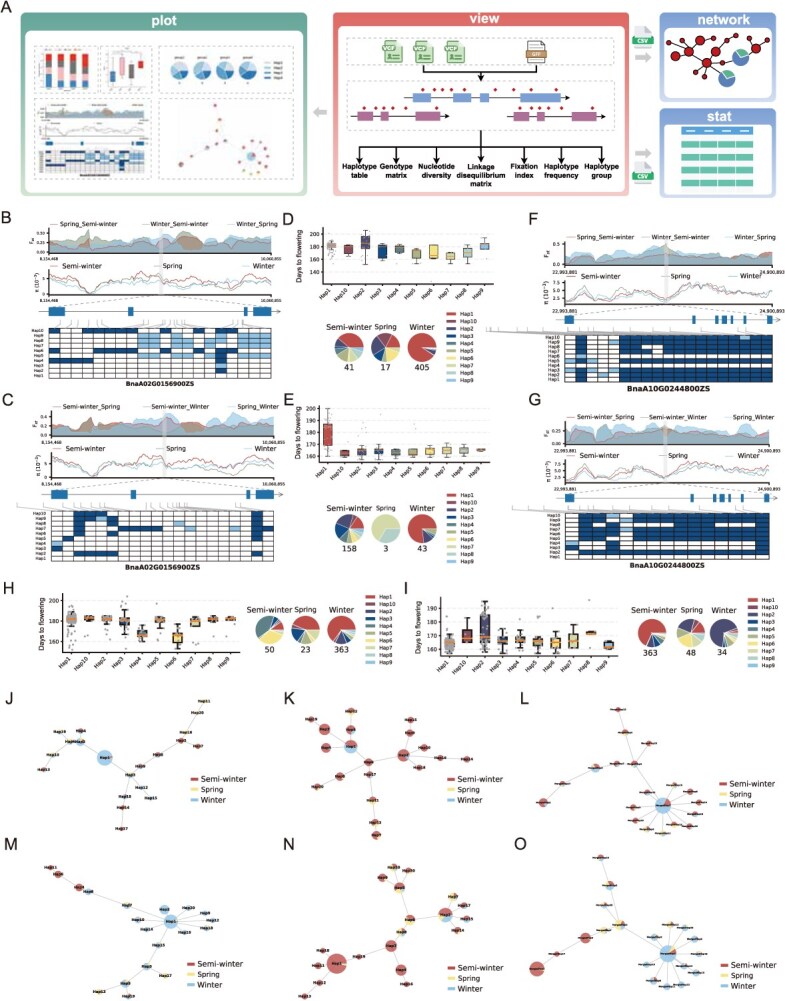
The development and application of Hastat. (A) The development schema of the gene haplotype mining tool. (B - C) The selection and haplotype analysis of *BnFT.A02* in BnaWGS-1007 and BnaWGS-655 panels*.* From top to bottom: *F_ST_* and *π* values in spring, semi-winter, and winter-type accessions across the 1 Mb genomic regions surrounding the gene; gene structure, blue rectangular boxes indicate exons and arrows indicate the direction of the strand; gene haplotype heatmap, blue indicates 1/1 genotypes, light blue indicates 0/1 genotypes, white indicates 0/0 genotypes. (D - E) The one-way ANOVA statistics and distribution of *BnFT.A02* haplotypes in BnaWGS-1007 and BnaWGS-655 panels. (F - G) The selection and haplotype analysis of *BnFLC.A10* in BnaWGS-1007 and BnaWGS-655 panels. (H - I) The one-way ANOVA statistic and distribution of *BnFLC.A10* haplotypes in BnaWGS-1007 and BnaWGS-655 panels. (J - L) The haplotype network analysis of *BnFT.A02* in BnaWGS-1007, BnaWGS-655, and the merged population. (M - O) The haplotype network analysis of *BnFLC.A10* in BnaWGS-1007, BnaWGS-655, and the merged population.

Rapeseed (*Brassica napus*) is a crucial oilseed crop that has successfully adapted to a wide range of climate zones and latitudes, evolving into three main ecotype groups: winter, semi-winter and spring types. In previous study, selective-sweep analysis and GWAS for flowering time confirmed that the *FLOWERING LOCUS T* (*FT*) orthologue on A02 (*BnaA02g12130D* or *BnaA02G0156900ZS*, hereafter as *BnFT.A02*) and the *FLOWERING LOCUS C* (*FLC*) orthologue on A10 (*BnaA10g22080D* or *BnaA10G0244800ZS*, hereafter as *BnFLC.A10*) were significantly associated with flowering-time variation [3]. Here, using this tool, we conducted an in-depth analysis of *BnFT.A02* and *BnFLC.A10* in rapeseed. We first collected a total of 1662 public rapeseed resequencing accessions (Table S1) from PRJNA476657 [3] (1007 samples, 7.95 Tb) and PRJNA358784 [4] (655 samples, 4.03 Tb) from NCBI, and based on ZS11 as the reference genome, variants detection was re-conducted using DeepVariant v1.5.0, and the final obtained variant sets were renamed BnaWGS-1007 and BnaWGS-655 respectively. To ensure robustness, we filtered variants with minor allele frequency (MAF) < 0.05 and missing rate > 0.2, as gene representation (Fig. S1) and sensitivity analyses (Fig. S2) demonstrated these thresholds optimally balance genotype quality and genomic representation.

To gain consistent insight into the genomic selection of genes during evolution [3], we compared fixation index and nucleotide diversity (*F_ST_* and π) (Tables S2 and S3) across the 1-megabase (Mb) genomic regions spanning *BnFT.A02* (Fig. 1B and C) and *BnFLC.A10* (Fig. 1F and G) genes in the BnaWGS-1007 and BnaWGS-655 panel. We identified strong evidence of ecotype divergence around the *BnFT.A02* gene between the spring and winter-type rapeseed. We identified strong evidence of ecotype divergence around *BnFLC.A10* between the semi-winter and winter-type rapeseed based on population fixation statistics (*F_ST_*). Besides, while a selective sweep was detected at the *BnFLC.A10* locus, the *BnFT.A02* region exhibited a distinct selection signal characterized by high nucleotide diversity, suggesting it underwent balancing selection for adaptive flexibility (Fig. S3) [5].

To explore the natural variation of genes under adaptation, we defined ten major haplotypes in the gene coding and promoter sequence (2000 bp from ATG) across the diversity panels (Tables S4–S7). For *BnFT.A02*, haplotype Hap1 appeared the most frequently in both the Bna-WGS-1007 (391 accessions) and Bna-WGS-655 (58 accessions) panels and possessed sequences consistent with the reference genome. Haplotype Hap1 is a late-flowering haplotype with an average flowering time of 181 and 180 days in the two panels, respectively (Fig. 1D and E). In Bna-WGS-655, Hap1 is significantly later than the other haplotypes carried with natural variations, which generally have an average flowering time of about 165 days (Table S8). Hap1 is mainly found in winter-type accessions, followed by semi-winter-type, and is rarely observed in spring-type, resulting in winter and spring-type accessions differentiating significantly in this region but not being different from semi-winter type. In contrast to *BnFT.A02*, we observed that *BnFLC.A10*, the haplotypes Hap1 (Bna-WGS-655) and Hap4 (Bna-WGS-1007) are identical to the reference sequence and the haplotype Hap6 (Bna-WGS-1007) is almost identical. These haplotypes are more inclined to flower early, with average flowering times of 164, 167, and 165 days, respectively, and significantly earlier than other haplotypes with natural variations and are mainly found in semi-winter-type accessions (Fig. 1H and I, Table S8). This contributed to the divergence of semi-winter-type accessions from winter-type and spring-type accessions in the region. Besides, natural variations were also present in the promoter region of *BnFLC.A10* and may influence flowering time by affecting gene expression [3].

Bna-WGS-1007 and Bna-WGS-655 are winter-type preferred population (66.40%) mainly from Europe and semi-winter-type preferred population (78.50%) mainly from China, respectively. They can therefore be used as representative populations of rapeseed origin and after-selection for haplotype network analysis (Tables S9–S12), respectively. Winter-type rapeseeds were first cultivated in Europe and have been suggested to be the original form of *B. napus* [4]; therefore, a major haplotype with the largest winter-type accessions will be formed, and most of the minor haplotypes were also winter-type biased in Bna-WGS-1007 (Fig. 1J and M). After being introduced into China, winter-type rapeseeds have undergone adaptive changes under a combination of natural and artificial selection to suit different geographical environments and climates. Two additional ecotypes, namely, semi-winter rapeseeds and spring-type rapeseeds, adapted to different vernalization times and temperatures, were gradually formed, which has led to most of the haplotypes being semi-winter biased in Bna-WGS-655 (Fig. 1K and N). This phenomenon is more pronounced in the haplotype network (Fig. 1L and O, Fig. S4) after merging two populations, especially for *BnFLC.A10*, where the semi-winter-biased haplotype is almost completely separated from the winter-type-biased haplotype, which is also consistent with our selection analysis results.

Collectively, a comprehensive gene haplotype mining tool was developed and used to perform in-depth analyses on two flowering genes, *BnFT.A02* and *BnFLC.A10*. The results of these analyses revealed a gradual decrease in the number of late-flowering haplotypes *BnFT.A02*^Hap1^ and an increase in the number of early-flowering haplotypes *BnFLC.A10*^Hap1^, *BnFLC.A10*^Hap4^, and *BnFLC.A10*^Hap6^ during evolution and adaptation. Significant artificial selection signals and certain promoter natural variations were identified in *BnFLC.A10*. The selection of these haplotypes may have contributed to the development of the modern semi-winter rapeseed cultivar, ZS11. This finding provides a novel breeding insight to further reduce the flowering time of rapeseed, and this tool will be valuable for future genomic design breeding and crop improvement.

## Supplementary Material

Web_Material_uhag018
