# Supplementary material for: Hastat: mining favorable haplotypes of trait-associated genes for breeding improvement in natural populations
Source: Hortic Res. 2026 Jan 20;13(4):uhag018. doi: 10.1093/hr/uhag018 (PMC13098370; doi:10.1093/hr/uhag018)
Supplement: Web_Material_uhag018 [file web_material_uhag018.pdf]

Supplementary Figure

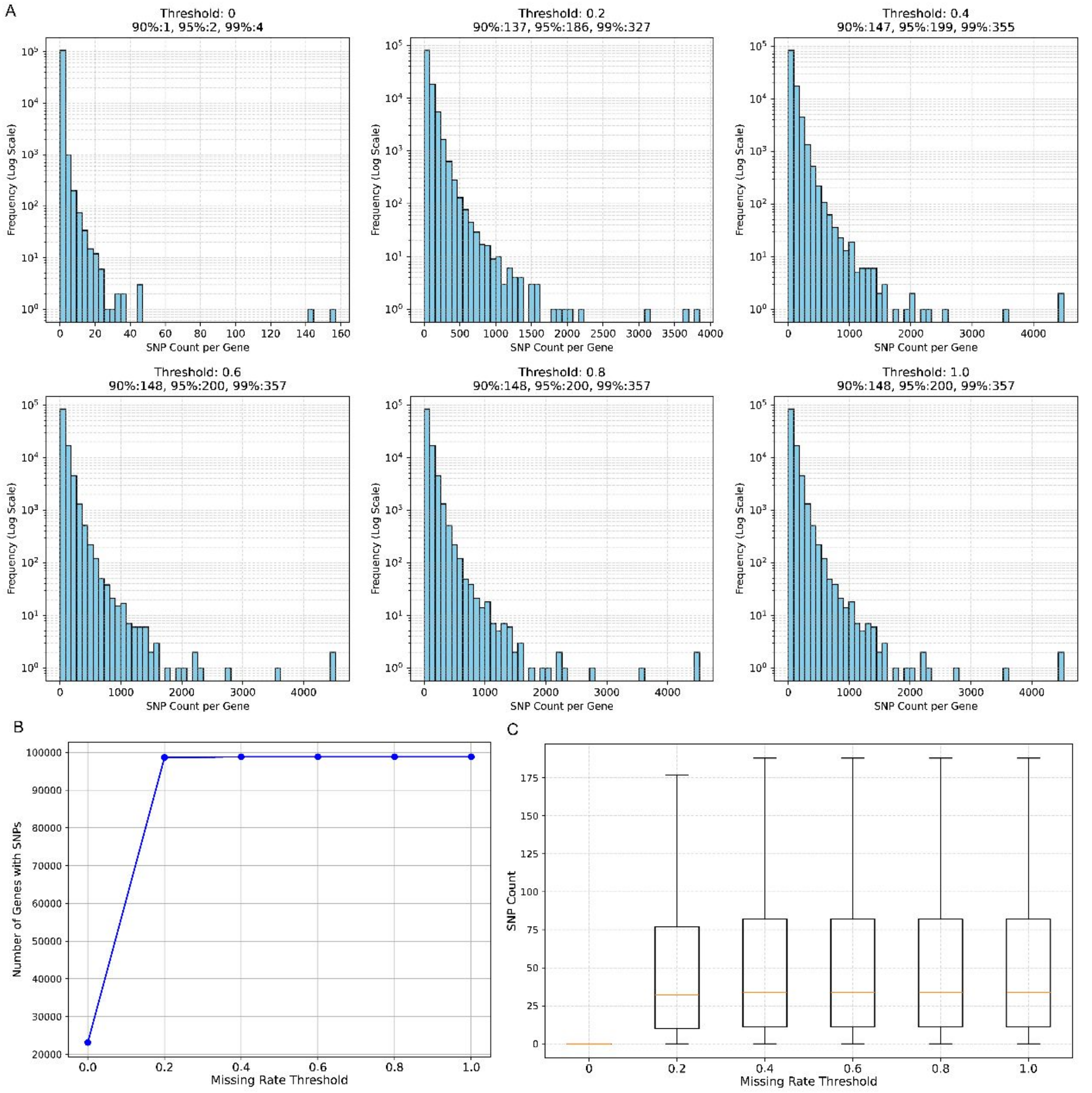

Fig S1 Evaluation of gene representation and SNP density across different missing data thresholds in BnaWGS-655 panel. (A) The distribution of SNP counts per gene under varying missing rate thresholds (0, 0.2, 0.4, 0.6, 0.8, and 1.0). The Y-axis represents the frequency of genes on a logarithmic scale to visualize the wide range of SNP densities. The 90th, 95th, and 99th percentiles of SNP counts per gene are indicated for each threshold. (B) The relationship between missing rate thresholds and the number of retained genes (genes containing at least one SNP). (C) Boxplots displaying the distribution of SNP counts per gene across different missing rate thresholds.

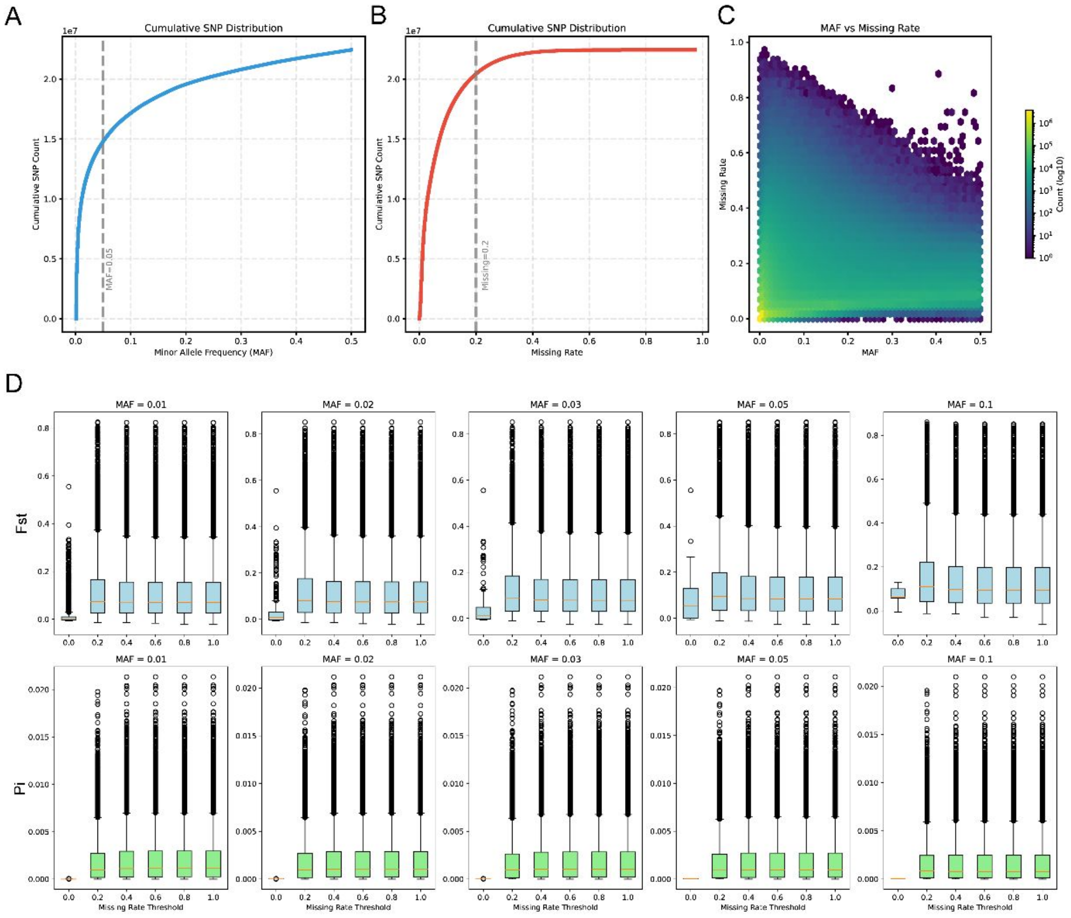

Fig S2 Characterization of variant quality statistics and sensitivity analysis of population genetic statistics under varying filtering parameters. (A) Cumulative distribution of SNPs relative to Minor Allele Frequency (MAF). The y-axis represents the cumulative number of SNPs, and the gray dashed vertical line marks the threshold of MAF = 0.05. (B) Cumulative distribution of SNPs relative to Missing Rate. The y-axis shows the cumulative count of variants, with the gray dashed vertical line indicating the threshold of Missing Rate = 0.2. (C) Hexbin density plot illustrating the relationship between MAF and Missing Rate. The color scale represents the log10-transformed count of variants in each hexagonal bin, highlighting the density distribution of variants across different quality metrics. (D) The distribution of Fixation Index (F_{ST}, top row, Semi-winter and Spring) and Nucleotide Diversity (π, bottom row, Semi-winter and Spring) values across different Minor Allele Frequency (MAF) thresholds (columns: 0.01, 0.02, 0.03, 0.05, 0.1) in BnaWGS-655. Within each panel, the X-axis represents the missing data rate threshold (0, 0.2, 0.4, 0.6, 0.8, 1.0), and the Y-axis represents the calculated statistic values.

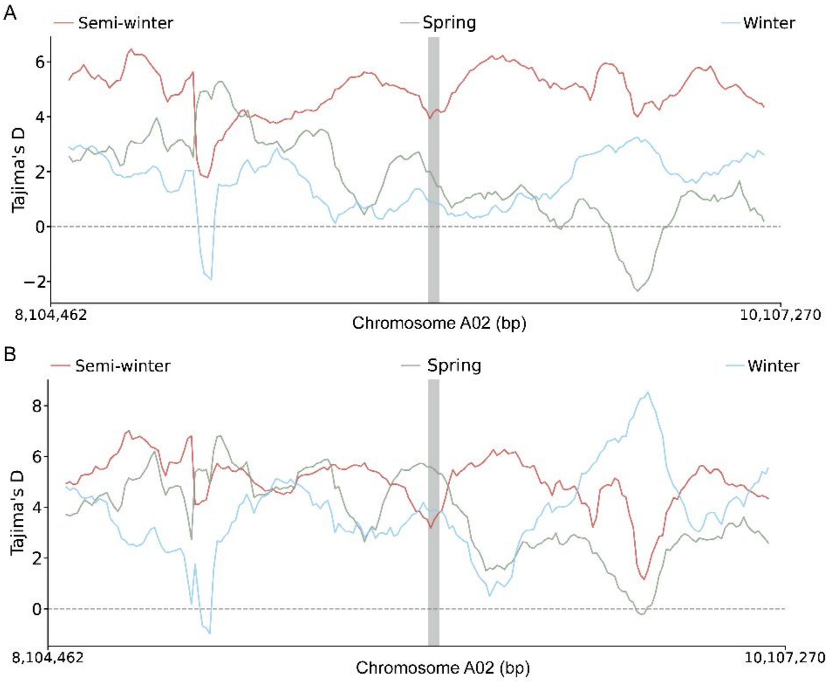

Fig S3 The balancing selection analysis of *BnFT.A02* in two panels. (A) Tajima's D values in spring, semi-winter, and winter-type accessions across the 1 Mb genomic regions surrounding the gene in BnaWGS-655 panel. (B) Tajima's D values in spring, semi-winter, and winter-type accessions across the 1 Mb genomic regions surrounding the gene in BnaWGS-1007 panel. The gray rectangular box indicates the region of *BnFT.A02*.

Review

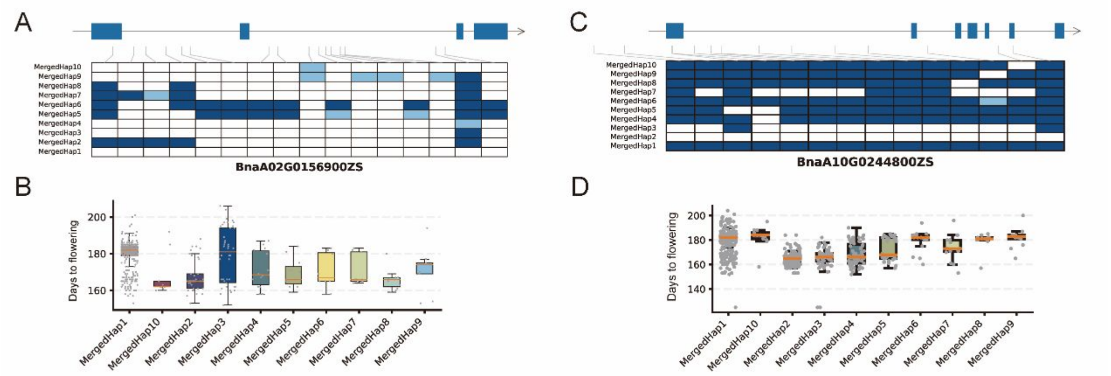

Fig S4 Gene haplotypes analysis and statistics in merged panels. A and B The gene haplotypes and one-way ANOVA statistics of *BnFT.A02* haplotypes based on core variants of merged panels. C and D The gene haplotypes and one-way ANOVA statistics of *BnFLC.A10* haplotypes based on core variants of merged panels

Supplemental materials and methods

The resequencing data collection and variation analysis

A total of 11.98 Tb of public rapeseed resequencing data from PRJNA476657 (1,007 accessions) and PRJNA358784 (655 accessions) were collected from NCBI and renamed BnaWGS-1,007 and BnaWGS-655, respectively. Raw sequencing reads were quality control using FastQC v0.11.9 (https://www.bioinformatics.babraham.ac.uk/projects/fastqc/) and low-quality reads were filtered using Fastp v0.23.2 (Chen et al. 2018). Clean reads were mapped to the rapeseed reference genome ZS11 using BWA v0.7.17 (Li and Durbin 2009). Duplicate reads were then removed using Sambamba v0.8.2 (Tarasov et al. 2015). Variant detection was performed using DeepVariant v1.5.0 (Poplin et al. 2018), and the gVCF files were merged using GLnexus v1.4.1 (Yun et al. 2021). Variants with minor allele frequencies less than 0.05 and genotype missing rates greater than 0.2 were filtered using VCFtools v0.1.16 (Danecek et al. 2011). Beagle v4.1 (Browning and Browning 2016) was used to impute missing genotypes, and variant effects were annotated using SnpEff v5.2 (Cingolani et al. 2012).

Development of a gene haplotype mining tool on population scale

Hastat was built on top of the Python programming language with modular design concept, which makes it easy for users to install, use and pipeline integrate. The tool consists of four main modules: view, stat, network, and plot. In the 'view' module, we used gffutils (https://github.com/daler/gffutils) and pysam (https://github.com/pysam-developers/pysam) to quickly process the file inputs, and the pandas (https://pandas.pydata.org/) library to develop the core algorithms for haplotype analysis, and scikit-allel (https://github.com/cggh/scikit-allel) to analyze genetic variation and calculate population genetic statistics. In the 'stat' module, we used one-way Analysis of Variance (ANOVA) to associate haplotypes and phenotypes for significance test, and TukeyHSD method for multiple corrections. In the 'network' module, we used Minimum Spanning Tree (MST) and Minimum Spanning Network (MSN) algorithms to construct haplotype networks. Finally, based on Matplotlib (https://matplotlib.org/) library, we developed an advanced visualization module 'plot', which not only creates publication quality plots, but also supports highly customized gene haplotype visual style and layout to make the figure fancier. The following provides a detailed introduction to each module:

The **view** module serves as the cornerstone of hastat, providing comprehensive capabilities for analyzing genetic variation data from VCF files. This module enables researchers to extract and analyze genotype information, haplotype patterns, and population genetic statistics for specific genes or genomic regions. It supports multiple analysis types, including raw genotype data extraction, haplotype table generation, haplotype group assignment, frequency analysis, nucleotide diversity (π) calculation, fixation index (F_{ST}) computation, and multi-population comparison studies. The module incorporates advanced features such as homologous gene analysis, allowing researchers to study gene families and paralogous relationships simultaneously. With

For Review Only

built-in heterozygosity filtering capabilities and customizable upstream/downstream region specifications, the view module provides a robust foundation for downstream statistical and visualization analyses.

The **stat** module implements sophisticated statistical methodologies for haplotype-phenotype association studies. Built upon the foundation of ANOVA and multiple comparison procedures, this module enables researchers to strictly assess the relationship between haplotype diversity and phenotypic variation. The module supports multiple statistical frameworks, including Tukey's Honestly Significant Difference (HSD) test and comprehensive pairwise testing methods. It incorporates minimum haplotype size thresholds to ensure statistical robustness and provides detailed output including mean differences, adjusted p-values, confidence intervals, and significance indicators. The stat module is particularly valuable for identifying haplotype-specific phenotypic effects and understanding the genetic basis of trait variation in natural populations.

The **network** module implements advanced algorithms for constructing and analyzing haplotype networks, providing insights into evolutionary relationships. This module utilizes Hamming distance calculations to measure genetic similarity between haplotypes and employs two distinct network construction methodologies: Minimum Spanning Tree (MST) and Minimum Spanning Network (MSN). The MST approach creates the most parsimonious network connecting all haplotypes, while the MSN method incorporates additional edges based on user-defined threshold factors, revealing more complex evolutionary relationships. The network module is essential for understanding haplotype evolution and identifying ancestral haplotypes.

The **plot** module provides a comprehensive visualization toolkit designed specifically for haplotype analysis results. This module encompasses five distinct visualization types, each optimized for different analytical perspectives. The bar plot functionality enables frequency-based comparisons across populations and haplotypes, while pie charts offer intuitive representation of haplotype distributions. Box plots facilitate statistical comparison of phenotypic values among haplotype groups, incorporating significance testing and multiple comparison procedures. The network visualization component transforms network analysis results into publication-ready graphical representations with customizable layouts, node styling, and edge properties. The gene haplotype plot integrates haplotype information with gene structure through Tom's Obvious Minimal Language (TOML)-based configuration. This feature allows researchers to display variant positions relative to gene features such as exons, introns, and regulatory regions with fully customizable visual elements. The TOML configuration system supports gene-specific styling, annotation positioning, haplotype color schemes, and multi-layered visual elements including π and F_{ST} data overlays. The TOML-based approach ensures reproducibility and enables easy sharing of visualization parameters across research teams.

For Review Only

Web server implementation

An online Gene haplotype analysis platform equipped with population resequenced data has been implemented using a Tencent Cloud Lighthouse application server (4

vCPUs, 8 GB RAM). The platform is built with VUE3 (https://vuejs.org/) for the front-end framework, Django (https://www.djangoproject.com/) for the back-end server, and PostgreSQL (https://www.postgresql.org/) for database management. Nginx (https://nginx.org/) is employed as a reverse proxy to facilitate efficient and secure request handling.

For Review Only

References

Browning BL and Browning SR. Genotype Imputation with Millions of Reference Samples. The American Journal of Human Genetics. 2016;**98**(1):116–126. https://doi.org/10.1016/j.ajhg.2015.11.020

Chen S, Zhou Y, Chen Y, and Gu J. fastp: an ultra-fast all-in-one FASTQ preprocessor. Bioinformatics. 2018;**34**(17):i884–i890. https://doi.org/10.1093/bioinformatics/bty560

Cingolani P, Platts A, Wang LL, Coon M, Nguyen T, Wang L, Land SJ, Lu X, and Ruden DM. A program for annotating and predicting the effects of single nucleotide polymorphisms, SnpEff: SNPs in the genome of Drosophila melanogaster strain w^{1118} ; iso-2; iso-3. Fly. 2012;**6**(2):80–92. https://doi.org/10.4161/fly.19695

Danecek P, Auton A, Abecasis G, Albers CA, Banks E, DePristo MA, Handsaker RE, Lunter G, Marth GT, Sherry ST, et al. The variant call format and VCFtools. Bioinformatics. 2011;**27**(15):2156–2158. https://doi.org/10.1093/bioinformatics/btr330

Li H and Durbin R. Fast and accurate short read alignment with Burrows–Wheeler transform. Bioinformatics. 2009;**25**(14):1754–1760. https://doi.org/10.1093/bioinformatics/btp324

Poplin R, Chang P-C, Alexander D, Schwartz S, Colthurst T, Ku A, Newburger D, Dijamco J, Nguyen N, Afshar PT, et al. A universal SNP and small-indel variant caller using deep neural networks. Nat Biotechnol. 2018;**36**(10):983–987. https://doi.org/10.1038/nbt.4235

Tarasov A, Vilella AJ, Cuppen E, Nijman IJ, and Prins P. Sambamba: fast processing of NGS alignment formats. Bioinformatics. 2015;**31**(12):2032–2034. https://doi.org/10.1093/bioinformatics/btv098

Yun T, Li H, Chang P-C, Lin MF, Carroll A, and McLean CY. Accurate, scalable cohort variant calls using DeepVariant and GLnexus. Bioinformatics. 2021;**36**(24):5582–5589. https://doi.org/10.1093/bioinformatics/btaa1081

For Review Only

Table S1. The basic information of two natural populations

Panel	Number	ecotype	Flowering time (Days)
BnaWGS-1007	145	Semi-winter	165.04±9.75
BnaWGS-1007	188	Spring	165.33±10.35
BnaWGS-1007	658	Winter	182.32±4.17
BnaWGS-655	514	Semi-winter	164.52±3.86
BnaWGS-655	81	Spring	165.6±5.56
BnaWGS-655	60	Winter	183.95±7.51

For Review Only

Table S2. ***F_{ST}*** **values in spring, semi-winter and winter-type accessions across the 1 Mb genomic region**

panel	gene	chrom	start	end	counts	fst
BnaWGS-1007	BnaA02G0156900ZS	scaffoldA02	8104469	8204468	1376	0.183657
BnaWGS-1007	BnaA02G0156900ZS	scaffoldA02	8114469	8214468	1411	0.172747
BnaWGS-1007	BnaA02G0156900ZS	scaffoldA02	8124469	8224468	1391	0.159511
BnaWGS-1007	BnaA02G0156900ZS	scaffoldA02	8134469	8234468	1206	0.155809
BnaWGS-1007	BnaA02G0156900ZS	scaffoldA02	8144469	8244468	1278	0.161811
BnaWGS-1007	BnaA02G0156900ZS	scaffoldA02	8154469	8254468	1239	0.156324
BnaWGS-1007	BnaA02G0156900ZS	scaffoldA02	8164469	8264468	1133	0.17529
BnaWGS-1007	BnaA02G0156900ZS	scaffoldA02	8174469	8274468	1097	0.183884
BnaWGS-1007	BnaA02G0156900ZS	scaffoldA02	8184469	8284468	1121	0.176274
BnaWGS-1007	BnaA02G0156900ZS	scaffoldA02	8194469	8294468	1049	0.184886
BnaWGS-1007	BnaA02G0156900ZS	scaffoldA02	8204469	8304468	1058	0.191539
BnaWGS-1007	BnaA02G0156900ZS	scaffoldA02	8214469	8314468	973	0.206505
BnaWGS-1007	BnaA02G0156900ZS	scaffoldA02	8224469	8324468	971	0.212406
BnaWGS-1007	BnaA02G0156900ZS	scaffoldA02	8234469	8334468	1034	0.202524
BnaWGS-1007	BnaA02G0156900ZS	scaffoldA02	8244469	8344468	941	0.18875
BnaWGS-1007	BnaA02G0156900ZS	scaffoldA02	8254469	8354468	898	0.181656
BnaWGS-1007	BnaA02G0156900ZS	scaffoldA02	8264469	8364468	1024	0.170811
BnaWGS-1007	BnaA02G0156900ZS	scaffoldA02	8274469	8374468	1058	0.161859
BnaWGS-1007	BnaA02G0156900ZS	scaffoldA02	8284469	8384468	1052	0.157085
BnaWGS-1007	BnaA02G0156900ZS	scaffoldA02	8294469	8394468	1001	0.160022
BnaWGS-1007	BnaA02G0156900ZS	scaffoldA02	8304469	8404468	934	0.160658
BnaWGS-1007	BnaA02G0156900ZS	scaffoldA02	8314469	8414468	914	0.16258
BnaWGS-1007	BnaA02G0156900ZS	scaffoldA02	8324469	8424468	964	0.151433
BnaWGS-1007	BnaA02G0156900ZS	scaffoldA02	8334469	8434468	935	0.156626
BnaWGS-1007	BnaA02G0156900ZS	scaffoldA02	8344469	8444468	858	0.163258
BnaWGS-1007	BnaA02G0156900ZS	scaffoldA02	8354469	8454468	831	0.149173
BnaWGS-1007	BnaA02G0156900ZS	scaffoldA02	8364469	8464468	648	0.124349
BnaWGS-1007	BnaA02G0156900ZS	scaffoldA02	8374469	8474468	545	0.108479
BnaWGS-1007	BnaA02G0156900ZS	scaffoldA02	8384469	8484468	455	0.114852
BnaWGS-1007	BnaA02G0156900ZS	scaffoldA02	8394469	8494468	432	0.110637
BnaWGS-1007	BnaA02G0156900ZS	scaffoldA02	8404469	8504468	420	0.10938
BnaWGS-1007	BnaA02G0156900ZS	scaffoldA02	8414469	8514468	354	0.097711
BnaWGS-1007	BnaA02G0156900ZS	scaffoldA02	8424469	8524468	196	0.091164
BnaWGS-1007	BnaA02G0156900ZS	scaffoldA02	8434469	8534468	122	0.091076
BnaWGS-1007	BnaA02G0156900ZS	scaffoldA02	8444469	8544468	87	0.073395
BnaWGS-1007	BnaA02G0156900ZS	scaffoldA02	8454469	8554468	38	0.138178
BnaWGS-1007	BnaA02G0156900ZS	scaffoldA02	8464469	8564468	80	0.172434
BnaWGS-1007	BnaA02G0156900ZS	scaffoldA02	8474469	8574468	149	0.207317
BnaWGS-1007	BnaA02G0156900ZS	scaffoldA02	8484469	8584468	160	0.2177
BnaWGS-1007	BnaA02G0156900ZS	scaffoldA02	8494469	8594468	190	0.223698
BnaWGS-1007	BnaA02G0156900ZS	scaffoldA02	8504469	8604468	312	0.194342
BnaWGS-1007	BnaA02G0156900ZS	scaffoldA02	8514469	8614468	444	0.182584
BnaWGS-1007	BnaA02G0156900ZS	scaffoldA02	8524469	8624468	468	0.202728
BnaWGS-1007	BnaA02G0156900ZS	scaffoldA02	8534469	8634468	579	0.193971
BnaWGS-1007	BnaA02G0156900ZS	scaffoldA02	8544469	8644468	646	0.191996
BnaWGS-1007	BnaA02G0156900ZS	scaffoldA02	8554469	8654468	756	0.190516
BnaWGS-1007	BnaA02G0156900ZS	scaffoldA02	8564469	8664468	770	0.187465
BnaWGS-1007	BnaA02G0156900ZS	scaffoldA02	8574469	8674468	778	0.187713
BnaWGS-1007	BnaA02G0156900ZS	scaffoldA02	8584469	8684468	791	0.187672
BnaWGS-1007	BnaA02G0156900ZS	scaffoldA02	8594469	8694468	831	0.188485
BnaWGS-1007	BnaA02G0156900ZS	scaffoldA02	8604469	8704468	815	0.180567
BnaWGS-1007	BnaA02G0156900ZS	scaffoldA02	8614469	8714468	831	0.178174
BnaWGS-1007	BnaA02G0156900ZS	scaffoldA02	8624469	8724468	995	0.210022
BnaWGS-1007	BnaA02G0156900ZS	scaffoldA02	8634469	8734468	1059	0.230913
BnaWGS-1007	BnaA02G0156900ZS	scaffoldA02	8644469	8744468	1035	0.236161
BnaWGS-1007	BnaA02G0156900ZS	scaffoldA02	8654469	8754468	939	0.261653
BnaWGS-1007	BnaA02G0156900ZS	scaffoldA02	8664469	8764468	919	0.269668
BnaWGS-1007	BnaA02G0156900ZS	scaffoldA02	8674469	8774468	936	0.275017

For Review Only

BnaWGS-1007	BnaA02G0156900ZS	scaffoldA02	8684469	8784468	1002	0.280695
BnaWGS-1007	BnaA02G0156900ZS	scaffoldA02	8694469	8794468	1029	0.289948
BnaWGS-1007	BnaA02G0156900ZS	scaffoldA02	8704469	8804468	1015	0.294788
BnaWGS-1007	BnaA02G0156900ZS	scaffoldA02	8714469	8814468	909	0.306429
BnaWGS-1007	BnaA02G0156900ZS	scaffoldA02	8724469	8824468	870	0.29075
BnaWGS-1007	BnaA02G0156900ZS	scaffoldA02	8734469	8834468	1032	0.264476
BnaWGS-1007	BnaA02G0156900ZS	scaffoldA02	8744469	8844468	1041	0.262453
BnaWGS-1007	BnaA02G0156900ZS	scaffoldA02	8754469	8854468	1032	0.247297
BnaWGS-1007	BnaA02G0156900ZS	scaffoldA02	8764469	8864468	1045	0.246144
BnaWGS-1007	BnaA02G0156900ZS	scaffoldA02	8774469	8874468	1056	0.238361
BnaWGS-1007	BnaA02G0156900ZS	scaffoldA02	8784469	8884468	1010	0.234401
BnaWGS-1007	BnaA02G0156900ZS	scaffoldA02	8794469	8894468	957	0.232311
BnaWGS-1007	BnaA02G0156900ZS	scaffoldA02	8804469	8904468	995	0.262402
BnaWGS-1007	BnaA02G0156900ZS	scaffoldA02	8814469	8914468	1191	0.304039
BnaWGS-1007	BnaA02G0156900ZS	scaffoldA02	8824469	8924468	1134	0.312382
BnaWGS-1007	BnaA02G0156900ZS	scaffoldA02	8834469	8934468	986	0.360122
BnaWGS-1007	BnaA02G0156900ZS	scaffoldA02	8844469	8944468	1109	0.334188
BnaWGS-1007	BnaA02G0156900ZS	scaffoldA02	8854469	8954468	1267	0.332169
BnaWGS-1007	BnaA02G0156900ZS	scaffoldA02	8864469	8964468	1394	0.329504
BnaWGS-1007	BnaA02G0156900ZS	scaffoldA02	8874469	8974468	1330	0.337611
BnaWGS-1007	BnaA02G0156900ZS	scaffoldA02	8884469	8984468	1450	0.339317
BnaWGS-1007	BnaA02G0156900ZS	scaffoldA02	8894469	8994468	1517	0.341882
BnaWGS-1007	BnaA02G0156900ZS	scaffoldA02	8904469	9004468	1448	0.334392
BnaWGS-1007	BnaA02G0156900ZS	scaffoldA02	8914469	9014468	1311	0.307374
BnaWGS-1007	BnaA02G0156900ZS	scaffoldA02	8924469	9024468	1341	0.321224
BnaWGS-1007	BnaA02G0156900ZS	scaffoldA02	8934469	9034468	1254	0.324971
BnaWGS-1007	BnaA02G0156900ZS	scaffoldA02	8944469	9044468	1185	0.358154
BnaWGS-1007	BnaA02G0156900ZS	scaffoldA02	8954469	9054468	1164	0.375378
BnaWGS-1007	BnaA02G0156900ZS	scaffoldA02	8964469	9064468	1165	0.379733
BnaWGS-1007	BnaA02G0156900ZS	scaffoldA02	8974469	9074468	1335	0.350534
BnaWGS-1007	BnaA02G0156900ZS	scaffoldA02	8984469	9084468	1331	0.315919
BnaWGS-1007	BnaA02G0156900ZS	scaffoldA02	8994469	9094468	1323	0.321672
BnaWGS-1007	BnaA02G0156900ZS	scaffoldA02	9004469	9104468	1309	0.331384
BnaWGS-1007	BnaA02G0156900ZS	scaffoldA02	9014469	9114468	1298	0.337982
BnaWGS-1007	BnaA02G0156900ZS	scaffoldA02	9024469	9124468	1312	0.338393
BnaWGS-1007	BnaA02G0156900ZS	scaffoldA02	9034469	9134468	1390	0.356928
BnaWGS-1007	BnaA02G0156900ZS	scaffoldA02	9044469	9144468	1438	0.369959
BnaWGS-1007	BnaA02G0156900ZS	scaffoldA02	9054469	9154468	1364	0.372567
BnaWGS-1007	BnaA02G0156900ZS	scaffoldA02	9064469	9164468	1231	0.383607
BnaWGS-1007	BnaA02G0156900ZS	scaffoldA02	9074469	9174468	1086	0.417603
BnaWGS-1007	BnaA02G0156900ZS	scaffoldA02	9084469	9184468	958	0.468528
BnaWGS-1007	BnaA02G0156900ZS	scaffoldA02	9094469	9194468	915	0.467366
BnaWGS-1007	BnaA02G0156900ZS	scaffoldA02	9104469	9204468	1040	0.47129
BnaWGS-1007	BnaA02G0156900ZS	scaffoldA02	9114469	9214468	1079	0.471194
BnaWGS-1007	BnaA02G0156900ZS	scaffoldA02	9124469	9224468	997	0.463406
BnaWGS-1007	BnaA02G0156900ZS	scaffoldA02	9134469	9234468	871	0.447445
BnaWGS-1007	BnaA02G0156900ZS	scaffoldA02	9144469	9244468	851	0.43178
BnaWGS-1007	BnaA02G0156900ZS	scaffoldA02	9154469	9254468	923	0.39455
BnaWGS-1007	BnaA02G0156900ZS	scaffoldA02	9164469	9264468	959	0.376707
BnaWGS-1007	BnaA02G0156900ZS	scaffoldA02	9174469	9274468	937	0.361294
BnaWGS-1007	BnaA02G0156900ZS	scaffoldA02	9184469	9284468	945	0.351014
BnaWGS-1007	BnaA02G0156900ZS	scaffoldA02	9194469	9294468	959	0.331689
BnaWGS-1007	BnaA02G0156900ZS	scaffoldA02	9204469	9304468	922	0.24263
BnaWGS-1007	BnaA02G0156900ZS	scaffoldA02	9214469	9314468	822	0.185872
BnaWGS-1007	BnaA02G0156900ZS	scaffoldA02	9224469	9324468	855	0.186896
BnaWGS-1007	BnaA02G0156900ZS	scaffoldA02	9234469	9334468	933	0.176706
BnaWGS-1007	BnaA02G0156900ZS	scaffoldA02	9244469	9344468	868	0.121881
BnaWGS-1007	BnaA02G0156900ZS	scaffoldA02	9254469	9354468	814	0.06603
BnaWGS-1007	BnaA02G0156900ZS	scaffoldA02	9264469	9364468	800	0.055282
BnaWGS-1007	BnaA02G0156900ZS	scaffoldA02	9274469	9374468	879	0.052027

For Review Only

BnaWGS-1007	BnaA02G0156900ZS	scaffoldA02	9284469	9384468	987	0.049223
BnaWGS-1007	BnaA02G0156900ZS	scaffoldA02	9294469	9394468	979	0.042061
BnaWGS-1007	BnaA02G0156900ZS	scaffoldA02	9304469	9404468	946	0.042862
BnaWGS-1007	BnaA02G0156900ZS	scaffoldA02	9314469	9414468	1028	0.039373
BnaWGS-1007	BnaA02G0156900ZS	scaffoldA02	9324469	9424468	1135	0.03318
BnaWGS-1007	BnaA02G0156900ZS	scaffoldA02	9334469	9434468	1082	0.032466
BnaWGS-1007	BnaA02G0156900ZS	scaffoldA02	9344469	9444468	1154	0.055827
BnaWGS-1007	BnaA02G0156900ZS	scaffoldA02	9354469	9454468	1109	0.056001
BnaWGS-1007	BnaA02G0156900ZS	scaffoldA02	9364469	9464468	1105	0.049532
BnaWGS-1007	BnaA02G0156900ZS	scaffoldA02	9374469	9474468	1014	0.05121
BnaWGS-1007	BnaA02G0156900ZS	scaffoldA02	9384469	9484468	970	0.046783
BnaWGS-1007	BnaA02G0156900ZS	scaffoldA02	9394469	9494468	1008	0.046485
BnaWGS-1007	BnaA02G0156900ZS	scaffoldA02	9404469	9504468	1012	0.06089
BnaWGS-1007	BnaA02G0156900ZS	scaffoldA02	9414469	9514468	902	0.064959
BnaWGS-1007	BnaA02G0156900ZS	scaffoldA02	9424469	9524468	746	0.101997
BnaWGS-1007	BnaA02G0156900ZS	scaffoldA02	9434469	9534468	889	0.224338
BnaWGS-1007	BnaA02G0156900ZS	scaffoldA02	9444469	9544468	856	0.262178
BnaWGS-1007	BnaA02G0156900ZS	scaffoldA02	9454469	9554468	888	0.319498
BnaWGS-1007	BnaA02G0156900ZS	scaffoldA02	9464469	9564468	892	0.336106
BnaWGS-1007	BnaA02G0156900ZS	scaffoldA02	9474469	9574468	999	0.348738
BnaWGS-1007	BnaA02G0156900ZS	scaffoldA02	9484469	9584468	910	0.364897
BnaWGS-1007	BnaA02G0156900ZS	scaffoldA02	9494469	9594468	914	0.365439
BnaWGS-1007	BnaA02G0156900ZS	scaffoldA02	9504469	9604468	953	0.361668
BnaWGS-1007	BnaA02G0156900ZS	scaffoldA02	9514469	9614468	964	0.360085
BnaWGS-1007	BnaA02G0156900ZS	scaffoldA02	9524469	9624468	957	0.360134
BnaWGS-1007	BnaA02G0156900ZS	scaffoldA02	9534469	9634468	804	0.339663
BnaWGS-1007	BnaA02G0156900ZS	scaffoldA02	9544469	9644468	790	0.312032
BnaWGS-1007	BnaA02G0156900ZS	scaffoldA02	9554469	9654468	903	0.237847
BnaWGS-1007	BnaA02G0156900ZS	scaffoldA02	9564469	9664468	913	0.22383
BnaWGS-1007	BnaA02G0156900ZS	scaffoldA02	9574469	9674468	808	0.212801
BnaWGS-1007	BnaA02G0156900ZS	scaffoldA02	9584469	9684468	847	0.234757
BnaWGS-1007	BnaA02G0156900ZS	scaffoldA02	9594469	9694468	796	0.258739
BnaWGS-1007	BnaA02G0156900ZS	scaffoldA02	9604469	9704468	730	0.277436
BnaWGS-1007	BnaA02G0156900ZS	scaffoldA02	9614469	9714468	868	0.32761
BnaWGS-1007	BnaA02G0156900ZS	scaffoldA02	9624469	9724468	906	0.34091
BnaWGS-1007	BnaA02G0156900ZS	scaffoldA02	9634469	9734468	1026	0.369264
BnaWGS-1007	BnaA02G0156900ZS	scaffoldA02	9644469	9744468	954	0.387248
BnaWGS-1007	BnaA02G0156900ZS	scaffoldA02	9654469	9754468	759	0.433569
BnaWGS-1007	BnaA02G0156900ZS	scaffoldA02	9664469	9764468	687	0.447738
BnaWGS-1007	BnaA02G0156900ZS	scaffoldA02	9674469	9774468	760	0.435748
BnaWGS-1007	BnaA02G0156900ZS	scaffoldA02	9684469	9784468	797	0.42633
BnaWGS-1007	BnaA02G0156900ZS	scaffoldA02	9694469	9794468	863	0.418629
BnaWGS-1007	BnaA02G0156900ZS	scaffoldA02	9704469	9804468	882	0.417581
BnaWGS-1007	BnaA02G0156900ZS	scaffoldA02	9714469	9814468	875	0.408611
BnaWGS-1007	BnaA02G0156900ZS	scaffoldA02	9724469	9824468	935	0.39486
BnaWGS-1007	BnaA02G0156900ZS	scaffoldA02	9734469	9834468	842	0.38484
BnaWGS-1007	BnaA02G0156900ZS	scaffoldA02	9744469	9844468	874	0.378165
BnaWGS-1007	BnaA02G0156900ZS	scaffoldA02	9754469	9854468	978	0.389165
BnaWGS-1007	BnaA02G0156900ZS	scaffoldA02	9764469	9864468	1116	0.386733
BnaWGS-1007	BnaA02G0156900ZS	scaffoldA02	9774469	9874468	1197	0.35844
BnaWGS-1007	BnaA02G0156900ZS	scaffoldA02	9784469	9884468	1299	0.320845
BnaWGS-1007	BnaA02G0156900ZS	scaffoldA02	9794469	9894468	1316	0.309
BnaWGS-1007	BnaA02G0156900ZS	scaffoldA02	9804469	9904468	1301	0.293923
BnaWGS-1007	BnaA02G0156900ZS	scaffoldA02	9814469	9914468	1328	0.286839
BnaWGS-1007	BnaA02G0156900ZS	scaffoldA02	9824469	9924468	1346	0.306352
BnaWGS-1007	BnaA02G0156900ZS	scaffoldA02	9834469	9934468	1303	0.305846
BnaWGS-1007	BnaA02G0156900ZS	scaffoldA02	9844469	9944468	1366	0.322288
BnaWGS-1007	BnaA02G0156900ZS	scaffoldA02	9854469	9954468	1299	0.311529
BnaWGS-1007	BnaA02G0156900ZS	scaffoldA02	9864469	9964468	1210	0.308019
BnaWGS-1007	BnaA02G0156900ZS	scaffoldA02	9874469	9974468	1185	0.322297

For Review Only

BnaWGS-1007	BnaA02G0156900ZS	scaffoldA02	9884469	9984468	1061	0.368947
BnaWGS-1007	BnaA02G0156900ZS	scaffoldA02	9894469	9994468	1110	0.374004
BnaWGS-1007	BnaA02G0156900ZS	scaffoldA02	9904469	10004468	1145	0.384415
BnaWGS-1007	BnaA02G0156900ZS	scaffoldA02	9914469	10014468	1181	0.36211
BnaWGS-1007	BnaA02G0156900ZS	scaffoldA02	9924469	10024468	1077	0.352202
BnaWGS-1007	BnaA02G0156900ZS	scaffoldA02	9934469	10034468	1104	0.355176
BnaWGS-1007	BnaA02G0156900ZS	scaffoldA02	9944469	10044468	1152	0.35585
BnaWGS-1007	BnaA02G0156900ZS	scaffoldA02	9954469	10054468	1225	0.376893
BnaWGS-1007	BnaA02G0156900ZS	scaffoldA02	9964469	10064468	1247	0.385952
BnaWGS-1007	BnaA02G0156900ZS	scaffoldA02	9974469	10074468	1139	0.398724
BnaWGS-1007	BnaA02G0156900ZS	scaffoldA02	9984469	10084468	1125	0.393223
BnaWGS-1007	BnaA02G0156900ZS	scaffoldA02	9994469	10094468	1046	0.403246
BnaWGS-1007	BnaA02G0156900ZS	scaffoldA02	10004469	10104468	1001	0.403678
BnaWGS-1007	BnaA02G0156900ZS	scaffoldA02	10014469	10107241	823	0.428295
BnaWGS-1007	BnaA02G0156900ZS	scaffoldA02	8104469	8204468	1376	0.279981
BnaWGS-1007	BnaA02G0156900ZS	scaffoldA02	8114469	8214468	1411	0.298831
BnaWGS-1007	BnaA02G0156900ZS	scaffoldA02	8124469	8224468	1391	0.293182
BnaWGS-1007	BnaA02G0156900ZS	scaffoldA02	8134469	8234468	1206	0.299382
BnaWGS-1007	BnaA02G0156900ZS	scaffoldA02	8144469	8244468	1278	0.301067
BnaWGS-1007	BnaA02G0156900ZS	scaffoldA02	8154469	8254468	1239	0.294739
BnaWGS-1007	BnaA02G0156900ZS	scaffoldA02	8164469	8264468	1133	0.30457
BnaWGS-1007	BnaA02G0156900ZS	scaffoldA02	8174469	8274468	1097	0.301225
BnaWGS-1007	BnaA02G0156900ZS	scaffoldA02	8184469	8284468	1121	0.297704
BnaWGS-1007	BnaA02G0156900ZS	scaffoldA02	8194469	8294468	1049	0.27931
BnaWGS-1007	BnaA02G0156900ZS	scaffoldA02	8204469	8304468	1058	0.292137
BnaWGS-1007	BnaA02G0156900ZS	scaffoldA02	8214469	8314468	973	0.273866
BnaWGS-1007	BnaA02G0156900ZS	scaffoldA02	8224469	8324468	971	0.295138
BnaWGS-1007	BnaA02G0156900ZS	scaffoldA02	8234469	8334468	1034	0.293018
BnaWGS-1007	BnaA02G0156900ZS	scaffoldA02	8244469	8344468	941	0.29688
BnaWGS-1007	BnaA02G0156900ZS	scaffoldA02	8254469	8354468	898	0.324845
BnaWGS-1007	BnaA02G0156900ZS	scaffoldA02	8264469	8364468	1024	0.322147
BnaWGS-1007	BnaA02G0156900ZS	scaffoldA02	8274469	8374468	1058	0.323774
BnaWGS-1007	BnaA02G0156900ZS	scaffoldA02	8284469	8384468	1052	0.314152
BnaWGS-1007	BnaA02G0156900ZS	scaffoldA02	8294469	8394468	1001	0.317966
BnaWGS-1007	BnaA02G0156900ZS	scaffoldA02	8304469	8404468	934	0.304025
BnaWGS-1007	BnaA02G0156900ZS	scaffoldA02	8314469	8414468	914	0.29489
BnaWGS-1007	BnaA02G0156900ZS	scaffoldA02	8324469	8424468	964	0.279044
BnaWGS-1007	BnaA02G0156900ZS	scaffoldA02	8334469	8434468	935	0.287484
BnaWGS-1007	BnaA02G0156900ZS	scaffoldA02	8344469	8444468	858	0.301039
BnaWGS-1007	BnaA02G0156900ZS	scaffoldA02	8354469	8454468	831	0.345216
BnaWGS-1007	BnaA02G0156900ZS	scaffoldA02	8364469	8464468	648	0.374178
BnaWGS-1007	BnaA02G0156900ZS	scaffoldA02	8374469	8474468	545	0.397484
BnaWGS-1007	BnaA02G0156900ZS	scaffoldA02	8384469	8484468	455	0.420454
BnaWGS-1007	BnaA02G0156900ZS	scaffoldA02	8394469	8494468	432	0.421428
BnaWGS-1007	BnaA02G0156900ZS	scaffoldA02	8404469	8504468	420	0.419269
BnaWGS-1007	BnaA02G0156900ZS	scaffoldA02	8414469	8514468	354	0.428756
BnaWGS-1007	BnaA02G0156900ZS	scaffoldA02	8424469	8524468	196	0.491072
BnaWGS-1007	BnaA02G0156900ZS	scaffoldA02	8434469	8534468	122	0.549122
BnaWGS-1007	BnaA02G0156900ZS	scaffoldA02	8444469	8544468	87	0.596567
BnaWGS-1007	BnaA02G0156900ZS	scaffoldA02	8454469	8554468	38	0.358885
BnaWGS-1007	BnaA02G0156900ZS	scaffoldA02	8464469	8564468	80	0.332588
BnaWGS-1007	BnaA02G0156900ZS	scaffoldA02	8474469	8574468	149	0.304097
BnaWGS-1007	BnaA02G0156900ZS	scaffoldA02	8484469	8584468	160	0.312557
BnaWGS-1007	BnaA02G0156900ZS	scaffoldA02	8494469	8594468	190	0.388426
BnaWGS-1007	BnaA02G0156900ZS	scaffoldA02	8504469	8604468	312	0.40858
BnaWGS-1007	BnaA02G0156900ZS	scaffoldA02	8514469	8614468	444	0.402199
BnaWGS-1007	BnaA02G0156900ZS	scaffoldA02	8524469	8624468	468	0.393598
BnaWGS-1007	BnaA02G0156900ZS	scaffoldA02	8534469	8634468	579	0.361461
BnaWGS-1007	BnaA02G0156900ZS	scaffoldA02	8544469	8644468	646	0.345291
BnaWGS-1007	BnaA02G0156900ZS	scaffoldA02	8554469	8654468	756	0.311911

For Review Only

BnaWGS-1007	BnaA02G0156900ZS	scaffoldA02	8564469	8664468	770	0.305076
BnaWGS-1007	BnaA02G0156900ZS	scaffoldA02	8574469	8674468	778	0.308303
BnaWGS-1007	BnaA02G0156900ZS	scaffoldA02	8584469	8684468	791	0.312958
BnaWGS-1007	BnaA02G0156900ZS	scaffoldA02	8594469	8694468	831	0.309972
BnaWGS-1007	BnaA02G0156900ZS	scaffoldA02	8604469	8704468	815	0.286421
BnaWGS-1007	BnaA02G0156900ZS	scaffoldA02	8614469	8714468	831	0.264354
BnaWGS-1007	BnaA02G0156900ZS	scaffoldA02	8624469	8724468	995	0.272848
BnaWGS-1007	BnaA02G0156900ZS	scaffoldA02	8634469	8734468	1059	0.278681
BnaWGS-1007	BnaA02G0156900ZS	scaffoldA02	8644469	8744468	1035	0.287545
BnaWGS-1007	BnaA02G0156900ZS	scaffoldA02	8654469	8754468	939	0.312952
BnaWGS-1007	BnaA02G0156900ZS	scaffoldA02	8664469	8764468	919	0.320111
BnaWGS-1007	BnaA02G0156900ZS	scaffoldA02	8674469	8774468	936	0.32636
BnaWGS-1007	BnaA02G0156900ZS	scaffoldA02	8684469	8784468	1002	0.313773
BnaWGS-1007	BnaA02G0156900ZS	scaffoldA02	8694469	8794468	1029	0.298779
BnaWGS-1007	BnaA02G0156900ZS	scaffoldA02	8704469	8804468	1015	0.314857
BnaWGS-1007	BnaA02G0156900ZS	scaffoldA02	8714469	8814468	909	0.323515
BnaWGS-1007	BnaA02G0156900ZS	scaffoldA02	8724469	8824468	870	0.319247
BnaWGS-1007	BnaA02G0156900ZS	scaffoldA02	8734469	8834468	1032	0.271028
BnaWGS-1007	BnaA02G0156900ZS	scaffoldA02	8744469	8844468	1041	0.269248
BnaWGS-1007	BnaA02G0156900ZS	scaffoldA02	8754469	8854468	1032	0.256254
BnaWGS-1007	BnaA02G0156900ZS	scaffoldA02	8764469	8864468	1045	0.249368
BnaWGS-1007	BnaA02G0156900ZS	scaffoldA02	8774469	8874468	1056	0.232781
BnaWGS-1007	BnaA02G0156900ZS	scaffoldA02	8784469	8884468	1010	0.233117
BnaWGS-1007	BnaA02G0156900ZS	scaffoldA02	8794469	8894468	957	0.238561
BnaWGS-1007	BnaA02G0156900ZS	scaffoldA02	8804469	8904468	995	0.213888
BnaWGS-1007	BnaA02G0156900ZS	scaffoldA02	8814469	8914468	1191	0.209198
BnaWGS-1007	BnaA02G0156900ZS	scaffoldA02	8824469	8924468	1134	0.227601
BnaWGS-1007	BnaA02G0156900ZS	scaffoldA02	8834469	8934468	986	0.306028
BnaWGS-1007	BnaA02G0156900ZS	scaffoldA02	8844469	8944468	1109	0.302531
BnaWGS-1007	BnaA02G0156900ZS	scaffoldA02	8854469	8954468	1267	0.324568
BnaWGS-1007	BnaA02G0156900ZS	scaffoldA02	8864469	8964468	1394	0.346985
BnaWGS-1007	BnaA02G0156900ZS	scaffoldA02	8874469	8974468	1330	0.357183
BnaWGS-1007	BnaA02G0156900ZS	scaffoldA02	8884469	8984468	1450	0.368502
BnaWGS-1007	BnaA02G0156900ZS	scaffoldA02	8894469	8994468	1517	0.363142
BnaWGS-1007	BnaA02G0156900ZS	scaffoldA02	8904469	9004468	1448	0.373228
BnaWGS-1007	BnaA02G0156900ZS	scaffoldA02	8914469	9014468	1311	0.400964
BnaWGS-1007	BnaA02G0156900ZS	scaffoldA02	8924469	9024468	1341	0.384966
BnaWGS-1007	BnaA02G0156900ZS	scaffoldA02	8934469	9034468	1254	0.375614
BnaWGS-1007	BnaA02G0156900ZS	scaffoldA02	8944469	9044468	1185	0.381137
BnaWGS-1007	BnaA02G0156900ZS	scaffoldA02	8954469	9054468	1164	0.346883
BnaWGS-1007	BnaA02G0156900ZS	scaffoldA02	8964469	9064468	1165	0.296707
BnaWGS-1007	BnaA02G0156900ZS	scaffoldA02	8974469	9074468	1335	0.259973
BnaWGS-1007	BnaA02G0156900ZS	scaffoldA02	8984469	9084468	1331	0.211548
BnaWGS-1007	BnaA02G0156900ZS	scaffoldA02	8994469	9094468	1323	0.195454
BnaWGS-1007	BnaA02G0156900ZS	scaffoldA02	9004469	9104468	1309	0.185269
BnaWGS-1007	BnaA02G0156900ZS	scaffoldA02	9014469	9114468	1298	0.160033
BnaWGS-1007	BnaA02G0156900ZS	scaffoldA02	9024469	9124468	1312	0.143194
BnaWGS-1007	BnaA02G0156900ZS	scaffoldA02	9034469	9134468	1390	0.116641
BnaWGS-1007	BnaA02G0156900ZS	scaffoldA02	9044469	9144468	1438	0.099009
BnaWGS-1007	BnaA02G0156900ZS	scaffoldA02	9054469	9154468	1364	0.088609
BnaWGS-1007	BnaA02G0156900ZS	scaffoldA02	9064469	9164468	1231	0.090377
BnaWGS-1007	BnaA02G0156900ZS	scaffoldA02	9074469	9174468	1086	0.089016
BnaWGS-1007	BnaA02G0156900ZS	scaffoldA02	9084469	9184468	958	0.092338
BnaWGS-1007	BnaA02G0156900ZS	scaffoldA02	9094469	9194468	915	0.088675
BnaWGS-1007	BnaA02G0156900ZS	scaffoldA02	9104469	9204468	1040	0.115864
BnaWGS-1007	BnaA02G0156900ZS	scaffoldA02	9114469	9214468	1079	0.129842
BnaWGS-1007	BnaA02G0156900ZS	scaffoldA02	9124469	9224468	997	0.139661
BnaWGS-1007	BnaA02G0156900ZS	scaffoldA02	9134469	9234468	871	0.176543
BnaWGS-1007	BnaA02G0156900ZS	scaffoldA02	9144469	9244468	851	0.204907
BnaWGS-1007	BnaA02G0156900ZS	scaffoldA02	9154469	9254468	923	0.245428

For Review Only

BnaWGS-1007	BnaA02G0156900ZS	scaffoldA02	9164469	9264468	959	0.2637
BnaWGS-1007	BnaA02G0156900ZS	scaffoldA02	9174469	9274468	937	0.295789
BnaWGS-1007	BnaA02G0156900ZS	scaffoldA02	9184469	9284468	945	0.323851
BnaWGS-1007	BnaA02G0156900ZS	scaffoldA02	9194469	9294468	959	0.35868
BnaWGS-1007	BnaA02G0156900ZS	scaffoldA02	9204469	9304468	922	0.419496
BnaWGS-1007	BnaA02G0156900ZS	scaffoldA02	9214469	9314468	822	0.447239
BnaWGS-1007	BnaA02G0156900ZS	scaffoldA02	9224469	9324468	855	0.492266
BnaWGS-1007	BnaA02G0156900ZS	scaffoldA02	9234469	9334468	933	0.500913
BnaWGS-1007	BnaA02G0156900ZS	scaffoldA02	9244469	9344468	868	0.525134
BnaWGS-1007	BnaA02G0156900ZS	scaffoldA02	9254469	9354468	814	0.551852
BnaWGS-1007	BnaA02G0156900ZS	scaffoldA02	9264469	9364468	800	0.540479
BnaWGS-1007	BnaA02G0156900ZS	scaffoldA02	9274469	9374468	879	0.528676
BnaWGS-1007	BnaA02G0156900ZS	scaffoldA02	9284469	9384468	987	0.524946
BnaWGS-1007	BnaA02G0156900ZS	scaffoldA02	9294469	9394468	979	0.520291
BnaWGS-1007	BnaA02G0156900ZS	scaffoldA02	9304469	9404468	946	0.50936
BnaWGS-1007	BnaA02G0156900ZS	scaffoldA02	9314469	9414468	1028	0.500568
BnaWGS-1007	BnaA02G0156900ZS	scaffoldA02	9324469	9424468	1135	0.453896
BnaWGS-1007	BnaA02G0156900ZS	scaffoldA02	9334469	9434468	1082	0.445782
BnaWGS-1007	BnaA02G0156900ZS	scaffoldA02	9344469	9444468	1154	0.422666
BnaWGS-1007	BnaA02G0156900ZS	scaffoldA02	9354469	9454468	1109	0.410224
BnaWGS-1007	BnaA02G0156900ZS	scaffoldA02	9364469	9464468	1105	0.40434
BnaWGS-1007	BnaA02G0156900ZS	scaffoldA02	9374469	9474468	1014	0.388059
BnaWGS-1007	BnaA02G0156900ZS	scaffoldA02	9384469	9484468	970	0.34748
BnaWGS-1007	BnaA02G0156900ZS	scaffoldA02	9394469	9494468	1008	0.318579
BnaWGS-1007	BnaA02G0156900ZS	scaffoldA02	9404469	9504468	1012	0.290606
BnaWGS-1007	BnaA02G0156900ZS	scaffoldA02	9414469	9514468	902	0.268866
BnaWGS-1007	BnaA02G0156900ZS	scaffoldA02	9424469	9524468	746	0.223242
BnaWGS-1007	BnaA02G0156900ZS	scaffoldA02	9434469	9534468	889	0.180546
BnaWGS-1007	BnaA02G0156900ZS	scaffoldA02	9444469	9544468	856	0.199012
BnaWGS-1007	BnaA02G0156900ZS	scaffoldA02	9454469	9554468	888	0.232636
BnaWGS-1007	BnaA02G0156900ZS	scaffoldA02	9464469	9564468	892	0.24944
BnaWGS-1007	BnaA02G0156900ZS	scaffoldA02	9474469	9574468	999	0.256085
BnaWGS-1007	BnaA02G0156900ZS	scaffoldA02	9484469	9584468	910	0.261763
BnaWGS-1007	BnaA02G0156900ZS	scaffoldA02	9494469	9594468	914	0.267347
BnaWGS-1007	BnaA02G0156900ZS	scaffoldA02	9504469	9604468	953	0.269909
BnaWGS-1007	BnaA02G0156900ZS	scaffoldA02	9514469	9614468	964	0.268795
BnaWGS-1007	BnaA02G0156900ZS	scaffoldA02	9524469	9624468	957	0.276244
BnaWGS-1007	BnaA02G0156900ZS	scaffoldA02	9534469	9634468	804	0.297531
BnaWGS-1007	BnaA02G0156900ZS	scaffoldA02	9544469	9644468	790	0.277413
BnaWGS-1007	BnaA02G0156900ZS	scaffoldA02	9554469	9654468	903	0.241538
BnaWGS-1007	BnaA02G0156900ZS	scaffoldA02	9564469	9664468	913	0.228399
BnaWGS-1007	BnaA02G0156900ZS	scaffoldA02	9574469	9674468	808	0.213232
BnaWGS-1007	BnaA02G0156900ZS	scaffoldA02	9584469	9684468	847	0.220541
BnaWGS-1007	BnaA02G0156900ZS	scaffoldA02	9594469	9694468	796	0.237858
BnaWGS-1007	BnaA02G0156900ZS	scaffoldA02	9604469	9704468	730	0.251775
BnaWGS-1007	BnaA02G0156900ZS	scaffoldA02	9614469	9714468	868	0.288591
BnaWGS-1007	BnaA02G0156900ZS	scaffoldA02	9624469	9724468	906	0.297775
BnaWGS-1007	BnaA02G0156900ZS	scaffoldA02	9634469	9734468	1026	0.323552
BnaWGS-1007	BnaA02G0156900ZS	scaffoldA02	9644469	9744468	954	0.337716
BnaWGS-1007	BnaA02G0156900ZS	scaffoldA02	9654469	9754468	759	0.367416
BnaWGS-1007	BnaA02G0156900ZS	scaffoldA02	9664469	9764468	687	0.376547
BnaWGS-1007	BnaA02G0156900ZS	scaffoldA02	9674469	9774468	760	0.377688
BnaWGS-1007	BnaA02G0156900ZS	scaffoldA02	9684469	9784468	797	0.354898
BnaWGS-1007	BnaA02G0156900ZS	scaffoldA02	9694469	9794468	863	0.347738
BnaWGS-1007	BnaA02G0156900ZS	scaffoldA02	9704469	9804468	882	0.34774
BnaWGS-1007	BnaA02G0156900ZS	scaffoldA02	9714469	9814468	875	0.326158
BnaWGS-1007	BnaA02G0156900ZS	scaffoldA02	9724469	9824468	935	0.326593
BnaWGS-1007	BnaA02G0156900ZS	scaffoldA02	9734469	9834468	842	0.309402
BnaWGS-1007	BnaA02G0156900ZS	scaffoldA02	9744469	9844468	874	0.31338
BnaWGS-1007	BnaA02G0156900ZS	scaffoldA02	9754469	9854468	978	0.316936

For Review Only

BnaWGS-1007	BnaA02G0156900ZS	scaffoldA02	9764469	9864468	1116	0.322144
BnaWGS-1007	BnaA02G0156900ZS	scaffoldA02	9774469	9874468	1197	0.316887
BnaWGS-1007	BnaA02G0156900ZS	scaffoldA02	9784469	9884468	1299	0.317125
BnaWGS-1007	BnaA02G0156900ZS	scaffoldA02	9794469	9894468	1316	0.33111
BnaWGS-1007	BnaA02G0156900ZS	scaffoldA02	9804469	9904468	1301	0.344433
BnaWGS-1007	BnaA02G0156900ZS	scaffoldA02	9814469	9914468	1328	0.376996
BnaWGS-1007	BnaA02G0156900ZS	scaffoldA02	9824469	9924468	1346	0.374015
BnaWGS-1007	BnaA02G0156900ZS	scaffoldA02	9834469	9934468	1303	0.367041
BnaWGS-1007	BnaA02G0156900ZS	scaffoldA02	9844469	9944468	1366	0.354904
BnaWGS-1007	BnaA02G0156900ZS	scaffoldA02	9854469	9954468	1299	0.358527
BnaWGS-1007	BnaA02G0156900ZS	scaffoldA02	9864469	9964468	1210	0.355377
BnaWGS-1007	BnaA02G0156900ZS	scaffoldA02	9874469	9974468	1185	0.38081
BnaWGS-1007	BnaA02G0156900ZS	scaffoldA02	9884469	9984468	1061	0.407265
BnaWGS-1007	BnaA02G0156900ZS	scaffoldA02	9894469	9994468	1110	0.391718
BnaWGS-1007	BnaA02G0156900ZS	scaffoldA02	9904469	10004468	1145	0.372554
BnaWGS-1007	BnaA02G0156900ZS	scaffoldA02	9914469	10014468	1181	0.324204
BnaWGS-1007	BnaA02G0156900ZS	scaffoldA02	9924469	10024468	1077	0.320381
BnaWGS-1007	BnaA02G0156900ZS	scaffoldA02	9934469	10034468	1104	0.330211
BnaWGS-1007	BnaA02G0156900ZS	scaffoldA02	9944469	10044468	1152	0.31812
BnaWGS-1007	BnaA02G0156900ZS	scaffoldA02	9954469	10054468	1225	0.329477
BnaWGS-1007	BnaA02G0156900ZS	scaffoldA02	9964469	10064468	1247	0.337167
BnaWGS-1007	BnaA02G0156900ZS	scaffoldA02	9974469	10074468	1139	0.327884
BnaWGS-1007	BnaA02G0156900ZS	scaffoldA02	9984469	10084468	1125	0.325055
BnaWGS-1007	BnaA02G0156900ZS	scaffoldA02	9994469	10094468	1046	0.315587
BnaWGS-1007	BnaA02G0156900ZS	scaffoldA02	10004469	10104468	1001	0.320572
BnaWGS-1007	BnaA02G0156900ZS	scaffoldA02	10014469	10107241	823	0.352843
BnaWGS-1007	BnaA02G0156900ZS	scaffoldA02	8104469	8204468	1376	0.25782
BnaWGS-1007	BnaA02G0156900ZS	scaffoldA02	8114469	8214468	1411	0.268479
BnaWGS-1007	BnaA02G0156900ZS	scaffoldA02	8124469	8224468	1391	0.263658
BnaWGS-1007	BnaA02G0156900ZS	scaffoldA02	8134469	8234468	1206	0.256869
BnaWGS-1007	BnaA02G0156900ZS	scaffoldA02	8144469	8244468	1278	0.221895
BnaWGS-1007	BnaA02G0156900ZS	scaffoldA02	8154469	8254468	1239	0.2072
BnaWGS-1007	BnaA02G0156900ZS	scaffoldA02	8164469	8264468	1133	0.222885
BnaWGS-1007	BnaA02G0156900ZS	scaffoldA02	8174469	8274468	1097	0.213689
BnaWGS-1007	BnaA02G0156900ZS	scaffoldA02	8184469	8284468	1121	0.200447
BnaWGS-1007	BnaA02G0156900ZS	scaffoldA02	8194469	8294468	1049	0.178944
BnaWGS-1007	BnaA02G0156900ZS	scaffoldA02	8204469	8304468	1058	0.173109
BnaWGS-1007	BnaA02G0156900ZS	scaffoldA02	8214469	8314468	973	0.140346
BnaWGS-1007	BnaA02G0156900ZS	scaffoldA02	8224469	8324468	971	0.137383
BnaWGS-1007	BnaA02G0156900ZS	scaffoldA02	8234469	8334468	1034	0.140443
BnaWGS-1007	BnaA02G0156900ZS	scaffoldA02	8244469	8344468	941	0.146436
BnaWGS-1007	BnaA02G0156900ZS	scaffoldA02	8254469	8354468	898	0.136581
BnaWGS-1007	BnaA02G0156900ZS	scaffoldA02	8264469	8364468	1024	0.102372
BnaWGS-1007	BnaA02G0156900ZS	scaffoldA02	8274469	8374468	1058	0.089003
BnaWGS-1007	BnaA02G0156900ZS	scaffoldA02	8284469	8384468	1052	0.087811
BnaWGS-1007	BnaA02G0156900ZS	scaffoldA02	8294469	8394468	1001	0.089793
BnaWGS-1007	BnaA02G0156900ZS	scaffoldA02	8304469	8404468	934	0.084502
BnaWGS-1007	BnaA02G0156900ZS	scaffoldA02	8314469	8414468	914	0.078374
BnaWGS-1007	BnaA02G0156900ZS	scaffoldA02	8324469	8424468	964	0.076279
BnaWGS-1007	BnaA02G0156900ZS	scaffoldA02	8334469	8434468	935	0.077001
BnaWGS-1007	BnaA02G0156900ZS	scaffoldA02	8344469	8444468	858	0.082138
BnaWGS-1007	BnaA02G0156900ZS	scaffoldA02	8354469	8454468	831	0.127996
BnaWGS-1007	BnaA02G0156900ZS	scaffoldA02	8364469	8464468	648	0.169527
BnaWGS-1007	BnaA02G0156900ZS	scaffoldA02	8374469	8474468	545	0.197602
BnaWGS-1007	BnaA02G0156900ZS	scaffoldA02	8384469	8484468	455	0.210438
BnaWGS-1007	BnaA02G0156900ZS	scaffoldA02	8394469	8494468	432	0.213176
BnaWGS-1007	BnaA02G0156900ZS	scaffoldA02	8404469	8504468	420	0.212437
BnaWGS-1007	BnaA02G0156900ZS	scaffoldA02	8414469	8514468	354	0.227637
BnaWGS-1007	BnaA02G0156900ZS	scaffoldA02	8424469	8524468	196	0.274553
BnaWGS-1007	BnaA02G0156900ZS	scaffoldA02	8434469	8534468	122	0.313309

For Review Only

BnaWGS-1007	BnaA02G0156900ZS	scaffoldA02	8444469	8544468	87	0.355002
BnaWGS-1007	BnaA02G0156900ZS	scaffoldA02	8454469	8554468	38	0.124319
BnaWGS-1007	BnaA02G0156900ZS	scaffoldA02	8464469	8564468	80	0.084291
BnaWGS-1007	BnaA02G0156900ZS	scaffoldA02	8474469	8574468	149	0.048995
BnaWGS-1007	BnaA02G0156900ZS	scaffoldA02	8484469	8584468	160	0.045718
BnaWGS-1007	BnaA02G0156900ZS	scaffoldA02	8494469	8594468	190	0.081212
BnaWGS-1007	BnaA02G0156900ZS	scaffoldA02	8504469	8604468	312	0.085629
BnaWGS-1007	BnaA02G0156900ZS	scaffoldA02	8514469	8614468	444	0.083747
BnaWGS-1007	BnaA02G0156900ZS	scaffoldA02	8524469	8624468	468	0.098371
BnaWGS-1007	BnaA02G0156900ZS	scaffoldA02	8534469	8634468	579	0.096033
BnaWGS-1007	BnaA02G0156900ZS	scaffoldA02	8544469	8644468	646	0.097995
BnaWGS-1007	BnaA02G0156900ZS	scaffoldA02	8554469	8654468	756	0.103996
BnaWGS-1007	BnaA02G0156900ZS	scaffoldA02	8564469	8664468	770	0.103402
BnaWGS-1007	BnaA02G0156900ZS	scaffoldA02	8574469	8674468	778	0.112268
BnaWGS-1007	BnaA02G0156900ZS	scaffoldA02	8584469	8684468	791	0.114472
BnaWGS-1007	BnaA02G0156900ZS	scaffoldA02	8594469	8694468	831	0.110917
BnaWGS-1007	BnaA02G0156900ZS	scaffoldA02	8604469	8704468	815	0.122904
BnaWGS-1007	BnaA02G0156900ZS	scaffoldA02	8614469	8714468	831	0.147068
BnaWGS-1007	BnaA02G0156900ZS	scaffoldA02	8624469	8724468	995	0.150318
BnaWGS-1007	BnaA02G0156900ZS	scaffoldA02	8634469	8734468	1059	0.14837
BnaWGS-1007	BnaA02G0156900ZS	scaffoldA02	8644469	8744468	1035	0.15481
BnaWGS-1007	BnaA02G0156900ZS	scaffoldA02	8654469	8754468	939	0.151164
BnaWGS-1007	BnaA02G0156900ZS	scaffoldA02	8664469	8764468	919	0.151555
BnaWGS-1007	BnaA02G0156900ZS	scaffoldA02	8674469	8774468	936	0.142207
BnaWGS-1007	BnaA02G0156900ZS	scaffoldA02	8684469	8784468	1002	0.14893
BnaWGS-1007	BnaA02G0156900ZS	scaffoldA02	8694469	8794468	1029	0.162366
BnaWGS-1007	BnaA02G0156900ZS	scaffoldA02	8704469	8804468	1015	0.179875
BnaWGS-1007	BnaA02G0156900ZS	scaffoldA02	8714469	8814468	909	0.182017
BnaWGS-1007	BnaA02G0156900ZS	scaffoldA02	8724469	8824468	870	0.16311
BnaWGS-1007	BnaA02G0156900ZS	scaffoldA02	8734469	8834468	1032	0.157564
BnaWGS-1007	BnaA02G0156900ZS	scaffoldA02	8744469	8844468	1041	0.154597
BnaWGS-1007	BnaA02G0156900ZS	scaffoldA02	8754469	8854468	1032	0.159024
BnaWGS-1007	BnaA02G0156900ZS	scaffoldA02	8764469	8864468	1045	0.159579
BnaWGS-1007	BnaA02G0156900ZS	scaffoldA02	8774469	8874468	1056	0.168915
BnaWGS-1007	BnaA02G0156900ZS	scaffoldA02	8784469	8884468	1010	0.168997
BnaWGS-1007	BnaA02G0156900ZS	scaffoldA02	8794469	8894468	957	0.161417
BnaWGS-1007	BnaA02G0156900ZS	scaffoldA02	8804469	8904468	995	0.144312
BnaWGS-1007	BnaA02G0156900ZS	scaffoldA02	8814469	8914468	1191	0.165145
BnaWGS-1007	BnaA02G0156900ZS	scaffoldA02	8824469	8924468	1134	0.188488
BnaWGS-1007	BnaA02G0156900ZS	scaffoldA02	8834469	8934468	986	0.235759
BnaWGS-1007	BnaA02G0156900ZS	scaffoldA02	8844469	8944468	1109	0.244042
BnaWGS-1007	BnaA02G0156900ZS	scaffoldA02	8854469	8954468	1267	0.250918
BnaWGS-1007	BnaA02G0156900ZS	scaffoldA02	8864469	8964468	1394	0.265741
BnaWGS-1007	BnaA02G0156900ZS	scaffoldA02	8874469	8974468	1330	0.276025
BnaWGS-1007	BnaA02G0156900ZS	scaffoldA02	8884469	8984468	1450	0.272668
BnaWGS-1007	BnaA02G0156900ZS	scaffoldA02	8894469	8994468	1517	0.273158
BnaWGS-1007	BnaA02G0156900ZS	scaffoldA02	8904469	9004468	1448	0.280911
BnaWGS-1007	BnaA02G0156900ZS	scaffoldA02	8914469	9014468	1311	0.288545
BnaWGS-1007	BnaA02G0156900ZS	scaffoldA02	8924469	9024468	1341	0.283287
BnaWGS-1007	BnaA02G0156900ZS	scaffoldA02	8934469	9034468	1254	0.261067
BnaWGS-1007	BnaA02G0156900ZS	scaffoldA02	8944469	9044468	1185	0.251571
BnaWGS-1007	BnaA02G0156900ZS	scaffoldA02	8954469	9054468	1164	0.246635
BnaWGS-1007	BnaA02G0156900ZS	scaffoldA02	8964469	9064468	1165	0.232856
BnaWGS-1007	BnaA02G0156900ZS	scaffoldA02	8974469	9074468	1335	0.205725
BnaWGS-1007	BnaA02G0156900ZS	scaffoldA02	8984469	9084468	1331	0.186736
BnaWGS-1007	BnaA02G0156900ZS	scaffoldA02	8994469	9094468	1323	0.182123
BnaWGS-1007	BnaA02G0156900ZS	scaffoldA02	9004469	9104468	1309	0.180547
BnaWGS-1007	BnaA02G0156900ZS	scaffoldA02	9014469	9114468	1298	0.163875
BnaWGS-1007	BnaA02G0156900ZS	scaffoldA02	9024469	9124468	1312	0.169602
BnaWGS-1007	BnaA02G0156900ZS	scaffoldA02	9034469	9134468	1390	0.23301

For Review Only

BnaWGS-1007	BnaA02G0156900ZS	scaffoldA02	9044469	9144468	1438	0.272698
BnaWGS-1007	BnaA02G0156900ZS	scaffoldA02	9054469	9154468	1364	0.29339
BnaWGS-1007	BnaA02G0156900ZS	scaffoldA02	9064469	9164468	1231	0.299484
BnaWGS-1007	BnaA02G0156900ZS	scaffoldA02	9074469	9174468	1086	0.328383
BnaWGS-1007	BnaA02G0156900ZS	scaffoldA02	9084469	9184468	958	0.357022
BnaWGS-1007	BnaA02G0156900ZS	scaffoldA02	9094469	9194468	915	0.367763
BnaWGS-1007	BnaA02G0156900ZS	scaffoldA02	9104469	9204468	1040	0.346631
BnaWGS-1007	BnaA02G0156900ZS	scaffoldA02	9114469	9214468	1079	0.340898
BnaWGS-1007	BnaA02G0156900ZS	scaffoldA02	9124469	9224468	997	0.3356
BnaWGS-1007	BnaA02G0156900ZS	scaffoldA02	9134469	9234468	871	0.292592
BnaWGS-1007	BnaA02G0156900ZS	scaffoldA02	9144469	9244468	851	0.235822
BnaWGS-1007	BnaA02G0156900ZS	scaffoldA02	9154469	9254468	923	0.211376
BnaWGS-1007	BnaA02G0156900ZS	scaffoldA02	9164469	9264468	959	0.215191
BnaWGS-1007	BnaA02G0156900ZS	scaffoldA02	9174469	9274468	937	0.23326
BnaWGS-1007	BnaA02G0156900ZS	scaffoldA02	9184469	9284468	945	0.250636
BnaWGS-1007	BnaA02G0156900ZS	scaffoldA02	9194469	9294468	959	0.270526
BnaWGS-1007	BnaA02G0156900ZS	scaffoldA02	9204469	9304468	922	0.317396
BnaWGS-1007	BnaA02G0156900ZS	scaffoldA02	9214469	9314468	822	0.345226
BnaWGS-1007	BnaA02G0156900ZS	scaffoldA02	9224469	9324468	855	0.373574
BnaWGS-1007	BnaA02G0156900ZS	scaffoldA02	9234469	9334468	933	0.385139
BnaWGS-1007	BnaA02G0156900ZS	scaffoldA02	9244469	9344468	868	0.403801
BnaWGS-1007	BnaA02G0156900ZS	scaffoldA02	9254469	9354468	814	0.411376
BnaWGS-1007	BnaA02G0156900ZS	scaffoldA02	9264469	9364468	800	0.411519
BnaWGS-1007	BnaA02G0156900ZS	scaffoldA02	9274469	9374468	879	0.413601
BnaWGS-1007	BnaA02G0156900ZS	scaffoldA02	9284469	9384468	987	0.40628
BnaWGS-1007	BnaA02G0156900ZS	scaffoldA02	9294469	9394468	979	0.400696
BnaWGS-1007	BnaA02G0156900ZS	scaffoldA02	9304469	9404468	946	0.391547
BnaWGS-1007	BnaA02G0156900ZS	scaffoldA02	9314469	9414468	1028	0.390813
BnaWGS-1007	BnaA02G0156900ZS	scaffoldA02	9324469	9424468	1135	0.383175
BnaWGS-1007	BnaA02G0156900ZS	scaffoldA02	9334469	9434468	1082	0.389076
BnaWGS-1007	BnaA02G0156900ZS	scaffoldA02	9344469	9444468	1154	0.374657
BnaWGS-1007	BnaA02G0156900ZS	scaffoldA02	9354469	9454468	1109	0.369376
BnaWGS-1007	BnaA02G0156900ZS	scaffoldA02	9364469	9464468	1105	0.368091
BnaWGS-1007	BnaA02G0156900ZS	scaffoldA02	9374469	9474468	1014	0.351965
BnaWGS-1007	BnaA02G0156900ZS	scaffoldA02	9384469	9484468	970	0.328482
BnaWGS-1007	BnaA02G0156900ZS	scaffoldA02	9394469	9494468	1008	0.308324
BnaWGS-1007	BnaA02G0156900ZS	scaffoldA02	9404469	9504468	1012	0.289735
BnaWGS-1007	BnaA02G0156900ZS	scaffoldA02	9414469	9514468	902	0.274252
BnaWGS-1007	BnaA02G0156900ZS	scaffoldA02	9424469	9524468	746	0.220961
BnaWGS-1007	BnaA02G0156900ZS	scaffoldA02	9434469	9534468	889	0.178487
BnaWGS-1007	BnaA02G0156900ZS	scaffoldA02	9444469	9544468	856	0.167408
BnaWGS-1007	BnaA02G0156900ZS	scaffoldA02	9454469	9554468	888	0.1616
BnaWGS-1007	BnaA02G0156900ZS	scaffoldA02	9464469	9564468	892	0.165324
BnaWGS-1007	BnaA02G0156900ZS	scaffoldA02	9474469	9574468	999	0.162241
BnaWGS-1007	BnaA02G0156900ZS	scaffoldA02	9484469	9584468	910	0.163687
BnaWGS-1007	BnaA02G0156900ZS	scaffoldA02	9494469	9594468	914	0.175717
BnaWGS-1007	BnaA02G0156900ZS	scaffoldA02	9504469	9604468	953	0.17877
BnaWGS-1007	BnaA02G0156900ZS	scaffoldA02	9514469	9614468	964	0.179952
BnaWGS-1007	BnaA02G0156900ZS	scaffoldA02	9524469	9624468	957	0.178177
BnaWGS-1007	BnaA02G0156900ZS	scaffoldA02	9534469	9634468	804	0.165067
BnaWGS-1007	BnaA02G0156900ZS	scaffoldA02	9544469	9644468	790	0.186464
BnaWGS-1007	BnaA02G0156900ZS	scaffoldA02	9554469	9654468	903	0.221766
BnaWGS-1007	BnaA02G0156900ZS	scaffoldA02	9564469	9664468	913	0.226118
BnaWGS-1007	BnaA02G0156900ZS	scaffoldA02	9574469	9674468	808	0.245663
BnaWGS-1007	BnaA02G0156900ZS	scaffoldA02	9584469	9684468	847	0.247243
BnaWGS-1007	BnaA02G0156900ZS	scaffoldA02	9594469	9694468	796	0.24685
BnaWGS-1007	BnaA02G0156900ZS	scaffoldA02	9604469	9704468	730	0.247956
BnaWGS-1007	BnaA02G0156900ZS	scaffoldA02	9614469	9714468	868	0.22712
BnaWGS-1007	BnaA02G0156900ZS	scaffoldA02	9624469	9724468	906	0.220675
BnaWGS-1007	BnaA02G0156900ZS	scaffoldA02	9634469	9734468	1026	0.209878

For Review Only

BnaWGS-1007	BnaA02G0156900ZS	scaffoldA02	9644469	9744468	954	0.195545
BnaWGS-1007	BnaA02G0156900ZS	scaffoldA02	9654469	9754468	759	0.135279
BnaWGS-1007	BnaA02G0156900ZS	scaffoldA02	9664469	9764468	687	0.100417
BnaWGS-1007	BnaA02G0156900ZS	scaffoldA02	9674469	9774468	760	0.089647
BnaWGS-1007	BnaA02G0156900ZS	scaffoldA02	9684469	9784468	797	0.159348
BnaWGS-1007	BnaA02G0156900ZS	scaffoldA02	9694469	9794468	863	0.193045
BnaWGS-1007	BnaA02G0156900ZS	scaffoldA02	9704469	9804468	882	0.227376
BnaWGS-1007	BnaA02G0156900ZS	scaffoldA02	9714469	9814468	875	0.2719
BnaWGS-1007	BnaA02G0156900ZS	scaffoldA02	9724469	9824468	935	0.292401
BnaWGS-1007	BnaA02G0156900ZS	scaffoldA02	9734469	9834468	842	0.309852
BnaWGS-1007	BnaA02G0156900ZS	scaffoldA02	9744469	9844468	874	0.312379
BnaWGS-1007	BnaA02G0156900ZS	scaffoldA02	9754469	9854468	978	0.301597
BnaWGS-1007	BnaA02G0156900ZS	scaffoldA02	9764469	9864468	1116	0.280523
BnaWGS-1007	BnaA02G0156900ZS	scaffoldA02	9774469	9874468	1197	0.282312
BnaWGS-1007	BnaA02G0156900ZS	scaffoldA02	9784469	9884468	1299	0.2583
BnaWGS-1007	BnaA02G0156900ZS	scaffoldA02	9794469	9894468	1316	0.262598
BnaWGS-1007	BnaA02G0156900ZS	scaffoldA02	9804469	9904468	1301	0.271091
BnaWGS-1007	BnaA02G0156900ZS	scaffoldA02	9814469	9914468	1328	0.272912
BnaWGS-1007	BnaA02G0156900ZS	scaffoldA02	9824469	9924468	1346	0.252625
BnaWGS-1007	BnaA02G0156900ZS	scaffoldA02	9834469	9934468	1303	0.248805
BnaWGS-1007	BnaA02G0156900ZS	scaffoldA02	9844469	9944468	1366	0.248744
BnaWGS-1007	BnaA02G0156900ZS	scaffoldA02	9854469	9954468	1299	0.256514
BnaWGS-1007	BnaA02G0156900ZS	scaffoldA02	9864469	9964468	1210	0.270053
BnaWGS-1007	BnaA02G0156900ZS	scaffoldA02	9874469	9974468	1185	0.287713
BnaWGS-1007	BnaA02G0156900ZS	scaffoldA02	9884469	9984468	1061	0.300119
BnaWGS-1007	BnaA02G0156900ZS	scaffoldA02	9894469	9994468	1110	0.288247
BnaWGS-1007	BnaA02G0156900ZS	scaffoldA02	9904469	10004468	1145	0.26846
BnaWGS-1007	BnaA02G0156900ZS	scaffoldA02	9914469	10014468	1181	0.244205
BnaWGS-1007	BnaA02G0156900ZS	scaffoldA02	9924469	10024468	1077	0.254103
BnaWGS-1007	BnaA02G0156900ZS	scaffoldA02	9934469	10034468	1104	0.249269
BnaWGS-1007	BnaA02G0156900ZS	scaffoldA02	9944469	10044468	1152	0.263506
BnaWGS-1007	BnaA02G0156900ZS	scaffoldA02	9954469	10054468	1225	0.248315
BnaWGS-1007	BnaA02G0156900ZS	scaffoldA02	9964469	10064468	1247	0.243493
BnaWGS-1007	BnaA02G0156900ZS	scaffoldA02	9974469	10074468	1139	0.226364
BnaWGS-1007	BnaA02G0156900ZS	scaffoldA02	9984469	10084468	1125	0.235022
BnaWGS-1007	BnaA02G0156900ZS	scaffoldA02	9994469	10094468	1046	0.232178
BnaWGS-1007	BnaA02G0156900ZS	scaffoldA02	10004469	10104468	1001	0.245464
BnaWGS-1007	BnaA02G0156900ZS	scaffoldA02	10014469	10107241	823	0.274657
BnaWGS-655	BnaA02G0156900ZS	scaffoldA02	8104469	8204468	1766	0.226419
BnaWGS-655	BnaA02G0156900ZS	scaffoldA02	8114469	8214468	1813	0.226953
BnaWGS-655	BnaA02G0156900ZS	scaffoldA02	8124469	8224468	1798	0.221323
BnaWGS-655	BnaA02G0156900ZS	scaffoldA02	8134469	8234468	1622	0.213325
BnaWGS-655	BnaA02G0156900ZS	scaffoldA02	8144469	8244468	1713	0.19841
BnaWGS-655	BnaA02G0156900ZS	scaffoldA02	8154469	8254468	1718	0.203617
BnaWGS-655	BnaA02G0156900ZS	scaffoldA02	8164469	8264468	1631	0.227865
BnaWGS-655	BnaA02G0156900ZS	scaffoldA02	8174469	8274468	1578	0.2306
BnaWGS-655	BnaA02G0156900ZS	scaffoldA02	8184469	8284468	1610	0.223179
BnaWGS-655	BnaA02G0156900ZS	scaffoldA02	8194469	8294468	1535	0.216878
BnaWGS-655	BnaA02G0156900ZS	scaffoldA02	8204469	8304468	1529	0.214692
BnaWGS-655	BnaA02G0156900ZS	scaffoldA02	8214469	8314468	1385	0.19465
BnaWGS-655	BnaA02G0156900ZS	scaffoldA02	8224469	8324468	1370	0.187744
BnaWGS-655	BnaA02G0156900ZS	scaffoldA02	8234469	8334468	1396	0.189558
BnaWGS-655	BnaA02G0156900ZS	scaffoldA02	8244469	8344468	1254	0.198904
BnaWGS-655	BnaA02G0156900ZS	scaffoldA02	8254469	8354468	1147	0.17645
BnaWGS-655	BnaA02G0156900ZS	scaffoldA02	8264469	8364468	1214	0.130216
BnaWGS-655	BnaA02G0156900ZS	scaffoldA02	8274469	8374468	1226	0.107695
BnaWGS-655	BnaA02G0156900ZS	scaffoldA02	8284469	8384468	1203	0.100435
BnaWGS-655	BnaA02G0156900ZS	scaffoldA02	8294469	8394468	1158	0.095187
BnaWGS-655	BnaA02G0156900ZS	scaffoldA02	8304469	8404468	1085	0.0895
BnaWGS-655	BnaA02G0156900ZS	scaffoldA02	8314469	8414468	1076	0.087157

For Review Only

BnaWGS-655	BnaA02G0156900ZS	scaffoldA02	8324469	8424468	1113	0.099906
BnaWGS-655	BnaA02G0156900ZS	scaffoldA02	8334469	8434468	1104	0.111389
BnaWGS-655	BnaA02G0156900ZS	scaffoldA02	8344469	8444468	1003	0.120097
BnaWGS-655	BnaA02G0156900ZS	scaffoldA02	8354469	8454468	981	0.178812
BnaWGS-655	BnaA02G0156900ZS	scaffoldA02	8364469	8464468	793	0.227921
BnaWGS-655	BnaA02G0156900ZS	scaffoldA02	8374469	8474468	680	0.261773
BnaWGS-655	BnaA02G0156900ZS	scaffoldA02	8384469	8484468	593	0.27826
BnaWGS-655	BnaA02G0156900ZS	scaffoldA02	8394469	8494468	557	0.286366
BnaWGS-655	BnaA02G0156900ZS	scaffoldA02	8404469	8504468	538	0.289841
BnaWGS-655	BnaA02G0156900ZS	scaffoldA02	8414469	8514468	436	0.313854
BnaWGS-655	BnaA02G0156900ZS	scaffoldA02	8424469	8524468	258	0.34835
BnaWGS-655	BnaA02G0156900ZS	scaffoldA02	8434469	8534468	163	0.373714
BnaWGS-655	BnaA02G0156900ZS	scaffoldA02	8444469	8544468	120	0.386555
BnaWGS-655	BnaA02G0156900ZS	scaffoldA02	8454469	8554468	72	0.285308
BnaWGS-655	BnaA02G0156900ZS	scaffoldA02	8464469	8564468	159	0.237587
BnaWGS-655	BnaA02G0156900ZS	scaffoldA02	8474469	8574468	272	0.191365
BnaWGS-655	BnaA02G0156900ZS	scaffoldA02	8484469	8584468	317	0.181753
BnaWGS-655	BnaA02G0156900ZS	scaffoldA02	8494469	8594468	355	0.172379
BnaWGS-655	BnaA02G0156900ZS	scaffoldA02	8504469	8604468	536	0.208448
BnaWGS-655	BnaA02G0156900ZS	scaffoldA02	8514469	8614468	763	0.226062
BnaWGS-655	BnaA02G0156900ZS	scaffoldA02	8524469	8624468	790	0.2309
BnaWGS-655	BnaA02G0156900ZS	scaffoldA02	8534469	8634468	910	0.220537
BnaWGS-655	BnaA02G0156900ZS	scaffoldA02	8544469	8644468	996	0.217311
BnaWGS-655	BnaA02G0156900ZS	scaffoldA02	8554469	8654468	1156	0.218257
BnaWGS-655	BnaA02G0156900ZS	scaffoldA02	8564469	8664468	1195	0.212993
BnaWGS-655	BnaA02G0156900ZS	scaffoldA02	8574469	8674468	1210	0.21735
BnaWGS-655	BnaA02G0156900ZS	scaffoldA02	8584469	8684468	1214	0.224173
BnaWGS-655	BnaA02G0156900ZS	scaffoldA02	8594469	8694468	1330	0.235422
BnaWGS-655	BnaA02G0156900ZS	scaffoldA02	8604469	8704468	1343	0.246893
BnaWGS-655	BnaA02G0156900ZS	scaffoldA02	8614469	8714468	1335	0.257384
BnaWGS-655	BnaA02G0156900ZS	scaffoldA02	8624469	8724468	1524	0.265497
BnaWGS-655	BnaA02G0156900ZS	scaffoldA02	8634469	8734468	1587	0.262722
BnaWGS-655	BnaA02G0156900ZS	scaffoldA02	8644469	8744468	1581	0.266763
BnaWGS-655	BnaA02G0156900ZS	scaffoldA02	8654469	8754468	1427	0.265746
BnaWGS-655	BnaA02G0156900ZS	scaffoldA02	8664469	8764468	1337	0.270378
BnaWGS-655	BnaA02G0156900ZS	scaffoldA02	8674469	8774468	1303	0.264434
BnaWGS-655	BnaA02G0156900ZS	scaffoldA02	8684469	8784468	1403	0.264094
BnaWGS-655	BnaA02G0156900ZS	scaffoldA02	8694469	8794468	1395	0.281906
BnaWGS-655	BnaA02G0156900ZS	scaffoldA02	8704469	8804468	1347	0.284617
BnaWGS-655	BnaA02G0156900ZS	scaffoldA02	8714469	8814468	1188	0.278745
BnaWGS-655	BnaA02G0156900ZS	scaffoldA02	8724469	8824468	1162	0.241402
BnaWGS-655	BnaA02G0156900ZS	scaffoldA02	8734469	8834468	1325	0.256288
BnaWGS-655	BnaA02G0156900ZS	scaffoldA02	8744469	8844468	1321	0.252626
BnaWGS-655	BnaA02G0156900ZS	scaffoldA02	8754469	8854468	1301	0.256278
BnaWGS-655	BnaA02G0156900ZS	scaffoldA02	8764469	8864468	1327	0.255975
BnaWGS-655	BnaA02G0156900ZS	scaffoldA02	8774469	8874468	1391	0.257823
BnaWGS-655	BnaA02G0156900ZS	scaffoldA02	8784469	8884468	1300	0.25655
BnaWGS-655	BnaA02G0156900ZS	scaffoldA02	8794469	8894468	1212	0.232604
BnaWGS-655	BnaA02G0156900ZS	scaffoldA02	8804469	8904468	1231	0.212785
BnaWGS-655	BnaA02G0156900ZS	scaffoldA02	8814469	8914468	1460	0.22276
BnaWGS-655	BnaA02G0156900ZS	scaffoldA02	8824469	8924468	1404	0.236798
BnaWGS-655	BnaA02G0156900ZS	scaffoldA02	8834469	8934468	1313	0.231447
BnaWGS-655	BnaA02G0156900ZS	scaffoldA02	8844469	8944468	1532	0.247286
BnaWGS-655	BnaA02G0156900ZS	scaffoldA02	8854469	8954468	1747	0.245193
BnaWGS-655	BnaA02G0156900ZS	scaffoldA02	8864469	8964468	1898	0.247273
BnaWGS-655	BnaA02G0156900ZS	scaffoldA02	8874469	8974468	1792	0.252467
BnaWGS-655	BnaA02G0156900ZS	scaffoldA02	8884469	8984468	2003	0.250945
BnaWGS-655	BnaA02G0156900ZS	scaffoldA02	8894469	8994468	2092	0.244482
BnaWGS-655	BnaA02G0156900ZS	scaffoldA02	8904469	9004468	2026	0.2422
BnaWGS-655	BnaA02G0156900ZS	scaffoldA02	8914469	9014468	1856	0.237144

BnaWGS-655	BnaA02G0156900ZS	scaffoldA02	8924469	9024468	1881	0.242761
BnaWGS-655	BnaA02G0156900ZS	scaffoldA02	8934469	9034468	1748	0.236432
BnaWGS-655	BnaA02G0156900ZS	scaffoldA02	8944469	9044468	1646	0.224167
BnaWGS-655	BnaA02G0156900ZS	scaffoldA02	8954469	9054468	1671	0.222394
BnaWGS-655	BnaA02G0156900ZS	scaffoldA02	8964469	9064468	1730	0.225361
BnaWGS-655	BnaA02G0156900ZS	scaffoldA02	8974469	9074468	2006	0.207203
BnaWGS-655	BnaA02G0156900ZS	scaffoldA02	8984469	9084468	1986	0.190772
BnaWGS-655	BnaA02G0156900ZS	scaffoldA02	8994469	9094468	1989	0.197739
BnaWGS-655	BnaA02G0156900ZS	scaffoldA02	9004469	9104468	1949	0.205368
BnaWGS-655	BnaA02G0156900ZS	scaffoldA02	9014469	9114468	1937	0.203955
BnaWGS-655	BnaA02G0156900ZS	scaffoldA02	9024469	9124468	1991	0.201845
BnaWGS-655	BnaA02G0156900ZS	scaffoldA02	9034469	9134468	2089	0.247184
BnaWGS-655	BnaA02G0156900ZS	scaffoldA02	9044469	9144468	2054	0.277375
BnaWGS-655	BnaA02G0156900ZS	scaffoldA02	9054469	9154468	1917	0.296569
BnaWGS-655	BnaA02G0156900ZS	scaffoldA02	9064469	9164468	1707	0.309224
BnaWGS-655	BnaA02G0156900ZS	scaffoldA02	9074469	9174468	1477	0.349341
BnaWGS-655	BnaA02G0156900ZS	scaffoldA02	9084469	9184468	1282	0.379522
BnaWGS-655	BnaA02G0156900ZS	scaffoldA02	9094469	9194468	1216	0.394454
BnaWGS-655	BnaA02G0156900ZS	scaffoldA02	9104469	9204468	1417	0.371942
BnaWGS-655	BnaA02G0156900ZS	scaffoldA02	9114469	9214468	1490	0.363856
BnaWGS-655	BnaA02G0156900ZS	scaffoldA02	9124469	9224468	1365	0.369244
BnaWGS-655	BnaA02G0156900ZS	scaffoldA02	9134469	9234468	1241	0.334256
BnaWGS-655	BnaA02G0156900ZS	scaffoldA02	9144469	9244468	1247	0.293326
BnaWGS-655	BnaA02G0156900ZS	scaffoldA02	9154469	9254468	1369	0.262843
BnaWGS-655	BnaA02G0156900ZS	scaffoldA02	9164469	9264468	1405	0.248747
BnaWGS-655	BnaA02G0156900ZS	scaffoldA02	9174469	9274468	1368	0.241692
BnaWGS-655	BnaA02G0156900ZS	scaffoldA02	9184469	9284468	1429	0.24203
BnaWGS-655	BnaA02G0156900ZS	scaffoldA02	9194469	9294468	1463	0.244513
BnaWGS-655	BnaA02G0156900ZS	scaffoldA02	9204469	9304468	1410	0.243163
BnaWGS-655	BnaA02G0156900ZS	scaffoldA02	9214469	9314468	1297	0.246848
BnaWGS-655	BnaA02G0156900ZS	scaffoldA02	9224469	9324468	1344	0.271452
BnaWGS-655	BnaA02G0156900ZS	scaffoldA02	9234469	9334468	1413	0.265899
BnaWGS-655	BnaA02G0156900ZS	scaffoldA02	9244469	9344468	1345	0.261151
BnaWGS-655	BnaA02G0156900ZS	scaffoldA02	9254469	9354468	1241	0.272021
BnaWGS-655	BnaA02G0156900ZS	scaffoldA02	9264469	9364468	1233	0.265863
BnaWGS-655	BnaA02G0156900ZS	scaffoldA02	9274469	9374468	1365	0.279199
BnaWGS-655	BnaA02G0156900ZS	scaffoldA02	9284469	9384468	1476	0.282129
BnaWGS-655	BnaA02G0156900ZS	scaffoldA02	9294469	9394468	1458	0.27669
BnaWGS-655	BnaA02G0156900ZS	scaffoldA02	9304469	9404468	1395	0.282909
BnaWGS-655	BnaA02G0156900ZS	scaffoldA02	9314469	9414468	1490	0.283453
BnaWGS-655	BnaA02G0156900ZS	scaffoldA02	9324469	9424468	1625	0.260848
BnaWGS-655	BnaA02G0156900ZS	scaffoldA02	9334469	9434468	1580	0.277715
BnaWGS-655	BnaA02G0156900ZS	scaffoldA02	9344469	9444468	1651	0.279643
BnaWGS-655	BnaA02G0156900ZS	scaffoldA02	9354469	9454468	1614	0.270049
BnaWGS-655	BnaA02G0156900ZS	scaffoldA02	9364469	9464468	1599	0.273145
BnaWGS-655	BnaA02G0156900ZS	scaffoldA02	9374469	9474468	1459	0.254899
BnaWGS-655	BnaA02G0156900ZS	scaffoldA02	9384469	9484468	1435	0.233492
BnaWGS-655	BnaA02G0156900ZS	scaffoldA02	9394469	9494468	1639	0.214044
BnaWGS-655	BnaA02G0156900ZS	scaffoldA02	9404469	9504468	1662	0.204294
BnaWGS-655	BnaA02G0156900ZS	scaffoldA02	9414469	9514468	1519	0.193594
BnaWGS-655	BnaA02G0156900ZS	scaffoldA02	9424469	9524468	1299	0.187667
BnaWGS-655	BnaA02G0156900ZS	scaffoldA02	9434469	9534468	1423	0.16943
BnaWGS-655	BnaA02G0156900ZS	scaffoldA02	9444469	9544468	1399	0.156998
BnaWGS-655	BnaA02G0156900ZS	scaffoldA02	9454469	9554468	1401	0.158387
BnaWGS-655	BnaA02G0156900ZS	scaffoldA02	9464469	9564468	1456	0.165577
BnaWGS-655	BnaA02G0156900ZS	scaffoldA02	9474469	9574468	1561	0.172963
BnaWGS-655	BnaA02G0156900ZS	scaffoldA02	9484469	9584468	1388	0.175656
BnaWGS-655	BnaA02G0156900ZS	scaffoldA02	9494469	9594468	1171	0.202692
BnaWGS-655	BnaA02G0156900ZS	scaffoldA02	9504469	9604468	1169	0.204605
BnaWGS-655	BnaA02G0156900ZS	scaffoldA02	9514469	9614468	1162	0.205911

For Review Only

BnaWGS-655	BnaA02G0156900ZS	scaffoldA02	9524469	9624468	1138	0.205892
BnaWGS-655	BnaA02G0156900ZS	scaffoldA02	9534469	9634468	943	0.199856
BnaWGS-655	BnaA02G0156900ZS	scaffoldA02	9544469	9644468	897	0.208239
BnaWGS-655	BnaA02G0156900ZS	scaffoldA02	9554469	9654468	988	0.220966
BnaWGS-655	BnaA02G0156900ZS	scaffoldA02	9564469	9664468	920	0.223398
BnaWGS-655	BnaA02G0156900ZS	scaffoldA02	9574469	9674468	801	0.22562
BnaWGS-655	BnaA02G0156900ZS	scaffoldA02	9584469	9684468	833	0.225878
BnaWGS-655	BnaA02G0156900ZS	scaffoldA02	9594469	9694468	844	0.206791
BnaWGS-655	BnaA02G0156900ZS	scaffoldA02	9604469	9704468	811	0.196565
BnaWGS-655	BnaA02G0156900ZS	scaffoldA02	9614469	9714468	981	0.186175
BnaWGS-655	BnaA02G0156900ZS	scaffoldA02	9624469	9724468	1032	0.183741
BnaWGS-655	BnaA02G0156900ZS	scaffoldA02	9634469	9734468	1238	0.174965
BnaWGS-655	BnaA02G0156900ZS	scaffoldA02	9644469	9744468	1176	0.167971
BnaWGS-655	BnaA02G0156900ZS	scaffoldA02	9654469	9754468	988	0.144821
BnaWGS-655	BnaA02G0156900ZS	scaffoldA02	9664469	9764468	943	0.133627
BnaWGS-655	BnaA02G0156900ZS	scaffoldA02	9674469	9774468	1107	0.136854
BnaWGS-655	BnaA02G0156900ZS	scaffoldA02	9684469	9784468	1219	0.165133
BnaWGS-655	BnaA02G0156900ZS	scaffoldA02	9694469	9794468	1279	0.170753
BnaWGS-655	BnaA02G0156900ZS	scaffoldA02	9704469	9804468	1364	0.19734
BnaWGS-655	BnaA02G0156900ZS	scaffoldA02	9714469	9814468	1401	0.250291
BnaWGS-655	BnaA02G0156900ZS	scaffoldA02	9724469	9824468	1485	0.265504
BnaWGS-655	BnaA02G0156900ZS	scaffoldA02	9734469	9834468	1363	0.27956
BnaWGS-655	BnaA02G0156900ZS	scaffoldA02	9744469	9844468	1414	0.275919
BnaWGS-655	BnaA02G0156900ZS	scaffoldA02	9754469	9854468	1536	0.26431
BnaWGS-655	BnaA02G0156900ZS	scaffoldA02	9764469	9864468	1751	0.248802
BnaWGS-655	BnaA02G0156900ZS	scaffoldA02	9774469	9874468	1792	0.249219
BnaWGS-655	BnaA02G0156900ZS	scaffoldA02	9784469	9884468	1849	0.228702
BnaWGS-655	BnaA02G0156900ZS	scaffoldA02	9794469	9894468	1869	0.230257
BnaWGS-655	BnaA02G0156900ZS	scaffoldA02	9804469	9904468	1795	0.23368
BnaWGS-655	BnaA02G0156900ZS	scaffoldA02	9814469	9914468	1801	0.222869
BnaWGS-655	BnaA02G0156900ZS	scaffoldA02	9824469	9924468	1866	0.212779
BnaWGS-655	BnaA02G0156900ZS	scaffoldA02	9834469	9934468	1869	0.209035
BnaWGS-655	BnaA02G0156900ZS	scaffoldA02	9844469	9944468	1948	0.209941
BnaWGS-655	BnaA02G0156900ZS	scaffoldA02	9854469	9954468	1876	0.21483
BnaWGS-655	BnaA02G0156900ZS	scaffoldA02	9864469	9964468	1720	0.227209
BnaWGS-655	BnaA02G0156900ZS	scaffoldA02	9874469	9974468	1747	0.257622
BnaWGS-655	BnaA02G0156900ZS	scaffoldA02	9884469	9984468	1673	0.266057
BnaWGS-655	BnaA02G0156900ZS	scaffoldA02	9894469	9994468	1749	0.26473
BnaWGS-655	BnaA02G0156900ZS	scaffoldA02	9904469	10004468	1745	0.251636
BnaWGS-655	BnaA02G0156900ZS	scaffoldA02	9914469	10014468	1745	0.231663
BnaWGS-655	BnaA02G0156900ZS	scaffoldA02	9924469	10024468	1561	0.236309
BnaWGS-655	BnaA02G0156900ZS	scaffoldA02	9934469	10034468	1521	0.239428
BnaWGS-655	BnaA02G0156900ZS	scaffoldA02	9944469	10044468	1597	0.261466
BnaWGS-655	BnaA02G0156900ZS	scaffoldA02	9954469	10054468	1800	0.245193
BnaWGS-655	BnaA02G0156900ZS	scaffoldA02	9964469	10064468	1875	0.236324
BnaWGS-655	BnaA02G0156900ZS	scaffoldA02	9974469	10074468	1695	0.205189
BnaWGS-655	BnaA02G0156900ZS	scaffoldA02	9984469	10084468	1648	0.210735
BnaWGS-655	BnaA02G0156900ZS	scaffoldA02	9994469	10094468	1536	0.203959
BnaWGS-655	BnaA02G0156900ZS	scaffoldA02	10004469	10104468	1483	0.205847
BnaWGS-655	BnaA02G0156900ZS	scaffoldA02	10014469	10107241	1313	0.215302
BnaWGS-655	BnaA02G0156900ZS	scaffoldA02	8104469	8204468	1766	0.161392
BnaWGS-655	BnaA02G0156900ZS	scaffoldA02	8114469	8214468	1813	0.158665
BnaWGS-655	BnaA02G0156900ZS	scaffoldA02	8124469	8224468	1798	0.150073
BnaWGS-655	BnaA02G0156900ZS	scaffoldA02	8134469	8234468	1622	0.152706
BnaWGS-655	BnaA02G0156900ZS	scaffoldA02	8144469	8244468	1713	0.16361
BnaWGS-655	BnaA02G0156900ZS	scaffoldA02	8154469	8254468	1718	0.172675
BnaWGS-655	BnaA02G0156900ZS	scaffoldA02	8164469	8264468	1631	0.181297
BnaWGS-655	BnaA02G0156900ZS	scaffoldA02	8174469	8274468	1578	0.184106
BnaWGS-655	BnaA02G0156900ZS	scaffoldA02	8184469	8284468	1610	0.182918
BnaWGS-655	BnaA02G0156900ZS	scaffoldA02	8194469	8294468	1535	0.18707

For Review Only

BnaWGS-655	BnaA02G0156900ZS	scaffoldA02	8204469	8304468	1529	0.195791
BnaWGS-655	BnaA02G0156900ZS	scaffoldA02	8214469	8314468	1385	0.196366
BnaWGS-655	BnaA02G0156900ZS	scaffoldA02	8224469	8324468	1370	0.200816
BnaWGS-655	BnaA02G0156900ZS	scaffoldA02	8234469	8334468	1396	0.189754
BnaWGS-655	BnaA02G0156900ZS	scaffoldA02	8244469	8344468	1254	0.180933
BnaWGS-655	BnaA02G0156900ZS	scaffoldA02	8254469	8354468	1147	0.168057
BnaWGS-655	BnaA02G0156900ZS	scaffoldA02	8264469	8364468	1214	0.14951
BnaWGS-655	BnaA02G0156900ZS	scaffoldA02	8274469	8374468	1226	0.140229
BnaWGS-655	BnaA02G0156900ZS	scaffoldA02	8284469	8384468	1203	0.128712
BnaWGS-655	BnaA02G0156900ZS	scaffoldA02	8294469	8394468	1158	0.122774
BnaWGS-655	BnaA02G0156900ZS	scaffoldA02	8304469	8404468	1085	0.112093
BnaWGS-655	BnaA02G0156900ZS	scaffoldA02	8314469	8414468	1076	0.112908
BnaWGS-655	BnaA02G0156900ZS	scaffoldA02	8324469	8424468	1113	0.115816
BnaWGS-655	BnaA02G0156900ZS	scaffoldA02	8334469	8434468	1104	0.131378
BnaWGS-655	BnaA02G0156900ZS	scaffoldA02	8344469	8444468	1003	0.148658
BnaWGS-655	BnaA02G0156900ZS	scaffoldA02	8354469	8454468	981	0.195647
BnaWGS-655	BnaA02G0156900ZS	scaffoldA02	8364469	8464468	793	0.235988
BnaWGS-655	BnaA02G0156900ZS	scaffoldA02	8374469	8474468	680	0.263532
BnaWGS-655	BnaA02G0156900ZS	scaffoldA02	8384469	8484468	593	0.277011
BnaWGS-655	BnaA02G0156900ZS	scaffoldA02	8394469	8494468	557	0.282737
BnaWGS-655	BnaA02G0156900ZS	scaffoldA02	8404469	8504468	538	0.285684
BnaWGS-655	BnaA02G0156900ZS	scaffoldA02	8414469	8514468	436	0.300924
BnaWGS-655	BnaA02G0156900ZS	scaffoldA02	8424469	8524468	258	0.334221
BnaWGS-655	BnaA02G0156900ZS	scaffoldA02	8434469	8534468	163	0.363145
BnaWGS-655	BnaA02G0156900ZS	scaffoldA02	8444469	8544468	120	0.360574
BnaWGS-655	BnaA02G0156900ZS	scaffoldA02	8454469	8554468	72	0.21472
BnaWGS-655	BnaA02G0156900ZS	scaffoldA02	8464469	8564468	159	0.157067
BnaWGS-655	BnaA02G0156900ZS	scaffoldA02	8474469	8574468	272	0.116133
BnaWGS-655	BnaA02G0156900ZS	scaffoldA02	8484469	8584468	317	0.11244
BnaWGS-655	BnaA02G0156900ZS	scaffoldA02	8494469	8594468	355	0.133185
BnaWGS-655	BnaA02G0156900ZS	scaffoldA02	8504469	8604468	536	0.236983
BnaWGS-655	BnaA02G0156900ZS	scaffoldA02	8514469	8614468	763	0.277018
BnaWGS-655	BnaA02G0156900ZS	scaffoldA02	8524469	8624468	790	0.272036
BnaWGS-655	BnaA02G0156900ZS	scaffoldA02	8534469	8634468	910	0.251232
BnaWGS-655	BnaA02G0156900ZS	scaffoldA02	8544469	8644468	996	0.238049
BnaWGS-655	BnaA02G0156900ZS	scaffoldA02	8554469	8654468	1156	0.221598
BnaWGS-655	BnaA02G0156900ZS	scaffoldA02	8564469	8664468	1195	0.218041
BnaWGS-655	BnaA02G0156900ZS	scaffoldA02	8574469	8674468	1210	0.223456
BnaWGS-655	BnaA02G0156900ZS	scaffoldA02	8584469	8684468	1214	0.234214
BnaWGS-655	BnaA02G0156900ZS	scaffoldA02	8594469	8694468	1330	0.239252
BnaWGS-655	BnaA02G0156900ZS	scaffoldA02	8604469	8704468	1343	0.225005
BnaWGS-655	BnaA02G0156900ZS	scaffoldA02	8614469	8714468	1335	0.201579
BnaWGS-655	BnaA02G0156900ZS	scaffoldA02	8624469	8724468	1524	0.204545
BnaWGS-655	BnaA02G0156900ZS	scaffoldA02	8634469	8734468	1587	0.209491
BnaWGS-655	BnaA02G0156900ZS	scaffoldA02	8644469	8744468	1581	0.21798
BnaWGS-655	BnaA02G0156900ZS	scaffoldA02	8654469	8754468	1427	0.234562
BnaWGS-655	BnaA02G0156900ZS	scaffoldA02	8664469	8764468	1337	0.246083
BnaWGS-655	BnaA02G0156900ZS	scaffoldA02	8674469	8774468	1303	0.255139
BnaWGS-655	BnaA02G0156900ZS	scaffoldA02	8684469	8784468	1403	0.235232
BnaWGS-655	BnaA02G0156900ZS	scaffoldA02	8694469	8794468	1395	0.221754
BnaWGS-655	BnaA02G0156900ZS	scaffoldA02	8704469	8804468	1347	0.23083
BnaWGS-655	BnaA02G0156900ZS	scaffoldA02	8714469	8814468	1188	0.232722
BnaWGS-655	BnaA02G0156900ZS	scaffoldA02	8724469	8824468	1162	0.224732
BnaWGS-655	BnaA02G0156900ZS	scaffoldA02	8734469	8834468	1325	0.202067
BnaWGS-655	BnaA02G0156900ZS	scaffoldA02	8744469	8844468	1321	0.196744
BnaWGS-655	BnaA02G0156900ZS	scaffoldA02	8754469	8854468	1301	0.1873
BnaWGS-655	BnaA02G0156900ZS	scaffoldA02	8764469	8864468	1327	0.180223
BnaWGS-655	BnaA02G0156900ZS	scaffoldA02	8774469	8874468	1391	0.163003
BnaWGS-655	BnaA02G0156900ZS	scaffoldA02	8784469	8884468	1300	0.165158
BnaWGS-655	BnaA02G0156900ZS	scaffoldA02	8794469	8894468	1212	0.165533

For Review Only

BnaWGS-655	BnaA02G0156900ZS	scaffoldA02	8804469	8904468	1231	0.139777
BnaWGS-655	BnaA02G0156900ZS	scaffoldA02	8814469	8914468	1460	0.143799
BnaWGS-655	BnaA02G0156900ZS	scaffoldA02	8824469	8924468	1404	0.141724
BnaWGS-655	BnaA02G0156900ZS	scaffoldA02	8834469	8934468	1313	0.147452
BnaWGS-655	BnaA02G0156900ZS	scaffoldA02	8844469	8944468	1532	0.146189
BnaWGS-655	BnaA02G0156900ZS	scaffoldA02	8854469	8954468	1747	0.153007
BnaWGS-655	BnaA02G0156900ZS	scaffoldA02	8864469	8964468	1898	0.162527
BnaWGS-655	BnaA02G0156900ZS	scaffoldA02	8874469	8974468	1792	0.16788
BnaWGS-655	BnaA02G0156900ZS	scaffoldA02	8884469	8984468	2003	0.175686
BnaWGS-655	BnaA02G0156900ZS	scaffoldA02	8894469	8994468	2092	0.179143
BnaWGS-655	BnaA02G0156900ZS	scaffoldA02	8904469	9004468	2026	0.178541
BnaWGS-655	BnaA02G0156900ZS	scaffoldA02	8914469	9014468	1856	0.178814
BnaWGS-655	BnaA02G0156900ZS	scaffoldA02	8924469	9024468	1881	0.179437
BnaWGS-655	BnaA02G0156900ZS	scaffoldA02	8934469	9034468	1748	0.183787
BnaWGS-655	BnaA02G0156900ZS	scaffoldA02	8944469	9044468	1646	0.183751
BnaWGS-655	BnaA02G0156900ZS	scaffoldA02	8954469	9054468	1671	0.165692
BnaWGS-655	BnaA02G0156900ZS	scaffoldA02	8964469	9064468	1730	0.1431
BnaWGS-655	BnaA02G0156900ZS	scaffoldA02	8974469	9074468	2006	0.136378
BnaWGS-655	BnaA02G0156900ZS	scaffoldA02	8984469	9084468	1986	0.119074
BnaWGS-655	BnaA02G0156900ZS	scaffoldA02	8994469	9094468	1989	0.108171
BnaWGS-655	BnaA02G0156900ZS	scaffoldA02	9004469	9104468	1949	0.105542
BnaWGS-655	BnaA02G0156900ZS	scaffoldA02	9014469	9114468	1937	0.096659
BnaWGS-655	BnaA02G0156900ZS	scaffoldA02	9024469	9124468	1991	0.084678
BnaWGS-655	BnaA02G0156900ZS	scaffoldA02	9034469	9134468	2089	0.07491
BnaWGS-655	BnaA02G0156900ZS	scaffoldA02	9044469	9144468	2054	0.067498
BnaWGS-655	BnaA02G0156900ZS	scaffoldA02	9054469	9154468	1917	0.06579
BnaWGS-655	BnaA02G0156900ZS	scaffoldA02	9064469	9164468	1707	0.06668
BnaWGS-655	BnaA02G0156900ZS	scaffoldA02	9074469	9174468	1477	0.054419
BnaWGS-655	BnaA02G0156900ZS	scaffoldA02	9084469	9184468	1282	0.047883
BnaWGS-655	BnaA02G0156900ZS	scaffoldA02	9094469	9194468	1216	0.043214
BnaWGS-655	BnaA02G0156900ZS	scaffoldA02	9104469	9204468	1417	0.053228
BnaWGS-655	BnaA02G0156900ZS	scaffoldA02	9114469	9214468	1490	0.057849
BnaWGS-655	BnaA02G0156900ZS	scaffoldA02	9124469	9224468	1365	0.061162
BnaWGS-655	BnaA02G0156900ZS	scaffoldA02	9134469	9234468	1241	0.073061
BnaWGS-655	BnaA02G0156900ZS	scaffoldA02	9144469	9244468	1247	0.083781
BnaWGS-655	BnaA02G0156900ZS	scaffoldA02	9154469	9254468	1369	0.110951
BnaWGS-655	BnaA02G0156900ZS	scaffoldA02	9164469	9264468	1405	0.119082
BnaWGS-655	BnaA02G0156900ZS	scaffoldA02	9174469	9274468	1368	0.129461
BnaWGS-655	BnaA02G0156900ZS	scaffoldA02	9184469	9284468	1429	0.149047
BnaWGS-655	BnaA02G0156900ZS	scaffoldA02	9194469	9294468	1463	0.170958
BnaWGS-655	BnaA02G0156900ZS	scaffoldA02	9204469	9304468	1410	0.192499
BnaWGS-655	BnaA02G0156900ZS	scaffoldA02	9214469	9314468	1297	0.205269
BnaWGS-655	BnaA02G0156900ZS	scaffoldA02	9224469	9324468	1344	0.229905
BnaWGS-655	BnaA02G0156900ZS	scaffoldA02	9234469	9334468	1413	0.227543
BnaWGS-655	BnaA02G0156900ZS	scaffoldA02	9244469	9344468	1345	0.235899
BnaWGS-655	BnaA02G0156900ZS	scaffoldA02	9254469	9354468	1241	0.247172
BnaWGS-655	BnaA02G0156900ZS	scaffoldA02	9264469	9364468	1233	0.241582
BnaWGS-655	BnaA02G0156900ZS	scaffoldA02	9274469	9374468	1365	0.251279
BnaWGS-655	BnaA02G0156900ZS	scaffoldA02	9284469	9384468	1476	0.251478
BnaWGS-655	BnaA02G0156900ZS	scaffoldA02	9294469	9394468	1458	0.245114
BnaWGS-655	BnaA02G0156900ZS	scaffoldA02	9304469	9404468	1395	0.251734
BnaWGS-655	BnaA02G0156900ZS	scaffoldA02	9314469	9414468	1490	0.251497
BnaWGS-655	BnaA02G0156900ZS	scaffoldA02	9324469	9424468	1625	0.225857
BnaWGS-655	BnaA02G0156900ZS	scaffoldA02	9334469	9434468	1580	0.238329
BnaWGS-655	BnaA02G0156900ZS	scaffoldA02	9344469	9444468	1651	0.233355
BnaWGS-655	BnaA02G0156900ZS	scaffoldA02	9354469	9454468	1614	0.22371
BnaWGS-655	BnaA02G0156900ZS	scaffoldA02	9364469	9464468	1599	0.22376
BnaWGS-655	BnaA02G0156900ZS	scaffoldA02	9374469	9474468	1459	0.207315
BnaWGS-655	BnaA02G0156900ZS	scaffoldA02	9384469	9484468	1435	0.180228
BnaWGS-655	BnaA02G0156900ZS	scaffoldA02	9394469	9494468	1639	0.157299

For Review Only

BnaWGS-655	BnaA02G0156900ZS	scaffoldA02	9404469	9504468	1662	0.137853
BnaWGS-655	BnaA02G0156900ZS	scaffoldA02	9414469	9514468	1519	0.124107
BnaWGS-655	BnaA02G0156900ZS	scaffoldA02	9424469	9524468	1299	0.115769
BnaWGS-655	BnaA02G0156900ZS	scaffoldA02	9434469	9534468	1423	0.102324
BnaWGS-655	BnaA02G0156900ZS	scaffoldA02	9444469	9544468	1399	0.114157
BnaWGS-655	BnaA02G0156900ZS	scaffoldA02	9454469	9554468	1401	0.13044
BnaWGS-655	BnaA02G0156900ZS	scaffoldA02	9464469	9564468	1456	0.144076
BnaWGS-655	BnaA02G0156900ZS	scaffoldA02	9474469	9574468	1561	0.150939
BnaWGS-655	BnaA02G0156900ZS	scaffoldA02	9484469	9584468	1388	0.158563
BnaWGS-655	BnaA02G0156900ZS	scaffoldA02	9494469	9594468	1171	0.177012
BnaWGS-655	BnaA02G0156900ZS	scaffoldA02	9504469	9604468	1169	0.188497
BnaWGS-655	BnaA02G0156900ZS	scaffoldA02	9514469	9614468	1162	0.188671
BnaWGS-655	BnaA02G0156900ZS	scaffoldA02	9524469	9624468	1138	0.194131
BnaWGS-655	BnaA02G0156900ZS	scaffoldA02	9534469	9634468	943	0.206237
BnaWGS-655	BnaA02G0156900ZS	scaffoldA02	9544469	9644468	897	0.188125
BnaWGS-655	BnaA02G0156900ZS	scaffoldA02	9554469	9654468	988	0.162859
BnaWGS-655	BnaA02G0156900ZS	scaffoldA02	9564469	9664468	920	0.151846
BnaWGS-655	BnaA02G0156900ZS	scaffoldA02	9574469	9674468	801	0.140396
BnaWGS-655	BnaA02G0156900ZS	scaffoldA02	9584469	9684468	833	0.144133
BnaWGS-655	BnaA02G0156900ZS	scaffoldA02	9594469	9694468	844	0.147046
BnaWGS-655	BnaA02G0156900ZS	scaffoldA02	9604469	9704468	811	0.142834
BnaWGS-655	BnaA02G0156900ZS	scaffoldA02	9614469	9714468	981	0.158826
BnaWGS-655	BnaA02G0156900ZS	scaffoldA02	9624469	9724468	1032	0.161104
BnaWGS-655	BnaA02G0156900ZS	scaffoldA02	9634469	9734468	1238	0.181685
BnaWGS-655	BnaA02G0156900ZS	scaffoldA02	9644469	9744468	1176	0.189277
BnaWGS-655	BnaA02G0156900ZS	scaffoldA02	9654469	9754468	988	0.213589
BnaWGS-655	BnaA02G0156900ZS	scaffoldA02	9664469	9764468	943	0.215736
BnaWGS-655	BnaA02G0156900ZS	scaffoldA02	9674469	9774468	1107	0.218171
BnaWGS-655	BnaA02G0156900ZS	scaffoldA02	9684469	9784468	1219	0.198829
BnaWGS-655	BnaA02G0156900ZS	scaffoldA02	9694469	9794468	1279	0.188538
BnaWGS-655	BnaA02G0156900ZS	scaffoldA02	9704469	9804468	1364	0.190822
BnaWGS-655	BnaA02G0156900ZS	scaffoldA02	9714469	9814468	1401	0.197147
BnaWGS-655	BnaA02G0156900ZS	scaffoldA02	9724469	9824468	1485	0.206086
BnaWGS-655	BnaA02G0156900ZS	scaffoldA02	9734469	9834468	1363	0.191443
BnaWGS-655	BnaA02G0156900ZS	scaffoldA02	9744469	9844468	1414	0.190149
BnaWGS-655	BnaA02G0156900ZS	scaffoldA02	9754469	9854468	1536	0.189207
BnaWGS-655	BnaA02G0156900ZS	scaffoldA02	9764469	9864468	1751	0.198738
BnaWGS-655	BnaA02G0156900ZS	scaffoldA02	9774469	9874468	1792	0.198135
BnaWGS-655	BnaA02G0156900ZS	scaffoldA02	9784469	9884468	1849	0.199332
BnaWGS-655	BnaA02G0156900ZS	scaffoldA02	9794469	9894468	1869	0.205416
BnaWGS-655	BnaA02G0156900ZS	scaffoldA02	9804469	9904468	1795	0.216031
BnaWGS-655	BnaA02G0156900ZS	scaffoldA02	9814469	9914468	1801	0.226039
BnaWGS-655	BnaA02G0156900ZS	scaffoldA02	9824469	9924468	1866	0.213853
BnaWGS-655	BnaA02G0156900ZS	scaffoldA02	9834469	9934468	1869	0.212396
BnaWGS-655	BnaA02G0156900ZS	scaffoldA02	9844469	9944468	1948	0.214553
BnaWGS-655	BnaA02G0156900ZS	scaffoldA02	9854469	9954468	1876	0.215231
BnaWGS-655	BnaA02G0156900ZS	scaffoldA02	9864469	9964468	1720	0.208349
BnaWGS-655	BnaA02G0156900ZS	scaffoldA02	9874469	9974468	1747	0.218348
BnaWGS-655	BnaA02G0156900ZS	scaffoldA02	9884469	9984468	1673	0.226595
BnaWGS-655	BnaA02G0156900ZS	scaffoldA02	9894469	9994468	1749	0.227915
BnaWGS-655	BnaA02G0156900ZS	scaffoldA02	9904469	10004468	1745	0.222489
BnaWGS-655	BnaA02G0156900ZS	scaffoldA02	9914469	10014468	1745	0.193162
BnaWGS-655	BnaA02G0156900ZS	scaffoldA02	9924469	10024468	1561	0.196217
BnaWGS-655	BnaA02G0156900ZS	scaffoldA02	9934469	10034468	1521	0.197435
BnaWGS-655	BnaA02G0156900ZS	scaffoldA02	9944469	10044468	1597	0.187974
BnaWGS-655	BnaA02G0156900ZS	scaffoldA02	9954469	10054468	1800	0.193963
BnaWGS-655	BnaA02G0156900ZS	scaffoldA02	9964469	10064468	1875	0.199338
BnaWGS-655	BnaA02G0156900ZS	scaffoldA02	9974469	10074468	1695	0.190655
BnaWGS-655	BnaA02G0156900ZS	scaffoldA02	9984469	10084468	1648	0.190907
BnaWGS-655	BnaA02G0156900ZS	scaffoldA02	9994469	10094468	1536	0.191738

For Review Only

BnaWGS-655	BnaA02G0156900ZS	scaffoldA02	10004469	10104468	1483	0.185335
BnaWGS-655	BnaA02G0156900ZS	scaffoldA02	10014469	10107241	1313	0.205747
BnaWGS-655	BnaA02G0156900ZS	scaffoldA02	8104469	8204468	1766	0.14548
BnaWGS-655	BnaA02G0156900ZS	scaffoldA02	8114469	8214468	1813	0.133753
BnaWGS-655	BnaA02G0156900ZS	scaffoldA02	8124469	8224468	1798	0.116849
BnaWGS-655	BnaA02G0156900ZS	scaffoldA02	8134469	8234468	1622	0.115115
BnaWGS-655	BnaA02G0156900ZS	scaffoldA02	8144469	8244468	1713	0.121161
BnaWGS-655	BnaA02G0156900ZS	scaffoldA02	8154469	8254468	1718	0.127601
BnaWGS-655	BnaA02G0156900ZS	scaffoldA02	8164469	8264468	1631	0.156628
BnaWGS-655	BnaA02G0156900ZS	scaffoldA02	8174469	8274468	1578	0.170318
BnaWGS-655	BnaA02G0156900ZS	scaffoldA02	8184469	8284468	1610	0.159191
BnaWGS-655	BnaA02G0156900ZS	scaffoldA02	8194469	8294468	1535	0.163721
BnaWGS-655	BnaA02G0156900ZS	scaffoldA02	8204469	8304468	1529	0.16577
BnaWGS-655	BnaA02G0156900ZS	scaffoldA02	8214469	8314468	1385	0.178561
BnaWGS-655	BnaA02G0156900ZS	scaffoldA02	8224469	8324468	1370	0.184352
BnaWGS-655	BnaA02G0156900ZS	scaffoldA02	8234469	8334468	1396	0.181721
BnaWGS-655	BnaA02G0156900ZS	scaffoldA02	8244469	8344468	1254	0.170269
BnaWGS-655	BnaA02G0156900ZS	scaffoldA02	8254469	8354468	1147	0.132825
BnaWGS-655	BnaA02G0156900ZS	scaffoldA02	8264469	8364468	1214	0.081622
BnaWGS-655	BnaA02G0156900ZS	scaffoldA02	8274469	8374468	1226	0.063953
BnaWGS-655	BnaA02G0156900ZS	scaffoldA02	8284469	8384468	1203	0.062337
BnaWGS-655	BnaA02G0156900ZS	scaffoldA02	8294469	8394468	1158	0.062467
BnaWGS-655	BnaA02G0156900ZS	scaffoldA02	8304469	8404468	1085	0.060594
BnaWGS-655	BnaA02G0156900ZS	scaffoldA02	8314469	8414468	1076	0.064017
BnaWGS-655	BnaA02G0156900ZS	scaffoldA02	8324469	8424468	1113	0.059168
BnaWGS-655	BnaA02G0156900ZS	scaffoldA02	8334469	8434468	1104	0.060808
BnaWGS-655	BnaA02G0156900ZS	scaffoldA02	8344469	8444468	1003	0.062466
BnaWGS-655	BnaA02G0156900ZS	scaffoldA02	8354469	8454468	981	0.059499
BnaWGS-655	BnaA02G0156900ZS	scaffoldA02	8364469	8464468	793	0.062143
BnaWGS-655	BnaA02G0156900ZS	scaffoldA02	8374469	8474468	680	0.062866
BnaWGS-655	BnaA02G0156900ZS	scaffoldA02	8384469	8484468	593	0.066892
BnaWGS-655	BnaA02G0156900ZS	scaffoldA02	8394469	8494468	557	0.067233
BnaWGS-655	BnaA02G0156900ZS	scaffoldA02	8404469	8504468	538	0.067611
BnaWGS-655	BnaA02G0156900ZS	scaffoldA02	8414469	8514468	436	0.058626
BnaWGS-655	BnaA02G0156900ZS	scaffoldA02	8424469	8524468	258	0.042843
BnaWGS-655	BnaA02G0156900ZS	scaffoldA02	8434469	8534468	163	0.027232
BnaWGS-655	BnaA02G0156900ZS	scaffoldA02	8444469	8544468	120	0.014794
BnaWGS-655	BnaA02G0156900ZS	scaffoldA02	8454469	8554468	72	0.125114
BnaWGS-655	BnaA02G0156900ZS	scaffoldA02	8464469	8564468	159	0.177462
BnaWGS-655	BnaA02G0156900ZS	scaffoldA02	8474469	8574468	272	0.195473
BnaWGS-655	BnaA02G0156900ZS	scaffoldA02	8484469	8584468	317	0.192621
BnaWGS-655	BnaA02G0156900ZS	scaffoldA02	8494469	8594468	355	0.19291
BnaWGS-655	BnaA02G0156900ZS	scaffoldA02	8504469	8604468	536	0.114817
BnaWGS-655	BnaA02G0156900ZS	scaffoldA02	8514469	8614468	763	0.084315
BnaWGS-655	BnaA02G0156900ZS	scaffoldA02	8524469	8624468	790	0.097656
BnaWGS-655	BnaA02G0156900ZS	scaffoldA02	8534469	8634468	910	0.098872
BnaWGS-655	BnaA02G0156900ZS	scaffoldA02	8544469	8644468	996	0.102689
BnaWGS-655	BnaA02G0156900ZS	scaffoldA02	8554469	8654468	1156	0.112336
BnaWGS-655	BnaA02G0156900ZS	scaffoldA02	8564469	8664468	1195	0.108437
BnaWGS-655	BnaA02G0156900ZS	scaffoldA02	8574469	8674468	1210	0.102822
BnaWGS-655	BnaA02G0156900ZS	scaffoldA02	8584469	8684468	1214	0.098096
BnaWGS-655	BnaA02G0156900ZS	scaffoldA02	8594469	8694468	1330	0.096465
BnaWGS-655	BnaA02G0156900ZS	scaffoldA02	8604469	8704468	1343	0.103706
BnaWGS-655	BnaA02G0156900ZS	scaffoldA02	8614469	8714468	1335	0.119298
BnaWGS-655	BnaA02G0156900ZS	scaffoldA02	8624469	8724468	1524	0.157028
BnaWGS-655	BnaA02G0156900ZS	scaffoldA02	8634469	8734468	1587	0.175314
BnaWGS-655	BnaA02G0156900ZS	scaffoldA02	8644469	8744468	1581	0.178867
BnaWGS-655	BnaA02G0156900ZS	scaffoldA02	8654469	8754468	1427	0.190964
BnaWGS-655	BnaA02G0156900ZS	scaffoldA02	8664469	8764468	1337	0.196106
BnaWGS-655	BnaA02G0156900ZS	scaffoldA02	8674469	8774468	1303	0.196922

For Review Only

BnaWGS-655	BnaA02G0156900ZS	scaffoldA02	8684469	8784468	1403	0.213513
BnaWGS-655	BnaA02G0156900ZS	scaffoldA02	8694469	8794468	1395	0.248468
BnaWGS-655	BnaA02G0156900ZS	scaffoldA02	8704469	8804468	1347	0.258762
BnaWGS-655	BnaA02G0156900ZS	scaffoldA02	8714469	8814468	1188	0.277832
BnaWGS-655	BnaA02G0156900ZS	scaffoldA02	8724469	8824468	1162	0.253161
BnaWGS-655	BnaA02G0156900ZS	scaffoldA02	8734469	8834468	1325	0.244879
BnaWGS-655	BnaA02G0156900ZS	scaffoldA02	8744469	8844468	1321	0.244447
BnaWGS-655	BnaA02G0156900ZS	scaffoldA02	8754469	8854468	1301	0.235693
BnaWGS-655	BnaA02G0156900ZS	scaffoldA02	8764469	8864468	1327	0.239809
BnaWGS-655	BnaA02G0156900ZS	scaffoldA02	8774469	8874468	1391	0.244224
BnaWGS-655	BnaA02G0156900ZS	scaffoldA02	8784469	8884468	1300	0.242721
BnaWGS-655	BnaA02G0156900ZS	scaffoldA02	8794469	8894468	1212	0.229335
BnaWGS-655	BnaA02G0156900ZS	scaffoldA02	8804469	8904468	1231	0.257905
BnaWGS-655	BnaA02G0156900ZS	scaffoldA02	8814469	8914468	1460	0.312386
BnaWGS-655	BnaA02G0156900ZS	scaffoldA02	8824469	8924468	1404	0.325126
BnaWGS-655	BnaA02G0156900ZS	scaffoldA02	8834469	8934468	1313	0.364102
BnaWGS-655	BnaA02G0156900ZS	scaffoldA02	8844469	8944468	1532	0.34098
BnaWGS-655	BnaA02G0156900ZS	scaffoldA02	8854469	8954468	1747	0.340913
BnaWGS-655	BnaA02G0156900ZS	scaffoldA02	8864469	8964468	1898	0.337357
BnaWGS-655	BnaA02G0156900ZS	scaffoldA02	8874469	8974468	1792	0.347018
BnaWGS-655	BnaA02G0156900ZS	scaffoldA02	8884469	8984468	2003	0.342131
BnaWGS-655	BnaA02G0156900ZS	scaffoldA02	8894469	8994468	2092	0.349402
BnaWGS-655	BnaA02G0156900ZS	scaffoldA02	8904469	9004468	2026	0.348569
BnaWGS-655	BnaA02G0156900ZS	scaffoldA02	8914469	9014468	1856	0.312261
BnaWGS-655	BnaA02G0156900ZS	scaffoldA02	8924469	9024468	1881	0.32398
BnaWGS-655	BnaA02G0156900ZS	scaffoldA02	8934469	9034468	1748	0.32608
BnaWGS-655	BnaA02G0156900ZS	scaffoldA02	8944469	9044468	1646	0.358961
BnaWGS-655	BnaA02G0156900ZS	scaffoldA02	8954469	9054468	1671	0.365916
BnaWGS-655	BnaA02G0156900ZS	scaffoldA02	8964469	9064468	1730	0.369319
BnaWGS-655	BnaA02G0156900ZS	scaffoldA02	8974469	9074468	2006	0.340089
BnaWGS-655	BnaA02G0156900ZS	scaffoldA02	8984469	9084468	1986	0.315815
BnaWGS-655	BnaA02G0156900ZS	scaffoldA02	8994469	9094468	1989	0.320226
BnaWGS-655	BnaA02G0156900ZS	scaffoldA02	9004469	9104468	1949	0.332786
BnaWGS-655	BnaA02G0156900ZS	scaffoldA02	9014469	9114468	1937	0.342291
BnaWGS-655	BnaA02G0156900ZS	scaffoldA02	9024469	9124468	1991	0.339979
BnaWGS-655	BnaA02G0156900ZS	scaffoldA02	9034469	9134468	2089	0.358513
BnaWGS-655	BnaA02G0156900ZS	scaffoldA02	9044469	9144468	2054	0.367004
BnaWGS-655	BnaA02G0156900ZS	scaffoldA02	9054469	9154468	1917	0.370281
BnaWGS-655	BnaA02G0156900ZS	scaffoldA02	9064469	9164468	1707	0.381511
BnaWGS-655	BnaA02G0156900ZS	scaffoldA02	9074469	9174468	1477	0.421719
BnaWGS-655	BnaA02G0156900ZS	scaffoldA02	9084469	9184468	1282	0.469532
BnaWGS-655	BnaA02G0156900ZS	scaffoldA02	9094469	9194468	1216	0.467346
BnaWGS-655	BnaA02G0156900ZS	scaffoldA02	9104469	9204468	1417	0.468564
BnaWGS-655	BnaA02G0156900ZS	scaffoldA02	9114469	9214468	1490	0.470232
BnaWGS-655	BnaA02G0156900ZS	scaffoldA02	9124469	9224468	1365	0.469065
BnaWGS-655	BnaA02G0156900ZS	scaffoldA02	9134469	9234468	1241	0.448791
BnaWGS-655	BnaA02G0156900ZS	scaffoldA02	9144469	9244468	1247	0.435027
BnaWGS-655	BnaA02G0156900ZS	scaffoldA02	9154469	9254468	1369	0.40861
BnaWGS-655	BnaA02G0156900ZS	scaffoldA02	9164469	9264468	1405	0.382469
BnaWGS-655	BnaA02G0156900ZS	scaffoldA02	9174469	9274468	1368	0.361459
BnaWGS-655	BnaA02G0156900ZS	scaffoldA02	9184469	9284468	1429	0.335743
BnaWGS-655	BnaA02G0156900ZS	scaffoldA02	9194469	9294468	1463	0.305113
BnaWGS-655	BnaA02G0156900ZS	scaffoldA02	9204469	9304468	1410	0.211497
BnaWGS-655	BnaA02G0156900ZS	scaffoldA02	9214469	9314468	1297	0.154213
BnaWGS-655	BnaA02G0156900ZS	scaffoldA02	9224469	9324468	1344	0.146024
BnaWGS-655	BnaA02G0156900ZS	scaffoldA02	9234469	9334468	1413	0.137813
BnaWGS-655	BnaA02G0156900ZS	scaffoldA02	9244469	9344468	1345	0.087501
BnaWGS-655	BnaA02G0156900ZS	scaffoldA02	9254469	9354468	1241	0.03516
BnaWGS-655	BnaA02G0156900ZS	scaffoldA02	9264469	9364468	1233	0.038425
BnaWGS-655	BnaA02G0156900ZS	scaffoldA02	9274469	9374468	1365	0.040419

For Review Only

BnaWGS-655	BnaA02G0156900ZS	scaffoldA02	9284469	9384468	1476	0.042486
BnaWGS-655	BnaA02G0156900ZS	scaffoldA02	9294469	9394468	1458	0.041446
BnaWGS-655	BnaA02G0156900ZS	scaffoldA02	9304469	9404468	1395	0.043889
BnaWGS-655	BnaA02G0156900ZS	scaffoldA02	9314469	9414468	1490	0.042082
BnaWGS-655	BnaA02G0156900ZS	scaffoldA02	9324469	9424468	1625	0.038032
BnaWGS-655	BnaA02G0156900ZS	scaffoldA02	9334469	9434468	1580	0.03699
BnaWGS-655	BnaA02G0156900ZS	scaffoldA02	9344469	9444468	1651	0.069375
BnaWGS-655	BnaA02G0156900ZS	scaffoldA02	9354469	9454468	1614	0.072314
BnaWGS-655	BnaA02G0156900ZS	scaffoldA02	9364469	9464468	1599	0.066777
BnaWGS-655	BnaA02G0156900ZS	scaffoldA02	9374469	9474468	1459	0.068779
BnaWGS-655	BnaA02G0156900ZS	scaffoldA02	9384469	9484468	1435	0.067749
BnaWGS-655	BnaA02G0156900ZS	scaffoldA02	9394469	9494468	1639	0.068018
BnaWGS-655	BnaA02G0156900ZS	scaffoldA02	9404469	9504468	1662	0.082361
BnaWGS-655	BnaA02G0156900ZS	scaffoldA02	9414469	9514468	1519	0.088319
BnaWGS-655	BnaA02G0156900ZS	scaffoldA02	9424469	9524468	1299	0.139522
BnaWGS-655	BnaA02G0156900ZS	scaffoldA02	9434469	9534468	1423	0.241351
BnaWGS-655	BnaA02G0156900ZS	scaffoldA02	9444469	9544468	1399	0.258223
BnaWGS-655	BnaA02G0156900ZS	scaffoldA02	9454469	9554468	1401	0.303064
BnaWGS-655	BnaA02G0156900ZS	scaffoldA02	9464469	9564468	1456	0.307256
BnaWGS-655	BnaA02G0156900ZS	scaffoldA02	9474469	9574468	1561	0.324903
BnaWGS-655	BnaA02G0156900ZS	scaffoldA02	9484469	9584468	1388	0.341702
BnaWGS-655	BnaA02G0156900ZS	scaffoldA02	9494469	9594468	1171	0.36781
BnaWGS-655	BnaA02G0156900ZS	scaffoldA02	9504469	9604468	1169	0.372954
BnaWGS-655	BnaA02G0156900ZS	scaffoldA02	9514469	9614468	1162	0.373778
BnaWGS-655	BnaA02G0156900ZS	scaffoldA02	9524469	9624468	1138	0.369698
BnaWGS-655	BnaA02G0156900ZS	scaffoldA02	9534469	9634468	943	0.346766
BnaWGS-655	BnaA02G0156900ZS	scaffoldA02	9544469	9644468	897	0.323591
BnaWGS-655	BnaA02G0156900ZS	scaffoldA02	9554469	9654468	988	0.262725
BnaWGS-655	BnaA02G0156900ZS	scaffoldA02	9564469	9664468	920	0.258886
BnaWGS-655	BnaA02G0156900ZS	scaffoldA02	9574469	9674468	801	0.249166
BnaWGS-655	BnaA02G0156900ZS	scaffoldA02	9584469	9684468	833	0.272114
BnaWGS-655	BnaA02G0156900ZS	scaffoldA02	9594469	9694468	844	0.281785
BnaWGS-655	BnaA02G0156900ZS	scaffoldA02	9604469	9704468	811	0.294156
BnaWGS-655	BnaA02G0156900ZS	scaffoldA02	9614469	9714468	981	0.356428
BnaWGS-655	BnaA02G0156900ZS	scaffoldA02	9624469	9724468	1032	0.376766
BnaWGS-655	BnaA02G0156900ZS	scaffoldA02	9634469	9734468	1238	0.401278
BnaWGS-655	BnaA02G0156900ZS	scaffoldA02	9644469	9744468	1176	0.421887
BnaWGS-655	BnaA02G0156900ZS	scaffoldA02	9654469	9754468	988	0.482145
BnaWGS-655	BnaA02G0156900ZS	scaffoldA02	9664469	9764468	943	0.495963
BnaWGS-655	BnaA02G0156900ZS	scaffoldA02	9674469	9774468	1107	0.474575
BnaWGS-655	BnaA02G0156900ZS	scaffoldA02	9684469	9784468	1219	0.461482
BnaWGS-655	BnaA02G0156900ZS	scaffoldA02	9694469	9794468	1279	0.446124
BnaWGS-655	BnaA02G0156900ZS	scaffoldA02	9704469	9804468	1364	0.445136
BnaWGS-655	BnaA02G0156900ZS	scaffoldA02	9714469	9814468	1401	0.426946
BnaWGS-655	BnaA02G0156900ZS	scaffoldA02	9724469	9824468	1485	0.405577
BnaWGS-655	BnaA02G0156900ZS	scaffoldA02	9734469	9834468	1363	0.390241
BnaWGS-655	BnaA02G0156900ZS	scaffoldA02	9744469	9844468	1414	0.381232
BnaWGS-655	BnaA02G0156900ZS	scaffoldA02	9754469	9854468	1536	0.38471
BnaWGS-655	BnaA02G0156900ZS	scaffoldA02	9764469	9864468	1751	0.37113
BnaWGS-655	BnaA02G0156900ZS	scaffoldA02	9774469	9874468	1792	0.345491
BnaWGS-655	BnaA02G0156900ZS	scaffoldA02	9784469	9884468	1849	0.309625
BnaWGS-655	BnaA02G0156900ZS	scaffoldA02	9794469	9894468	1869	0.296427
BnaWGS-655	BnaA02G0156900ZS	scaffoldA02	9804469	9904468	1795	0.268661
BnaWGS-655	BnaA02G0156900ZS	scaffoldA02	9814469	9914468	1801	0.243316
BnaWGS-655	BnaA02G0156900ZS	scaffoldA02	9824469	9924468	1866	0.256388
BnaWGS-655	BnaA02G0156900ZS	scaffoldA02	9834469	9934468	1869	0.264229
BnaWGS-655	BnaA02G0156900ZS	scaffoldA02	9844469	9944468	1948	0.283607
BnaWGS-655	BnaA02G0156900ZS	scaffoldA02	9854469	9954468	1876	0.276314
BnaWGS-655	BnaA02G0156900ZS	scaffoldA02	9864469	9964468	1720	0.280326
BnaWGS-655	BnaA02G0156900ZS	scaffoldA02	9874469	9974468	1747	0.284216

For Review Only

BnaWGS-655	BnaA02G0156900ZS	scaffoldA02	9884469	9984468	1673	0.309515
BnaWGS-655	BnaA02G0156900ZS	scaffoldA02	9894469	9994468	1749	0.331112
BnaWGS-655	BnaA02G0156900ZS	scaffoldA02	9904469	10004468	1745	0.353394
BnaWGS-655	BnaA02G0156900ZS	scaffoldA02	9914469	10014468	1745	0.344447
BnaWGS-655	BnaA02G0156900ZS	scaffoldA02	9924469	10024468	1561	0.339007
BnaWGS-655	BnaA02G0156900ZS	scaffoldA02	9934469	10034468	1521	0.337966
BnaWGS-655	BnaA02G0156900ZS	scaffoldA02	9944469	10044468	1597	0.334833
BnaWGS-655	BnaA02G0156900ZS	scaffoldA02	9954469	10054468	1800	0.352662
BnaWGS-655	BnaA02G0156900ZS	scaffoldA02	9964469	10064468	1875	0.355864
BnaWGS-655	BnaA02G0156900ZS	scaffoldA02	9974469	10074468	1695	0.377352
BnaWGS-655	BnaA02G0156900ZS	scaffoldA02	9984469	10084468	1648	0.379184
BnaWGS-655	BnaA02G0156900ZS	scaffoldA02	9994469	10094468	1536	0.389872
BnaWGS-655	BnaA02G0156900ZS	scaffoldA02	10004469	10104468	1483	0.383887
BnaWGS-655	BnaA02G0156900ZS	scaffoldA02	10014469	10107241	1313	0.401062
BnaWGS-1007	BnaA10G0244800ZS	scaffoldA10	22943882	23043881	511	0.199589
BnaWGS-1007	BnaA10G0244800ZS	scaffoldA10	22953882	23053881	488	0.194111
BnaWGS-1007	BnaA10G0244800ZS	scaffoldA10	22963882	23063881	489	0.182187
BnaWGS-1007	BnaA10G0244800ZS	scaffoldA10	22973882	23073881	512	0.161132
BnaWGS-1007	BnaA10G0244800ZS	scaffoldA10	22983882	23083881	485	0.171042
BnaWGS-1007	BnaA10G0244800ZS	scaffoldA10	22993882	23093881	513	0.165857
BnaWGS-1007	BnaA10G0244800ZS	scaffoldA10	23003882	23103881	536	0.226984
BnaWGS-1007	BnaA10G0244800ZS	scaffoldA10	23013882	23113881	569	0.258108
BnaWGS-1007	BnaA10G0244800ZS	scaffoldA10	23023882	23123881	568	0.264416
BnaWGS-1007	BnaA10G0244800ZS	scaffoldA10	23033882	23133881	655	0.297217
BnaWGS-1007	BnaA10G0244800ZS	scaffoldA10	23043882	23143881	746	0.305108
BnaWGS-1007	BnaA10G0244800ZS	scaffoldA10	23053882	23153881	833	0.300581
BnaWGS-1007	BnaA10G0244800ZS	scaffoldA10	23063882	23163881	942	0.292715
BnaWGS-1007	BnaA10G0244800ZS	scaffoldA10	23073882	23173881	981	0.285421
BnaWGS-1007	BnaA10G0244800ZS	scaffoldA10	23083882	23183881	1068	0.292506
BnaWGS-1007	BnaA10G0244800ZS	scaffoldA10	23093882	23193881	1086	0.294133
BnaWGS-1007	BnaA10G0244800ZS	scaffoldA10	23103882	23203881	1103	0.280228
BnaWGS-1007	BnaA10G0244800ZS	scaffoldA10	23113882	23213881	1212	0.279664
BnaWGS-1007	BnaA10G0244800ZS	scaffoldA10	23123882	23223881	1293	0.285454
BnaWGS-1007	BnaA10G0244800ZS	scaffoldA10	23133882	23233881	1177	0.281507
BnaWGS-1007	BnaA10G0244800ZS	scaffoldA10	23143882	23243881	1046	0.280326
BnaWGS-1007	BnaA10G0244800ZS	scaffoldA10	23153882	23253881	953	0.281537
BnaWGS-1007	BnaA10G0244800ZS	scaffoldA10	23163882	23263881	900	0.274742
BnaWGS-1007	BnaA10G0244800ZS	scaffoldA10	23173882	23273881	837	0.282117
BnaWGS-1007	BnaA10G0244800ZS	scaffoldA10	23183882	23283881	773	0.260688
BnaWGS-1007	BnaA10G0244800ZS	scaffoldA10	23193882	23293881	739	0.228582
BnaWGS-1007	BnaA10G0244800ZS	scaffoldA10	23203882	23303881	713	0.212025
BnaWGS-1007	BnaA10G0244800ZS	scaffoldA10	23213882	23313881	594	0.149195
BnaWGS-1007	BnaA10G0244800ZS	scaffoldA10	23223882	23323881	618	0.065421
BnaWGS-1007	BnaA10G0244800ZS	scaffoldA10	23233882	23333881	680	0.088154
BnaWGS-1007	BnaA10G0244800ZS	scaffoldA10	23243882	23343881	825	0.12978
BnaWGS-1007	BnaA10G0244800ZS	scaffoldA10	23253882	23353881	878	0.147188
BnaWGS-1007	BnaA10G0244800ZS	scaffoldA10	23263882	23363881	800	0.154818
BnaWGS-1007	BnaA10G0244800ZS	scaffoldA10	23273882	23373881	785	0.163745
BnaWGS-1007	BnaA10G0244800ZS	scaffoldA10	23283882	23383881	747	0.170505
BnaWGS-1007	BnaA10G0244800ZS	scaffoldA10	23293882	23393881	670	0.189234
BnaWGS-1007	BnaA10G0244800ZS	scaffoldA10	23303882	23403881	665	0.207256
BnaWGS-1007	BnaA10G0244800ZS	scaffoldA10	23313882	23413881	668	0.215073
BnaWGS-1007	BnaA10G0244800ZS	scaffoldA10	23323882	23423881	605	0.232762
BnaWGS-1007	BnaA10G0244800ZS	scaffoldA10	23333882	23433881	587	0.229637
BnaWGS-1007	BnaA10G0244800ZS	scaffoldA10	23343882	23443881	494	0.224184
BnaWGS-1007	BnaA10G0244800ZS	scaffoldA10	23353882	23453881	461	0.21195
BnaWGS-1007	BnaA10G0244800ZS	scaffoldA10	23363882	23463881	497	0.217498
BnaWGS-1007	BnaA10G0244800ZS	scaffoldA10	23373882	23473881	521	0.220302
BnaWGS-1007	BnaA10G0244800ZS	scaffoldA10	23383882	23483881	603	0.234664
BnaWGS-1007	BnaA10G0244800ZS	scaffoldA10	23393882	23493881	685	0.251043

For Review Only

BnaWGS-1007	BnaA10G0244800ZS	scaffoldA10	23403882	23503881	733	0.299944
BnaWGS-1007	BnaA10G0244800ZS	scaffoldA10	23413882	23513881	872	0.334703
BnaWGS-1007	BnaA10G0244800ZS	scaffoldA10	23423882	23523881	894	0.343199
BnaWGS-1007	BnaA10G0244800ZS	scaffoldA10	23433882	23533881	872	0.351321
BnaWGS-1007	BnaA10G0244800ZS	scaffoldA10	23443882	23543881	1015	0.343899
BnaWGS-1007	BnaA10G0244800ZS	scaffoldA10	23453882	23553881	1068	0.343884
BnaWGS-1007	BnaA10G0244800ZS	scaffoldA10	23463882	23563881	1090	0.343607
BnaWGS-1007	BnaA10G0244800ZS	scaffoldA10	23473882	23573881	1074	0.346707
BnaWGS-1007	BnaA10G0244800ZS	scaffoldA10	23483882	23583881	1002	0.346207
BnaWGS-1007	BnaA10G0244800ZS	scaffoldA10	23493882	23593881	978	0.336752
BnaWGS-1007	BnaA10G0244800ZS	scaffoldA10	23503882	23603881	999	0.317841
BnaWGS-1007	BnaA10G0244800ZS	scaffoldA10	23513882	23613881	831	0.290631
BnaWGS-1007	BnaA10G0244800ZS	scaffoldA10	23523882	23623881	851	0.264889
BnaWGS-1007	BnaA10G0244800ZS	scaffoldA10	23533882	23633881	934	0.269418
BnaWGS-1007	BnaA10G0244800ZS	scaffoldA10	23543882	23643881	830	0.26105
BnaWGS-1007	BnaA10G0244800ZS	scaffoldA10	23553882	23653881	856	0.254501
BnaWGS-1007	BnaA10G0244800ZS	scaffoldA10	23563882	23663881	890	0.240913
BnaWGS-1007	BnaA10G0244800ZS	scaffoldA10	23573882	23673881	1076	0.235589
BnaWGS-1007	BnaA10G0244800ZS	scaffoldA10	23583882	23683881	1220	0.240666
BnaWGS-1007	BnaA10G0244800ZS	scaffoldA10	23593882	23693881	1194	0.238701
BnaWGS-1007	BnaA10G0244800ZS	scaffoldA10	23603882	23703881	1129	0.220109
BnaWGS-1007	BnaA10G0244800ZS	scaffoldA10	23613882	23713881	1189	0.236124
BnaWGS-1007	BnaA10G0244800ZS	scaffoldA10	23623882	23723881	1142	0.260758
BnaWGS-1007	BnaA10G0244800ZS	scaffoldA10	23633882	23733881	1113	0.252644
BnaWGS-1007	BnaA10G0244800ZS	scaffoldA10	23643882	23743881	1172	0.242528
BnaWGS-1007	BnaA10G0244800ZS	scaffoldA10	23653882	23753881	1237	0.260847
BnaWGS-1007	BnaA10G0244800ZS	scaffoldA10	23663882	23763881	1262	0.280588
BnaWGS-1007	BnaA10G0244800ZS	scaffoldA10	23673882	23773881	1134	0.318732
BnaWGS-1007	BnaA10G0244800ZS	scaffoldA10	23683882	23783881	1076	0.322488
BnaWGS-1007	BnaA10G0244800ZS	scaffoldA10	23693882	23793881	1115	0.347715
BnaWGS-1007	BnaA10G0244800ZS	scaffoldA10	23703882	23803881	1167	0.379199
BnaWGS-1007	BnaA10G0244800ZS	scaffoldA10	23713882	23813881	1176	0.385173
BnaWGS-1007	BnaA10G0244800ZS	scaffoldA10	23723882	23823881	1178	0.392487
BnaWGS-1007	BnaA10G0244800ZS	scaffoldA10	23733882	23833881	1122	0.410635
BnaWGS-1007	BnaA10G0244800ZS	scaffoldA10	23743882	23843881	998	0.436557
BnaWGS-1007	BnaA10G0244800ZS	scaffoldA10	23753882	23853881	913	0.453342
BnaWGS-1007	BnaA10G0244800ZS	scaffoldA10	23763882	23863881	916	0.462433
BnaWGS-1007	BnaA10G0244800ZS	scaffoldA10	23773882	23873881	848	0.448606
BnaWGS-1007	BnaA10G0244800ZS	scaffoldA10	23783882	23883881	793	0.449254
BnaWGS-1007	BnaA10G0244800ZS	scaffoldA10	23793882	23893881	744	0.434714
BnaWGS-1007	BnaA10G0244800ZS	scaffoldA10	23803882	23903881	684	0.420823
BnaWGS-1007	BnaA10G0244800ZS	scaffoldA10	23813882	23913881	623	0.414712
BnaWGS-1007	BnaA10G0244800ZS	scaffoldA10	23823882	23923881	567	0.395083
BnaWGS-1007	BnaA10G0244800ZS	scaffoldA10	23833882	23933881	546	0.367978
BnaWGS-1007	BnaA10G0244800ZS	scaffoldA10	23843882	23943881	580	0.335434
BnaWGS-1007	BnaA10G0244800ZS	scaffoldA10	23853882	23953881	513	0.311973
BnaWGS-1007	BnaA10G0244800ZS	scaffoldA10	23863882	23963881	409	0.232727
BnaWGS-1007	BnaA10G0244800ZS	scaffoldA10	23873882	23973881	432	0.245311
BnaWGS-1007	BnaA10G0244800ZS	scaffoldA10	23883882	23983881	588	0.171631
BnaWGS-1007	BnaA10G0244800ZS	scaffoldA10	23893882	23993881	585	0.175193
BnaWGS-1007	BnaA10G0244800ZS	scaffoldA10	23903882	24003881	620	0.163206
BnaWGS-1007	BnaA10G0244800ZS	scaffoldA10	23913882	24013881	659	0.214446
BnaWGS-1007	BnaA10G0244800ZS	scaffoldA10	23923882	24023881	750	0.248971
BnaWGS-1007	BnaA10G0244800ZS	scaffoldA10	23933882	24033881	747	0.259801
BnaWGS-1007	BnaA10G0244800ZS	scaffoldA10	23943882	24043881	823	0.277849
BnaWGS-1007	BnaA10G0244800ZS	scaffoldA10	23953882	24053881	831	0.29206
BnaWGS-1007	BnaA10G0244800ZS	scaffoldA10	23963882	24063881	919	0.299293
BnaWGS-1007	BnaA10G0244800ZS	scaffoldA10	23973882	24073881	1039	0.320029
BnaWGS-1007	BnaA10G0244800ZS	scaffoldA10	23983882	24083881	1020	0.344236
BnaWGS-1007	BnaA10G0244800ZS	scaffoldA10	23993882	24093881	1197	0.323939

For Review Only

BnaWGS-1007	BnaA10G0244800ZS	scaffoldA10	24003882	24103881	1342	0.314666
BnaWGS-1007	BnaA10G0244800ZS	scaffoldA10	24013882	24113881	1400	0.304215
BnaWGS-1007	BnaA10G0244800ZS	scaffoldA10	24023882	24123881	1506	0.276312
BnaWGS-1007	BnaA10G0244800ZS	scaffoldA10	24033882	24133881	1553	0.268599
BnaWGS-1007	BnaA10G0244800ZS	scaffoldA10	24043882	24143881	1523	0.260992
BnaWGS-1007	BnaA10G0244800ZS	scaffoldA10	24053882	24153881	1592	0.255855
BnaWGS-1007	BnaA10G0244800ZS	scaffoldA10	24063882	24163881	1607	0.25743
BnaWGS-1007	BnaA10G0244800ZS	scaffoldA10	24073882	24173881	1645	0.258863
BnaWGS-1007	BnaA10G0244800ZS	scaffoldA10	24083882	24183881	1712	0.27021
BnaWGS-1007	BnaA10G0244800ZS	scaffoldA10	24093882	24193881	1794	0.269952
BnaWGS-1007	BnaA10G0244800ZS	scaffoldA10	24103882	24203881	1636	0.267763
BnaWGS-1007	BnaA10G0244800ZS	scaffoldA10	24113882	24213881	1549	0.260331
BnaWGS-1007	BnaA10G0244800ZS	scaffoldA10	24123882	24223881	1535	0.270338
BnaWGS-1007	BnaA10G0244800ZS	scaffoldA10	24133882	24233881	1641	0.269776
BnaWGS-1007	BnaA10G0244800ZS	scaffoldA10	24143882	24243881	1663	0.264492
BnaWGS-1007	BnaA10G0244800ZS	scaffoldA10	24153882	24253881	1761	0.253223
BnaWGS-1007	BnaA10G0244800ZS	scaffoldA10	24163882	24263881	1778	0.246297
BnaWGS-1007	BnaA10G0244800ZS	scaffoldA10	24173882	24273881	1804	0.256558
BnaWGS-1007	BnaA10G0244800ZS	scaffoldA10	24183882	24283881	1655	0.245275
BnaWGS-1007	BnaA10G0244800ZS	scaffoldA10	24193882	24293881	1439	0.250889
BnaWGS-1007	BnaA10G0244800ZS	scaffoldA10	24203882	24303881	1554	0.244819
BnaWGS-1007	BnaA10G0244800ZS	scaffoldA10	24213882	24313881	1699	0.250695
BnaWGS-1007	BnaA10G0244800ZS	scaffoldA10	24223882	24323881	1713	0.251711
BnaWGS-1007	BnaA10G0244800ZS	scaffoldA10	24233882	24333881	1662	0.251277
BnaWGS-1007	BnaA10G0244800ZS	scaffoldA10	24243882	24343881	1729	0.253135
BnaWGS-1007	BnaA10G0244800ZS	scaffoldA10	24253882	24353881	1748	0.254036
BnaWGS-1007	BnaA10G0244800ZS	scaffoldA10	24263882	24363881	1674	0.254963
BnaWGS-1007	BnaA10G0244800ZS	scaffoldA10	24273882	24373881	1603	0.225803
BnaWGS-1007	BnaA10G0244800ZS	scaffoldA10	24283882	24383881	1647	0.231572
BnaWGS-1007	BnaA10G0244800ZS	scaffoldA10	24293882	24393881	1623	0.237354
BnaWGS-1007	BnaA10G0244800ZS	scaffoldA10	24303882	24403881	1448	0.248645
BnaWGS-1007	BnaA10G0244800ZS	scaffoldA10	24313882	24413881	1427	0.240275
BnaWGS-1007	BnaA10G0244800ZS	scaffoldA10	24323882	24423881	1434	0.227994
BnaWGS-1007	BnaA10G0244800ZS	scaffoldA10	24333882	24433881	1449	0.222601
BnaWGS-1007	BnaA10G0244800ZS	scaffoldA10	24343882	24443881	1324	0.224997
BnaWGS-1007	BnaA10G0244800ZS	scaffoldA10	24353882	24453881	1165	0.230592
BnaWGS-1007	BnaA10G0244800ZS	scaffoldA10	24363882	24463881	1306	0.230244
BnaWGS-1007	BnaA10G0244800ZS	scaffoldA10	24373882	24473881	1314	0.203556
BnaWGS-1007	BnaA10G0244800ZS	scaffoldA10	24383882	24483881	1244	0.196898
BnaWGS-1007	BnaA10G0244800ZS	scaffoldA10	24393882	24493881	1326	0.181974
BnaWGS-1007	BnaA10G0244800ZS	scaffoldA10	24403882	24503881	1382	0.175055
BnaWGS-1007	BnaA10G0244800ZS	scaffoldA10	24413882	24513881	1286	0.172186
BnaWGS-1007	BnaA10G0244800ZS	scaffoldA10	24423882	24523881	1126	0.177866
BnaWGS-1007	BnaA10G0244800ZS	scaffoldA10	24433882	24533881	1033	0.175589
BnaWGS-1007	BnaA10G0244800ZS	scaffoldA10	24443882	24543881	1049	0.17117
BnaWGS-1007	BnaA10G0244800ZS	scaffoldA10	24453882	24553881	1114	0.166188
BnaWGS-1007	BnaA10G0244800ZS	scaffoldA10	24463882	24563881	1052	0.138497
BnaWGS-1007	BnaA10G0244800ZS	scaffoldA10	24473882	24573881	1023	0.131518
BnaWGS-1007	BnaA10G0244800ZS	scaffoldA10	24483882	24583881	1056	0.125583
BnaWGS-1007	BnaA10G0244800ZS	scaffoldA10	24493882	24593881	980	0.113167
BnaWGS-1007	BnaA10G0244800ZS	scaffoldA10	24503882	24603881	1008	0.101102
BnaWGS-1007	BnaA10G0244800ZS	scaffoldA10	24513882	24613881	1025	0.109218
BnaWGS-1007	BnaA10G0244800ZS	scaffoldA10	24523882	24623881	1086	0.128223
BnaWGS-1007	BnaA10G0244800ZS	scaffoldA10	24533882	24633881	1145	0.142059
BnaWGS-1007	BnaA10G0244800ZS	scaffoldA10	24543882	24643881	1182	0.162214
BnaWGS-1007	BnaA10G0244800ZS	scaffoldA10	24553882	24653881	1172	0.184729
BnaWGS-1007	BnaA10G0244800ZS	scaffoldA10	24563882	24663881	1138	0.199767
BnaWGS-1007	BnaA10G0244800ZS	scaffoldA10	24573882	24673881	1024	0.227545
BnaWGS-1007	BnaA10G0244800ZS	scaffoldA10	24583882	24683881	1010	0.241713
BnaWGS-1007	BnaA10G0244800ZS	scaffoldA10	24593882	24693881	1040	0.253733

BnaWGS-1007	BnaA10G0244800ZS	scaffoldA10	24603882	24703881	1011	0.258854
BnaWGS-1007	BnaA10G0244800ZS	scaffoldA10	24613882	24713881	980	0.259525
BnaWGS-1007	BnaA10G0244800ZS	scaffoldA10	24623882	24723881	910	0.258069
BnaWGS-1007	BnaA10G0244800ZS	scaffoldA10	24633882	24733881	915	0.255197
BnaWGS-1007	BnaA10G0244800ZS	scaffoldA10	24643882	24743881	913	0.256101
BnaWGS-1007	BnaA10G0244800ZS	scaffoldA10	24653882	24753881	932	0.243393
BnaWGS-1007	BnaA10G0244800ZS	scaffoldA10	24663882	24763881	838	0.248636
BnaWGS-1007	BnaA10G0244800ZS	scaffoldA10	24673882	24773881	802	0.239946
BnaWGS-1007	BnaA10G0244800ZS	scaffoldA10	24683882	24783881	849	0.220045
BnaWGS-1007	BnaA10G0244800ZS	scaffoldA10	24693882	24793881	845	0.20388
BnaWGS-1007	BnaA10G0244800ZS	scaffoldA10	24703882	24803881	783	0.21504
BnaWGS-1007	BnaA10G0244800ZS	scaffoldA10	24713882	24813881	778	0.200181
BnaWGS-1007	BnaA10G0244800ZS	scaffoldA10	24723882	24823881	926	0.181624
BnaWGS-1007	BnaA10G0244800ZS	scaffoldA10	24733882	24833881	935	0.174063
BnaWGS-1007	BnaA10G0244800ZS	scaffoldA10	24743882	24843881	961	0.147642
BnaWGS-1007	BnaA10G0244800ZS	scaffoldA10	24753882	24853881	910	0.141188
BnaWGS-1007	BnaA10G0244800ZS	scaffoldA10	24763882	24863881	923	0.143713
BnaWGS-1007	BnaA10G0244800ZS	scaffoldA10	24773882	24873881	1033	0.148681
BnaWGS-1007	BnaA10G0244800ZS	scaffoldA10	24783882	24883881	932	0.151401
BnaWGS-1007	BnaA10G0244800ZS	scaffoldA10	24793882	24893881	899	0.153527
BnaWGS-1007	BnaA10G0244800ZS	scaffoldA10	24803882	24903881	1034	0.156733
BnaWGS-1007	BnaA10G0244800ZS	scaffoldA10	24813882	24913881	1090	0.159078
BnaWGS-1007	BnaA10G0244800ZS	scaffoldA10	24823882	24923881	1222	0.210509
BnaWGS-1007	BnaA10G0244800ZS	scaffoldA10	24833882	24933881	1312	0.220693
BnaWGS-1007	BnaA10G0244800ZS	scaffoldA10	24843882	24943881	1248	0.239183
BnaWGS-1007	BnaA10G0244800ZS	scaffoldA10	24853882	24947904	1187	0.241996
BnaWGS-1007	BnaA10G0244800ZS	scaffoldA10	22943882	23043881	511	0.206499
BnaWGS-1007	BnaA10G0244800ZS	scaffoldA10	22953882	23053881	488	0.202001
BnaWGS-1007	BnaA10G0244800ZS	scaffoldA10	22963882	23063881	489	0.189752
BnaWGS-1007	BnaA10G0244800ZS	scaffoldA10	22973882	23073881	512	0.170026
BnaWGS-1007	BnaA10G0244800ZS	scaffoldA10	22983882	23083881	485	0.179693
BnaWGS-1007	BnaA10G0244800ZS	scaffoldA10	22993882	23093881	513	0.076118
BnaWGS-1007	BnaA10G0244800ZS	scaffoldA10	23003882	23103881	536	0.047624
BnaWGS-1007	BnaA10G0244800ZS	scaffoldA10	23013882	23113881	569	0.0401
BnaWGS-1007	BnaA10G0244800ZS	scaffoldA10	23023882	23123881	568	0.039002
BnaWGS-1007	BnaA10G0244800ZS	scaffoldA10	23033882	23133881	655	0.074565
BnaWGS-1007	BnaA10G0244800ZS	scaffoldA10	23043882	23143881	746	0.100739
BnaWGS-1007	BnaA10G0244800ZS	scaffoldA10	23053882	23153881	833	0.1187
BnaWGS-1007	BnaA10G0244800ZS	scaffoldA10	23063882	23163881	942	0.138416
BnaWGS-1007	BnaA10G0244800ZS	scaffoldA10	23073882	23173881	981	0.150103
BnaWGS-1007	BnaA10G0244800ZS	scaffoldA10	23083882	23183881	1068	0.173092
BnaWGS-1007	BnaA10G0244800ZS	scaffoldA10	23093882	23193881	1086	0.213501
BnaWGS-1007	BnaA10G0244800ZS	scaffoldA10	23103882	23203881	1103	0.228767
BnaWGS-1007	BnaA10G0244800ZS	scaffoldA10	23113882	23213881	1212	0.224565
BnaWGS-1007	BnaA10G0244800ZS	scaffoldA10	23123882	23223881	1293	0.217237
BnaWGS-1007	BnaA10G0244800ZS	scaffoldA10	23133882	23233881	1177	0.226293
BnaWGS-1007	BnaA10G0244800ZS	scaffoldA10	23143882	23243881	1046	0.232973
BnaWGS-1007	BnaA10G0244800ZS	scaffoldA10	23153882	23253881	953	0.235854
BnaWGS-1007	BnaA10G0244800ZS	scaffoldA10	23163882	23263881	900	0.22421
BnaWGS-1007	BnaA10G0244800ZS	scaffoldA10	23173882	23273881	837	0.219846
BnaWGS-1007	BnaA10G0244800ZS	scaffoldA10	23183882	23283881	773	0.190899
BnaWGS-1007	BnaA10G0244800ZS	scaffoldA10	23193882	23293881	739	0.121099
BnaWGS-1007	BnaA10G0244800ZS	scaffoldA10	23203882	23303881	713	0.077602
BnaWGS-1007	BnaA10G0244800ZS	scaffoldA10	23213882	23313881	594	0.049003
BnaWGS-1007	BnaA10G0244800ZS	scaffoldA10	23223882	23323881	618	0.047085
BnaWGS-1007	BnaA10G0244800ZS	scaffoldA10	23233882	23333881	680	0.084109
BnaWGS-1007	BnaA10G0244800ZS	scaffoldA10	23243882	23343881	825	0.133745
BnaWGS-1007	BnaA10G0244800ZS	scaffoldA10	23253882	23353881	878	0.150565
BnaWGS-1007	BnaA10G0244800ZS	scaffoldA10	23263882	23363881	800	0.16675
BnaWGS-1007	BnaA10G0244800ZS	scaffoldA10	23273882	23373881	785	0.188357

For Review Only

BnaWGS-1007	BnaA10G0244800ZS	scaffoldA10	23283882	23383881	747	0.195555
BnaWGS-1007	BnaA10G0244800ZS	scaffoldA10	23293882	23393881	670	0.216368
BnaWGS-1007	BnaA10G0244800ZS	scaffoldA10	23303882	23403881	665	0.235649
BnaWGS-1007	BnaA10G0244800ZS	scaffoldA10	23313882	23413881	668	0.239838
BnaWGS-1007	BnaA10G0244800ZS	scaffoldA10	23323882	23423881	605	0.249435
BnaWGS-1007	BnaA10G0244800ZS	scaffoldA10	23333882	23433881	587	0.236804
BnaWGS-1007	BnaA10G0244800ZS	scaffoldA10	23343882	23443881	494	0.224385
BnaWGS-1007	BnaA10G0244800ZS	scaffoldA10	23353882	23453881	461	0.216456
BnaWGS-1007	BnaA10G0244800ZS	scaffoldA10	23363882	23463881	497	0.201214
BnaWGS-1007	BnaA10G0244800ZS	scaffoldA10	23373882	23473881	521	0.168458
BnaWGS-1007	BnaA10G0244800ZS	scaffoldA10	23383882	23483881	603	0.158474
BnaWGS-1007	BnaA10G0244800ZS	scaffoldA10	23393882	23493881	685	0.13436
BnaWGS-1007	BnaA10G0244800ZS	scaffoldA10	23403882	23503881	733	0.098407
BnaWGS-1007	BnaA10G0244800ZS	scaffoldA10	23413882	23513881	872	0.098477
BnaWGS-1007	BnaA10G0244800ZS	scaffoldA10	23423882	23523881	894	0.103216
BnaWGS-1007	BnaA10G0244800ZS	scaffoldA10	23433882	23533881	872	0.110889
BnaWGS-1007	BnaA10G0244800ZS	scaffoldA10	23443882	23543881	1015	0.149026
BnaWGS-1007	BnaA10G0244800ZS	scaffoldA10	23453882	23553881	1068	0.149309
BnaWGS-1007	BnaA10G0244800ZS	scaffoldA10	23463882	23563881	1090	0.151108
BnaWGS-1007	BnaA10G0244800ZS	scaffoldA10	23473882	23573881	1074	0.153247
BnaWGS-1007	BnaA10G0244800ZS	scaffoldA10	23483882	23583881	1002	0.15927
BnaWGS-1007	BnaA10G0244800ZS	scaffoldA10	23493882	23593881	978	0.166912
BnaWGS-1007	BnaA10G0244800ZS	scaffoldA10	23503882	23603881	999	0.16487
BnaWGS-1007	BnaA10G0244800ZS	scaffoldA10	23513882	23613881	831	0.171586
BnaWGS-1007	BnaA10G0244800ZS	scaffoldA10	23523882	23623881	851	0.181477
BnaWGS-1007	BnaA10G0244800ZS	scaffoldA10	23533882	23633881	934	0.177939
BnaWGS-1007	BnaA10G0244800ZS	scaffoldA10	23543882	23643881	830	0.150357
BnaWGS-1007	BnaA10G0244800ZS	scaffoldA10	23553882	23653881	856	0.155299
BnaWGS-1007	BnaA10G0244800ZS	scaffoldA10	23563882	23663881	890	0.149234
BnaWGS-1007	BnaA10G0244800ZS	scaffoldA10	23573882	23673881	1076	0.171283
BnaWGS-1007	BnaA10G0244800ZS	scaffoldA10	23583882	23683881	1220	0.181326
BnaWGS-1007	BnaA10G0244800ZS	scaffoldA10	23593882	23693881	1194	0.182055
BnaWGS-1007	BnaA10G0244800ZS	scaffoldA10	23603882	23703881	1129	0.192512
BnaWGS-1007	BnaA10G0244800ZS	scaffoldA10	23613882	23713881	1189	0.194921
BnaWGS-1007	BnaA10G0244800ZS	scaffoldA10	23623882	23723881	1142	0.207552
BnaWGS-1007	BnaA10G0244800ZS	scaffoldA10	23633882	23733881	1113	0.233547
BnaWGS-1007	BnaA10G0244800ZS	scaffoldA10	23643882	23743881	1172	0.247839
BnaWGS-1007	BnaA10G0244800ZS	scaffoldA10	23653882	23753881	1237	0.259277
BnaWGS-1007	BnaA10G0244800ZS	scaffoldA10	23663882	23763881	1262	0.285448
BnaWGS-1007	BnaA10G0244800ZS	scaffoldA10	23673882	23773881	1134	0.296701
BnaWGS-1007	BnaA10G0244800ZS	scaffoldA10	23683882	23783881	1076	0.31102
BnaWGS-1007	BnaA10G0244800ZS	scaffoldA10	23693882	23793881	1115	0.325323
BnaWGS-1007	BnaA10G0244800ZS	scaffoldA10	23703882	23803881	1167	0.327315
BnaWGS-1007	BnaA10G0244800ZS	scaffoldA10	23713882	23813881	1176	0.342576
BnaWGS-1007	BnaA10G0244800ZS	scaffoldA10	23723882	23823881	1178	0.348035
BnaWGS-1007	BnaA10G0244800ZS	scaffoldA10	23733882	23833881	1122	0.333992
BnaWGS-1007	BnaA10G0244800ZS	scaffoldA10	23743882	23843881	998	0.336142
BnaWGS-1007	BnaA10G0244800ZS	scaffoldA10	23753882	23853881	913	0.349965
BnaWGS-1007	BnaA10G0244800ZS	scaffoldA10	23763882	23863881	916	0.365222
BnaWGS-1007	BnaA10G0244800ZS	scaffoldA10	23773882	23873881	848	0.368508
BnaWGS-1007	BnaA10G0244800ZS	scaffoldA10	23783882	23883881	793	0.372409
BnaWGS-1007	BnaA10G0244800ZS	scaffoldA10	23793882	23893881	744	0.379104
BnaWGS-1007	BnaA10G0244800ZS	scaffoldA10	23803882	23903881	684	0.40209
BnaWGS-1007	BnaA10G0244800ZS	scaffoldA10	23813882	23913881	623	0.415026
BnaWGS-1007	BnaA10G0244800ZS	scaffoldA10	23823882	23923881	567	0.412685
BnaWGS-1007	BnaA10G0244800ZS	scaffoldA10	23833882	23933881	546	0.440674
BnaWGS-1007	BnaA10G0244800ZS	scaffoldA10	23843882	23943881	580	0.450115
BnaWGS-1007	BnaA10G0244800ZS	scaffoldA10	23853882	23953881	513	0.46605
BnaWGS-1007	BnaA10G0244800ZS	scaffoldA10	23863882	23963881	409	0.469901
BnaWGS-1007	BnaA10G0244800ZS	scaffoldA10	23873882	23973881	432	0.522463

For Review Only

BnaWGS-1007	BnaA10G0244800ZS	scaffoldA10	23883882	23983881	588	0.407645
BnaWGS-1007	BnaA10G0244800ZS	scaffoldA10	23893882	23993881	585	0.409586
BnaWGS-1007	BnaA10G0244800ZS	scaffoldA10	23903882	24003881	620	0.377573
BnaWGS-1007	BnaA10G0244800ZS	scaffoldA10	23913882	24013881	659	0.361574
BnaWGS-1007	BnaA10G0244800ZS	scaffoldA10	23923882	24023881	750	0.350563
BnaWGS-1007	BnaA10G0244800ZS	scaffoldA10	23933882	24033881	747	0.338975
BnaWGS-1007	BnaA10G0244800ZS	scaffoldA10	23943882	24043881	823	0.319481
BnaWGS-1007	BnaA10G0244800ZS	scaffoldA10	23953882	24053881	831	0.304922
BnaWGS-1007	BnaA10G0244800ZS	scaffoldA10	23963882	24063881	919	0.302234
BnaWGS-1007	BnaA10G0244800ZS	scaffoldA10	23973882	24073881	1039	0.273461
BnaWGS-1007	BnaA10G0244800ZS	scaffoldA10	23983882	24083881	1020	0.258935
BnaWGS-1007	BnaA10G0244800ZS	scaffoldA10	23993882	24093881	1197	0.214933
BnaWGS-1007	BnaA10G0244800ZS	scaffoldA10	24003882	24103881	1342	0.214825
BnaWGS-1007	BnaA10G0244800ZS	scaffoldA10	24013882	24113881	1400	0.207943
BnaWGS-1007	BnaA10G0244800ZS	scaffoldA10	24023882	24123881	1506	0.192928
BnaWGS-1007	BnaA10G0244800ZS	scaffoldA10	24033882	24133881	1553	0.192586
BnaWGS-1007	BnaA10G0244800ZS	scaffoldA10	24043882	24143881	1523	0.202378
BnaWGS-1007	BnaA10G0244800ZS	scaffoldA10	24053882	24153881	1592	0.205509
BnaWGS-1007	BnaA10G0244800ZS	scaffoldA10	24063882	24163881	1607	0.205968
BnaWGS-1007	BnaA10G0244800ZS	scaffoldA10	24073882	24173881	1645	0.202596
BnaWGS-1007	BnaA10G0244800ZS	scaffoldA10	24083882	24183881	1712	0.216368
BnaWGS-1007	BnaA10G0244800ZS	scaffoldA10	24093882	24193881	1794	0.222544
BnaWGS-1007	BnaA10G0244800ZS	scaffoldA10	24103882	24203881	1636	0.219569
BnaWGS-1007	BnaA10G0244800ZS	scaffoldA10	24113882	24213881	1549	0.213916
BnaWGS-1007	BnaA10G0244800ZS	scaffoldA10	24123882	24223881	1535	0.217767
BnaWGS-1007	BnaA10G0244800ZS	scaffoldA10	24133882	24233881	1641	0.21291
BnaWGS-1007	BnaA10G0244800ZS	scaffoldA10	24143882	24243881	1663	0.191111
BnaWGS-1007	BnaA10G0244800ZS	scaffoldA10	24153882	24253881	1761	0.183392
BnaWGS-1007	BnaA10G0244800ZS	scaffoldA10	24163882	24263881	1778	0.182923
BnaWGS-1007	BnaA10G0244800ZS	scaffoldA10	24173882	24273881	1804	0.200015
BnaWGS-1007	BnaA10G0244800ZS	scaffoldA10	24183882	24283881	1655	0.198749
BnaWGS-1007	BnaA10G0244800ZS	scaffoldA10	24193882	24293881	1439	0.218299
BnaWGS-1007	BnaA10G0244800ZS	scaffoldA10	24203882	24303881	1554	0.228428
BnaWGS-1007	BnaA10G0244800ZS	scaffoldA10	24213882	24313881	1699	0.23299
BnaWGS-1007	BnaA10G0244800ZS	scaffoldA10	24223882	24323881	1713	0.239482
BnaWGS-1007	BnaA10G0244800ZS	scaffoldA10	24233882	24333881	1662	0.244995
BnaWGS-1007	BnaA10G0244800ZS	scaffoldA10	24243882	24343881	1729	0.24631
BnaWGS-1007	BnaA10G0244800ZS	scaffoldA10	24253882	24353881	1748	0.254862
BnaWGS-1007	BnaA10G0244800ZS	scaffoldA10	24263882	24363881	1674	0.248352
BnaWGS-1007	BnaA10G0244800ZS	scaffoldA10	24273882	24373881	1603	0.265754
BnaWGS-1007	BnaA10G0244800ZS	scaffoldA10	24283882	24383881	1647	0.274146
BnaWGS-1007	BnaA10G0244800ZS	scaffoldA10	24293882	24393881	1623	0.267516
BnaWGS-1007	BnaA10G0244800ZS	scaffoldA10	24303882	24403881	1448	0.267552
BnaWGS-1007	BnaA10G0244800ZS	scaffoldA10	24313882	24413881	1427	0.268704
BnaWGS-1007	BnaA10G0244800ZS	scaffoldA10	24323882	24423881	1434	0.251816
BnaWGS-1007	BnaA10G0244800ZS	scaffoldA10	24333882	24433881	1449	0.254902
BnaWGS-1007	BnaA10G0244800ZS	scaffoldA10	24343882	24443881	1324	0.270852
BnaWGS-1007	BnaA10G0244800ZS	scaffoldA10	24353882	24453881	1165	0.286518
BnaWGS-1007	BnaA10G0244800ZS	scaffoldA10	24363882	24463881	1306	0.266912
BnaWGS-1007	BnaA10G0244800ZS	scaffoldA10	24373882	24473881	1314	0.193121
BnaWGS-1007	BnaA10G0244800ZS	scaffoldA10	24383882	24483881	1244	0.171333
BnaWGS-1007	BnaA10G0244800ZS	scaffoldA10	24393882	24493881	1326	0.163634
BnaWGS-1007	BnaA10G0244800ZS	scaffoldA10	24403882	24503881	1382	0.159841
BnaWGS-1007	BnaA10G0244800ZS	scaffoldA10	24413882	24513881	1286	0.153239
BnaWGS-1007	BnaA10G0244800ZS	scaffoldA10	24423882	24523881	1126	0.172205
BnaWGS-1007	BnaA10G0244800ZS	scaffoldA10	24433882	24533881	1033	0.162658
BnaWGS-1007	BnaA10G0244800ZS	scaffoldA10	24443882	24543881	1049	0.15211
BnaWGS-1007	BnaA10G0244800ZS	scaffoldA10	24453882	24553881	1114	0.13962
BnaWGS-1007	BnaA10G0244800ZS	scaffoldA10	24463882	24563881	1052	0.146598
BnaWGS-1007	BnaA10G0244800ZS	scaffoldA10	24473882	24573881	1023	0.171376

For Review Only

BnaWGS-1007	BnaA10G0244800ZS	scaffoldA10	24483882	24583881	1056	0.180401
BnaWGS-1007	BnaA10G0244800ZS	scaffoldA10	24493882	24593881	980	0.190767
BnaWGS-1007	BnaA10G0244800ZS	scaffoldA10	24503882	24603881	1008	0.20643
BnaWGS-1007	BnaA10G0244800ZS	scaffoldA10	24513882	24613881	1025	0.207125
BnaWGS-1007	BnaA10G0244800ZS	scaffoldA10	24523882	24623881	1086	0.198382
BnaWGS-1007	BnaA10G0244800ZS	scaffoldA10	24533882	24633881	1145	0.189132
BnaWGS-1007	BnaA10G0244800ZS	scaffoldA10	24543882	24643881	1182	0.201161
BnaWGS-1007	BnaA10G0244800ZS	scaffoldA10	24553882	24653881	1172	0.209587
BnaWGS-1007	BnaA10G0244800ZS	scaffoldA10	24563882	24663881	1138	0.232665
BnaWGS-1007	BnaA10G0244800ZS	scaffoldA10	24573882	24673881	1024	0.236152
BnaWGS-1007	BnaA10G0244800ZS	scaffoldA10	24583882	24683881	1010	0.231328
BnaWGS-1007	BnaA10G0244800ZS	scaffoldA10	24593882	24693881	1040	0.221892
BnaWGS-1007	BnaA10G0244800ZS	scaffoldA10	24603882	24703881	1011	0.209371
BnaWGS-1007	BnaA10G0244800ZS	scaffoldA10	24613882	24713881	980	0.203869
BnaWGS-1007	BnaA10G0244800ZS	scaffoldA10	24623882	24723881	910	0.205477
BnaWGS-1007	BnaA10G0244800ZS	scaffoldA10	24633882	24733881	915	0.208855
BnaWGS-1007	BnaA10G0244800ZS	scaffoldA10	24643882	24743881	913	0.201728
BnaWGS-1007	BnaA10G0244800ZS	scaffoldA10	24653882	24753881	932	0.186599
BnaWGS-1007	BnaA10G0244800ZS	scaffoldA10	24663882	24763881	838	0.155696
BnaWGS-1007	BnaA10G0244800ZS	scaffoldA10	24673882	24773881	802	0.139908
BnaWGS-1007	BnaA10G0244800ZS	scaffoldA10	24683882	24783881	849	0.130953
BnaWGS-1007	BnaA10G0244800ZS	scaffoldA10	24693882	24793881	845	0.150081
BnaWGS-1007	BnaA10G0244800ZS	scaffoldA10	24703882	24803881	783	0.149459
BnaWGS-1007	BnaA10G0244800ZS	scaffoldA10	24713882	24813881	778	0.167445
BnaWGS-1007	BnaA10G0244800ZS	scaffoldA10	24723882	24823881	926	0.207536
BnaWGS-1007	BnaA10G0244800ZS	scaffoldA10	24733882	24833881	935	0.224391
BnaWGS-1007	BnaA10G0244800ZS	scaffoldA10	24743882	24843881	961	0.220837
BnaWGS-1007	BnaA10G0244800ZS	scaffoldA10	24753882	24853881	910	0.227071
BnaWGS-1007	BnaA10G0244800ZS	scaffoldA10	24763882	24863881	923	0.230834
BnaWGS-1007	BnaA10G0244800ZS	scaffoldA10	24773882	24873881	1033	0.227356
BnaWGS-1007	BnaA10G0244800ZS	scaffoldA10	24783882	24883881	932	0.255226
BnaWGS-1007	BnaA10G0244800ZS	scaffoldA10	24793882	24893881	899	0.253833
BnaWGS-1007	BnaA10G0244800ZS	scaffoldA10	24803882	24903881	1034	0.245225
BnaWGS-1007	BnaA10G0244800ZS	scaffoldA10	24813882	24913881	1090	0.254898
BnaWGS-1007	BnaA10G0244800ZS	scaffoldA10	24823882	24923881	1222	0.208797
BnaWGS-1007	BnaA10G0244800ZS	scaffoldA10	24833882	24933881	1312	0.206235
BnaWGS-1007	BnaA10G0244800ZS	scaffoldA10	24843882	24943881	1248	0.221743
BnaWGS-1007	BnaA10G0244800ZS	scaffoldA10	24853882	24947904	1187	0.226829
BnaWGS-1007	BnaA10G0244800ZS	scaffoldA10	22943882	23043881	511	0.018671
BnaWGS-1007	BnaA10G0244800ZS	scaffoldA10	22953882	23053881	488	0.0173
BnaWGS-1007	BnaA10G0244800ZS	scaffoldA10	22963882	23063881	489	0.020325
BnaWGS-1007	BnaA10G0244800ZS	scaffoldA10	22973882	23073881	512	0.020074
BnaWGS-1007	BnaA10G0244800ZS	scaffoldA10	22983882	23083881	485	0.020892
BnaWGS-1007	BnaA10G0244800ZS	scaffoldA10	22993882	23093881	513	0.064439
BnaWGS-1007	BnaA10G0244800ZS	scaffoldA10	23003882	23103881	536	0.106952
BnaWGS-1007	BnaA10G0244800ZS	scaffoldA10	23013882	23113881	569	0.134043
BnaWGS-1007	BnaA10G0244800ZS	scaffoldA10	23023882	23123881	568	0.140853
BnaWGS-1007	BnaA10G0244800ZS	scaffoldA10	23033882	23133881	655	0.166868
BnaWGS-1007	BnaA10G0244800ZS	scaffoldA10	23043882	23143881	746	0.182478
BnaWGS-1007	BnaA10G0244800ZS	scaffoldA10	23053882	23153881	833	0.18123
BnaWGS-1007	BnaA10G0244800ZS	scaffoldA10	23063882	23163881	942	0.179649
BnaWGS-1007	BnaA10G0244800ZS	scaffoldA10	23073882	23173881	981	0.188514
BnaWGS-1007	BnaA10G0244800ZS	scaffoldA10	23083882	23183881	1068	0.185871
BnaWGS-1007	BnaA10G0244800ZS	scaffoldA10	23093882	23193881	1086	0.195286
BnaWGS-1007	BnaA10G0244800ZS	scaffoldA10	23103882	23203881	1103	0.193754
BnaWGS-1007	BnaA10G0244800ZS	scaffoldA10	23113882	23213881	1212	0.190705
BnaWGS-1007	BnaA10G0244800ZS	scaffoldA10	23123882	23223881	1293	0.192722
BnaWGS-1007	BnaA10G0244800ZS	scaffoldA10	23133882	23233881	1177	0.19103
BnaWGS-1007	BnaA10G0244800ZS	scaffoldA10	23143882	23243881	1046	0.187769
BnaWGS-1007	BnaA10G0244800ZS	scaffoldA10	23153882	23253881	953	0.190039

For Review Only

BnaWGS-1007	BnaA10G0244800ZS	scaffoldA10	23163882	23263881	900	0.188834
BnaWGS-1007	BnaA10G0244800ZS	scaffoldA10	23173882	23273881	837	0.188683
BnaWGS-1007	BnaA10G0244800ZS	scaffoldA10	23183882	23283881	773	0.18961
BnaWGS-1007	BnaA10G0244800ZS	scaffoldA10	23193882	23293881	739	0.170316
BnaWGS-1007	BnaA10G0244800ZS	scaffoldA10	23203882	23303881	713	0.153449
BnaWGS-1007	BnaA10G0244800ZS	scaffoldA10	23213882	23313881	594	0.122519
BnaWGS-1007	BnaA10G0244800ZS	scaffoldA10	23223882	23323881	618	0.067031
BnaWGS-1007	BnaA10G0244800ZS	scaffoldA10	23233882	23333881	680	0.036778
BnaWGS-1007	BnaA10G0244800ZS	scaffoldA10	23243882	23343881	825	0.036529
BnaWGS-1007	BnaA10G0244800ZS	scaffoldA10	23253882	23353881	878	0.040169
BnaWGS-1007	BnaA10G0244800ZS	scaffoldA10	23263882	23363881	800	0.044847
BnaWGS-1007	BnaA10G0244800ZS	scaffoldA10	23273882	23373881	785	0.051038
BnaWGS-1007	BnaA10G0244800ZS	scaffoldA10	23283882	23383881	747	0.052587
BnaWGS-1007	BnaA10G0244800ZS	scaffoldA10	23293882	23393881	670	0.052625
BnaWGS-1007	BnaA10G0244800ZS	scaffoldA10	23303882	23403881	665	0.049257
BnaWGS-1007	BnaA10G0244800ZS	scaffoldA10	23313882	23413881	668	0.054865
BnaWGS-1007	BnaA10G0244800ZS	scaffoldA10	23323882	23423881	605	0.067659
BnaWGS-1007	BnaA10G0244800ZS	scaffoldA10	23333882	23433881	587	0.086014
BnaWGS-1007	BnaA10G0244800ZS	scaffoldA10	23343882	23443881	494	0.093101
BnaWGS-1007	BnaA10G0244800ZS	scaffoldA10	23353882	23453881	461	0.093413
BnaWGS-1007	BnaA10G0244800ZS	scaffoldA10	23363882	23463881	497	0.09191
BnaWGS-1007	BnaA10G0244800ZS	scaffoldA10	23373882	23473881	521	0.085536
BnaWGS-1007	BnaA10G0244800ZS	scaffoldA10	23383882	23483881	603	0.095794
BnaWGS-1007	BnaA10G0244800ZS	scaffoldA10	23393882	23493881	685	0.107149
BnaWGS-1007	BnaA10G0244800ZS	scaffoldA10	23403882	23503881	733	0.133842
BnaWGS-1007	BnaA10G0244800ZS	scaffoldA10	23413882	23513881	872	0.14232
BnaWGS-1007	BnaA10G0244800ZS	scaffoldA10	23423882	23523881	894	0.139924
BnaWGS-1007	BnaA10G0244800ZS	scaffoldA10	23433882	23533881	872	0.145348
BnaWGS-1007	BnaA10G0244800ZS	scaffoldA10	23443882	23543881	1015	0.153229
BnaWGS-1007	BnaA10G0244800ZS	scaffoldA10	23453882	23553881	1068	0.155954
BnaWGS-1007	BnaA10G0244800ZS	scaffoldA10	23463882	23563881	1090	0.153689
BnaWGS-1007	BnaA10G0244800ZS	scaffoldA10	23473882	23573881	1074	0.1568
BnaWGS-1007	BnaA10G0244800ZS	scaffoldA10	23483882	23583881	1002	0.156284
BnaWGS-1007	BnaA10G0244800ZS	scaffoldA10	23493882	23593881	978	0.15472
BnaWGS-1007	BnaA10G0244800ZS	scaffoldA10	23503882	23603881	999	0.149665
BnaWGS-1007	BnaA10G0244800ZS	scaffoldA10	23513882	23613881	831	0.151079
BnaWGS-1007	BnaA10G0244800ZS	scaffoldA10	23523882	23623881	851	0.160951
BnaWGS-1007	BnaA10G0244800ZS	scaffoldA10	23533882	23633881	934	0.160382
BnaWGS-1007	BnaA10G0244800ZS	scaffoldA10	23543882	23643881	830	0.160766
BnaWGS-1007	BnaA10G0244800ZS	scaffoldA10	23553882	23653881	856	0.158175
BnaWGS-1007	BnaA10G0244800ZS	scaffoldA10	23563882	23663881	890	0.147822
BnaWGS-1007	BnaA10G0244800ZS	scaffoldA10	23573882	23673881	1076	0.143743
BnaWGS-1007	BnaA10G0244800ZS	scaffoldA10	23583882	23683881	1220	0.138185
BnaWGS-1007	BnaA10G0244800ZS	scaffoldA10	23593882	23693881	1194	0.137683
BnaWGS-1007	BnaA10G0244800ZS	scaffoldA10	23603882	23703881	1129	0.12935
BnaWGS-1007	BnaA10G0244800ZS	scaffoldA10	23613882	23713881	1189	0.134402
BnaWGS-1007	BnaA10G0244800ZS	scaffoldA10	23623882	23723881	1142	0.125366
BnaWGS-1007	BnaA10G0244800ZS	scaffoldA10	23633882	23733881	1113	0.129599
BnaWGS-1007	BnaA10G0244800ZS	scaffoldA10	23643882	23743881	1172	0.140145
BnaWGS-1007	BnaA10G0244800ZS	scaffoldA10	23653882	23753881	1237	0.150352
BnaWGS-1007	BnaA10G0244800ZS	scaffoldA10	23663882	23763881	1262	0.156757
BnaWGS-1007	BnaA10G0244800ZS	scaffoldA10	23673882	23773881	1134	0.175551
BnaWGS-1007	BnaA10G0244800ZS	scaffoldA10	23683882	23783881	1076	0.186863
BnaWGS-1007	BnaA10G0244800ZS	scaffoldA10	23693882	23793881	1115	0.19089
BnaWGS-1007	BnaA10G0244800ZS	scaffoldA10	23703882	23803881	1167	0.201412
BnaWGS-1007	BnaA10G0244800ZS	scaffoldA10	23713882	23813881	1176	0.199375
BnaWGS-1007	BnaA10G0244800ZS	scaffoldA10	23723882	23823881	1178	0.206602
BnaWGS-1007	BnaA10G0244800ZS	scaffoldA10	23733882	23833881	1122	0.20959
BnaWGS-1007	BnaA10G0244800ZS	scaffoldA10	23743882	23843881	998	0.204503
BnaWGS-1007	BnaA10G0244800ZS	scaffoldA10	23753882	23853881	913	0.204853

For Review Only

BnaWGS-1007	BnaA10G0244800ZS	scaffoldA10	23763882	23863881	916	0.219713
BnaWGS-1007	BnaA10G0244800ZS	scaffoldA10	23773882	23873881	848	0.209779
BnaWGS-1007	BnaA10G0244800ZS	scaffoldA10	23783882	23883881	793	0.217472
BnaWGS-1007	BnaA10G0244800ZS	scaffoldA10	23793882	23893881	744	0.225067
BnaWGS-1007	BnaA10G0244800ZS	scaffoldA10	23803882	23903881	684	0.224497
BnaWGS-1007	BnaA10G0244800ZS	scaffoldA10	23813882	23913881	623	0.234463
BnaWGS-1007	BnaA10G0244800ZS	scaffoldA10	23823882	23923881	567	0.235686
BnaWGS-1007	BnaA10G0244800ZS	scaffoldA10	23833882	23933881	546	0.230507
BnaWGS-1007	BnaA10G0244800ZS	scaffoldA10	23843882	23943881	580	0.23397
BnaWGS-1007	BnaA10G0244800ZS	scaffoldA10	23853882	23953881	513	0.244735
BnaWGS-1007	BnaA10G0244800ZS	scaffoldA10	23863882	23963881	409	0.254858
BnaWGS-1007	BnaA10G0244800ZS	scaffoldA10	23873882	23973881	432	0.275435
BnaWGS-1007	BnaA10G0244800ZS	scaffoldA10	23883882	23983881	588	0.241211
BnaWGS-1007	BnaA10G0244800ZS	scaffoldA10	23893882	23993881	585	0.236329
BnaWGS-1007	BnaA10G0244800ZS	scaffoldA10	23903882	24003881	620	0.243319
BnaWGS-1007	BnaA10G0244800ZS	scaffoldA10	23913882	24013881	659	0.2359
BnaWGS-1007	BnaA10G0244800ZS	scaffoldA10	23923882	24023881	750	0.23957
BnaWGS-1007	BnaA10G0244800ZS	scaffoldA10	23933882	24033881	747	0.233334
BnaWGS-1007	BnaA10G0244800ZS	scaffoldA10	23943882	24043881	823	0.221481
BnaWGS-1007	BnaA10G0244800ZS	scaffoldA10	23953882	24053881	831	0.217841
BnaWGS-1007	BnaA10G0244800ZS	scaffoldA10	23963882	24063881	919	0.218771
BnaWGS-1007	BnaA10G0244800ZS	scaffoldA10	23973882	24073881	1039	0.204487
BnaWGS-1007	BnaA10G0244800ZS	scaffoldA10	23983882	24083881	1020	0.205743
BnaWGS-1007	BnaA10G0244800ZS	scaffoldA10	23993882	24093881	1197	0.210937
BnaWGS-1007	BnaA10G0244800ZS	scaffoldA10	24003882	24103881	1342	0.190034
BnaWGS-1007	BnaA10G0244800ZS	scaffoldA10	24013882	24113881	1400	0.191711
BnaWGS-1007	BnaA10G0244800ZS	scaffoldA10	24023882	24123881	1506	0.181963
BnaWGS-1007	BnaA10G0244800ZS	scaffoldA10	24033882	24133881	1553	0.183072
BnaWGS-1007	BnaA10G0244800ZS	scaffoldA10	24043882	24143881	1523	0.193422
BnaWGS-1007	BnaA10G0244800ZS	scaffoldA10	24053882	24153881	1592	0.194757
BnaWGS-1007	BnaA10G0244800ZS	scaffoldA10	24063882	24163881	1607	0.186883
BnaWGS-1007	BnaA10G0244800ZS	scaffoldA10	24073882	24173881	1645	0.167919
BnaWGS-1007	BnaA10G0244800ZS	scaffoldA10	24083882	24183881	1712	0.172905
BnaWGS-1007	BnaA10G0244800ZS	scaffoldA10	24093882	24193881	1794	0.170896
BnaWGS-1007	BnaA10G0244800ZS	scaffoldA10	24103882	24203881	1636	0.179733
BnaWGS-1007	BnaA10G0244800ZS	scaffoldA10	24113882	24213881	1549	0.180631
BnaWGS-1007	BnaA10G0244800ZS	scaffoldA10	24123882	24223881	1535	0.184215
BnaWGS-1007	BnaA10G0244800ZS	scaffoldA10	24133882	24233881	1641	0.186747
BnaWGS-1007	BnaA10G0244800ZS	scaffoldA10	24143882	24243881	1663	0.175989
BnaWGS-1007	BnaA10G0244800ZS	scaffoldA10	24153882	24253881	1761	0.16715
BnaWGS-1007	BnaA10G0244800ZS	scaffoldA10	24163882	24263881	1778	0.173555
BnaWGS-1007	BnaA10G0244800ZS	scaffoldA10	24173882	24273881	1804	0.185675
BnaWGS-1007	BnaA10G0244800ZS	scaffoldA10	24183882	24283881	1655	0.183139
BnaWGS-1007	BnaA10G0244800ZS	scaffoldA10	24193882	24293881	1439	0.170714
BnaWGS-1007	BnaA10G0244800ZS	scaffoldA10	24203882	24303881	1554	0.177647
BnaWGS-1007	BnaA10G0244800ZS	scaffoldA10	24213882	24313881	1699	0.15942
BnaWGS-1007	BnaA10G0244800ZS	scaffoldA10	24223882	24323881	1713	0.153503
BnaWGS-1007	BnaA10G0244800ZS	scaffoldA10	24233882	24333881	1662	0.145673
BnaWGS-1007	BnaA10G0244800ZS	scaffoldA10	24243882	24343881	1729	0.143177
BnaWGS-1007	BnaA10G0244800ZS	scaffoldA10	24253882	24353881	1748	0.144383
BnaWGS-1007	BnaA10G0244800ZS	scaffoldA10	24263882	24363881	1674	0.133695
BnaWGS-1007	BnaA10G0244800ZS	scaffoldA10	24273882	24373881	1603	0.142708
BnaWGS-1007	BnaA10G0244800ZS	scaffoldA10	24283882	24383881	1647	0.152786
BnaWGS-1007	BnaA10G0244800ZS	scaffoldA10	24293882	24393881	1623	0.158299
BnaWGS-1007	BnaA10G0244800ZS	scaffoldA10	24303882	24403881	1448	0.144252
BnaWGS-1007	BnaA10G0244800ZS	scaffoldA10	24313882	24413881	1427	0.162256
BnaWGS-1007	BnaA10G0244800ZS	scaffoldA10	24323882	24423881	1434	0.157133
BnaWGS-1007	BnaA10G0244800ZS	scaffoldA10	24333882	24433881	1449	0.159451
BnaWGS-1007	BnaA10G0244800ZS	scaffoldA10	24343882	24443881	1324	0.1624
BnaWGS-1007	BnaA10G0244800ZS	scaffoldA10	24353882	24453881	1165	0.173838

For Review Only

BnaWGS-1007	BnaA10G0244800ZS	scaffoldA10	24363882	24463881	1306	0.178759
BnaWGS-1007	BnaA10G0244800ZS	scaffoldA10	24373882	24473881	1314	0.176256
BnaWGS-1007	BnaA10G0244800ZS	scaffoldA10	24383882	24483881	1244	0.162981
BnaWGS-1007	BnaA10G0244800ZS	scaffoldA10	24393882	24493881	1326	0.161647
BnaWGS-1007	BnaA10G0244800ZS	scaffoldA10	24403882	24503881	1382	0.161443
BnaWGS-1007	BnaA10G0244800ZS	scaffoldA10	24413882	24513881	1286	0.155615
BnaWGS-1007	BnaA10G0244800ZS	scaffoldA10	24423882	24523881	1126	0.165332
BnaWGS-1007	BnaA10G0244800ZS	scaffoldA10	24433882	24533881	1033	0.165304
BnaWGS-1007	BnaA10G0244800ZS	scaffoldA10	24443882	24543881	1049	0.157998
BnaWGS-1007	BnaA10G0244800ZS	scaffoldA10	24453882	24553881	1114	0.145657
BnaWGS-1007	BnaA10G0244800ZS	scaffoldA10	24463882	24563881	1052	0.136195
BnaWGS-1007	BnaA10G0244800ZS	scaffoldA10	24473882	24573881	1023	0.146183
BnaWGS-1007	BnaA10G0244800ZS	scaffoldA10	24483882	24583881	1056	0.155868
BnaWGS-1007	BnaA10G0244800ZS	scaffoldA10	24493882	24593881	980	0.170696
BnaWGS-1007	BnaA10G0244800ZS	scaffoldA10	24503882	24603881	1008	0.201784
BnaWGS-1007	BnaA10G0244800ZS	scaffoldA10	24513882	24613881	1025	0.206959
BnaWGS-1007	BnaA10G0244800ZS	scaffoldA10	24523882	24623881	1086	0.207115
BnaWGS-1007	BnaA10G0244800ZS	scaffoldA10	24533882	24633881	1145	0.204524
BnaWGS-1007	BnaA10G0244800ZS	scaffoldA10	24543882	24643881	1182	0.205803
BnaWGS-1007	BnaA10G0244800ZS	scaffoldA10	24553882	24653881	1172	0.221728
BnaWGS-1007	BnaA10G0244800ZS	scaffoldA10	24563882	24663881	1138	0.24584
BnaWGS-1007	BnaA10G0244800ZS	scaffoldA10	24573882	24673881	1024	0.242076
BnaWGS-1007	BnaA10G0244800ZS	scaffoldA10	24583882	24683881	1010	0.229434
BnaWGS-1007	BnaA10G0244800ZS	scaffoldA10	24593882	24693881	1040	0.214876
BnaWGS-1007	BnaA10G0244800ZS	scaffoldA10	24603882	24703881	1011	0.192589
BnaWGS-1007	BnaA10G0244800ZS	scaffoldA10	24613882	24713881	980	0.191888
BnaWGS-1007	BnaA10G0244800ZS	scaffoldA10	24623882	24723881	910	0.186282
BnaWGS-1007	BnaA10G0244800ZS	scaffoldA10	24633882	24733881	915	0.176421
BnaWGS-1007	BnaA10G0244800ZS	scaffoldA10	24643882	24743881	913	0.172235
BnaWGS-1007	BnaA10G0244800ZS	scaffoldA10	24653882	24753881	932	0.158096
BnaWGS-1007	BnaA10G0244800ZS	scaffoldA10	24663882	24763881	838	0.135084
BnaWGS-1007	BnaA10G0244800ZS	scaffoldA10	24673882	24773881	802	0.131786
BnaWGS-1007	BnaA10G0244800ZS	scaffoldA10	24683882	24783881	849	0.147046
BnaWGS-1007	BnaA10G0244800ZS	scaffoldA10	24693882	24793881	845	0.156343
BnaWGS-1007	BnaA10G0244800ZS	scaffoldA10	24703882	24803881	783	0.149362
BnaWGS-1007	BnaA10G0244800ZS	scaffoldA10	24713882	24813881	778	0.152466
BnaWGS-1007	BnaA10G0244800ZS	scaffoldA10	24723882	24823881	926	0.192739
BnaWGS-1007	BnaA10G0244800ZS	scaffoldA10	24733882	24833881	935	0.222378
BnaWGS-1007	BnaA10G0244800ZS	scaffoldA10	24743882	24843881	961	0.244946
BnaWGS-1007	BnaA10G0244800ZS	scaffoldA10	24753882	24853881	910	0.263095
BnaWGS-1007	BnaA10G0244800ZS	scaffoldA10	24763882	24863881	923	0.268697
BnaWGS-1007	BnaA10G0244800ZS	scaffoldA10	24773882	24873881	1033	0.273745
BnaWGS-1007	BnaA10G0244800ZS	scaffoldA10	24783882	24883881	932	0.291833
BnaWGS-1007	BnaA10G0244800ZS	scaffoldA10	24793882	24893881	899	0.293297
BnaWGS-1007	BnaA10G0244800ZS	scaffoldA10	24803882	24903881	1034	0.287844
BnaWGS-1007	BnaA10G0244800ZS	scaffoldA10	24813882	24913881	1090	0.290469
BnaWGS-1007	BnaA10G0244800ZS	scaffoldA10	24823882	24923881	1222	0.30487
BnaWGS-1007	BnaA10G0244800ZS	scaffoldA10	24833882	24933881	1312	0.292421
BnaWGS-1007	BnaA10G0244800ZS	scaffoldA10	24843882	24943881	1248	0.294184
BnaWGS-1007	BnaA10G0244800ZS	scaffoldA10	24853882	24947904	1187	0.294915
BnaWGS-655	BnaA10G0244800ZS	scaffoldA10	22943882	23043881	663	0.033797
BnaWGS-655	BnaA10G0244800ZS	scaffoldA10	22953882	23053881	624	0.035505
BnaWGS-655	BnaA10G0244800ZS	scaffoldA10	22963882	23063881	607	0.039357
BnaWGS-655	BnaA10G0244800ZS	scaffoldA10	22973882	23073881	600	0.045966
BnaWGS-655	BnaA10G0244800ZS	scaffoldA10	22983882	23083881	564	0.049464
BnaWGS-655	BnaA10G0244800ZS	scaffoldA10	22993882	23093881	591	0.130607
BnaWGS-655	BnaA10G0244800ZS	scaffoldA10	23003882	23103881	625	0.190521
BnaWGS-655	BnaA10G0244800ZS	scaffoldA10	23013882	23113881	625	0.230263
BnaWGS-655	BnaA10G0244800ZS	scaffoldA10	23023882	23123881	616	0.237519
BnaWGS-655	BnaA10G0244800ZS	scaffoldA10	23033882	23133881	718	0.278732

For Review Only

BnaWGS-655	BnaA10G0244800ZS	scaffoldA10	23043882	23143881	856	0.298022
BnaWGS-655	BnaA10G0244800ZS	scaffoldA10	23053882	23153881	1085	0.2739
BnaWGS-655	BnaA10G0244800ZS	scaffoldA10	23063882	23163881	1274	0.268542
BnaWGS-655	BnaA10G0244800ZS	scaffoldA10	23073882	23173881	1354	0.265586
BnaWGS-655	BnaA10G0244800ZS	scaffoldA10	23083882	23183881	1433	0.261884
BnaWGS-655	BnaA10G0244800ZS	scaffoldA10	23093882	23193881	1528	0.259732
BnaWGS-655	BnaA10G0244800ZS	scaffoldA10	23103882	23203881	1613	0.247956
BnaWGS-655	BnaA10G0244800ZS	scaffoldA10	23113882	23213881	1821	0.245682
BnaWGS-655	BnaA10G0244800ZS	scaffoldA10	23123882	23223881	1914	0.249779
BnaWGS-655	BnaA10G0244800ZS	scaffoldA10	23133882	23233881	1906	0.231278
BnaWGS-655	BnaA10G0244800ZS	scaffoldA10	23143882	23243881	1740	0.215274
BnaWGS-655	BnaA10G0244800ZS	scaffoldA10	23153882	23253881	1621	0.21292
BnaWGS-655	BnaA10G0244800ZS	scaffoldA10	23163882	23263881	1493	0.205139
BnaWGS-655	BnaA10G0244800ZS	scaffoldA10	23173882	23273881	1412	0.205336
BnaWGS-655	BnaA10G0244800ZS	scaffoldA10	23183882	23283881	1338	0.204015
BnaWGS-655	BnaA10G0244800ZS	scaffoldA10	23193882	23293881	1150	0.192137
BnaWGS-655	BnaA10G0244800ZS	scaffoldA10	23203882	23303881	1074	0.175871
BnaWGS-655	BnaA10G0244800ZS	scaffoldA10	23213882	23313881	900	0.109577
BnaWGS-655	BnaA10G0244800ZS	scaffoldA10	23223882	23323881	928	0.039905
BnaWGS-655	BnaA10G0244800ZS	scaffoldA10	23233882	23333881	897	0.019975
BnaWGS-655	BnaA10G0244800ZS	scaffoldA10	23243882	23343881	1111	0.026118
BnaWGS-655	BnaA10G0244800ZS	scaffoldA10	23253882	23353881	1092	0.031515
BnaWGS-655	BnaA10G0244800ZS	scaffoldA10	23263882	23363881	1068	0.033434
BnaWGS-655	BnaA10G0244800ZS	scaffoldA10	23273882	23373881	1060	0.038357
BnaWGS-655	BnaA10G0244800ZS	scaffoldA10	23283882	23383881	1028	0.039523
BnaWGS-655	BnaA10G0244800ZS	scaffoldA10	23293882	23393881	1068	0.03863
BnaWGS-655	BnaA10G0244800ZS	scaffoldA10	23303882	23403881	1068	0.03929
BnaWGS-655	BnaA10G0244800ZS	scaffoldA10	23313882	23413881	1032	0.045137
BnaWGS-655	BnaA10G0244800ZS	scaffoldA10	23323882	23423881	957	0.058439
BnaWGS-655	BnaA10G0244800ZS	scaffoldA10	23333882	23433881	901	0.077337
BnaWGS-655	BnaA10G0244800ZS	scaffoldA10	23343882	23443881	726	0.08619
BnaWGS-655	BnaA10G0244800ZS	scaffoldA10	23353882	23453881	661	0.091938
BnaWGS-655	BnaA10G0244800ZS	scaffoldA10	23363882	23463881	689	0.099827
BnaWGS-655	BnaA10G0244800ZS	scaffoldA10	23373882	23473881	763	0.100997
BnaWGS-655	BnaA10G0244800ZS	scaffoldA10	23383882	23483881	866	0.115759
BnaWGS-655	BnaA10G0244800ZS	scaffoldA10	23393882	23493881	880	0.138097
BnaWGS-655	BnaA10G0244800ZS	scaffoldA10	23403882	23503881	901	0.179774
BnaWGS-655	BnaA10G0244800ZS	scaffoldA10	23413882	23513881	1082	0.211602
BnaWGS-655	BnaA10G0244800ZS	scaffoldA10	23423882	23523881	1122	0.218791
BnaWGS-655	BnaA10G0244800ZS	scaffoldA10	23433882	23533881	1098	0.226386
BnaWGS-655	BnaA10G0244800ZS	scaffoldA10	23443882	23543881	1317	0.232187
BnaWGS-655	BnaA10G0244800ZS	scaffoldA10	23453882	23553881	1372	0.232873
BnaWGS-655	BnaA10G0244800ZS	scaffoldA10	23463882	23563881	1385	0.230979
BnaWGS-655	BnaA10G0244800ZS	scaffoldA10	23473882	23573881	1360	0.232647
BnaWGS-655	BnaA10G0244800ZS	scaffoldA10	23483882	23583881	1289	0.231087
BnaWGS-655	BnaA10G0244800ZS	scaffoldA10	23493882	23593881	1326	0.218541
BnaWGS-655	BnaA10G0244800ZS	scaffoldA10	23503882	23603881	1368	0.208093
BnaWGS-655	BnaA10G0244800ZS	scaffoldA10	23513882	23613881	1161	0.192034
BnaWGS-655	BnaA10G0244800ZS	scaffoldA10	23523882	23623881	1220	0.196715
BnaWGS-655	BnaA10G0244800ZS	scaffoldA10	23533882	23633881	1326	0.197381
BnaWGS-655	BnaA10G0244800ZS	scaffoldA10	23543882	23643881	1146	0.19182
BnaWGS-655	BnaA10G0244800ZS	scaffoldA10	23553882	23653881	1143	0.196097
BnaWGS-655	BnaA10G0244800ZS	scaffoldA10	23563882	23663881	1193	0.207797
BnaWGS-655	BnaA10G0244800ZS	scaffoldA10	23573882	23673881	1432	0.202087
BnaWGS-655	BnaA10G0244800ZS	scaffoldA10	23583882	23683881	1621	0.195989
BnaWGS-655	BnaA10G0244800ZS	scaffoldA10	23593882	23693881	1556	0.202146
BnaWGS-655	BnaA10G0244800ZS	scaffoldA10	23603882	23703881	1568	0.198543
BnaWGS-655	BnaA10G0244800ZS	scaffoldA10	23613882	23713881	1681	0.197016
BnaWGS-655	BnaA10G0244800ZS	scaffoldA10	23623882	23723881	1593	0.189241
BnaWGS-655	BnaA10G0244800ZS	scaffoldA10	23633882	23733881	1557	0.193936

For Review Only

BnaWGS-655	BnaA10G0244800ZS	scaffoldA10	23643882	23743881	1566	0.201446
BnaWGS-655	BnaA10G0244800ZS	scaffoldA10	23653882	23753881	1693	0.201028
BnaWGS-655	BnaA10G0244800ZS	scaffoldA10	23663882	23763881	1712	0.192383
BnaWGS-655	BnaA10G0244800ZS	scaffoldA10	23673882	23773881	1548	0.204235
BnaWGS-655	BnaA10G0244800ZS	scaffoldA10	23683882	23783881	1467	0.215569
BnaWGS-655	BnaA10G0244800ZS	scaffoldA10	23693882	23793881	1524	0.220726
BnaWGS-655	BnaA10G0244800ZS	scaffoldA10	23703882	23803881	1550	0.222602
BnaWGS-655	BnaA10G0244800ZS	scaffoldA10	23713882	23813881	1510	0.225933
BnaWGS-655	BnaA10G0244800ZS	scaffoldA10	23723882	23823881	1516	0.228491
BnaWGS-655	BnaA10G0244800ZS	scaffoldA10	23733882	23833881	1453	0.226083
BnaWGS-655	BnaA10G0244800ZS	scaffoldA10	23743882	23843881	1423	0.218241
BnaWGS-655	BnaA10G0244800ZS	scaffoldA10	23753882	23853881	1328	0.21735
BnaWGS-655	BnaA10G0244800ZS	scaffoldA10	23763882	23863881	1360	0.235685
BnaWGS-655	BnaA10G0244800ZS	scaffoldA10	23773882	23873881	1230	0.232602
BnaWGS-655	BnaA10G0244800ZS	scaffoldA10	23783882	23883881	1158	0.240262
BnaWGS-655	BnaA10G0244800ZS	scaffoldA10	23793882	23893881	1126	0.250621
BnaWGS-655	BnaA10G0244800ZS	scaffoldA10	23803882	23903881	1005	0.259631
BnaWGS-655	BnaA10G0244800ZS	scaffoldA10	23813882	23913881	931	0.267175
BnaWGS-655	BnaA10G0244800ZS	scaffoldA10	23823882	23923881	861	0.270136
BnaWGS-655	BnaA10G0244800ZS	scaffoldA10	23833882	23933881	831	0.271133
BnaWGS-655	BnaA10G0244800ZS	scaffoldA10	23843882	23943881	823	0.280989
BnaWGS-655	BnaA10G0244800ZS	scaffoldA10	23853882	23953881	728	0.295357
BnaWGS-655	BnaA10G0244800ZS	scaffoldA10	23863882	23963881	597	0.296263
BnaWGS-655	BnaA10G0244800ZS	scaffoldA10	23873882	23973881	663	0.313759
BnaWGS-655	BnaA10G0244800ZS	scaffoldA10	23883882	23983881	787	0.28003
BnaWGS-655	BnaA10G0244800ZS	scaffoldA10	23893882	23993881	776	0.271696
BnaWGS-655	BnaA10G0244800ZS	scaffoldA10	23903882	24003881	794	0.280068
BnaWGS-655	BnaA10G0244800ZS	scaffoldA10	23913882	24013881	862	0.273961
BnaWGS-655	BnaA10G0244800ZS	scaffoldA10	23923882	24023881	993	0.283481
BnaWGS-655	BnaA10G0244800ZS	scaffoldA10	23933882	24033881	1010	0.277621
BnaWGS-655	BnaA10G0244800ZS	scaffoldA10	23943882	24043881	1121	0.258793
BnaWGS-655	BnaA10G0244800ZS	scaffoldA10	23953882	24053881	1138	0.251404
BnaWGS-655	BnaA10G0244800ZS	scaffoldA10	23963882	24063881	1234	0.246122
BnaWGS-655	BnaA10G0244800ZS	scaffoldA10	23973882	24073881	1325	0.226939
BnaWGS-655	BnaA10G0244800ZS	scaffoldA10	23983882	24083881	1322	0.221871
BnaWGS-655	BnaA10G0244800ZS	scaffoldA10	23993882	24093881	1427	0.227594
BnaWGS-655	BnaA10G0244800ZS	scaffoldA10	24003882	24103881	1572	0.205379
BnaWGS-655	BnaA10G0244800ZS	scaffoldA10	24013882	24113881	1616	0.205615
BnaWGS-655	BnaA10G0244800ZS	scaffoldA10	24023882	24123881	1711	0.192661
BnaWGS-655	BnaA10G0244800ZS	scaffoldA10	24033882	24133881	1737	0.195588
BnaWGS-655	BnaA10G0244800ZS	scaffoldA10	24043882	24143881	1725	0.209041
BnaWGS-655	BnaA10G0244800ZS	scaffoldA10	24053882	24153881	1796	0.215353
BnaWGS-655	BnaA10G0244800ZS	scaffoldA10	24063882	24163881	1814	0.210792
BnaWGS-655	BnaA10G0244800ZS	scaffoldA10	24073882	24173881	1836	0.203692
BnaWGS-655	BnaA10G0244800ZS	scaffoldA10	24083882	24183881	1993	0.211377
BnaWGS-655	BnaA10G0244800ZS	scaffoldA10	24093882	24193881	2122	0.215152
BnaWGS-655	BnaA10G0244800ZS	scaffoldA10	24103882	24203881	1993	0.230059
BnaWGS-655	BnaA10G0244800ZS	scaffoldA10	24113882	24213881	1887	0.236615
BnaWGS-655	BnaA10G0244800ZS	scaffoldA10	24123882	24223881	1926	0.239491
BnaWGS-655	BnaA10G0244800ZS	scaffoldA10	24133882	24233881	2032	0.242841
BnaWGS-655	BnaA10G0244800ZS	scaffoldA10	24143882	24243881	2093	0.241389
BnaWGS-655	BnaA10G0244800ZS	scaffoldA10	24153882	24253881	2152	0.233527
BnaWGS-655	BnaA10G0244800ZS	scaffoldA10	24163882	24263881	2159	0.24192
BnaWGS-655	BnaA10G0244800ZS	scaffoldA10	24173882	24273881	2190	0.247203
BnaWGS-655	BnaA10G0244800ZS	scaffoldA10	24183882	24283881	2032	0.24381
BnaWGS-655	BnaA10G0244800ZS	scaffoldA10	24193882	24293881	1863	0.226796
BnaWGS-655	BnaA10G0244800ZS	scaffoldA10	24203882	24303881	1936	0.226541
BnaWGS-655	BnaA10G0244800ZS	scaffoldA10	24213882	24313881	2055	0.207262
BnaWGS-655	BnaA10G0244800ZS	scaffoldA10	24223882	24323881	1956	0.202936
BnaWGS-655	BnaA10G0244800ZS	scaffoldA10	24233882	24333881	1961	0.192562

For Review Only

BnaWGS-655	BnaA10G0244800ZS	scaffoldA10	24243882	24343881	2003	0.184719
BnaWGS-655	BnaA10G0244800ZS	scaffoldA10	24253882	24353881	2142	0.185604
BnaWGS-655	BnaA10G0244800ZS	scaffoldA10	24263882	24363881	2073	0.169095
BnaWGS-655	BnaA10G0244800ZS	scaffoldA10	24273882	24373881	2050	0.182064
BnaWGS-655	BnaA10G0244800ZS	scaffoldA10	24283882	24383881	2054	0.189348
BnaWGS-655	BnaA10G0244800ZS	scaffoldA10	24293882	24393881	1969	0.194069
BnaWGS-655	BnaA10G0244800ZS	scaffoldA10	24303882	24403881	1811	0.181256
BnaWGS-655	BnaA10G0244800ZS	scaffoldA10	24313882	24413881	1845	0.197867
BnaWGS-655	BnaA10G0244800ZS	scaffoldA10	24323882	24423881	1931	0.189241
BnaWGS-655	BnaA10G0244800ZS	scaffoldA10	24333882	24433881	1929	0.185477
BnaWGS-655	BnaA10G0244800ZS	scaffoldA10	24343882	24443881	1795	0.177396
BnaWGS-655	BnaA10G0244800ZS	scaffoldA10	24353882	24453881	1546	0.177026
BnaWGS-655	BnaA10G0244800ZS	scaffoldA10	24363882	24463881	1749	0.182215
BnaWGS-655	BnaA10G0244800ZS	scaffoldA10	24373882	24473881	1713	0.170697
BnaWGS-655	BnaA10G0244800ZS	scaffoldA10	24383882	24483881	1610	0.163241
BnaWGS-655	BnaA10G0244800ZS	scaffoldA10	24393882	24493881	1706	0.165203
BnaWGS-655	BnaA10G0244800ZS	scaffoldA10	24403882	24503881	1799	0.165446
BnaWGS-655	BnaA10G0244800ZS	scaffoldA10	24413882	24513881	1675	0.156688
BnaWGS-655	BnaA10G0244800ZS	scaffoldA10	24423882	24523881	1463	0.16249
BnaWGS-655	BnaA10G0244800ZS	scaffoldA10	24433882	24533881	1338	0.163997
BnaWGS-655	BnaA10G0244800ZS	scaffoldA10	24443882	24543881	1326	0.158893
BnaWGS-655	BnaA10G0244800ZS	scaffoldA10	24453882	24553881	1472	0.1523
BnaWGS-655	BnaA10G0244800ZS	scaffoldA10	24463882	24563881	1372	0.137756
BnaWGS-655	BnaA10G0244800ZS	scaffoldA10	24473882	24573881	1397	0.143347
BnaWGS-655	BnaA10G0244800ZS	scaffoldA10	24483882	24583881	1425	0.143925
BnaWGS-655	BnaA10G0244800ZS	scaffoldA10	24493882	24593881	1317	0.1466
BnaWGS-655	BnaA10G0244800ZS	scaffoldA10	24503882	24603881	1311	0.163755
BnaWGS-655	BnaA10G0244800ZS	scaffoldA10	24513882	24613881	1361	0.164149
BnaWGS-655	BnaA10G0244800ZS	scaffoldA10	24523882	24623881	1473	0.169208
BnaWGS-655	BnaA10G0244800ZS	scaffoldA10	24533882	24633881	1514	0.172758
BnaWGS-655	BnaA10G0244800ZS	scaffoldA10	24543882	24643881	1529	0.174507
BnaWGS-655	BnaA10G0244800ZS	scaffoldA10	24553882	24653881	1509	0.18293
BnaWGS-655	BnaA10G0244800ZS	scaffoldA10	24563882	24663881	1412	0.200529
BnaWGS-655	BnaA10G0244800ZS	scaffoldA10	24573882	24673881	1274	0.193537
BnaWGS-655	BnaA10G0244800ZS	scaffoldA10	24583882	24683881	1340	0.187563
BnaWGS-655	BnaA10G0244800ZS	scaffoldA10	24593882	24693881	1438	0.176703
BnaWGS-655	BnaA10G0244800ZS	scaffoldA10	24603882	24703881	1443	0.160796
BnaWGS-655	BnaA10G0244800ZS	scaffoldA10	24613882	24713881	1383	0.157808
BnaWGS-655	BnaA10G0244800ZS	scaffoldA10	24623882	24723881	1273	0.146737
BnaWGS-655	BnaA10G0244800ZS	scaffoldA10	24633882	24733881	1302	0.138734
BnaWGS-655	BnaA10G0244800ZS	scaffoldA10	24643882	24743881	1330	0.137501
BnaWGS-655	BnaA10G0244800ZS	scaffoldA10	24653882	24753881	1341	0.132125
BnaWGS-655	BnaA10G0244800ZS	scaffoldA10	24663882	24763881	1253	0.116281
BnaWGS-655	BnaA10G0244800ZS	scaffoldA10	24673882	24773881	1181	0.115178
BnaWGS-655	BnaA10G0244800ZS	scaffoldA10	24683882	24783881	1170	0.125468
BnaWGS-655	BnaA10G0244800ZS	scaffoldA10	24693882	24793881	1246	0.126465
BnaWGS-655	BnaA10G0244800ZS	scaffoldA10	24703882	24803881	1228	0.120134
BnaWGS-655	BnaA10G0244800ZS	scaffoldA10	24713882	24813881	1307	0.118847
BnaWGS-655	BnaA10G0244800ZS	scaffoldA10	24723882	24823881	1466	0.141299
BnaWGS-655	BnaA10G0244800ZS	scaffoldA10	24733882	24833881	1448	0.154781
BnaWGS-655	BnaA10G0244800ZS	scaffoldA10	24743882	24843881	1519	0.166265
BnaWGS-655	BnaA10G0244800ZS	scaffoldA10	24753882	24853881	1428	0.169515
BnaWGS-655	BnaA10G0244800ZS	scaffoldA10	24763882	24863881	1451	0.172314
BnaWGS-655	BnaA10G0244800ZS	scaffoldA10	24773882	24873881	1607	0.180488
BnaWGS-655	BnaA10G0244800ZS	scaffoldA10	24783882	24883881	1467	0.187697
BnaWGS-655	BnaA10G0244800ZS	scaffoldA10	24793882	24893881	1285	0.199008
BnaWGS-655	BnaA10G0244800ZS	scaffoldA10	24803882	24903881	1392	0.206621
BnaWGS-655	BnaA10G0244800ZS	scaffoldA10	24813882	24913881	1477	0.21238
BnaWGS-655	BnaA10G0244800ZS	scaffoldA10	24823882	24923881	1636	0.223204
BnaWGS-655	BnaA10G0244800ZS	scaffoldA10	24833882	24933881	1790	0.21433

For Review Only

BnaWGS-655	BnaA10G0244800ZS	scaffoldA10	24843882	24943881	1654	0.216444
BnaWGS-655	BnaA10G0244800ZS	scaffoldA10	24853882	24947904	1580	0.218757
BnaWGS-655	BnaA10G0244800ZS	scaffoldA10	22943882	23043881	663	0.28633
BnaWGS-655	BnaA10G0244800ZS	scaffoldA10	22953882	23053881	624	0.285292
BnaWGS-655	BnaA10G0244800ZS	scaffoldA10	22963882	23063881	607	0.263929
BnaWGS-655	BnaA10G0244800ZS	scaffoldA10	22973882	23073881	600	0.224049
BnaWGS-655	BnaA10G0244800ZS	scaffoldA10	22983882	23083881	564	0.233639
BnaWGS-655	BnaA10G0244800ZS	scaffoldA10	22993882	23093881	591	0.069067
BnaWGS-655	BnaA10G0244800ZS	scaffoldA10	23003882	23103881	625	0.04165
BnaWGS-655	BnaA10G0244800ZS	scaffoldA10	23013882	23113881	625	0.038185
BnaWGS-655	BnaA10G0244800ZS	scaffoldA10	23023882	23123881	616	0.037716
BnaWGS-655	BnaA10G0244800ZS	scaffoldA10	23033882	23133881	718	0.071845
BnaWGS-655	BnaA10G0244800ZS	scaffoldA10	23043882	23143881	856	0.104173
BnaWGS-655	BnaA10G0244800ZS	scaffoldA10	23053882	23153881	1085	0.120464
BnaWGS-655	BnaA10G0244800ZS	scaffoldA10	23063882	23163881	1274	0.142253
BnaWGS-655	BnaA10G0244800ZS	scaffoldA10	23073882	23173881	1354	0.154679
BnaWGS-655	BnaA10G0244800ZS	scaffoldA10	23083882	23183881	1433	0.181111
BnaWGS-655	BnaA10G0244800ZS	scaffoldA10	23093882	23193881	1528	0.223403
BnaWGS-655	BnaA10G0244800ZS	scaffoldA10	23103882	23203881	1613	0.234128
BnaWGS-655	BnaA10G0244800ZS	scaffoldA10	23113882	23213881	1821	0.229762
BnaWGS-655	BnaA10G0244800ZS	scaffoldA10	23123882	23223881	1914	0.222976
BnaWGS-655	BnaA10G0244800ZS	scaffoldA10	23133882	23233881	1906	0.222608
BnaWGS-655	BnaA10G0244800ZS	scaffoldA10	23143882	23243881	1740	0.225774
BnaWGS-655	BnaA10G0244800ZS	scaffoldA10	23153882	23253881	1621	0.225205
BnaWGS-655	BnaA10G0244800ZS	scaffoldA10	23163882	23263881	1493	0.219857
BnaWGS-655	BnaA10G0244800ZS	scaffoldA10	23173882	23273881	1412	0.213518
BnaWGS-655	BnaA10G0244800ZS	scaffoldA10	23183882	23283881	1338	0.187754
BnaWGS-655	BnaA10G0244800ZS	scaffoldA10	23193882	23293881	1150	0.136539
BnaWGS-655	BnaA10G0244800ZS	scaffoldA10	23203882	23303881	1074	0.105645
BnaWGS-655	BnaA10G0244800ZS	scaffoldA10	23213882	23313881	900	0.064608
BnaWGS-655	BnaA10G0244800ZS	scaffoldA10	23223882	23323881	928	0.060578
BnaWGS-655	BnaA10G0244800ZS	scaffoldA10	23233882	23333881	897	0.144951
BnaWGS-655	BnaA10G0244800ZS	scaffoldA10	23243882	23343881	1111	0.250677
BnaWGS-655	BnaA10G0244800ZS	scaffoldA10	23253882	23353881	1092	0.284166
BnaWGS-655	BnaA10G0244800ZS	scaffoldA10	23263882	23363881	1068	0.291047
BnaWGS-655	BnaA10G0244800ZS	scaffoldA10	23273882	23373881	1060	0.321227
BnaWGS-655	BnaA10G0244800ZS	scaffoldA10	23283882	23383881	1028	0.330886
BnaWGS-655	BnaA10G0244800ZS	scaffoldA10	23293882	23393881	1068	0.340308
BnaWGS-655	BnaA10G0244800ZS	scaffoldA10	23303882	23403881	1068	0.364678
BnaWGS-655	BnaA10G0244800ZS	scaffoldA10	23313882	23413881	1032	0.369723
BnaWGS-655	BnaA10G0244800ZS	scaffoldA10	23323882	23423881	957	0.382723
BnaWGS-655	BnaA10G0244800ZS	scaffoldA10	23333882	23433881	901	0.350663
BnaWGS-655	BnaA10G0244800ZS	scaffoldA10	23343882	23443881	726	0.288754
BnaWGS-655	BnaA10G0244800ZS	scaffoldA10	23353882	23453881	661	0.273162
BnaWGS-655	BnaA10G0244800ZS	scaffoldA10	23363882	23463881	689	0.260313
BnaWGS-655	BnaA10G0244800ZS	scaffoldA10	23373882	23473881	763	0.196261
BnaWGS-655	BnaA10G0244800ZS	scaffoldA10	23383882	23483881	866	0.180061
BnaWGS-655	BnaA10G0244800ZS	scaffoldA10	23393882	23493881	880	0.151692
BnaWGS-655	BnaA10G0244800ZS	scaffoldA10	23403882	23503881	901	0.111777
BnaWGS-655	BnaA10G0244800ZS	scaffoldA10	23413882	23513881	1082	0.099273
BnaWGS-655	BnaA10G0244800ZS	scaffoldA10	23423882	23523881	1122	0.098109
BnaWGS-655	BnaA10G0244800ZS	scaffoldA10	23433882	23533881	1098	0.098357
BnaWGS-655	BnaA10G0244800ZS	scaffoldA10	23443882	23543881	1317	0.136189
BnaWGS-655	BnaA10G0244800ZS	scaffoldA10	23453882	23553881	1372	0.13849
BnaWGS-655	BnaA10G0244800ZS	scaffoldA10	23463882	23563881	1385	0.137053
BnaWGS-655	BnaA10G0244800ZS	scaffoldA10	23473882	23573881	1360	0.140742
BnaWGS-655	BnaA10G0244800ZS	scaffoldA10	23483882	23583881	1289	0.144967
BnaWGS-655	BnaA10G0244800ZS	scaffoldA10	23493882	23593881	1326	0.143763
BnaWGS-655	BnaA10G0244800ZS	scaffoldA10	23503882	23603881	1368	0.141151
BnaWGS-655	BnaA10G0244800ZS	scaffoldA10	23513882	23613881	1161	0.158654

For Review Only

BnaWGS-655	BnaA10G0244800ZS	scaffoldA10	23523882	23623881	1220	0.177136
BnaWGS-655	BnaA10G0244800ZS	scaffoldA10	23533882	23633881	1326	0.17564
BnaWGS-655	BnaA10G0244800ZS	scaffoldA10	23543882	23643881	1146	0.152901
BnaWGS-655	BnaA10G0244800ZS	scaffoldA10	23553882	23653881	1143	0.153335
BnaWGS-655	BnaA10G0244800ZS	scaffoldA10	23563882	23663881	1193	0.142221
BnaWGS-655	BnaA10G0244800ZS	scaffoldA10	23573882	23673881	1432	0.184393
BnaWGS-655	BnaA10G0244800ZS	scaffoldA10	23583882	23683881	1621	0.193018
BnaWGS-655	BnaA10G0244800ZS	scaffoldA10	23593882	23693881	1556	0.200106
BnaWGS-655	BnaA10G0244800ZS	scaffoldA10	23603882	23703881	1568	0.211261
BnaWGS-655	BnaA10G0244800ZS	scaffoldA10	23613882	23713881	1681	0.206222
BnaWGS-655	BnaA10G0244800ZS	scaffoldA10	23623882	23723881	1593	0.212363
BnaWGS-655	BnaA10G0244800ZS	scaffoldA10	23633882	23733881	1557	0.228572
BnaWGS-655	BnaA10G0244800ZS	scaffoldA10	23643882	23743881	1566	0.230835
BnaWGS-655	BnaA10G0244800ZS	scaffoldA10	23653882	23753881	1693	0.225521
BnaWGS-655	BnaA10G0244800ZS	scaffoldA10	23663882	23763881	1712	0.247361
BnaWGS-655	BnaA10G0244800ZS	scaffoldA10	23673882	23773881	1548	0.237807
BnaWGS-655	BnaA10G0244800ZS	scaffoldA10	23683882	23783881	1467	0.243395
BnaWGS-655	BnaA10G0244800ZS	scaffoldA10	23693882	23793881	1524	0.256869
BnaWGS-655	BnaA10G0244800ZS	scaffoldA10	23703882	23803881	1550	0.277678
BnaWGS-655	BnaA10G0244800ZS	scaffoldA10	23713882	23813881	1510	0.295946
BnaWGS-655	BnaA10G0244800ZS	scaffoldA10	23723882	23823881	1516	0.298704
BnaWGS-655	BnaA10G0244800ZS	scaffoldA10	23733882	23833881	1453	0.287524
BnaWGS-655	BnaA10G0244800ZS	scaffoldA10	23743882	23843881	1423	0.286874
BnaWGS-655	BnaA10G0244800ZS	scaffoldA10	23753882	23853881	1328	0.301711
BnaWGS-655	BnaA10G0244800ZS	scaffoldA10	23763882	23863881	1360	0.302028
BnaWGS-655	BnaA10G0244800ZS	scaffoldA10	23773882	23873881	1230	0.301985
BnaWGS-655	BnaA10G0244800ZS	scaffoldA10	23783882	23883881	1158	0.306638
BnaWGS-655	BnaA10G0244800ZS	scaffoldA10	23793882	23893881	1126	0.303258
BnaWGS-655	BnaA10G0244800ZS	scaffoldA10	23803882	23903881	1005	0.298611
BnaWGS-655	BnaA10G0244800ZS	scaffoldA10	23813882	23913881	931	0.298337
BnaWGS-655	BnaA10G0244800ZS	scaffoldA10	23823882	23923881	861	0.28821
BnaWGS-655	BnaA10G0244800ZS	scaffoldA10	23833882	23933881	831	0.306691
BnaWGS-655	BnaA10G0244800ZS	scaffoldA10	23843882	23943881	823	0.318338
BnaWGS-655	BnaA10G0244800ZS	scaffoldA10	23853882	23953881	728	0.3271
BnaWGS-655	BnaA10G0244800ZS	scaffoldA10	23863882	23963881	597	0.33812
BnaWGS-655	BnaA10G0244800ZS	scaffoldA10	23873882	23973881	663	0.362359
BnaWGS-655	BnaA10G0244800ZS	scaffoldA10	23883882	23983881	787	0.326042
BnaWGS-655	BnaA10G0244800ZS	scaffoldA10	23893882	23993881	776	0.326424
BnaWGS-655	BnaA10G0244800ZS	scaffoldA10	23903882	24003881	794	0.301199
BnaWGS-655	BnaA10G0244800ZS	scaffoldA10	23913882	24013881	862	0.300285
BnaWGS-655	BnaA10G0244800ZS	scaffoldA10	23923882	24023881	993	0.28979
BnaWGS-655	BnaA10G0244800ZS	scaffoldA10	23933882	24033881	1010	0.286307
BnaWGS-655	BnaA10G0244800ZS	scaffoldA10	23943882	24043881	1121	0.273301
BnaWGS-655	BnaA10G0244800ZS	scaffoldA10	23953882	24053881	1138	0.269133
BnaWGS-655	BnaA10G0244800ZS	scaffoldA10	23963882	24063881	1234	0.259144
BnaWGS-655	BnaA10G0244800ZS	scaffoldA10	23973882	24073881	1325	0.255663
BnaWGS-655	BnaA10G0244800ZS	scaffoldA10	23983882	24083881	1322	0.247456
BnaWGS-655	BnaA10G0244800ZS	scaffoldA10	23993882	24093881	1427	0.219656
BnaWGS-655	BnaA10G0244800ZS	scaffoldA10	24003882	24103881	1572	0.211178
BnaWGS-655	BnaA10G0244800ZS	scaffoldA10	24013882	24113881	1616	0.204265
BnaWGS-655	BnaA10G0244800ZS	scaffoldA10	24023882	24123881	1711	0.188911
BnaWGS-655	BnaA10G0244800ZS	scaffoldA10	24033882	24133881	1737	0.18911
BnaWGS-655	BnaA10G0244800ZS	scaffoldA10	24043882	24143881	1725	0.196085
BnaWGS-655	BnaA10G0244800ZS	scaffoldA10	24053882	24153881	1796	0.193854
BnaWGS-655	BnaA10G0244800ZS	scaffoldA10	24063882	24163881	1814	0.195604
BnaWGS-655	BnaA10G0244800ZS	scaffoldA10	24073882	24173881	1836	0.187087
BnaWGS-655	BnaA10G0244800ZS	scaffoldA10	24083882	24183881	1993	0.197518
BnaWGS-655	BnaA10G0244800ZS	scaffoldA10	24093882	24193881	2122	0.196121
BnaWGS-655	BnaA10G0244800ZS	scaffoldA10	24103882	24203881	1993	0.197676
BnaWGS-655	BnaA10G0244800ZS	scaffoldA10	24113882	24213881	1887	0.19168

For Review Only

BnaWGS-655	BnaA10G0244800ZS	scaffoldA10	24123882	24223881	1926	0.18837
BnaWGS-655	BnaA10G0244800ZS	scaffoldA10	24133882	24233881	2032	0.184004
BnaWGS-655	BnaA10G0244800ZS	scaffoldA10	24143882	24243881	2093	0.164506
BnaWGS-655	BnaA10G0244800ZS	scaffoldA10	24153882	24253881	2152	0.161217
BnaWGS-655	BnaA10G0244800ZS	scaffoldA10	24163882	24263881	2159	0.163024
BnaWGS-655	BnaA10G0244800ZS	scaffoldA10	24173882	24273881	2190	0.178146
BnaWGS-655	BnaA10G0244800ZS	scaffoldA10	24183882	24283881	2032	0.16839
BnaWGS-655	BnaA10G0244800ZS	scaffoldA10	24193882	24293881	1863	0.184095
BnaWGS-655	BnaA10G0244800ZS	scaffoldA10	24203882	24303881	1936	0.195013
BnaWGS-655	BnaA10G0244800ZS	scaffoldA10	24213882	24313881	2055	0.200175
BnaWGS-655	BnaA10G0244800ZS	scaffoldA10	24223882	24323881	1956	0.217816
BnaWGS-655	BnaA10G0244800ZS	scaffoldA10	24233882	24333881	1961	0.2193
BnaWGS-655	BnaA10G0244800ZS	scaffoldA10	24243882	24343881	2003	0.231818
BnaWGS-655	BnaA10G0244800ZS	scaffoldA10	24253882	24353881	2142	0.239246
BnaWGS-655	BnaA10G0244800ZS	scaffoldA10	24263882	24363881	2073	0.233173
BnaWGS-655	BnaA10G0244800ZS	scaffoldA10	24273882	24373881	2050	0.25314
BnaWGS-655	BnaA10G0244800ZS	scaffoldA10	24283882	24383881	2054	0.256824
BnaWGS-655	BnaA10G0244800ZS	scaffoldA10	24293882	24393881	1969	0.256357
BnaWGS-655	BnaA10G0244800ZS	scaffoldA10	24303882	24403881	1811	0.255161
BnaWGS-655	BnaA10G0244800ZS	scaffoldA10	24313882	24413881	1845	0.249957
BnaWGS-655	BnaA10G0244800ZS	scaffoldA10	24323882	24423881	1931	0.227747
BnaWGS-655	BnaA10G0244800ZS	scaffoldA10	24333882	24433881	1929	0.224559
BnaWGS-655	BnaA10G0244800ZS	scaffoldA10	24343882	24443881	1795	0.220935
BnaWGS-655	BnaA10G0244800ZS	scaffoldA10	24353882	24453881	1546	0.216986
BnaWGS-655	BnaA10G0244800ZS	scaffoldA10	24363882	24463881	1749	0.203438
BnaWGS-655	BnaA10G0244800ZS	scaffoldA10	24373882	24473881	1713	0.134896
BnaWGS-655	BnaA10G0244800ZS	scaffoldA10	24383882	24483881	1610	0.125372
BnaWGS-655	BnaA10G0244800ZS	scaffoldA10	24393882	24493881	1706	0.120941
BnaWGS-655	BnaA10G0244800ZS	scaffoldA10	24403882	24503881	1799	0.117435
BnaWGS-655	BnaA10G0244800ZS	scaffoldA10	24413882	24513881	1675	0.118923
BnaWGS-655	BnaA10G0244800ZS	scaffoldA10	24423882	24523881	1463	0.137413
BnaWGS-655	BnaA10G0244800ZS	scaffoldA10	24433882	24533881	1338	0.133797
BnaWGS-655	BnaA10G0244800ZS	scaffoldA10	24443882	24543881	1326	0.141108
BnaWGS-655	BnaA10G0244800ZS	scaffoldA10	24453882	24553881	1472	0.133505
BnaWGS-655	BnaA10G0244800ZS	scaffoldA10	24463882	24563881	1372	0.130287
BnaWGS-655	BnaA10G0244800ZS	scaffoldA10	24473882	24573881	1397	0.145557
BnaWGS-655	BnaA10G0244800ZS	scaffoldA10	24483882	24583881	1425	0.147898
BnaWGS-655	BnaA10G0244800ZS	scaffoldA10	24493882	24593881	1317	0.149852
BnaWGS-655	BnaA10G0244800ZS	scaffoldA10	24503882	24603881	1311	0.15532
BnaWGS-655	BnaA10G0244800ZS	scaffoldA10	24513882	24613881	1361	0.151991
BnaWGS-655	BnaA10G0244800ZS	scaffoldA10	24523882	24623881	1473	0.14163
BnaWGS-655	BnaA10G0244800ZS	scaffoldA10	24533882	24633881	1514	0.137259
BnaWGS-655	BnaA10G0244800ZS	scaffoldA10	24543882	24643881	1529	0.140823
BnaWGS-655	BnaA10G0244800ZS	scaffoldA10	24553882	24653881	1509	0.151302
BnaWGS-655	BnaA10G0244800ZS	scaffoldA10	24563882	24663881	1412	0.163068
BnaWGS-655	BnaA10G0244800ZS	scaffoldA10	24573882	24673881	1274	0.178137
BnaWGS-655	BnaA10G0244800ZS	scaffoldA10	24583882	24683881	1340	0.196136
BnaWGS-655	BnaA10G0244800ZS	scaffoldA10	24593882	24693881	1438	0.204206
BnaWGS-655	BnaA10G0244800ZS	scaffoldA10	24603882	24703881	1443	0.198007
BnaWGS-655	BnaA10G0244800ZS	scaffoldA10	24613882	24713881	1383	0.197848
BnaWGS-655	BnaA10G0244800ZS	scaffoldA10	24623882	24723881	1273	0.208606
BnaWGS-655	BnaA10G0244800ZS	scaffoldA10	24633882	24733881	1302	0.211655
BnaWGS-655	BnaA10G0244800ZS	scaffoldA10	24643882	24743881	1330	0.200468
BnaWGS-655	BnaA10G0244800ZS	scaffoldA10	24653882	24753881	1341	0.188083
BnaWGS-655	BnaA10G0244800ZS	scaffoldA10	24663882	24763881	1253	0.185543
BnaWGS-655	BnaA10G0244800ZS	scaffoldA10	24673882	24773881	1181	0.167335
BnaWGS-655	BnaA10G0244800ZS	scaffoldA10	24683882	24783881	1170	0.137391
BnaWGS-655	BnaA10G0244800ZS	scaffoldA10	24693882	24793881	1246	0.115079
BnaWGS-655	BnaA10G0244800ZS	scaffoldA10	24703882	24803881	1228	0.113237
BnaWGS-655	BnaA10G0244800ZS	scaffoldA10	24713882	24813881	1307	0.107314

For Review Only

BnaWGS-655	BnaA10G0244800ZS	scaffoldA10	24723882	24823881	1466	0.112092
BnaWGS-655	BnaA10G0244800ZS	scaffoldA10	24733882	24833881	1448	0.111944
BnaWGS-655	BnaA10G0244800ZS	scaffoldA10	24743882	24843881	1519	0.101201
BnaWGS-655	BnaA10G0244800ZS	scaffoldA10	24753882	24853881	1428	0.10087
BnaWGS-655	BnaA10G0244800ZS	scaffoldA10	24763882	24863881	1451	0.101046
BnaWGS-655	BnaA10G0244800ZS	scaffoldA10	24773882	24873881	1607	0.101263
BnaWGS-655	BnaA10G0244800ZS	scaffoldA10	24783882	24883881	1467	0.10719
BnaWGS-655	BnaA10G0244800ZS	scaffoldA10	24793882	24893881	1285	0.110616
BnaWGS-655	BnaA10G0244800ZS	scaffoldA10	24803882	24903881	1392	0.110766
BnaWGS-655	BnaA10G0244800ZS	scaffoldA10	24813882	24913881	1477	0.123107
BnaWGS-655	BnaA10G0244800ZS	scaffoldA10	24823882	24923881	1636	0.109201
BnaWGS-655	BnaA10G0244800ZS	scaffoldA10	24833882	24933881	1790	0.115023
BnaWGS-655	BnaA10G0244800ZS	scaffoldA10	24843882	24943881	1654	0.131484
BnaWGS-655	BnaA10G0244800ZS	scaffoldA10	24853882	24947904	1580	0.135229
BnaWGS-655	BnaA10G0244800ZS	scaffoldA10	22943882	23043881	663	0.208375
BnaWGS-655	BnaA10G0244800ZS	scaffoldA10	22953882	23053881	624	0.211268
BnaWGS-655	BnaA10G0244800ZS	scaffoldA10	22963882	23063881	607	0.204439
BnaWGS-655	BnaA10G0244800ZS	scaffoldA10	22973882	23073881	600	0.187966
BnaWGS-655	BnaA10G0244800ZS	scaffoldA10	22983882	23083881	564	0.194122
BnaWGS-655	BnaA10G0244800ZS	scaffoldA10	22993882	23093881	591	0.208009
BnaWGS-655	BnaA10G0244800ZS	scaffoldA10	23003882	23103881	625	0.290481
BnaWGS-655	BnaA10G0244800ZS	scaffoldA10	23013882	23113881	625	0.328548
BnaWGS-655	BnaA10G0244800ZS	scaffoldA10	23023882	23123881	616	0.336693
BnaWGS-655	BnaA10G0244800ZS	scaffoldA10	23033882	23133881	718	0.363477
BnaWGS-655	BnaA10G0244800ZS	scaffoldA10	23043882	23143881	856	0.370118
BnaWGS-655	BnaA10G0244800ZS	scaffoldA10	23053882	23153881	1085	0.349032
BnaWGS-655	BnaA10G0244800ZS	scaffoldA10	23063882	23163881	1274	0.334232
BnaWGS-655	BnaA10G0244800ZS	scaffoldA10	23073882	23173881	1354	0.328267
BnaWGS-655	BnaA10G0244800ZS	scaffoldA10	23083882	23183881	1433	0.338907
BnaWGS-655	BnaA10G0244800ZS	scaffoldA10	23093882	23193881	1528	0.33763
BnaWGS-655	BnaA10G0244800ZS	scaffoldA10	23103882	23203881	1613	0.32011
BnaWGS-655	BnaA10G0244800ZS	scaffoldA10	23113882	23213881	1821	0.326754
BnaWGS-655	BnaA10G0244800ZS	scaffoldA10	23123882	23223881	1914	0.33656
BnaWGS-655	BnaA10G0244800ZS	scaffoldA10	23133882	23233881	1906	0.328496
BnaWGS-655	BnaA10G0244800ZS	scaffoldA10	23143882	23243881	1740	0.32263
BnaWGS-655	BnaA10G0244800ZS	scaffoldA10	23153882	23253881	1621	0.325393
BnaWGS-655	BnaA10G0244800ZS	scaffoldA10	23163882	23263881	1493	0.328082
BnaWGS-655	BnaA10G0244800ZS	scaffoldA10	23173882	23273881	1412	0.334667
BnaWGS-655	BnaA10G0244800ZS	scaffoldA10	23183882	23283881	1338	0.315822
BnaWGS-655	BnaA10G0244800ZS	scaffoldA10	23193882	23293881	1150	0.301562
BnaWGS-655	BnaA10G0244800ZS	scaffoldA10	23203882	23303881	1074	0.289868
BnaWGS-655	BnaA10G0244800ZS	scaffoldA10	23213882	23313881	900	0.194724
BnaWGS-655	BnaA10G0244800ZS	scaffoldA10	23223882	23323881	928	0.077117
BnaWGS-655	BnaA10G0244800ZS	scaffoldA10	23233882	23333881	897	0.131895
BnaWGS-655	BnaA10G0244800ZS	scaffoldA10	23243882	23343881	1111	0.238049
BnaWGS-655	BnaA10G0244800ZS	scaffoldA10	23253882	23353881	1092	0.268468
BnaWGS-655	BnaA10G0244800ZS	scaffoldA10	23263882	23363881	1068	0.275134
BnaWGS-655	BnaA10G0244800ZS	scaffoldA10	23273882	23373881	1060	0.287394
BnaWGS-655	BnaA10G0244800ZS	scaffoldA10	23283882	23383881	1028	0.296314
BnaWGS-655	BnaA10G0244800ZS	scaffoldA10	23293882	23393881	1068	0.309132
BnaWGS-655	BnaA10G0244800ZS	scaffoldA10	23303882	23403881	1068	0.327528
BnaWGS-655	BnaA10G0244800ZS	scaffoldA10	23313882	23413881	1032	0.34487
BnaWGS-655	BnaA10G0244800ZS	scaffoldA10	23323882	23423881	957	0.362285
BnaWGS-655	BnaA10G0244800ZS	scaffoldA10	23333882	23433881	901	0.347899
BnaWGS-655	BnaA10G0244800ZS	scaffoldA10	23343882	23443881	726	0.298763
BnaWGS-655	BnaA10G0244800ZS	scaffoldA10	23353882	23453881	661	0.269702
BnaWGS-655	BnaA10G0244800ZS	scaffoldA10	23363882	23463881	689	0.277934
BnaWGS-655	BnaA10G0244800ZS	scaffoldA10	23373882	23473881	763	0.268481
BnaWGS-655	BnaA10G0244800ZS	scaffoldA10	23383882	23483881	866	0.274582
BnaWGS-655	BnaA10G0244800ZS	scaffoldA10	23393882	23493881	880	0.29074

For Review Only

BnaWGS-655	BnaA10G0244800ZS	scaffoldA10	23403882	23503881	901	0.342476
BnaWGS-655	BnaA10G0244800ZS	scaffoldA10	23413882	23513881	1082	0.36758
BnaWGS-655	BnaA10G0244800ZS	scaffoldA10	23423882	23523881	1122	0.368408
BnaWGS-655	BnaA10G0244800ZS	scaffoldA10	23433882	23533881	1098	0.375668
BnaWGS-655	BnaA10G0244800ZS	scaffoldA10	23443882	23543881	1317	0.367455
BnaWGS-655	BnaA10G0244800ZS	scaffoldA10	23453882	23553881	1372	0.37114
BnaWGS-655	BnaA10G0244800ZS	scaffoldA10	23463882	23563881	1385	0.375858
BnaWGS-655	BnaA10G0244800ZS	scaffoldA10	23473882	23573881	1360	0.384503
BnaWGS-655	BnaA10G0244800ZS	scaffoldA10	23483882	23583881	1289	0.38735
BnaWGS-655	BnaA10G0244800ZS	scaffoldA10	23493882	23593881	1326	0.376539
BnaWGS-655	BnaA10G0244800ZS	scaffoldA10	23503882	23603881	1368	0.363226
BnaWGS-655	BnaA10G0244800ZS	scaffoldA10	23513882	23613881	1161	0.347642
BnaWGS-655	BnaA10G0244800ZS	scaffoldA10	23523882	23623881	1220	0.316928
BnaWGS-655	BnaA10G0244800ZS	scaffoldA10	23533882	23633881	1326	0.3197
BnaWGS-655	BnaA10G0244800ZS	scaffoldA10	23543882	23643881	1146	0.319414
BnaWGS-655	BnaA10G0244800ZS	scaffoldA10	23553882	23653881	1143	0.311008
BnaWGS-655	BnaA10G0244800ZS	scaffoldA10	23563882	23663881	1193	0.290885
BnaWGS-655	BnaA10G0244800ZS	scaffoldA10	23573882	23673881	1432	0.272899
BnaWGS-655	BnaA10G0244800ZS	scaffoldA10	23583882	23683881	1621	0.26329
BnaWGS-655	BnaA10G0244800ZS	scaffoldA10	23593882	23693881	1556	0.255144
BnaWGS-655	BnaA10G0244800ZS	scaffoldA10	23603882	23703881	1568	0.210338
BnaWGS-655	BnaA10G0244800ZS	scaffoldA10	23613882	23713881	1681	0.217976
BnaWGS-655	BnaA10G0244800ZS	scaffoldA10	23623882	23723881	1593	0.241202
BnaWGS-655	BnaA10G0244800ZS	scaffoldA10	23633882	23733881	1557	0.228891
BnaWGS-655	BnaA10G0244800ZS	scaffoldA10	23643882	23743881	1566	0.218881
BnaWGS-655	BnaA10G0244800ZS	scaffoldA10	23653882	23753881	1693	0.228732
BnaWGS-655	BnaA10G0244800ZS	scaffoldA10	23663882	23763881	1712	0.235841
BnaWGS-655	BnaA10G0244800ZS	scaffoldA10	23673882	23773881	1548	0.257473
BnaWGS-655	BnaA10G0244800ZS	scaffoldA10	23683882	23783881	1467	0.258928
BnaWGS-655	BnaA10G0244800ZS	scaffoldA10	23693882	23793881	1524	0.284582
BnaWGS-655	BnaA10G0244800ZS	scaffoldA10	23703882	23803881	1550	0.341746
BnaWGS-655	BnaA10G0244800ZS	scaffoldA10	23713882	23813881	1510	0.354203
BnaWGS-655	BnaA10G0244800ZS	scaffoldA10	23723882	23823881	1516	0.356086
BnaWGS-655	BnaA10G0244800ZS	scaffoldA10	23733882	23833881	1453	0.369638
BnaWGS-655	BnaA10G0244800ZS	scaffoldA10	23743882	23843881	1423	0.386527
BnaWGS-655	BnaA10G0244800ZS	scaffoldA10	23753882	23853881	1328	0.399775
BnaWGS-655	BnaA10G0244800ZS	scaffoldA10	23763882	23863881	1360	0.408594
BnaWGS-655	BnaA10G0244800ZS	scaffoldA10	23773882	23873881	1230	0.394258
BnaWGS-655	BnaA10G0244800ZS	scaffoldA10	23783882	23883881	1158	0.399333
BnaWGS-655	BnaA10G0244800ZS	scaffoldA10	23793882	23893881	1126	0.365275
BnaWGS-655	BnaA10G0244800ZS	scaffoldA10	23803882	23903881	1005	0.339683
BnaWGS-655	BnaA10G0244800ZS	scaffoldA10	23813882	23913881	931	0.323991
BnaWGS-655	BnaA10G0244800ZS	scaffoldA10	23823882	23923881	861	0.300681
BnaWGS-655	BnaA10G0244800ZS	scaffoldA10	23833882	23933881	831	0.272217
BnaWGS-655	BnaA10G0244800ZS	scaffoldA10	23843882	23943881	823	0.244398
BnaWGS-655	BnaA10G0244800ZS	scaffoldA10	23853882	23953881	728	0.221978
BnaWGS-655	BnaA10G0244800ZS	scaffoldA10	23863882	23963881	597	0.146118
BnaWGS-655	BnaA10G0244800ZS	scaffoldA10	23873882	23973881	663	0.132649
BnaWGS-655	BnaA10G0244800ZS	scaffoldA10	23883882	23983881	787	0.120364
BnaWGS-655	BnaA10G0244800ZS	scaffoldA10	23893882	23993881	776	0.129186
BnaWGS-655	BnaA10G0244800ZS	scaffoldA10	23903882	24003881	794	0.130431
BnaWGS-655	BnaA10G0244800ZS	scaffoldA10	23913882	24013881	862	0.178234
BnaWGS-655	BnaA10G0244800ZS	scaffoldA10	23923882	24023881	993	0.206484
BnaWGS-655	BnaA10G0244800ZS	scaffoldA10	23933882	24033881	1010	0.219378
BnaWGS-655	BnaA10G0244800ZS	scaffoldA10	23943882	24043881	1121	0.243071
BnaWGS-655	BnaA10G0244800ZS	scaffoldA10	23953882	24053881	1138	0.259482
BnaWGS-655	BnaA10G0244800ZS	scaffoldA10	23963882	24063881	1234	0.274817
BnaWGS-655	BnaA10G0244800ZS	scaffoldA10	23973882	24073881	1325	0.320368
BnaWGS-655	BnaA10G0244800ZS	scaffoldA10	23983882	24083881	1322	0.350056
BnaWGS-655	BnaA10G0244800ZS	scaffoldA10	23993882	24093881	1427	0.362922

For Review Only

BnaWGS-655	BnaA10G0244800ZS	scaffoldA10	24003882	24103881	1572	0.351505
BnaWGS-655	BnaA10G0244800ZS	scaffoldA10	24013882	24113881	1616	0.34373
BnaWGS-655	BnaA10G0244800ZS	scaffoldA10	24023882	24123881	1711	0.337064
BnaWGS-655	BnaA10G0244800ZS	scaffoldA10	24033882	24133881	1737	0.330748
BnaWGS-655	BnaA10G0244800ZS	scaffoldA10	24043882	24143881	1725	0.319774
BnaWGS-655	BnaA10G0244800ZS	scaffoldA10	24053882	24153881	1796	0.3143
BnaWGS-655	BnaA10G0244800ZS	scaffoldA10	24063882	24163881	1814	0.322435
BnaWGS-655	BnaA10G0244800ZS	scaffoldA10	24073882	24173881	1836	0.325742
BnaWGS-655	BnaA10G0244800ZS	scaffoldA10	24083882	24183881	1993	0.338931
BnaWGS-655	BnaA10G0244800ZS	scaffoldA10	24093882	24193881	2122	0.328712
BnaWGS-655	BnaA10G0244800ZS	scaffoldA10	24103882	24203881	1993	0.328705
BnaWGS-655	BnaA10G0244800ZS	scaffoldA10	24113882	24213881	1887	0.326416
BnaWGS-655	BnaA10G0244800ZS	scaffoldA10	24123882	24223881	1926	0.322533
BnaWGS-655	BnaA10G0244800ZS	scaffoldA10	24133882	24233881	2032	0.327646
BnaWGS-655	BnaA10G0244800ZS	scaffoldA10	24143882	24243881	2093	0.327027
BnaWGS-655	BnaA10G0244800ZS	scaffoldA10	24153882	24253881	2152	0.318954
BnaWGS-655	BnaA10G0244800ZS	scaffoldA10	24163882	24263881	2159	0.309243
BnaWGS-655	BnaA10G0244800ZS	scaffoldA10	24173882	24273881	2190	0.31097
BnaWGS-655	BnaA10G0244800ZS	scaffoldA10	24183882	24283881	2032	0.293073
BnaWGS-655	BnaA10G0244800ZS	scaffoldA10	24193882	24293881	1863	0.288796
BnaWGS-655	BnaA10G0244800ZS	scaffoldA10	24203882	24303881	1936	0.291162
BnaWGS-655	BnaA10G0244800ZS	scaffoldA10	24213882	24313881	2055	0.290855
BnaWGS-655	BnaA10G0244800ZS	scaffoldA10	24223882	24323881	1956	0.29521
BnaWGS-655	BnaA10G0244800ZS	scaffoldA10	24233882	24333881	1961	0.28434
BnaWGS-655	BnaA10G0244800ZS	scaffoldA10	24243882	24343881	2003	0.287279
BnaWGS-655	BnaA10G0244800ZS	scaffoldA10	24253882	24353881	2142	0.278033
BnaWGS-655	BnaA10G0244800ZS	scaffoldA10	24263882	24363881	2073	0.27533
BnaWGS-655	BnaA10G0244800ZS	scaffoldA10	24273882	24373881	2050	0.243817
BnaWGS-655	BnaA10G0244800ZS	scaffoldA10	24283882	24383881	2054	0.248374
BnaWGS-655	BnaA10G0244800ZS	scaffoldA10	24293882	24393881	1969	0.258937
BnaWGS-655	BnaA10G0244800ZS	scaffoldA10	24303882	24403881	1811	0.258544
BnaWGS-655	BnaA10G0244800ZS	scaffoldA10	24313882	24413881	1845	0.254345
BnaWGS-655	BnaA10G0244800ZS	scaffoldA10	24323882	24423881	1931	0.248095
BnaWGS-655	BnaA10G0244800ZS	scaffoldA10	24333882	24433881	1929	0.244062
BnaWGS-655	BnaA10G0244800ZS	scaffoldA10	24343882	24443881	1795	0.238572
BnaWGS-655	BnaA10G0244800ZS	scaffoldA10	24353882	24453881	1546	0.245167
BnaWGS-655	BnaA10G0244800ZS	scaffoldA10	24363882	24463881	1749	0.254404
BnaWGS-655	BnaA10G0244800ZS	scaffoldA10	24373882	24473881	1713	0.25687
BnaWGS-655	BnaA10G0244800ZS	scaffoldA10	24383882	24483881	1610	0.254528
BnaWGS-655	BnaA10G0244800ZS	scaffoldA10	24393882	24493881	1706	0.242822
BnaWGS-655	BnaA10G0244800ZS	scaffoldA10	24403882	24503881	1799	0.23791
BnaWGS-655	BnaA10G0244800ZS	scaffoldA10	24413882	24513881	1675	0.237385
BnaWGS-655	BnaA10G0244800ZS	scaffoldA10	24423882	24523881	1463	0.241256
BnaWGS-655	BnaA10G0244800ZS	scaffoldA10	24433882	24533881	1338	0.243211
BnaWGS-655	BnaA10G0244800ZS	scaffoldA10	24443882	24543881	1326	0.253214
BnaWGS-655	BnaA10G0244800ZS	scaffoldA10	24453882	24553881	1472	0.243819
BnaWGS-655	BnaA10G0244800ZS	scaffoldA10	24463882	24563881	1372	0.205478
BnaWGS-655	BnaA10G0244800ZS	scaffoldA10	24473882	24573881	1397	0.177192
BnaWGS-655	BnaA10G0244800ZS	scaffoldA10	24483882	24583881	1425	0.169463
BnaWGS-655	BnaA10G0244800ZS	scaffoldA10	24493882	24593881	1317	0.154351
BnaWGS-655	BnaA10G0244800ZS	scaffoldA10	24503882	24603881	1311	0.140696
BnaWGS-655	BnaA10G0244800ZS	scaffoldA10	24513882	24613881	1361	0.1458
BnaWGS-655	BnaA10G0244800ZS	scaffoldA10	24523882	24623881	1473	0.168442
BnaWGS-655	BnaA10G0244800ZS	scaffoldA10	24533882	24633881	1514	0.183431
BnaWGS-655	BnaA10G0244800ZS	scaffoldA10	24543882	24643881	1529	0.19408
BnaWGS-655	BnaA10G0244800ZS	scaffoldA10	24553882	24653881	1509	0.225656
BnaWGS-655	BnaA10G0244800ZS	scaffoldA10	24563882	24663881	1412	0.252638
BnaWGS-655	BnaA10G0244800ZS	scaffoldA10	24573882	24673881	1274	0.303641
BnaWGS-655	BnaA10G0244800ZS	scaffoldA10	24583882	24683881	1340	0.335464
BnaWGS-655	BnaA10G0244800ZS	scaffoldA10	24593882	24693881	1438	0.359158

For Review Only

BnaWGS-655	BnaA10G0244800ZS	scaffoldA10	24603882	24703881	1443	0.362984
BnaWGS-655	BnaA10G0244800ZS	scaffoldA10	24613882	24713881	1383	0.375097
BnaWGS-655	BnaA10G0244800ZS	scaffoldA10	24623882	24723881	1273	0.381822
BnaWGS-655	BnaA10G0244800ZS	scaffoldA10	24633882	24733881	1302	0.386956
BnaWGS-655	BnaA10G0244800ZS	scaffoldA10	24643882	24743881	1330	0.389218
BnaWGS-655	BnaA10G0244800ZS	scaffoldA10	24653882	24753881	1341	0.374107
BnaWGS-655	BnaA10G0244800ZS	scaffoldA10	24663882	24763881	1253	0.378024
BnaWGS-655	BnaA10G0244800ZS	scaffoldA10	24673882	24773881	1181	0.363879
BnaWGS-655	BnaA10G0244800ZS	scaffoldA10	24683882	24783881	1170	0.322546
BnaWGS-655	BnaA10G0244800ZS	scaffoldA10	24693882	24793881	1246	0.274179
BnaWGS-655	BnaA10G0244800ZS	scaffoldA10	24703882	24803881	1228	0.279214
BnaWGS-655	BnaA10G0244800ZS	scaffoldA10	24713882	24813881	1307	0.249989
BnaWGS-655	BnaA10G0244800ZS	scaffoldA10	24723882	24823881	1466	0.233808
BnaWGS-655	BnaA10G0244800ZS	scaffoldA10	24733882	24833881	1448	0.211327
BnaWGS-655	BnaA10G0244800ZS	scaffoldA10	24743882	24843881	1519	0.165389
BnaWGS-655	BnaA10G0244800ZS	scaffoldA10	24753882	24853881	1428	0.140341
BnaWGS-655	BnaA10G0244800ZS	scaffoldA10	24763882	24863881	1451	0.142331
BnaWGS-655	BnaA10G0244800ZS	scaffoldA10	24773882	24873881	1607	0.143643
BnaWGS-655	BnaA10G0244800ZS	scaffoldA10	24783882	24883881	1467	0.149548
BnaWGS-655	BnaA10G0244800ZS	scaffoldA10	24793882	24893881	1285	0.159136
BnaWGS-655	BnaA10G0244800ZS	scaffoldA10	24803882	24903881	1392	0.162478
BnaWGS-655	BnaA10G0244800ZS	scaffoldA10	24813882	24913881	1477	0.155029
BnaWGS-655	BnaA10G0244800ZS	scaffoldA10	24823882	24923881	1636	0.217566
BnaWGS-655	BnaA10G0244800ZS	scaffoldA10	24833882	24933881	1790	0.241988
BnaWGS-655	BnaA10G0244800ZS	scaffoldA10	24843882	24943881	1654	0.269418
BnaWGS-655	BnaA10G0244800ZS	scaffoldA10	24853882	24947904	1580	0.275223

For Review Only

s surrounding gene

groups
Winter_Spring
Winter_Spring
Winter_Spring
Winter_Spring
Winter_Spring
Winter_Spring
Winter_Spring
Winter_Spring
Winter_Spring
Winter_Spring
Winter_Spring
Winter_Spring
Winter_Spring
Winter_Spring
Winter_Spring
Winter_Spring
Winter_Spring
Winter_Spring
Winter_Spring
Winter_Spring
Winter_Spring
Winter_Spring
Winter_Spring
Winter_Spring
Winter_Spring
Winter_Spring
Winter_Spring
Winter_Spring
Winter_Spring
Winter_Spring
Winter_Spring
Winter_Spring
Winter_Spring
Winter_Spring
Winter_Spring
Winter_Spring
Winter_Spring
Winter_Spring
Winter_Spring
Winter_Spring
Winter_Spring
Winter_Spring
Winter_Spring
Winter_Spring
Winter_Spring
Winter_Spring
Winter_Spring
Winter_Spring
Winter_Spring
Winter_Spring
Winter_Spring
Winter_Spring
Winter_Spring
Winter_Spring
Winter_Spring
Winter_Spring
Winter_Spring
Winter_Spring

For Review Only

Winter_Spring
Winter_Spring
Winter_Spring
Winter_Spring
Winter_Spring
Winter_Spring
Winter_Spring
Winter_Spring
Winter_Spring
Winter_Spring
Winter_Spring
Winter_Spring
Winter_Spring
Winter_Spring
Winter_Spring
Winter_Spring
Winter_Spring
Winter_Spring
Winter_Spring
Winter_Spring
Winter_Spring
Winter_Spring
Winter_Spring
Winter_Spring
Winter_Spring
Winter_Spring
Winter_Spring
Winter_Spring
Winter_Spring
Winter_Spring
Winter_Spring
Winter_Spring
Winter_Spring
Winter_Spring
Winter_Spring
Winter_Spring
Winter_Spring
Winter_Spring
Winter_Spring
Winter_Spring
Winter_Spring
Winter_Spring
Winter_Spring
Winter_Spring
Winter_Spring
Winter_Spring
Winter_Spring
Winter_Spring
Winter_Spring
Winter_Spring
Winter_Spring
Winter_Spring
Winter_Spring
Winter_Spring
Winter_Spring
Winter_Spring
Winter_Spring
Winter_Spring
Winter_Spring
Winter_Spring

For Review Only

Winter_Spring
Winter_Spring
Winter_Spring
Winter_Spring
Winter_Spring
Winter_Spring
Winter_Spring
Winter_Spring
Winter_Spring
Winter_Spring
Winter_Spring
Winter_Spring
Winter_Spring
Winter_Spring
Winter_Spring
Winter_Spring
Winter_Spring
Winter_Spring
Winter_Spring
Winter_Spring
Winter_Spring
Winter_Spring
Winter_Spring
Winter_Spring
Winter_Spring
Winter_Spring
Winter_Spring
Winter_Spring
Winter_Spring
Winter_Spring
Winter_Spring
Winter_Spring
Winter_Spring
Winter_Spring
Winter_Spring
Winter_Spring
Winter_Spring
Winter_Spring
Winter_Spring
Winter_Spring
Winter_Spring
Winter_Spring
Winter_Spring
Winter_Spring
Winter_Spring
Winter_Spring
Winter_Spring
Winter_Spring
Winter_Spring
Winter_Spring
Winter_Spring
Winter_Spring
Winter_Spring
Winter_Spring
Winter_Spring
Winter_Spring
Winter_Spring
Winter_Spring
Winter_Spring
Winter_Spring

For Review Only

Winter_Spring
Winter_Spring
Winter_Spring
Winter_Spring
Winter_Spring
Winter_Spring
Winter_Spring
Winter_Spring
Winter_Spring
Winter_Spring
Winter_Spring
Winter_Spring
Winter_Spring
Winter_Spring
Winter_Semi-winter
Winter_Semi-winter
Winter_Semi-winter
Winter_Semi-winter
Winter_Semi-winter
Winter_Semi-winter
Winter_Semi-winter
Winter_Semi-winter
Winter_Semi-winter
Winter_Semi-winter
Winter_Semi-winter
Winter_Semi-winter
Winter_Semi-winter
Winter_Semi-winter
Winter_Semi-winter
Winter_Semi-winter
Winter_Semi-winter
Winter_Semi-winter
Winter_Semi-winter
Winter_Semi-winter
Winter_Semi-winter
Winter_Semi-winter
Winter_Semi-winter
Winter_Semi-winter
Winter_Semi-winter
Winter_Semi-winter
Winter_Semi-winter
Winter_Semi-winter
Winter_Semi-winter
Winter_Semi-winter
Winter_Semi-winter
Winter_Semi-winter
Winter_Semi-winter
Winter_Semi-winter
Winter_Semi-winter
Winter_Semi-winter
Winter_Semi-winter
Winter_Semi-winter
Winter_Semi-winter
Winter_Semi-winter
Winter_Semi-winter
Winter_Semi-winter
Winter_Semi-winter
Winter_Semi-winter
Winter_Semi-winter
Winter_Semi-winter

For Review Only

Winter_Semi-winter
Winter_Semi-winter
Winter_Semi-winter
Winter_Semi-winter
Winter_Semi-winter
Winter_Semi-winter
Winter_Semi-winter
Winter_Semi-winter
Winter_Semi-winter
Winter_Semi-winter
Winter_Semi-winter
Winter_Semi-winter
Winter_Semi-winter
Winter_Semi-winter
Winter_Semi-winter
Winter_Semi-winter
Winter_Semi-winter
Winter_Semi-winter
Winter_Semi-winter
Winter_Semi-winter
Winter_Semi-winter
Winter_Semi-winter
Winter_Semi-winter
Winter_Semi-winter
Winter_Semi-winter
Winter_Semi-winter
Winter_Semi-winter
Winter_Semi-winter
Winter_Semi-winter
Winter_Semi-winter
Winter_Semi-winter
Winter_Semi-winter
Winter_Semi-winter
Winter_Semi-winter
Winter_Semi-winter
Winter_Semi-winter
Winter_Semi-winter
Winter_Semi-winter
Winter_Semi-winter
Winter_Semi-winter
Winter_Semi-winter
Winter_Semi-winter
Winter_Semi-winter
Winter_Semi-winter
Winter_Semi-winter
Winter_Semi-winter
Winter_Semi-winter
Winter_Semi-winter
Winter_Semi-winter
Winter_Semi-winter
Winter_Semi-winter
Winter_Semi-winter
Winter_Semi-winter
Winter_Semi-winter
Winter_Semi-winter
Winter_Semi-winter
Winter_Semi-winter
Winter_Semi-winter
Winter_Semi-winter
Winter_Semi-winter

For Review Only

Winter_Semi-winter
Winter_Semi-winter
Winter_Semi-winter
Winter_Semi-winter
Winter_Semi-winter
Winter_Semi-winter
Winter_Semi-winter
Winter_Semi-winter
Winter_Semi-winter
Winter_Semi-winter
Winter_Semi-winter
Winter_Semi-winter
Winter_Semi-winter
Winter_Semi-winter
Winter_Semi-winter
Winter_Semi-winter
Winter_Semi-winter
Winter_Semi-winter
Winter_Semi-winter
Winter_Semi-winter
Winter_Semi-winter
Winter_Semi-winter
Winter_Semi-winter
Winter_Semi-winter
Winter_Semi-winter
Winter_Semi-winter
Winter_Semi-winter
Winter_Semi-winter
Winter_Semi-winter
Winter_Semi-winter
Winter_Semi-winter
Winter_Semi-winter
Winter_Semi-winter
Winter_Semi-winter
Winter_Semi-winter
Winter_Semi-winter
Winter_Semi-winter
Winter_Semi-winter
Winter_Semi-winter
Winter_Semi-winter
Winter_Semi-winter
Winter_Semi-winter
Winter_Semi-winter
Winter_Semi-winter
Winter_Semi-winter
Winter_Semi-winter
Winter_Semi-winter
Winter_Semi-winter
Winter_Semi-winter
Winter_Semi-winter
Winter_Semi-winter
Winter_Semi-winter
Winter_Semi-winter
Winter_Semi-winter
Winter_Semi-winter
Winter_Semi-winter
Winter_Semi-winter
Winter_Semi-winter
Winter_Semi-winter
Winter_Semi-winter

For Review Only

Winter_Semi-winter
Winter_Semi-winter
Winter_Semi-winter
Winter_Semi-winter
Winter_Semi-winter
Winter_Semi-winter
Winter_Semi-winter
Winter_Semi-winter
Winter_Semi-winter
Winter_Semi-winter
Winter_Semi-winter
Winter_Semi-winter
Winter_Semi-winter
Winter_Semi-winter
Winter_Semi-winter
Winter_Semi-winter
Winter_Semi-winter
Winter_Semi-winter
Winter_Semi-winter
Winter_Semi-winter
Winter_Semi-winter
Winter_Semi-winter
Winter_Semi-winter
Winter_Semi-winter
Winter_Semi-winter
Winter_Semi-winter
Spring_Semi-winter
Spring_Semi-winter
Spring_Semi-winter
Spring_Semi-winter
Spring_Semi-winter
Spring_Semi-winter
Spring_Semi-winter
Spring_Semi-winter
Spring_Semi-winter
Spring_Semi-winter
Spring_Semi-winter
Spring_Semi-winter
Spring_Semi-winter
Spring_Semi-winter
Spring_Semi-winter
Spring_Semi-winter
Spring_Semi-winter
Spring_Semi-winter
Spring_Semi-winter
Spring_Semi-winter
Spring_Semi-winter
Spring_Semi-winter
Spring_Semi-winter
Spring_Semi-winter
Spring_Semi-winter
Spring_Semi-winter
Spring_Semi-winter
Spring_Semi-winter
Spring_Semi-winter
Spring_Semi-winter
Spring_Semi-winter
Spring_Semi-winter
Spring_Semi-winter
Spring_Semi-winter

For Review Only

Spring_Semi-winter
Spring_Semi-winter
Spring_Semi-winter
Spring_Semi-winter
Spring_Semi-winter
Spring_Semi-winter
Spring_Semi-winter
Spring_Semi-winter
Spring_Semi-winter
Spring_Semi-winter
Spring_Semi-winter
Spring_Semi-winter
Spring_Semi-winter
Spring_Semi-winter
Spring_Semi-winter
Spring_Semi-winter
Spring_Semi-winter
Spring_Semi-winter
Spring_Semi-winter
Spring_Semi-winter
Spring_Semi-winter
Spring_Semi-winter
Spring_Semi-winter
Spring_Semi-winter
Spring_Semi-winter
Spring_Semi-winter
Spring_Semi-winter
Spring_Semi-winter
Spring_Semi-winter
Spring_Semi-winter
Spring_Semi-winter
Spring_Semi-winter
Spring_Semi-winter
Spring_Semi-winter
Spring_Semi-winter
Spring_Semi-winter
Spring_Semi-winter
Spring_Semi-winter
Spring_Semi-winter
Spring_Semi-winter
Spring_Semi-winter
Spring_Semi-winter
Spring_Semi-winter
Spring_Semi-winter
Spring_Semi-winter
Spring_Semi-winter
Spring_Semi-winter
Spring_Semi-winter
Spring_Semi-winter
Spring_Semi-winter
Spring_Semi-winter
Spring_Semi-winter
Spring_Semi-winter
Spring_Semi-winter
Spring_Semi-winter
Spring_Semi-winter
Spring_Semi-winter
Spring_Semi-winter
Spring_Semi-winter
Spring_Semi-winter

For Review Only

Spring_Semi-winter
Spring_Semi-winter
Spring_Semi-winter
Spring_Semi-winter
Spring_Semi-winter
Spring_Semi-winter
Spring_Semi-winter
Spring_Semi-winter
Spring_Semi-winter
Spring_Semi-winter
Spring_Semi-winter
Spring_Semi-winter
Spring_Semi-winter
Spring_Semi-winter
Spring_Semi-winter
Spring_Semi-winter
Spring_Semi-winter
Spring_Semi-winter
Spring_Semi-winter
Spring_Semi-winter
Spring_Semi-winter
Spring_Semi-winter
Spring_Semi-winter
Spring_Semi-winter
Spring_Semi-winter
Spring_Semi-winter
Spring_Semi-winter
Spring_Semi-winter
Spring_Semi-winter
Spring_Semi-winter
Spring_Semi-winter
Spring_Semi-winter
Spring_Semi-winter
Spring_Semi-winter
Spring_Semi-winter
Spring_Semi-winter
Spring_Semi-winter
Spring_Semi-winter
Spring_Semi-winter
Spring_Semi-winter
Spring_Semi-winter
Spring_Semi-winter
Spring_Semi-winter
Spring_Semi-winter
Spring_Semi-winter
Spring_Semi-winter
Spring_Semi-winter
Spring_Semi-winter
Spring_Semi-winter
Spring_Semi-winter
Spring_Semi-winter
Spring_Semi-winter
Spring_Semi-winter
Spring_Semi-winter
Spring_Semi-winter
Spring_Semi-winter
Spring_Semi-winter
Spring_Semi-winter
Spring_Semi-winter
Spring_Semi-winter

For Review Only

Spring_Semi-winter
Spring_Semi-winter
Spring_Semi-winter
Spring_Semi-winter
Spring_Semi-winter
Spring_Semi-winter
Spring_Semi-winter
Spring_Semi-winter
Spring_Semi-winter
Spring_Semi-winter
Spring_Semi-winter
Spring_Semi-winter
Spring_Semi-winter
Spring_Semi-winter
Spring_Semi-winter
Spring_Semi-winter
Spring_Semi-winter
Spring_Semi-winter
Spring_Semi-winter
Spring_Semi-winter
Spring_Semi-winter
Spring_Semi-winter
Spring_Semi-winter
Spring_Semi-winter
Spring_Semi-winter
Spring_Semi-winter
Spring_Semi-winter
Spring_Semi-winter
Spring_Semi-winter
Spring_Semi-winter
Spring_Semi-winter
Spring_Semi-winter
Spring_Semi-winter
Spring_Semi-winter
Spring_Semi-winter
Spring_Semi-winter
Spring_Semi-winter
Spring_Semi-winter
Semi-winter_Spring
Semi-winter_Spring
Semi-winter_Spring
Semi-winter_Spring
Semi-winter_Spring
Semi-winter_Spring
Semi-winter_Spring
Semi-winter_Spring
Semi-winter_Spring
Semi-winter_Spring
Semi-winter_Spring
Semi-winter_Spring
Semi-winter_Spring
Semi-winter_Spring
Semi-winter_Spring
Semi-winter_Spring
Semi-winter_Spring
Semi-winter_Spring
Semi-winter_Spring
Semi-winter_Spring
Semi-winter_Spring
Semi-winter_Spring

For Review Only

Semi-winter_Spring
Semi-winter_Spring
Semi-winter_Spring
Semi-winter_Spring
Semi-winter_Spring
Semi-winter_Spring
Semi-winter_Spring
Semi-winter_Spring
Semi-winter_Spring
Semi-winter_Spring
Semi-winter_Spring
Semi-winter_Spring
Semi-winter_Spring
Semi-winter_Spring
Semi-winter_Spring
Semi-winter_Spring
Semi-winter_Spring
Semi-winter_Spring
Semi-winter_Spring
Semi-winter_Spring
Semi-winter_Spring
Semi-winter_Spring
Semi-winter_Spring
Semi-winter_Spring
Semi-winter_Spring
Semi-winter_Spring
Semi-winter_Spring
Semi-winter_Spring
Semi-winter_Spring
Semi-winter_Spring
Semi-winter_Spring
Semi-winter_Spring
Semi-winter_Spring
Semi-winter_Spring
Semi-winter_Spring
Semi-winter_Spring
Semi-winter_Spring
Semi-winter_Spring
Semi-winter_Spring
Semi-winter_Spring
Semi-winter_Spring
Semi-winter_Spring
Semi-winter_Spring
Semi-winter_Spring
Semi-winter_Spring
Semi-winter_Spring
Semi-winter_Spring
Semi-winter_Spring
Semi-winter_Spring
Semi-winter_Spring
Semi-winter_Spring
Semi-winter_Spring
Semi-winter_Spring
Semi-winter_Spring
Semi-winter_Spring
Semi-winter_Spring
Semi-winter_Spring
Semi-winter_Spring
Semi-winter_Spring
Semi-winter_Spring

For Review Only

Semi-winter_Spring
Semi-winter_Spring
Semi-winter_Spring
Semi-winter_Spring
Semi-winter_Spring
Semi-winter_Spring
Semi-winter_Spring
Semi-winter_Spring
Semi-winter_Spring
Semi-winter_Spring
Semi-winter_Spring
Semi-winter_Spring
Semi-winter_Spring
Semi-winter_Spring
Semi-winter_Spring
Semi-winter_Spring
Semi-winter_Spring
Semi-winter_Spring
Semi-winter_Spring
Semi-winter_Spring
Semi-winter_Spring
Semi-winter_Spring
Semi-winter_Spring
Semi-winter_Spring
Semi-winter_Spring
Semi-winter_Spring
Semi-winter_Spring
Semi-winter_Spring
Semi-winter_Spring
Semi-winter_Spring
Semi-winter_Spring
Semi-winter_Spring
Semi-winter_Spring
Semi-winter_Spring
Semi-winter_Spring
Semi-winter_Spring
Semi-winter_Spring
Semi-winter_Spring
Semi-winter_Spring
Semi-winter_Spring
Semi-winter_Spring
Semi-winter_Spring
Semi-winter_Spring
Semi-winter_Spring
Semi-winter_Spring
Semi-winter_Spring
Semi-winter_Spring
Semi-winter_Spring
Semi-winter_Spring
Semi-winter_Spring
Semi-winter_Spring
Semi-winter_Spring
Semi-winter_Spring
Semi-winter_Spring
Semi-winter_Spring
Semi-winter_Spring
Semi-winter_Spring
Semi-winter_Spring
Semi-winter_Spring
Semi-winter_Spring

For Review Only

Semi-winter_Spring
Semi-winter_Spring
Semi-winter_Spring
Semi-winter_Spring
Semi-winter_Spring
Semi-winter_Spring
Semi-winter_Spring
Semi-winter_Spring
Semi-winter_Spring
Semi-winter_Spring
Semi-winter_Spring
Semi-winter_Spring
Semi-winter_Spring
Semi-winter_Spring
Semi-winter_Spring
Semi-winter_Spring
Semi-winter_Spring
Semi-winter_Spring
Semi-winter_Spring
Semi-winter_Spring
Semi-winter_Spring
Semi-winter_Spring
Semi-winter_Spring
Semi-winter_Spring
Semi-winter_Spring
Semi-winter_Spring
Semi-winter_Spring
Semi-winter_Spring
Semi-winter_Spring
Semi-winter_Spring
Semi-winter_Spring
Semi-winter_Spring
Semi-winter_Spring
Semi-winter_Spring
Semi-winter_Spring
Semi-winter_Spring
Semi-winter_Spring
Semi-winter_Spring
Semi-winter_Spring
Semi-winter_Spring
Semi-winter_Spring
Semi-winter_Spring
Semi-winter_Spring
Semi-winter_Spring
Semi-winter_Spring
Semi-winter_Spring
Semi-winter_Spring
Semi-winter_Spring
Semi-winter_Spring
Semi-winter_Spring
Semi-winter_Winter
Semi-winter_Winter
Semi-winter_Winter
Semi-winter_Winter
Semi-winter_Winter
Semi-winter_Winter
Semi-winter_Winter
Semi-winter_Winter
Semi-winter_Winter
Semi-winter_Winter

For Review Only

Semi-winter_Winter
Semi-winter_Winter
Semi-winter_Winter
Semi-winter_Winter
Semi-winter_Winter
Semi-winter_Winter
Semi-winter_Winter
Semi-winter_Winter
Semi-winter_Winter
Semi-winter_Winter
Semi-winter_Winter
Semi-winter_Winter
Semi-winter_Winter
Semi-winter_Winter
Semi-winter_Winter
Semi-winter_Winter
Semi-winter_Winter
Semi-winter_Winter
Semi-winter_Winter
Semi-winter_Winter
Semi-winter_Winter
Semi-winter_Winter
Semi-winter_Winter
Semi-winter_Winter
Semi-winter_Winter
Semi-winter_Winter
Semi-winter_Winter
Semi-winter_Winter
Semi-winter_Winter
Semi-winter_Winter
Semi-winter_Winter
Semi-winter_Winter
Semi-winter_Winter
Semi-winter_Winter
Semi-winter_Winter
Semi-winter_Winter
Semi-winter_Winter
Semi-winter_Winter
Semi-winter_Winter
Semi-winter_Winter
Semi-winter_Winter
Semi-winter_Winter
Semi-winter_Winter
Semi-winter_Winter
Semi-winter_Winter
Semi-winter_Winter
Semi-winter_Winter
Semi-winter_Winter
Semi-winter_Winter
Semi-winter_Winter
Semi-winter_Winter
Semi-winter_Winter
Semi-winter_Winter
Semi-winter_Winter
Semi-winter_Winter
Semi-winter_Winter
Semi-winter_Winter
Semi-winter_Winter
Semi-winter_Winter
Semi-winter_Winter

For Review Only

Semi-winter_Winter
Semi-winter_Winter
Semi-winter_Winter
Semi-winter_Winter
Semi-winter_Winter
Semi-winter_Winter
Semi-winter_Winter
Semi-winter_Winter
Semi-winter_Winter
Semi-winter_Winter
Semi-winter_Winter
Semi-winter_Winter
Semi-winter_Winter
Semi-winter_Winter
Semi-winter_Winter
Semi-winter_Winter
Semi-winter_Winter
Semi-winter_Winter
Semi-winter_Winter
Semi-winter_Winter
Semi-winter_Winter
Semi-winter_Winter
Semi-winter_Winter
Semi-winter_Winter
Semi-winter_Winter
Semi-winter_Winter
Semi-winter_Winter
Semi-winter_Winter
Semi-winter_Winter
Semi-winter_Winter
Semi-winter_Winter
Semi-winter_Winter
Semi-winter_Winter
Semi-winter_Winter
Semi-winter_Winter
Semi-winter_Winter
Semi-winter_Winter
Semi-winter_Winter
Semi-winter_Winter
Semi-winter_Winter
Semi-winter_Winter
Semi-winter_Winter
Semi-winter_Winter
Semi-winter_Winter
Semi-winter_Winter
Semi-winter_Winter
Semi-winter_Winter
Semi-winter_Winter
Semi-winter_Winter
Semi-winter_Winter
Semi-winter_Winter
Semi-winter_Winter
Semi-winter_Winter
Semi-winter_Winter
Semi-winter_Winter
Semi-winter_Winter
Semi-winter_Winter
Semi-winter_Winter
Semi-winter_Winter
Semi-winter_Winter

For Review Only

Semi-winter_Winter
Semi-winter_Winter
Semi-winter_Winter
Semi-winter_Winter
Semi-winter_Winter
Semi-winter_Winter
Semi-winter_Winter
Semi-winter_Winter
Semi-winter_Winter
Semi-winter_Winter
Semi-winter_Winter
Semi-winter_Winter
Semi-winter_Winter
Semi-winter_Winter
Semi-winter_Winter
Semi-winter_Winter
Semi-winter_Winter
Semi-winter_Winter
Semi-winter_Winter
Semi-winter_Winter
Semi-winter_Winter
Semi-winter_Winter
Semi-winter_Winter
Semi-winter_Winter
Semi-winter_Winter
Semi-winter_Winter
Semi-winter_Winter
Semi-winter_Winter
Semi-winter_Winter
Semi-winter_Winter
Semi-winter_Winter
Semi-winter_Winter
Semi-winter_Winter
Semi-winter_Winter
Semi-winter_Winter
Semi-winter_Winter
Semi-winter_Winter
Semi-winter_Winter
Semi-winter_Winter
Semi-winter_Winter
Semi-winter_Winter
Semi-winter_Winter
Semi-winter_Winter
Semi-winter_Winter
Semi-winter_Winter
Semi-winter_Winter
Semi-winter_Winter
Semi-winter_Winter
Semi-winter_Winter
Semi-winter_Winter
Semi-winter_Winter
Semi-winter_Winter
Semi-winter_Winter
Semi-winter_Winter
Semi-winter_Winter
Semi-winter_Winter
Semi-winter_Winter
Semi-winter_Winter
Semi-winter_Winter
Semi-winter_Winter

For Review Only

Semi-winter_Winter
Semi-winter_Winter
Spring_Winter
Spring_Winter
Spring_Winter
Spring_Winter
Spring_Winter
Spring_Winter
Spring_Winter
Spring_Winter
Spring_Winter
Spring_Winter
Spring_Winter
Spring_Winter
Spring_Winter
Spring_Winter
Spring_Winter
Spring_Winter
Spring_Winter
Spring_Winter
Spring_Winter
Spring_Winter
Spring_Winter
Spring_Winter
Spring_Winter
Spring_Winter
Spring_Winter
Spring_Winter
Spring_Winter
Spring_Winter
Spring_Winter
Spring_Winter
Spring_Winter
Spring_Winter
Spring_Winter
Spring_Winter
Spring_Winter
Spring_Winter
Spring_Winter
Spring_Winter
Spring_Winter
Spring_Winter
Spring_Winter
Spring_Winter
Spring_Winter
Spring_Winter
Spring_Winter
Spring_Winter
Spring_Winter
Spring_Winter
Spring_Winter
Spring_Winter
Spring_Winter
Spring_Winter
Spring_Winter
Spring_Winter
Spring_Winter
Spring_Winter
Spring_Winter
Spring_Winter

For Review Only

Spring_Winter
Spring_Winter
Spring_Winter
Spring_Winter
Spring_Winter
Spring_Winter
Spring_Winter
Spring_Winter
Spring_Winter
Spring_Winter
Spring_Winter
Spring_Winter
Spring_Winter
Spring_Winter
Spring_Winter
Spring_Winter
Spring_Winter
Spring_Winter
Spring_Winter
Spring_Winter
Spring_Winter
Spring_Winter
Spring_Winter
Spring_Winter
Spring_Winter
Spring_Winter
Spring_Winter
Spring_Winter
Spring_Winter
Spring_Winter
Spring_Winter
Spring_Winter
Spring_Winter
Spring_Winter
Spring_Winter
Spring_Winter
Spring_Winter
Spring_Winter
Spring_Winter
Spring_Winter
Spring_Winter
Spring_Winter
Spring_Winter
Spring_Winter
Spring_Winter
Spring_Winter
Spring_Winter
Spring_Winter
Spring_Winter
Spring_Winter
Spring_Winter
Spring_Winter
Spring_Winter
Spring_Winter
Spring_Winter
Spring_Winter
Spring_Winter
Spring_Winter
Spring_Winter
Spring_Winter

For Review Only

Spring_Winter
Spring_Winter
Spring_Winter
Spring_Winter
Spring_Winter
Spring_Winter
Spring_Winter
Spring_Winter
Spring_Winter
Spring_Winter
Spring_Winter
Spring_Winter
Spring_Winter
Spring_Winter
Spring_Winter
Spring_Winter
Spring_Winter
Spring_Winter
Spring_Winter
Spring_Winter
Spring_Winter
Spring_Winter
Spring_Winter
Spring_Winter
Spring_Winter
Spring_Winter
Spring_Winter
Spring_Winter
Spring_Winter
Spring_Winter
Spring_Winter
Spring_Winter
Spring_Winter
Spring_Winter
Spring_Winter
Spring_Winter
Spring_Winter
Spring_Winter
Spring_Winter
Spring_Winter
Spring_Winter
Spring_Winter
Spring_Winter
Spring_Winter
Spring_Winter
Spring_Winter
Spring_Winter
Spring_Winter
Spring_Winter
Spring_Winter
Spring_Winter
Spring_Winter
Spring_Winter
Spring_Winter
Spring_Winter
Spring_Winter
Spring_Winter
Spring_Winter
Spring_Winter
Spring_Winter

For Review Only

Spring_Winter
Spring_Winter
Spring_Winter
Spring_Winter
Spring_Winter
Spring_Winter
Spring_Winter
Spring_Winter
Spring_Winter
Spring_Winter
Spring_Winter
Spring_Winter
Spring_Winter
Spring_Winter
Winter_Spring
Winter_Spring
Winter_Spring
Winter_Spring
Winter_Spring
Winter_Spring
Winter_Spring
Winter_Spring
Winter_Spring
Winter_Spring
Winter_Spring
Winter_Spring
Winter_Spring
Winter_Spring
Winter_Spring
Winter_Spring
Winter_Spring
Winter_Spring
Winter_Spring
Winter_Spring
Winter_Spring
Winter_Spring
Winter_Spring
Winter_Spring
Winter_Spring
Winter_Spring
Winter_Spring
Winter_Spring
Winter_Spring
Winter_Spring
Winter_Spring
Winter_Spring
Winter_Spring
Winter_Spring
Winter_Spring
Winter_Spring
Winter_Spring
Winter_Spring
Winter_Spring
Winter_Spring
Winter_Spring
Winter_Spring
Winter_Spring
Winter_Spring
Winter_Spring
Winter_Spring

For Review Only

Winter_Spring
Winter_Spring
Winter_Spring
Winter_Spring
Winter_Spring
Winter_Spring
Winter_Spring
Winter_Spring
Winter_Spring
Winter_Spring
Winter_Spring
Winter_Spring
Winter_Spring
Winter_Spring
Winter_Spring
Winter_Spring
Winter_Spring
Winter_Spring
Winter_Spring
Winter_Spring
Winter_Spring
Winter_Spring
Winter_Spring
Winter_Spring
Winter_Spring
Winter_Spring
Winter_Spring
Winter_Spring
Winter_Spring
Winter_Spring
Winter_Spring
Winter_Spring
Winter_Spring
Winter_Spring
Winter_Spring
Winter_Spring
Winter_Spring
Winter_Spring
Winter_Spring
Winter_Spring
Winter_Spring
Winter_Spring
Winter_Spring
Winter_Spring
Winter_Spring
Winter_Spring
Winter_Spring
Winter_Spring
Winter_Spring
Winter_Spring
Winter_Spring
Winter_Spring
Winter_Spring
Winter_Spring
Winter_Spring
Winter_Spring
Winter_Spring
Winter_Spring
Winter_Spring
Winter_Spring

For Review Only

Winter_Spring
Winter_Spring
Winter_Spring
Winter_Spring
Winter_Spring
Winter_Spring
Winter_Spring
Winter_Spring
Winter_Spring
Winter_Spring
Winter_Spring
Winter_Spring
Winter_Spring
Winter_Spring
Winter_Spring
Winter_Spring
Winter_Spring
Winter_Spring
Winter_Spring
Winter_Spring
Winter_Spring
Winter_Spring
Winter_Spring
Winter_Spring
Winter_Spring
Winter_Spring
Winter_Spring
Winter_Spring
Winter_Spring
Winter_Spring
Winter_Spring
Winter_Spring
Winter_Spring
Winter_Spring
Winter_Spring
Winter_Spring
Winter_Spring
Winter_Spring
Winter_Spring
Winter_Spring
Winter_Spring
Winter_Spring
Winter_Spring
Winter_Spring
Winter_Spring
Winter_Spring
Winter_Spring
Winter_Spring
Winter_Spring
Winter_Spring
Winter_Spring
Winter_Spring
Winter_Spring
Winter_Spring
Winter_Spring
Winter_Spring
Winter_Spring
Winter_Spring
Winter_Spring
Winter_Spring

For Review Only

Winter_Spring
Winter_Spring
Winter_Spring
Winter_Spring
Winter_Spring
Winter_Spring
Winter_Spring
Winter_Spring
Winter_Spring
Winter_Spring
Winter_Spring
Winter_Spring
Winter_Spring
Winter_Spring
Winter_Spring
Winter_Spring
Winter_Spring
Winter_Spring
Winter_Spring
Winter_Spring
Winter_Spring
Winter_Spring
Winter_Spring
Winter_Spring
Winter_Spring
Winter_Spring
Winter_Semi-winter
Winter_Semi-winter
Winter_Semi-winter
Winter_Semi-winter
Winter_Semi-winter
Winter_Semi-winter
Winter_Semi-winter
Winter_Semi-winter
Winter_Semi-winter
Winter_Semi-winter
Winter_Semi-winter
Winter_Semi-winter
Winter_Semi-winter
Winter_Semi-winter
Winter_Semi-winter
Winter_Semi-winter
Winter_Semi-winter
Winter_Semi-winter
Winter_Semi-winter
Winter_Semi-winter
Winter_Semi-winter
Winter_Semi-winter
Winter_Semi-winter
Winter_Semi-winter
Winter_Semi-winter
Winter_Semi-winter
Winter_Semi-winter
Winter_Semi-winter
Winter_Semi-winter
Winter_Semi-winter
Winter_Semi-winter
Winter_Semi-winter
Winter_Semi-winter
Winter_Semi-winter

For Review Only

Winter_Semi-winter
Winter_Semi-winter
Winter_Semi-winter
Winter_Semi-winter
Winter_Semi-winter
Winter_Semi-winter
Winter_Semi-winter
Winter_Semi-winter
Winter_Semi-winter
Winter_Semi-winter
Winter_Semi-winter
Winter_Semi-winter
Winter_Semi-winter
Winter_Semi-winter
Winter_Semi-winter
Winter_Semi-winter
Winter_Semi-winter
Winter_Semi-winter
Winter_Semi-winter
Winter_Semi-winter
Winter_Semi-winter
Winter_Semi-winter
Winter_Semi-winter
Winter_Semi-winter
Winter_Semi-winter
Winter_Semi-winter
Winter_Semi-winter
Winter_Semi-winter
Winter_Semi-winter
Winter_Semi-winter
Winter_Semi-winter
Winter_Semi-winter
Winter_Semi-winter
Winter_Semi-winter
Winter_Semi-winter
Winter_Semi-winter
Winter_Semi-winter
Winter_Semi-winter
Winter_Semi-winter
Winter_Semi-winter
Winter_Semi-winter
Winter_Semi-winter
Winter_Semi-winter
Winter_Semi-winter
Winter_Semi-winter
Winter_Semi-winter
Winter_Semi-winter
Winter_Semi-winter
Winter_Semi-winter
Winter_Semi-winter
Winter_Semi-winter
Winter_Semi-winter
Winter_Semi-winter
Winter_Semi-winter
Winter_Semi-winter
Winter_Semi-winter
Winter_Semi-winter
Winter_Semi-winter
Winter_Semi-winter
Winter_Semi-winter

For Review Only

Winter_Semi-winter
Winter_Semi-winter
Winter_Semi-winter
Winter_Semi-winter
Winter_Semi-winter
Winter_Semi-winter
Winter_Semi-winter
Winter_Semi-winter
Winter_Semi-winter
Winter_Semi-winter
Winter_Semi-winter
Winter_Semi-winter
Winter_Semi-winter
Winter_Semi-winter
Winter_Semi-winter
Winter_Semi-winter
Winter_Semi-winter
Winter_Semi-winter
Winter_Semi-winter
Winter_Semi-winter
Winter_Semi-winter
Winter_Semi-winter
Winter_Semi-winter
Winter_Semi-winter
Winter_Semi-winter
Winter_Semi-winter
Winter_Semi-winter
Winter_Semi-winter
Winter_Semi-winter
Winter_Semi-winter
Winter_Semi-winter
Winter_Semi-winter
Winter_Semi-winter
Winter_Semi-winter
Winter_Semi-winter
Winter_Semi-winter
Winter_Semi-winter
Winter_Semi-winter
Winter_Semi-winter
Winter_Semi-winter
Winter_Semi-winter
Winter_Semi-winter
Winter_Semi-winter
Winter_Semi-winter
Winter_Semi-winter
Winter_Semi-winter
Winter_Semi-winter
Winter_Semi-winter
Winter_Semi-winter
Winter_Semi-winter
Winter_Semi-winter
Winter_Semi-winter
Winter_Semi-winter
Winter_Semi-winter
Winter_Semi-winter
Winter_Semi-winter
Winter_Semi-winter
Winter_Semi-winter
Winter_Semi-winter
Winter_Semi-winter

For Review Only

Winter_Semi-winter
Winter_Semi-winter
Winter_Semi-winter
Winter_Semi-winter
Winter_Semi-winter
Winter_Semi-winter
Winter_Semi-winter
Winter_Semi-winter
Winter_Semi-winter
Winter_Semi-winter
Winter_Semi-winter
Winter_Semi-winter
Winter_Semi-winter
Winter_Semi-winter
Winter_Semi-winter
Winter_Semi-winter
Winter_Semi-winter
Winter_Semi-winter
Winter_Semi-winter
Winter_Semi-winter
Winter_Semi-winter
Winter_Semi-winter
Winter_Semi-winter
Winter_Semi-winter
Winter_Semi-winter
Winter_Semi-winter
Winter_Semi-winter
Winter_Semi-winter
Winter_Semi-winter
Winter_Semi-winter
Winter_Semi-winter
Winter_Semi-winter
Winter_Semi-winter
Winter_Semi-winter
Winter_Semi-winter
Winter_Semi-winter
Winter_Semi-winter
Winter_Semi-winter
Spring_Semi-winter
Spring_Semi-winter
Spring_Semi-winter
Spring_Semi-winter
Spring_Semi-winter
Spring_Semi-winter
Spring_Semi-winter
Spring_Semi-winter
Spring_Semi-winter
Spring_Semi-winter
Spring_Semi-winter
Spring_Semi-winter
Spring_Semi-winter
Spring_Semi-winter
Spring_Semi-winter
Spring_Semi-winter
Spring_Semi-winter
Spring_Semi-winter
Spring_Semi-winter
Spring_Semi-winter
Spring_Semi-winter
Spring_Semi-winter

For Review Only

Spring_Semi-winter
Spring_Semi-winter
Spring_Semi-winter
Spring_Semi-winter
Spring_Semi-winter
Spring_Semi-winter
Spring_Semi-winter
Spring_Semi-winter
Spring_Semi-winter
Spring_Semi-winter
Spring_Semi-winter
Spring_Semi-winter
Spring_Semi-winter
Spring_Semi-winter
Spring_Semi-winter
Spring_Semi-winter
Spring_Semi-winter
Spring_Semi-winter
Spring_Semi-winter
Spring_Semi-winter
Spring_Semi-winter
Spring_Semi-winter
Spring_Semi-winter
Spring_Semi-winter
Spring_Semi-winter
Spring_Semi-winter
Spring_Semi-winter
Spring_Semi-winter
Spring_Semi-winter
Spring_Semi-winter
Spring_Semi-winter
Spring_Semi-winter
Spring_Semi-winter
Spring_Semi-winter
Spring_Semi-winter
Spring_Semi-winter
Spring_Semi-winter
Spring_Semi-winter
Spring_Semi-winter
Spring_Semi-winter
Spring_Semi-winter
Spring_Semi-winter
Spring_Semi-winter
Spring_Semi-winter
Spring_Semi-winter
Spring_Semi-winter
Spring_Semi-winter
Spring_Semi-winter
Spring_Semi-winter
Spring_Semi-winter
Spring_Semi-winter
Spring_Semi-winter
Spring_Semi-winter
Spring_Semi-winter
Spring_Semi-winter
Spring_Semi-winter
Spring_Semi-winter
Spring_Semi-winter
Spring_Semi-winter
Spring_Semi-winter

For Review Only

Spring_Semi-winter
Spring_Semi-winter
Spring_Semi-winter
Spring_Semi-winter
Spring_Semi-winter
Spring_Semi-winter
Spring_Semi-winter
Spring_Semi-winter
Spring_Semi-winter
Spring_Semi-winter
Spring_Semi-winter
Spring_Semi-winter
Spring_Semi-winter
Spring_Semi-winter
Spring_Semi-winter
Spring_Semi-winter
Spring_Semi-winter
Spring_Semi-winter
Spring_Semi-winter
Spring_Semi-winter
Spring_Semi-winter
Spring_Semi-winter
Spring_Semi-winter
Spring_Semi-winter
Spring_Semi-winter
Spring_Semi-winter
Spring_Semi-winter
Spring_Semi-winter
Spring_Semi-winter
Spring_Semi-winter
Spring_Semi-winter
Spring_Semi-winter
Spring_Semi-winter
Spring_Semi-winter
Spring_Semi-winter
Spring_Semi-winter
Spring_Semi-winter
Spring_Semi-winter
Spring_Semi-winter
Spring_Semi-winter
Spring_Semi-winter
Spring_Semi-winter
Spring_Semi-winter
Spring_Semi-winter
Spring_Semi-winter
Spring_Semi-winter
Spring_Semi-winter
Spring_Semi-winter
Spring_Semi-winter
Spring_Semi-winter
Spring_Semi-winter
Spring_Semi-winter
Spring_Semi-winter
Spring_Semi-winter
Spring_Semi-winter
Spring_Semi-winter
Spring_Semi-winter
Spring_Semi-winter
Spring_Semi-winter
Spring_Semi-winter

For Review Only

Spring_Semi-winter
Spring_Semi-winter
Spring_Semi-winter
Spring_Semi-winter
Spring_Semi-winter
Spring_Semi-winter
Spring_Semi-winter
Spring_Semi-winter
Spring_Semi-winter
Spring_Semi-winter
Spring_Semi-winter
Spring_Semi-winter
Spring_Semi-winter
Spring_Semi-winter
Spring_Semi-winter
Spring_Semi-winter
Spring_Semi-winter
Spring_Semi-winter
Spring_Semi-winter
Spring_Semi-winter
Spring_Semi-winter
Spring_Semi-winter
Spring_Semi-winter
Spring_Semi-winter
Spring_Semi-winter
Spring_Semi-winter
Spring_Semi-winter
Spring_Semi-winter
Spring_Semi-winter
Spring_Semi-winter
Spring_Semi-winter
Spring_Semi-winter
Spring_Semi-winter
Spring_Semi-winter
Spring_Semi-winter
Spring_Semi-winter
Spring_Semi-winter
Spring_Semi-winter
Spring_Semi-winter
Spring_Semi-winter
Spring_Semi-winter
Spring_Semi-winter
Spring_Semi-winter
Spring_Semi-winter
Spring_Semi-winter
Spring_Semi-winter
Spring_Semi-winter
Spring_Semi-winter
Spring_Semi-winter
Spring_Semi-winter
Semi-winter_Spring
Semi-winter_Spring
Semi-winter_Spring
Semi-winter_Spring
Semi-winter_Spring
Semi-winter_Spring
Semi-winter_Spring
Semi-winter_Spring
Semi-winter_Spring
Semi-winter_Spring

For Review Only

Semi-winter_Spring
Semi-winter_Spring
Semi-winter_Spring
Semi-winter_Spring
Semi-winter_Spring
Semi-winter_Spring
Semi-winter_Spring
Semi-winter_Spring
Semi-winter_Spring
Semi-winter_Spring
Semi-winter_Spring
Semi-winter_Spring
Semi-winter_Spring
Semi-winter_Spring
Semi-winter_Spring
Semi-winter_Spring
Semi-winter_Spring
Semi-winter_Spring
Semi-winter_Spring
Semi-winter_Spring
Semi-winter_Spring
Semi-winter_Spring
Semi-winter_Spring
Semi-winter_Spring
Semi-winter_Spring
Semi-winter_Spring
Semi-winter_Spring
Semi-winter_Spring
Semi-winter_Spring
Semi-winter_Spring
Semi-winter_Spring
Semi-winter_Spring
Semi-winter_Spring
Semi-winter_Spring
Semi-winter_Spring
Semi-winter_Spring
Semi-winter_Spring
Semi-winter_Spring
Semi-winter_Spring
Semi-winter_Spring
Semi-winter_Spring
Semi-winter_Spring
Semi-winter_Spring
Semi-winter_Spring
Semi-winter_Spring
Semi-winter_Spring
Semi-winter_Spring
Semi-winter_Spring
Semi-winter_Spring
Semi-winter_Spring
Semi-winter_Spring
Semi-winter_Spring
Semi-winter_Spring
Semi-winter_Spring
Semi-winter_Spring
Semi-winter_Spring
Semi-winter_Spring
Semi-winter_Spring
Semi-winter_Spring
Semi-winter_Spring

For Review Only

Semi-winter_Spring
Semi-winter_Spring
Semi-winter_Spring
Semi-winter_Spring
Semi-winter_Spring
Semi-winter_Spring
Semi-winter_Spring
Semi-winter_Spring
Semi-winter_Spring
Semi-winter_Spring
Semi-winter_Spring
Semi-winter_Spring
Semi-winter_Spring
Semi-winter_Spring
Semi-winter_Spring
Semi-winter_Spring
Semi-winter_Spring
Semi-winter_Spring
Semi-winter_Spring
Semi-winter_Spring
Semi-winter_Spring
Semi-winter_Spring
Semi-winter_Spring
Semi-winter_Spring
Semi-winter_Spring
Semi-winter_Spring
Semi-winter_Spring
Semi-winter_Spring
Semi-winter_Spring
Semi-winter_Spring
Semi-winter_Spring
Semi-winter_Spring
Semi-winter_Spring
Semi-winter_Spring
Semi-winter_Spring
Semi-winter_Spring
Semi-winter_Spring
Semi-winter_Spring
Semi-winter_Spring
Semi-winter_Spring
Semi-winter_Spring
Semi-winter_Spring
Semi-winter_Spring
Semi-winter_Spring
Semi-winter_Spring
Semi-winter_Spring
Semi-winter_Spring
Semi-winter_Spring
Semi-winter_Spring
Semi-winter_Spring
Semi-winter_Spring
Semi-winter_Spring
Semi-winter_Spring
Semi-winter_Spring
Semi-winter_Spring
Semi-winter_Spring
Semi-winter_Spring
Semi-winter_Spring
Semi-winter_Spring
Semi-winter_Spring

For Review Only

Semi-winter_Spring
Semi-winter_Spring
Semi-winter_Spring
Semi-winter_Spring
Semi-winter_Spring
Semi-winter_Spring
Semi-winter_Spring
Semi-winter_Spring
Semi-winter_Spring
Semi-winter_Spring
Semi-winter_Spring
Semi-winter_Spring
Semi-winter_Spring
Semi-winter_Spring
Semi-winter_Spring
Semi-winter_Spring
Semi-winter_Spring
Semi-winter_Spring
Semi-winter_Spring
Semi-winter_Spring
Semi-winter_Spring
Semi-winter_Spring
Semi-winter_Spring
Semi-winter_Spring
Semi-winter_Spring
Semi-winter_Spring
Semi-winter_Spring
Semi-winter_Spring
Semi-winter_Spring
Semi-winter_Spring
Semi-winter_Spring
Semi-winter_Spring
Semi-winter_Spring
Semi-winter_Spring
Semi-winter_Spring
Semi-winter_Spring
Semi-winter_Spring
Semi-winter_Spring
Semi-winter_Spring
Semi-winter_Spring
Semi-winter_Spring
Semi-winter_Spring
Semi-winter_Spring
Semi-winter_Spring
Semi-winter_Spring
Semi-winter_Spring
Semi-winter_Spring
Semi-winter_Spring
Semi-winter_Spring
Semi-winter_Spring
Semi-winter_Spring
Semi-winter_Spring
Semi-winter_Spring
Semi-winter_Spring
Semi-winter_Spring
Semi-winter_Spring
Semi-winter_Spring
Semi-winter_Spring
Semi-winter_Spring
Semi-winter_Spring

For Review Only

Semi-winter_Spring
Semi-winter_Spring
Semi-winter_Winter
Semi-winter_Winter
Semi-winter_Winter
Semi-winter_Winter
Semi-winter_Winter
Semi-winter_Winter
Semi-winter_Winter
Semi-winter_Winter
Semi-winter_Winter
Semi-winter_Winter
Semi-winter_Winter
Semi-winter_Winter
Semi-winter_Winter
Semi-winter_Winter
Semi-winter_Winter
Semi-winter_Winter
Semi-winter_Winter
Semi-winter_Winter
Semi-winter_Winter
Semi-winter_Winter
Semi-winter_Winter
Semi-winter_Winter
Semi-winter_Winter
Semi-winter_Winter
Semi-winter_Winter
Semi-winter_Winter
Semi-winter_Winter
Semi-winter_Winter
Semi-winter_Winter
Semi-winter_Winter
Semi-winter_Winter
Semi-winter_Winter
Semi-winter_Winter
Semi-winter_Winter
Semi-winter_Winter
Semi-winter_Winter
Semi-winter_Winter
Semi-winter_Winter
Semi-winter_Winter
Semi-winter_Winter
Semi-winter_Winter
Semi-winter_Winter
Semi-winter_Winter
Semi-winter_Winter
Semi-winter_Winter
Semi-winter_Winter
Semi-winter_Winter
Semi-winter_Winter
Semi-winter_Winter
Semi-winter_Winter
Semi-winter_Winter
Semi-winter_Winter
Semi-winter_Winter
Semi-winter_Winter
Semi-winter_Winter
Semi-winter_Winter
Semi-winter_Winter
Semi-winter_Winter
Semi-winter_Winter

For Review Only

Semi-winter_Winter
Semi-winter_Winter
Semi-winter_Winter
Semi-winter_Winter
Semi-winter_Winter
Semi-winter_Winter
Semi-winter_Winter
Semi-winter_Winter
Semi-winter_Winter
Semi-winter_Winter
Semi-winter_Winter
Semi-winter_Winter
Semi-winter_Winter
Semi-winter_Winter
Semi-winter_Winter
Semi-winter_Winter
Semi-winter_Winter
Semi-winter_Winter
Semi-winter_Winter
Semi-winter_Winter
Semi-winter_Winter
Semi-winter_Winter
Semi-winter_Winter
Semi-winter_Winter
Semi-winter_Winter
Semi-winter_Winter
Semi-winter_Winter
Semi-winter_Winter
Semi-winter_Winter
Semi-winter_Winter
Semi-winter_Winter
Semi-winter_Winter
Semi-winter_Winter
Semi-winter_Winter
Semi-winter_Winter
Semi-winter_Winter
Semi-winter_Winter
Semi-winter_Winter
Semi-winter_Winter
Semi-winter_Winter
Semi-winter_Winter
Semi-winter_Winter
Semi-winter_Winter
Semi-winter_Winter
Semi-winter_Winter
Semi-winter_Winter
Semi-winter_Winter
Semi-winter_Winter
Semi-winter_Winter
Semi-winter_Winter
Semi-winter_Winter
Semi-winter_Winter
Semi-winter_Winter
Semi-winter_Winter
Semi-winter_Winter
Semi-winter_Winter
Semi-winter_Winter
Semi-winter_Winter
Semi-winter_Winter
Semi-winter_Winter

For Review Only

Semi-winter_Winter
Semi-winter_Winter
Semi-winter_Winter
Semi-winter_Winter
Semi-winter_Winter
Semi-winter_Winter
Semi-winter_Winter
Semi-winter_Winter
Semi-winter_Winter
Semi-winter_Winter
Semi-winter_Winter
Semi-winter_Winter
Semi-winter_Winter
Semi-winter_Winter
Semi-winter_Winter
Semi-winter_Winter
Semi-winter_Winter
Semi-winter_Winter
Semi-winter_Winter
Semi-winter_Winter
Semi-winter_Winter
Semi-winter_Winter
Semi-winter_Winter
Semi-winter_Winter
Semi-winter_Winter
Semi-winter_Winter
Semi-winter_Winter
Semi-winter_Winter
Semi-winter_Winter
Semi-winter_Winter
Semi-winter_Winter
Semi-winter_Winter
Semi-winter_Winter
Semi-winter_Winter
Semi-winter_Winter
Semi-winter_Winter
Semi-winter_Winter
Semi-winter_Winter
Semi-winter_Winter
Semi-winter_Winter
Semi-winter_Winter
Semi-winter_Winter
Semi-winter_Winter
Semi-winter_Winter
Semi-winter_Winter
Semi-winter_Winter
Semi-winter_Winter
Semi-winter_Winter
Semi-winter_Winter
Semi-winter_Winter
Semi-winter_Winter
Semi-winter_Winter
Semi-winter_Winter
Semi-winter_Winter
Semi-winter_Winter
Semi-winter_Winter
Semi-winter_Winter
Semi-winter_Winter
Semi-winter_Winter
Semi-winter_Winter

For Review Only

Semi-winter_Winter
Semi-winter_Winter
Semi-winter_Winter
Semi-winter_Winter
Semi-winter_Winter
Semi-winter_Winter
Semi-winter_Winter
Semi-winter_Winter
Semi-winter_Winter
Semi-winter_Winter
Semi-winter_Winter
Semi-winter_Winter
Semi-winter_Winter
Semi-winter_Winter
Spring_Winter
Spring_Winter
Spring_Winter
Spring_Winter
Spring_Winter
Spring_Winter
Spring_Winter
Spring_Winter
Spring_Winter
Spring_Winter
Spring_Winter
Spring_Winter
Spring_Winter
Spring_Winter
Spring_Winter
Spring_Winter
Spring_Winter
Spring_Winter
Spring_Winter
Spring_Winter
Spring_Winter
Spring_Winter
Spring_Winter
Spring_Winter
Spring_Winter
Spring_Winter
Spring_Winter
Spring_Winter
Spring_Winter
Spring_Winter
Spring_Winter
Spring_Winter
Spring_Winter
Spring_Winter
Spring_Winter
Spring_Winter
Spring_Winter
Spring_Winter
Spring_Winter
Spring_Winter
Spring_Winter
Spring_Winter
Spring_Winter
Spring_Winter
Spring_Winter
Spring_Winter

For Review Only

Spring_Winter
Spring_Winter
Spring_Winter
Spring_Winter
Spring_Winter
Spring_Winter
Spring_Winter
Spring_Winter
Spring_Winter
Spring_Winter
Spring_Winter
Spring_Winter
Spring_Winter
Spring_Winter
Spring_Winter
Spring_Winter
Spring_Winter
Spring_Winter
Spring_Winter
Spring_Winter
Spring_Winter
Spring_Winter
Spring_Winter
Spring_Winter
Spring_Winter
Spring_Winter
Spring_Winter
Spring_Winter
Spring_Winter
Spring_Winter
Spring_Winter
Spring_Winter
Spring_Winter
Spring_Winter
Spring_Winter
Spring_Winter
Spring_Winter
Spring_Winter
Spring_Winter
Spring_Winter
Spring_Winter
Spring_Winter
Spring_Winter
Spring_Winter
Spring_Winter
Spring_Winter
Spring_Winter
Spring_Winter
Spring_Winter
Spring_Winter
Spring_Winter
Spring_Winter
Spring_Winter
Spring_Winter
Spring_Winter
Spring_Winter
Spring_Winter
Spring_Winter
Spring_Winter
Spring_Winter

For Review Only

Spring_Winter
Spring_Winter
Spring_Winter
Spring_Winter
Spring_Winter
Spring_Winter
Spring_Winter
Spring_Winter
Spring_Winter
Spring_Winter
Spring_Winter
Spring_Winter
Spring_Winter
Spring_Winter
Spring_Winter
Spring_Winter
Spring_Winter
Spring_Winter
Spring_Winter
Spring_Winter
Spring_Winter
Spring_Winter
Spring_Winter
Spring_Winter
Spring_Winter
Spring_Winter
Spring_Winter
Spring_Winter
Spring_Winter
Spring_Winter
Spring_Winter
Spring_Winter
Spring_Winter
Spring_Winter
Spring_Winter
Spring_Winter
Spring_Winter
Spring_Winter
Spring_Winter
Spring_Winter
Spring_Winter
Spring_Winter
Spring_Winter
Spring_Winter
Spring_Winter
Spring_Winter
Spring_Winter
Spring_Winter
Spring_Winter
Spring_Winter
Spring_Winter
Spring_Winter
Spring_Winter
Spring_Winter
Spring_Winter
Spring_Winter
Spring_Winter
Spring_Winter
Spring_Winter
Spring_Winter

For Review Only

Spring_Winter
Spring_Winter
Spring_Winter
Spring_Winter
Spring_Winter
Spring_Winter
Spring_Winter
Spring_Winter
Spring_Winter
Spring_Winter
Spring_Winter
Spring_Winter
Spring_Winter
Spring_Winter
Spring_Winter
Spring_Winter
Spring_Winter
Spring_Winter
Spring_Winter
Spring_Winter
Spring_Winter
Spring_Winter
Spring_Winter
Spring_Winter
Spring_Winter
Spring_Winter

For Review Only

Table S3. π values in spring, semi-winter and winter-type accessions across the 1 Mb genomic region

panel	gene	chrom	start	end	n_bases	counts
BnaWGS-1007	BnaA02G0156900ZS	scaffoldA02	8104469	8204468	100000	1376
BnaWGS-1007	BnaA02G0156900ZS	scaffoldA02	8114469	8214468	100000	1411
BnaWGS-1007	BnaA02G0156900ZS	scaffoldA02	8124469	8224468	100000	1391
BnaWGS-1007	BnaA02G0156900ZS	scaffoldA02	8134469	8234468	100000	1206
BnaWGS-1007	BnaA02G0156900ZS	scaffoldA02	8144469	8244468	100000	1278
BnaWGS-1007	BnaA02G0156900ZS	scaffoldA02	8154469	8254468	100000	1239
BnaWGS-1007	BnaA02G0156900ZS	scaffoldA02	8164469	8264468	100000	1133
BnaWGS-1007	BnaA02G0156900ZS	scaffoldA02	8174469	8274468	100000	1097
BnaWGS-1007	BnaA02G0156900ZS	scaffoldA02	8184469	8284468	100000	1121
BnaWGS-1007	BnaA02G0156900ZS	scaffoldA02	8194469	8294468	100000	1049
BnaWGS-1007	BnaA02G0156900ZS	scaffoldA02	8204469	8304468	100000	1058
BnaWGS-1007	BnaA02G0156900ZS	scaffoldA02	8214469	8314468	100000	973
BnaWGS-1007	BnaA02G0156900ZS	scaffoldA02	8224469	8324468	100000	971
BnaWGS-1007	BnaA02G0156900ZS	scaffoldA02	8234469	8334468	100000	1034
BnaWGS-1007	BnaA02G0156900ZS	scaffoldA02	8244469	8344468	100000	941
BnaWGS-1007	BnaA02G0156900ZS	scaffoldA02	8254469	8354468	100000	898
BnaWGS-1007	BnaA02G0156900ZS	scaffoldA02	8264469	8364468	100000	1024
BnaWGS-1007	BnaA02G0156900ZS	scaffoldA02	8274469	8374468	100000	1058
BnaWGS-1007	BnaA02G0156900ZS	scaffoldA02	8284469	8384468	100000	1052
BnaWGS-1007	BnaA02G0156900ZS	scaffoldA02	8294469	8394468	100000	1001
BnaWGS-1007	BnaA02G0156900ZS	scaffoldA02	8304469	8404468	100000	934
BnaWGS-1007	BnaA02G0156900ZS	scaffoldA02	8314469	8414468	100000	914
BnaWGS-1007	BnaA02G0156900ZS	scaffoldA02	8324469	8424468	100000	964
BnaWGS-1007	BnaA02G0156900ZS	scaffoldA02	8334469	8434468	100000	935
BnaWGS-1007	BnaA02G0156900ZS	scaffoldA02	8344469	8444468	100000	858
BnaWGS-1007	BnaA02G0156900ZS	scaffoldA02	8354469	8454468	100000	831
BnaWGS-1007	BnaA02G0156900ZS	scaffoldA02	8364469	8464468	100000	648
BnaWGS-1007	BnaA02G0156900ZS	scaffoldA02	8374469	8474468	100000	545
BnaWGS-1007	BnaA02G0156900ZS	scaffoldA02	8384469	8484468	100000	455
BnaWGS-1007	BnaA02G0156900ZS	scaffoldA02	8394469	8494468	100000	432
BnaWGS-1007	BnaA02G0156900ZS	scaffoldA02	8404469	8504468	100000	420
BnaWGS-1007	BnaA02G0156900ZS	scaffoldA02	8414469	8514468	100000	354
BnaWGS-1007	BnaA02G0156900ZS	scaffoldA02	8424469	8524468	100000	196
BnaWGS-1007	BnaA02G0156900ZS	scaffoldA02	8434469	8534468	100000	122
BnaWGS-1007	BnaA02G0156900ZS	scaffoldA02	8444469	8544468	100000	87
BnaWGS-1007	BnaA02G0156900ZS	scaffoldA02	8454469	8554468	100000	38
BnaWGS-1007	BnaA02G0156900ZS	scaffoldA02	8464469	8564468	100000	80
BnaWGS-1007	BnaA02G0156900ZS	scaffoldA02	8474469	8574468	100000	149
BnaWGS-1007	BnaA02G0156900ZS	scaffoldA02	8484469	8584468	100000	160
BnaWGS-1007	BnaA02G0156900ZS	scaffoldA02	8494469	8594468	100000	190
BnaWGS-1007	BnaA02G0156900ZS	scaffoldA02	8504469	8604468	100000	312
BnaWGS-1007	BnaA02G0156900ZS	scaffoldA02	8514469	8614468	100000	444
BnaWGS-1007	BnaA02G0156900ZS	scaffoldA02	8524469	8624468	100000	468
BnaWGS-1007	BnaA02G0156900ZS	scaffoldA02	8534469	8634468	100000	579
BnaWGS-1007	BnaA02G0156900ZS	scaffoldA02	8544469	8644468	100000	646
BnaWGS-1007	BnaA02G0156900ZS	scaffoldA02	8554469	8654468	100000	756
BnaWGS-1007	BnaA02G0156900ZS	scaffoldA02	8564469	8664468	100000	770
BnaWGS-1007	BnaA02G0156900ZS	scaffoldA02	8574469	8674468	100000	778
BnaWGS-1007	BnaA02G0156900ZS	scaffoldA02	8584469	8684468	100000	791
BnaWGS-1007	BnaA02G0156900ZS	scaffoldA02	8594469	8694468	100000	831
BnaWGS-1007	BnaA02G0156900ZS	scaffoldA02	8604469	8704468	100000	815
BnaWGS-1007	BnaA02G0156900ZS	scaffoldA02	8614469	8714468	100000	831
BnaWGS-1007	BnaA02G0156900ZS	scaffoldA02	8624469	8724468	100000	995
BnaWGS-1007	BnaA02G0156900ZS	scaffoldA02	8634469	8734468	100000	1059
BnaWGS-1007	BnaA02G0156900ZS	scaffoldA02	8644469	8744468	100000	1035
BnaWGS-1007	BnaA02G0156900ZS	scaffoldA02	8654469	8754468	100000	939
BnaWGS-1007	BnaA02G0156900ZS	scaffoldA02	8664469	8764468	100000	919
BnaWGS-1007	BnaA02G0156900ZS	scaffoldA02	8674469	8774468	100000	936

For Review Only

BnaWGS-1007	BnaA02G0156900ZS	scaffoldA02	8684469	8784468	100000	1002
BnaWGS-1007	BnaA02G0156900ZS	scaffoldA02	8694469	8794468	100000	1029
BnaWGS-1007	BnaA02G0156900ZS	scaffoldA02	8704469	8804468	100000	1015
BnaWGS-1007	BnaA02G0156900ZS	scaffoldA02	8714469	8814468	100000	909
BnaWGS-1007	BnaA02G0156900ZS	scaffoldA02	8724469	8824468	100000	870
BnaWGS-1007	BnaA02G0156900ZS	scaffoldA02	8734469	8834468	100000	1032
BnaWGS-1007	BnaA02G0156900ZS	scaffoldA02	8744469	8844468	100000	1041
BnaWGS-1007	BnaA02G0156900ZS	scaffoldA02	8754469	8854468	100000	1032
BnaWGS-1007	BnaA02G0156900ZS	scaffoldA02	8764469	8864468	100000	1045
BnaWGS-1007	BnaA02G0156900ZS	scaffoldA02	8774469	8874468	100000	1056
BnaWGS-1007	BnaA02G0156900ZS	scaffoldA02	8784469	8884468	100000	1010
BnaWGS-1007	BnaA02G0156900ZS	scaffoldA02	8794469	8894468	100000	957
BnaWGS-1007	BnaA02G0156900ZS	scaffoldA02	8804469	8904468	100000	995
BnaWGS-1007	BnaA02G0156900ZS	scaffoldA02	8814469	8914468	100000	1191
BnaWGS-1007	BnaA02G0156900ZS	scaffoldA02	8824469	8924468	100000	1134
BnaWGS-1007	BnaA02G0156900ZS	scaffoldA02	8834469	8934468	100000	986
BnaWGS-1007	BnaA02G0156900ZS	scaffoldA02	8844469	8944468	100000	1109
BnaWGS-1007	BnaA02G0156900ZS	scaffoldA02	8854469	8954468	100000	1267
BnaWGS-1007	BnaA02G0156900ZS	scaffoldA02	8864469	8964468	100000	1394
BnaWGS-1007	BnaA02G0156900ZS	scaffoldA02	8874469	8974468	100000	1330
BnaWGS-1007	BnaA02G0156900ZS	scaffoldA02	8884469	8984468	100000	1450
BnaWGS-1007	BnaA02G0156900ZS	scaffoldA02	8894469	8994468	100000	1517
BnaWGS-1007	BnaA02G0156900ZS	scaffoldA02	8904469	9004468	100000	1448
BnaWGS-1007	BnaA02G0156900ZS	scaffoldA02	8914469	9014468	100000	1311
BnaWGS-1007	BnaA02G0156900ZS	scaffoldA02	8924469	9024468	100000	1341
BnaWGS-1007	BnaA02G0156900ZS	scaffoldA02	8934469	9034468	100000	1254
BnaWGS-1007	BnaA02G0156900ZS	scaffoldA02	8944469	9044468	100000	1185
BnaWGS-1007	BnaA02G0156900ZS	scaffoldA02	8954469	9054468	100000	1164
BnaWGS-1007	BnaA02G0156900ZS	scaffoldA02	8964469	9064468	100000	1165
BnaWGS-1007	BnaA02G0156900ZS	scaffoldA02	8974469	9074468	100000	1335
BnaWGS-1007	BnaA02G0156900ZS	scaffoldA02	8984469	9084468	100000	1331
BnaWGS-1007	BnaA02G0156900ZS	scaffoldA02	8994469	9094468	100000	1323
BnaWGS-1007	BnaA02G0156900ZS	scaffoldA02	9004469	9104468	100000	1309
BnaWGS-1007	BnaA02G0156900ZS	scaffoldA02	9014469	9114468	100000	1298
BnaWGS-1007	BnaA02G0156900ZS	scaffoldA02	9024469	9124468	100000	1312
BnaWGS-1007	BnaA02G0156900ZS	scaffoldA02	9034469	9134468	100000	1390
BnaWGS-1007	BnaA02G0156900ZS	scaffoldA02	9044469	9144468	100000	1438
BnaWGS-1007	BnaA02G0156900ZS	scaffoldA02	9054469	9154468	100000	1364
BnaWGS-1007	BnaA02G0156900ZS	scaffoldA02	9064469	9164468	100000	1231
BnaWGS-1007	BnaA02G0156900ZS	scaffoldA02	9074469	9174468	100000	1086
BnaWGS-1007	BnaA02G0156900ZS	scaffoldA02	9084469	9184468	100000	958
BnaWGS-1007	BnaA02G0156900ZS	scaffoldA02	9094469	9194468	100000	915
BnaWGS-1007	BnaA02G0156900ZS	scaffoldA02	9104469	9204468	100000	1040
BnaWGS-1007	BnaA02G0156900ZS	scaffoldA02	9114469	9214468	100000	1079
BnaWGS-1007	BnaA02G0156900ZS	scaffoldA02	9124469	9224468	100000	997
BnaWGS-1007	BnaA02G0156900ZS	scaffoldA02	9134469	9234468	100000	871
BnaWGS-1007	BnaA02G0156900ZS	scaffoldA02	9144469	9244468	100000	851
BnaWGS-1007	BnaA02G0156900ZS	scaffoldA02	9154469	9254468	100000	923
BnaWGS-1007	BnaA02G0156900ZS	scaffoldA02	9164469	9264468	100000	959
BnaWGS-1007	BnaA02G0156900ZS	scaffoldA02	9174469	9274468	100000	937
BnaWGS-1007	BnaA02G0156900ZS	scaffoldA02	9184469	9284468	100000	945
BnaWGS-1007	BnaA02G0156900ZS	scaffoldA02	9194469	9294468	100000	959
BnaWGS-1007	BnaA02G0156900ZS	scaffoldA02	9204469	9304468	100000	922
BnaWGS-1007	BnaA02G0156900ZS	scaffoldA02	9214469	9314468	100000	822
BnaWGS-1007	BnaA02G0156900ZS	scaffoldA02	9224469	9324468	100000	855
BnaWGS-1007	BnaA02G0156900ZS	scaffoldA02	9234469	9334468	100000	933
BnaWGS-1007	BnaA02G0156900ZS	scaffoldA02	9244469	9344468	100000	868
BnaWGS-1007	BnaA02G0156900ZS	scaffoldA02	9254469	9354468	100000	814
BnaWGS-1007	BnaA02G0156900ZS	scaffoldA02	9264469	9364468	100000	800
BnaWGS-1007	BnaA02G0156900ZS	scaffoldA02	9274469	9374468	100000	879

For Review Only

BnaWGS-1007	BnaA02G0156900ZS	scaffoldA02	9284469	9384468	100000	987
BnaWGS-1007	BnaA02G0156900ZS	scaffoldA02	9294469	9394468	100000	979
BnaWGS-1007	BnaA02G0156900ZS	scaffoldA02	9304469	9404468	100000	946
BnaWGS-1007	BnaA02G0156900ZS	scaffoldA02	9314469	9414468	100000	1028
BnaWGS-1007	BnaA02G0156900ZS	scaffoldA02	9324469	9424468	100000	1135
BnaWGS-1007	BnaA02G0156900ZS	scaffoldA02	9334469	9434468	100000	1082
BnaWGS-1007	BnaA02G0156900ZS	scaffoldA02	9344469	9444468	100000	1154
BnaWGS-1007	BnaA02G0156900ZS	scaffoldA02	9354469	9454468	100000	1109
BnaWGS-1007	BnaA02G0156900ZS	scaffoldA02	9364469	9464468	100000	1105
BnaWGS-1007	BnaA02G0156900ZS	scaffoldA02	9374469	9474468	100000	1014
BnaWGS-1007	BnaA02G0156900ZS	scaffoldA02	9384469	9484468	100000	970
BnaWGS-1007	BnaA02G0156900ZS	scaffoldA02	9394469	9494468	100000	1008
BnaWGS-1007	BnaA02G0156900ZS	scaffoldA02	9404469	9504468	100000	1012
BnaWGS-1007	BnaA02G0156900ZS	scaffoldA02	9414469	9514468	100000	902
BnaWGS-1007	BnaA02G0156900ZS	scaffoldA02	9424469	9524468	100000	746
BnaWGS-1007	BnaA02G0156900ZS	scaffoldA02	9434469	9534468	100000	889
BnaWGS-1007	BnaA02G0156900ZS	scaffoldA02	9444469	9544468	100000	856
BnaWGS-1007	BnaA02G0156900ZS	scaffoldA02	9454469	9554468	100000	888
BnaWGS-1007	BnaA02G0156900ZS	scaffoldA02	9464469	9564468	100000	892
BnaWGS-1007	BnaA02G0156900ZS	scaffoldA02	9474469	9574468	100000	999
BnaWGS-1007	BnaA02G0156900ZS	scaffoldA02	9484469	9584468	100000	910
BnaWGS-1007	BnaA02G0156900ZS	scaffoldA02	9494469	9594468	100000	914
BnaWGS-1007	BnaA02G0156900ZS	scaffoldA02	9504469	9604468	100000	953
BnaWGS-1007	BnaA02G0156900ZS	scaffoldA02	9514469	9614468	100000	964
BnaWGS-1007	BnaA02G0156900ZS	scaffoldA02	9524469	9624468	100000	957
BnaWGS-1007	BnaA02G0156900ZS	scaffoldA02	9534469	9634468	100000	804
BnaWGS-1007	BnaA02G0156900ZS	scaffoldA02	9544469	9644468	100000	790
BnaWGS-1007	BnaA02G0156900ZS	scaffoldA02	9554469	9654468	100000	903
BnaWGS-1007	BnaA02G0156900ZS	scaffoldA02	9564469	9664468	100000	913
BnaWGS-1007	BnaA02G0156900ZS	scaffoldA02	9574469	9674468	100000	808
BnaWGS-1007	BnaA02G0156900ZS	scaffoldA02	9584469	9684468	100000	847
BnaWGS-1007	BnaA02G0156900ZS	scaffoldA02	9594469	9694468	100000	796
BnaWGS-1007	BnaA02G0156900ZS	scaffoldA02	9604469	9704468	100000	730
BnaWGS-1007	BnaA02G0156900ZS	scaffoldA02	9614469	9714468	100000	868
BnaWGS-1007	BnaA02G0156900ZS	scaffoldA02	9624469	9724468	100000	906
BnaWGS-1007	BnaA02G0156900ZS	scaffoldA02	9634469	9734468	100000	1026
BnaWGS-1007	BnaA02G0156900ZS	scaffoldA02	9644469	9744468	100000	954
BnaWGS-1007	BnaA02G0156900ZS	scaffoldA02	9654469	9754468	100000	759
BnaWGS-1007	BnaA02G0156900ZS	scaffoldA02	9664469	9764468	100000	687
BnaWGS-1007	BnaA02G0156900ZS	scaffoldA02	9674469	9774468	100000	760
BnaWGS-1007	BnaA02G0156900ZS	scaffoldA02	9684469	9784468	100000	797
BnaWGS-1007	BnaA02G0156900ZS	scaffoldA02	9694469	9794468	100000	863
BnaWGS-1007	BnaA02G0156900ZS	scaffoldA02	9704469	9804468	100000	882
BnaWGS-1007	BnaA02G0156900ZS	scaffoldA02	9714469	9814468	100000	875
BnaWGS-1007	BnaA02G0156900ZS	scaffoldA02	9724469	9824468	100000	935
BnaWGS-1007	BnaA02G0156900ZS	scaffoldA02	9734469	9834468	100000	842
BnaWGS-1007	BnaA02G0156900ZS	scaffoldA02	9744469	9844468	100000	874
BnaWGS-1007	BnaA02G0156900ZS	scaffoldA02	9754469	9854468	100000	978
BnaWGS-1007	BnaA02G0156900ZS	scaffoldA02	9764469	9864468	100000	1116
BnaWGS-1007	BnaA02G0156900ZS	scaffoldA02	9774469	9874468	100000	1197
BnaWGS-1007	BnaA02G0156900ZS	scaffoldA02	9784469	9884468	100000	1299
BnaWGS-1007	BnaA02G0156900ZS	scaffoldA02	9794469	9894468	100000	1316
BnaWGS-1007	BnaA02G0156900ZS	scaffoldA02	9804469	9904468	100000	1301
BnaWGS-1007	BnaA02G0156900ZS	scaffoldA02	9814469	9914468	100000	1328
BnaWGS-1007	BnaA02G0156900ZS	scaffoldA02	9824469	9924468	100000	1346
BnaWGS-1007	BnaA02G0156900ZS	scaffoldA02	9834469	9934468	100000	1303
BnaWGS-1007	BnaA02G0156900ZS	scaffoldA02	9844469	9944468	100000	1366
BnaWGS-1007	BnaA02G0156900ZS	scaffoldA02	9854469	9954468	100000	1299
BnaWGS-1007	BnaA02G0156900ZS	scaffoldA02	9864469	9964468	100000	1210
BnaWGS-1007	BnaA02G0156900ZS	scaffoldA02	9874469	9974468	100000	1185

For Review Only

BnaWGS-1007	BnaA02G0156900ZS	scaffoldA02	9884469	9984468	100000	1061
BnaWGS-1007	BnaA02G0156900ZS	scaffoldA02	9894469	9994468	100000	1110
BnaWGS-1007	BnaA02G0156900ZS	scaffoldA02	9904469	10004468	100000	1145
BnaWGS-1007	BnaA02G0156900ZS	scaffoldA02	9914469	10014468	100000	1181
BnaWGS-1007	BnaA02G0156900ZS	scaffoldA02	9924469	10024468	100000	1077
BnaWGS-1007	BnaA02G0156900ZS	scaffoldA02	9934469	10034468	100000	1104
BnaWGS-1007	BnaA02G0156900ZS	scaffoldA02	9944469	10044468	100000	1152
BnaWGS-1007	BnaA02G0156900ZS	scaffoldA02	9954469	10054468	100000	1225
BnaWGS-1007	BnaA02G0156900ZS	scaffoldA02	9964469	10064468	100000	1247
BnaWGS-1007	BnaA02G0156900ZS	scaffoldA02	9974469	10074468	100000	1139
BnaWGS-1007	BnaA02G0156900ZS	scaffoldA02	9984469	10084468	100000	1125
BnaWGS-1007	BnaA02G0156900ZS	scaffoldA02	9994469	10094468	100000	1046
BnaWGS-1007	BnaA02G0156900ZS	scaffoldA02	10004469	10104468	100000	1001
BnaWGS-1007	BnaA02G0156900ZS	scaffoldA02	10014469	10107241	92773	823
BnaWGS-1007	BnaA02G0156900ZS	scaffoldA02	8104469	8204468	100000	1376
BnaWGS-1007	BnaA02G0156900ZS	scaffoldA02	8114469	8214468	100000	1411
BnaWGS-1007	BnaA02G0156900ZS	scaffoldA02	8124469	8224468	100000	1391
BnaWGS-1007	BnaA02G0156900ZS	scaffoldA02	8134469	8234468	100000	1206
BnaWGS-1007	BnaA02G0156900ZS	scaffoldA02	8144469	8244468	100000	1278
BnaWGS-1007	BnaA02G0156900ZS	scaffoldA02	8154469	8254468	100000	1239
BnaWGS-1007	BnaA02G0156900ZS	scaffoldA02	8164469	8264468	100000	1133
BnaWGS-1007	BnaA02G0156900ZS	scaffoldA02	8174469	8274468	100000	1097
BnaWGS-1007	BnaA02G0156900ZS	scaffoldA02	8184469	8284468	100000	1121
BnaWGS-1007	BnaA02G0156900ZS	scaffoldA02	8194469	8294468	100000	1049
BnaWGS-1007	BnaA02G0156900ZS	scaffoldA02	8204469	8304468	100000	1058
BnaWGS-1007	BnaA02G0156900ZS	scaffoldA02	8214469	8314468	100000	973
BnaWGS-1007	BnaA02G0156900ZS	scaffoldA02	8224469	8324468	100000	971
BnaWGS-1007	BnaA02G0156900ZS	scaffoldA02	8234469	8334468	100000	1034
BnaWGS-1007	BnaA02G0156900ZS	scaffoldA02	8244469	8344468	100000	941
BnaWGS-1007	BnaA02G0156900ZS	scaffoldA02	8254469	8354468	100000	898
BnaWGS-1007	BnaA02G0156900ZS	scaffoldA02	8264469	8364468	100000	1024
BnaWGS-1007	BnaA02G0156900ZS	scaffoldA02	8274469	8374468	100000	1058
BnaWGS-1007	BnaA02G0156900ZS	scaffoldA02	8284469	8384468	100000	1052
BnaWGS-1007	BnaA02G0156900ZS	scaffoldA02	8294469	8394468	100000	1001
BnaWGS-1007	BnaA02G0156900ZS	scaffoldA02	8304469	8404468	100000	934
BnaWGS-1007	BnaA02G0156900ZS	scaffoldA02	8314469	8414468	100000	914
BnaWGS-1007	BnaA02G0156900ZS	scaffoldA02	8324469	8424468	100000	964
BnaWGS-1007	BnaA02G0156900ZS	scaffoldA02	8334469	8434468	100000	935
BnaWGS-1007	BnaA02G0156900ZS	scaffoldA02	8344469	8444468	100000	858
BnaWGS-1007	BnaA02G0156900ZS	scaffoldA02	8354469	8454468	100000	831
BnaWGS-1007	BnaA02G0156900ZS	scaffoldA02	8364469	8464468	100000	648
BnaWGS-1007	BnaA02G0156900ZS	scaffoldA02	8374469	8474468	100000	545
BnaWGS-1007	BnaA02G0156900ZS	scaffoldA02	8384469	8484468	100000	455
BnaWGS-1007	BnaA02G0156900ZS	scaffoldA02	8394469	8494468	100000	432
BnaWGS-1007	BnaA02G0156900ZS	scaffoldA02	8404469	8504468	100000	420
BnaWGS-1007	BnaA02G0156900ZS	scaffoldA02	8414469	8514468	100000	354
BnaWGS-1007	BnaA02G0156900ZS	scaffoldA02	8424469	8524468	100000	196
BnaWGS-1007	BnaA02G0156900ZS	scaffoldA02	8434469	8534468	100000	122
BnaWGS-1007	BnaA02G0156900ZS	scaffoldA02	8444469	8544468	100000	87
BnaWGS-1007	BnaA02G0156900ZS	scaffoldA02	8454469	8554468	100000	38
BnaWGS-1007	BnaA02G0156900ZS	scaffoldA02	8464469	8564468	100000	80
BnaWGS-1007	BnaA02G0156900ZS	scaffoldA02	8474469	8574468	100000	149
BnaWGS-1007	BnaA02G0156900ZS	scaffoldA02	8484469	8584468	100000	160
BnaWGS-1007	BnaA02G0156900ZS	scaffoldA02	8494469	8594468	100000	190
BnaWGS-1007	BnaA02G0156900ZS	scaffoldA02	8504469	8604468	100000	312
BnaWGS-1007	BnaA02G0156900ZS	scaffoldA02	8514469	8614468	100000	444
BnaWGS-1007	BnaA02G0156900ZS	scaffoldA02	8524469	8624468	100000	468
BnaWGS-1007	BnaA02G0156900ZS	scaffoldA02	8534469	8634468	100000	579
BnaWGS-1007	BnaA02G0156900ZS	scaffoldA02	8544469	8644468	100000	646
BnaWGS-1007	BnaA02G0156900ZS	scaffoldA02	8554469	8654468	100000	756

For Review Only

BnaWGS-1007	BnaA02G0156900ZS	scaffoldA02	8564469	8664468	100000	770
BnaWGS-1007	BnaA02G0156900ZS	scaffoldA02	8574469	8674468	100000	778
BnaWGS-1007	BnaA02G0156900ZS	scaffoldA02	8584469	8684468	100000	791
BnaWGS-1007	BnaA02G0156900ZS	scaffoldA02	8594469	8694468	100000	831
BnaWGS-1007	BnaA02G0156900ZS	scaffoldA02	8604469	8704468	100000	815
BnaWGS-1007	BnaA02G0156900ZS	scaffoldA02	8614469	8714468	100000	831
BnaWGS-1007	BnaA02G0156900ZS	scaffoldA02	8624469	8724468	100000	995
BnaWGS-1007	BnaA02G0156900ZS	scaffoldA02	8634469	8734468	100000	1059
BnaWGS-1007	BnaA02G0156900ZS	scaffoldA02	8644469	8744468	100000	1035
BnaWGS-1007	BnaA02G0156900ZS	scaffoldA02	8654469	8754468	100000	939
BnaWGS-1007	BnaA02G0156900ZS	scaffoldA02	8664469	8764468	100000	919
BnaWGS-1007	BnaA02G0156900ZS	scaffoldA02	8674469	8774468	100000	936
BnaWGS-1007	BnaA02G0156900ZS	scaffoldA02	8684469	8784468	100000	1002
BnaWGS-1007	BnaA02G0156900ZS	scaffoldA02	8694469	8794468	100000	1029
BnaWGS-1007	BnaA02G0156900ZS	scaffoldA02	8704469	8804468	100000	1015
BnaWGS-1007	BnaA02G0156900ZS	scaffoldA02	8714469	8814468	100000	909
BnaWGS-1007	BnaA02G0156900ZS	scaffoldA02	8724469	8824468	100000	870
BnaWGS-1007	BnaA02G0156900ZS	scaffoldA02	8734469	8834468	100000	1032
BnaWGS-1007	BnaA02G0156900ZS	scaffoldA02	8744469	8844468	100000	1041
BnaWGS-1007	BnaA02G0156900ZS	scaffoldA02	8754469	8854468	100000	1032
BnaWGS-1007	BnaA02G0156900ZS	scaffoldA02	8764469	8864468	100000	1045
BnaWGS-1007	BnaA02G0156900ZS	scaffoldA02	8774469	8874468	100000	1056
BnaWGS-1007	BnaA02G0156900ZS	scaffoldA02	8784469	8884468	100000	1010
BnaWGS-1007	BnaA02G0156900ZS	scaffoldA02	8794469	8894468	100000	957
BnaWGS-1007	BnaA02G0156900ZS	scaffoldA02	8804469	8904468	100000	995
BnaWGS-1007	BnaA02G0156900ZS	scaffoldA02	8814469	8914468	100000	1191
BnaWGS-1007	BnaA02G0156900ZS	scaffoldA02	8824469	8924468	100000	1134
BnaWGS-1007	BnaA02G0156900ZS	scaffoldA02	8834469	8934468	100000	986
BnaWGS-1007	BnaA02G0156900ZS	scaffoldA02	8844469	8944468	100000	1109
BnaWGS-1007	BnaA02G0156900ZS	scaffoldA02	8854469	8954468	100000	1267
BnaWGS-1007	BnaA02G0156900ZS	scaffoldA02	8864469	8964468	100000	1394
BnaWGS-1007	BnaA02G0156900ZS	scaffoldA02	8874469	8974468	100000	1330
BnaWGS-1007	BnaA02G0156900ZS	scaffoldA02	8884469	8984468	100000	1450
BnaWGS-1007	BnaA02G0156900ZS	scaffoldA02	8894469	8994468	100000	1517
BnaWGS-1007	BnaA02G0156900ZS	scaffoldA02	8904469	9004468	100000	1448
BnaWGS-1007	BnaA02G0156900ZS	scaffoldA02	8914469	9014468	100000	1311
BnaWGS-1007	BnaA02G0156900ZS	scaffoldA02	8924469	9024468	100000	1341
BnaWGS-1007	BnaA02G0156900ZS	scaffoldA02	8934469	9034468	100000	1254
BnaWGS-1007	BnaA02G0156900ZS	scaffoldA02	8944469	9044468	100000	1185
BnaWGS-1007	BnaA02G0156900ZS	scaffoldA02	8954469	9054468	100000	1164
BnaWGS-1007	BnaA02G0156900ZS	scaffoldA02	8964469	9064468	100000	1165
BnaWGS-1007	BnaA02G0156900ZS	scaffoldA02	8974469	9074468	100000	1335
BnaWGS-1007	BnaA02G0156900ZS	scaffoldA02	8984469	9084468	100000	1331
BnaWGS-1007	BnaA02G0156900ZS	scaffoldA02	8994469	9094468	100000	1323
BnaWGS-1007	BnaA02G0156900ZS	scaffoldA02	9004469	9104468	100000	1309
BnaWGS-1007	BnaA02G0156900ZS	scaffoldA02	9014469	9114468	100000	1298
BnaWGS-1007	BnaA02G0156900ZS	scaffoldA02	9024469	9124468	100000	1312
BnaWGS-1007	BnaA02G0156900ZS	scaffoldA02	9034469	9134468	100000	1390
BnaWGS-1007	BnaA02G0156900ZS	scaffoldA02	9044469	9144468	100000	1438
BnaWGS-1007	BnaA02G0156900ZS	scaffoldA02	9054469	9154468	100000	1364
BnaWGS-1007	BnaA02G0156900ZS	scaffoldA02	9064469	9164468	100000	1231
BnaWGS-1007	BnaA02G0156900ZS	scaffoldA02	9074469	9174468	100000	1086
BnaWGS-1007	BnaA02G0156900ZS	scaffoldA02	9084469	9184468	100000	958
BnaWGS-1007	BnaA02G0156900ZS	scaffoldA02	9094469	9194468	100000	915
BnaWGS-1007	BnaA02G0156900ZS	scaffoldA02	9104469	9204468	100000	1040
BnaWGS-1007	BnaA02G0156900ZS	scaffoldA02	9114469	9214468	100000	1079
BnaWGS-1007	BnaA02G0156900ZS	scaffoldA02	9124469	9224468	100000	997
BnaWGS-1007	BnaA02G0156900ZS	scaffoldA02	9134469	9234468	100000	871
BnaWGS-1007	BnaA02G0156900ZS	scaffoldA02	9144469	9244468	100000	851
BnaWGS-1007	BnaA02G0156900ZS	scaffoldA02	9154469	9254468	100000	923

For Review Only

BnaWGS-1007	BnaA02G0156900ZS	scaffoldA02	9164469	9264468	100000	959
BnaWGS-1007	BnaA02G0156900ZS	scaffoldA02	9174469	9274468	100000	937
BnaWGS-1007	BnaA02G0156900ZS	scaffoldA02	9184469	9284468	100000	945
BnaWGS-1007	BnaA02G0156900ZS	scaffoldA02	9194469	9294468	100000	959
BnaWGS-1007	BnaA02G0156900ZS	scaffoldA02	9204469	9304468	100000	922
BnaWGS-1007	BnaA02G0156900ZS	scaffoldA02	9214469	9314468	100000	822
BnaWGS-1007	BnaA02G0156900ZS	scaffoldA02	9224469	9324468	100000	855
BnaWGS-1007	BnaA02G0156900ZS	scaffoldA02	9234469	9334468	100000	933
BnaWGS-1007	BnaA02G0156900ZS	scaffoldA02	9244469	9344468	100000	868
BnaWGS-1007	BnaA02G0156900ZS	scaffoldA02	9254469	9354468	100000	814
BnaWGS-1007	BnaA02G0156900ZS	scaffoldA02	9264469	9364468	100000	800
BnaWGS-1007	BnaA02G0156900ZS	scaffoldA02	9274469	9374468	100000	879
BnaWGS-1007	BnaA02G0156900ZS	scaffoldA02	9284469	9384468	100000	987
BnaWGS-1007	BnaA02G0156900ZS	scaffoldA02	9294469	9394468	100000	979
BnaWGS-1007	BnaA02G0156900ZS	scaffoldA02	9304469	9404468	100000	946
BnaWGS-1007	BnaA02G0156900ZS	scaffoldA02	9314469	9414468	100000	1028
BnaWGS-1007	BnaA02G0156900ZS	scaffoldA02	9324469	9424468	100000	1135
BnaWGS-1007	BnaA02G0156900ZS	scaffoldA02	9334469	9434468	100000	1082
BnaWGS-1007	BnaA02G0156900ZS	scaffoldA02	9344469	9444468	100000	1154
BnaWGS-1007	BnaA02G0156900ZS	scaffoldA02	9354469	9454468	100000	1109
BnaWGS-1007	BnaA02G0156900ZS	scaffoldA02	9364469	9464468	100000	1105
BnaWGS-1007	BnaA02G0156900ZS	scaffoldA02	9374469	9474468	100000	1014
BnaWGS-1007	BnaA02G0156900ZS	scaffoldA02	9384469	9484468	100000	970
BnaWGS-1007	BnaA02G0156900ZS	scaffoldA02	9394469	9494468	100000	1008
BnaWGS-1007	BnaA02G0156900ZS	scaffoldA02	9404469	9504468	100000	1012
BnaWGS-1007	BnaA02G0156900ZS	scaffoldA02	9414469	9514468	100000	902
BnaWGS-1007	BnaA02G0156900ZS	scaffoldA02	9424469	9524468	100000	746
BnaWGS-1007	BnaA02G0156900ZS	scaffoldA02	9434469	9534468	100000	889
BnaWGS-1007	BnaA02G0156900ZS	scaffoldA02	9444469	9544468	100000	856
BnaWGS-1007	BnaA02G0156900ZS	scaffoldA02	9454469	9554468	100000	888
BnaWGS-1007	BnaA02G0156900ZS	scaffoldA02	9464469	9564468	100000	892
BnaWGS-1007	BnaA02G0156900ZS	scaffoldA02	9474469	9574468	100000	999
BnaWGS-1007	BnaA02G0156900ZS	scaffoldA02	9484469	9584468	100000	910
BnaWGS-1007	BnaA02G0156900ZS	scaffoldA02	9494469	9594468	100000	914
BnaWGS-1007	BnaA02G0156900ZS	scaffoldA02	9504469	9604468	100000	953
BnaWGS-1007	BnaA02G0156900ZS	scaffoldA02	9514469	9614468	100000	964
BnaWGS-1007	BnaA02G0156900ZS	scaffoldA02	9524469	9624468	100000	957
BnaWGS-1007	BnaA02G0156900ZS	scaffoldA02	9534469	9634468	100000	804
BnaWGS-1007	BnaA02G0156900ZS	scaffoldA02	9544469	9644468	100000	790
BnaWGS-1007	BnaA02G0156900ZS	scaffoldA02	9554469	9654468	100000	903
BnaWGS-1007	BnaA02G0156900ZS	scaffoldA02	9564469	9664468	100000	913
BnaWGS-1007	BnaA02G0156900ZS	scaffoldA02	9574469	9674468	100000	808
BnaWGS-1007	BnaA02G0156900ZS	scaffoldA02	9584469	9684468	100000	847
BnaWGS-1007	BnaA02G0156900ZS	scaffoldA02	9594469	9694468	100000	796
BnaWGS-1007	BnaA02G0156900ZS	scaffoldA02	9604469	9704468	100000	730
BnaWGS-1007	BnaA02G0156900ZS	scaffoldA02	9614469	9714468	100000	868
BnaWGS-1007	BnaA02G0156900ZS	scaffoldA02	9624469	9724468	100000	906
BnaWGS-1007	BnaA02G0156900ZS	scaffoldA02	9634469	9734468	100000	1026
BnaWGS-1007	BnaA02G0156900ZS	scaffoldA02	9644469	9744468	100000	954
BnaWGS-1007	BnaA02G0156900ZS	scaffoldA02	9654469	9754468	100000	759
BnaWGS-1007	BnaA02G0156900ZS	scaffoldA02	9664469	9764468	100000	687
BnaWGS-1007	BnaA02G0156900ZS	scaffoldA02	9674469	9774468	100000	760
BnaWGS-1007	BnaA02G0156900ZS	scaffoldA02	9684469	9784468	100000	797
BnaWGS-1007	BnaA02G0156900ZS	scaffoldA02	9694469	9794468	100000	863
BnaWGS-1007	BnaA02G0156900ZS	scaffoldA02	9704469	9804468	100000	882
BnaWGS-1007	BnaA02G0156900ZS	scaffoldA02	9714469	9814468	100000	875
BnaWGS-1007	BnaA02G0156900ZS	scaffoldA02	9724469	9824468	100000	935
BnaWGS-1007	BnaA02G0156900ZS	scaffoldA02	9734469	9834468	100000	842
BnaWGS-1007	BnaA02G0156900ZS	scaffoldA02	9744469	9844468	100000	874
BnaWGS-1007	BnaA02G0156900ZS	scaffoldA02	9754469	9854468	100000	978

For Review Only

BnaWGS-1007	BnaA02G0156900ZS	scaffoldA02	9764469	9864468	100000	1116
BnaWGS-1007	BnaA02G0156900ZS	scaffoldA02	9774469	9874468	100000	1197
BnaWGS-1007	BnaA02G0156900ZS	scaffoldA02	9784469	9884468	100000	1299
BnaWGS-1007	BnaA02G0156900ZS	scaffoldA02	9794469	9894468	100000	1316
BnaWGS-1007	BnaA02G0156900ZS	scaffoldA02	9804469	9904468	100000	1301
BnaWGS-1007	BnaA02G0156900ZS	scaffoldA02	9814469	9914468	100000	1328
BnaWGS-1007	BnaA02G0156900ZS	scaffoldA02	9824469	9924468	100000	1346
BnaWGS-1007	BnaA02G0156900ZS	scaffoldA02	9834469	9934468	100000	1303
BnaWGS-1007	BnaA02G0156900ZS	scaffoldA02	9844469	9944468	100000	1366
BnaWGS-1007	BnaA02G0156900ZS	scaffoldA02	9854469	9954468	100000	1299
BnaWGS-1007	BnaA02G0156900ZS	scaffoldA02	9864469	9964468	100000	1210
BnaWGS-1007	BnaA02G0156900ZS	scaffoldA02	9874469	9974468	100000	1185
BnaWGS-1007	BnaA02G0156900ZS	scaffoldA02	9884469	9984468	100000	1061
BnaWGS-1007	BnaA02G0156900ZS	scaffoldA02	9894469	9994468	100000	1110
BnaWGS-1007	BnaA02G0156900ZS	scaffoldA02	9904469	10004468	100000	1145
BnaWGS-1007	BnaA02G0156900ZS	scaffoldA02	9914469	10014468	100000	1181
BnaWGS-1007	BnaA02G0156900ZS	scaffoldA02	9924469	10024468	100000	1077
BnaWGS-1007	BnaA02G0156900ZS	scaffoldA02	9934469	10034468	100000	1104
BnaWGS-1007	BnaA02G0156900ZS	scaffoldA02	9944469	10044468	100000	1152
BnaWGS-1007	BnaA02G0156900ZS	scaffoldA02	9954469	10054468	100000	1225
BnaWGS-1007	BnaA02G0156900ZS	scaffoldA02	9964469	10064468	100000	1247
BnaWGS-1007	BnaA02G0156900ZS	scaffoldA02	9974469	10074468	100000	1139
BnaWGS-1007	BnaA02G0156900ZS	scaffoldA02	9984469	10084468	100000	1125
BnaWGS-1007	BnaA02G0156900ZS	scaffoldA02	9994469	10094468	100000	1046
BnaWGS-1007	BnaA02G0156900ZS	scaffoldA02	10004469	10104468	100000	1001
BnaWGS-1007	BnaA02G0156900ZS	scaffoldA02	10014469	10107241	92773	823
BnaWGS-1007	BnaA02G0156900ZS	scaffoldA02	8104469	8204468	100000	1376
BnaWGS-1007	BnaA02G0156900ZS	scaffoldA02	8114469	8214468	100000	1411
BnaWGS-1007	BnaA02G0156900ZS	scaffoldA02	8124469	8224468	100000	1391
BnaWGS-1007	BnaA02G0156900ZS	scaffoldA02	8134469	8234468	100000	1206
BnaWGS-1007	BnaA02G0156900ZS	scaffoldA02	8144469	8244468	100000	1278
BnaWGS-1007	BnaA02G0156900ZS	scaffoldA02	8154469	8254468	100000	1239
BnaWGS-1007	BnaA02G0156900ZS	scaffoldA02	8164469	8264468	100000	1133
BnaWGS-1007	BnaA02G0156900ZS	scaffoldA02	8174469	8274468	100000	1097
BnaWGS-1007	BnaA02G0156900ZS	scaffoldA02	8184469	8284468	100000	1121
BnaWGS-1007	BnaA02G0156900ZS	scaffoldA02	8194469	8294468	100000	1049
BnaWGS-1007	BnaA02G0156900ZS	scaffoldA02	8204469	8304468	100000	1058
BnaWGS-1007	BnaA02G0156900ZS	scaffoldA02	8214469	8314468	100000	973
BnaWGS-1007	BnaA02G0156900ZS	scaffoldA02	8224469	8324468	100000	971
BnaWGS-1007	BnaA02G0156900ZS	scaffoldA02	8234469	8334468	100000	1034
BnaWGS-1007	BnaA02G0156900ZS	scaffoldA02	8244469	8344468	100000	941
BnaWGS-1007	BnaA02G0156900ZS	scaffoldA02	8254469	8354468	100000	898
BnaWGS-1007	BnaA02G0156900ZS	scaffoldA02	8264469	8364468	100000	1024
BnaWGS-1007	BnaA02G0156900ZS	scaffoldA02	8274469	8374468	100000	1058
BnaWGS-1007	BnaA02G0156900ZS	scaffoldA02	8284469	8384468	100000	1052
BnaWGS-1007	BnaA02G0156900ZS	scaffoldA02	8294469	8394468	100000	1001
BnaWGS-1007	BnaA02G0156900ZS	scaffoldA02	8304469	8404468	100000	934
BnaWGS-1007	BnaA02G0156900ZS	scaffoldA02	8314469	8414468	100000	914
BnaWGS-1007	BnaA02G0156900ZS	scaffoldA02	8324469	8424468	100000	964
BnaWGS-1007	BnaA02G0156900ZS	scaffoldA02	8334469	8434468	100000	935
BnaWGS-1007	BnaA02G0156900ZS	scaffoldA02	8344469	8444468	100000	858
BnaWGS-1007	BnaA02G0156900ZS	scaffoldA02	8354469	8454468	100000	831
BnaWGS-1007	BnaA02G0156900ZS	scaffoldA02	8364469	8464468	100000	648
BnaWGS-1007	BnaA02G0156900ZS	scaffoldA02	8374469	8474468	100000	545
BnaWGS-1007	BnaA02G0156900ZS	scaffoldA02	8384469	8484468	100000	455
BnaWGS-1007	BnaA02G0156900ZS	scaffoldA02	8394469	8494468	100000	432
BnaWGS-1007	BnaA02G0156900ZS	scaffoldA02	8404469	8504468	100000	420
BnaWGS-1007	BnaA02G0156900ZS	scaffoldA02	8414469	8514468	100000	354
BnaWGS-1007	BnaA02G0156900ZS	scaffoldA02	8424469	8524468	100000	196
BnaWGS-1007	BnaA02G0156900ZS	scaffoldA02	8434469	8534468	100000	122

For Review Only

BnaWGS-1007	BnaA02G0156900ZS	scaffoldA02	8444469	8544468	100000	87
BnaWGS-1007	BnaA02G0156900ZS	scaffoldA02	8454469	8554468	100000	38
BnaWGS-1007	BnaA02G0156900ZS	scaffoldA02	8464469	8564468	100000	80
BnaWGS-1007	BnaA02G0156900ZS	scaffoldA02	8474469	8574468	100000	149
BnaWGS-1007	BnaA02G0156900ZS	scaffoldA02	8484469	8584468	100000	160
BnaWGS-1007	BnaA02G0156900ZS	scaffoldA02	8494469	8594468	100000	190
BnaWGS-1007	BnaA02G0156900ZS	scaffoldA02	8504469	8604468	100000	312
BnaWGS-1007	BnaA02G0156900ZS	scaffoldA02	8514469	8614468	100000	444
BnaWGS-1007	BnaA02G0156900ZS	scaffoldA02	8524469	8624468	100000	468
BnaWGS-1007	BnaA02G0156900ZS	scaffoldA02	8534469	8634468	100000	579
BnaWGS-1007	BnaA02G0156900ZS	scaffoldA02	8544469	8644468	100000	646
BnaWGS-1007	BnaA02G0156900ZS	scaffoldA02	8554469	8654468	100000	756
BnaWGS-1007	BnaA02G0156900ZS	scaffoldA02	8564469	8664468	100000	770
BnaWGS-1007	BnaA02G0156900ZS	scaffoldA02	8574469	8674468	100000	778
BnaWGS-1007	BnaA02G0156900ZS	scaffoldA02	8584469	8684468	100000	791
BnaWGS-1007	BnaA02G0156900ZS	scaffoldA02	8594469	8694468	100000	831
BnaWGS-1007	BnaA02G0156900ZS	scaffoldA02	8604469	8704468	100000	815
BnaWGS-1007	BnaA02G0156900ZS	scaffoldA02	8614469	8714468	100000	831
BnaWGS-1007	BnaA02G0156900ZS	scaffoldA02	8624469	8724468	100000	995
BnaWGS-1007	BnaA02G0156900ZS	scaffoldA02	8634469	8734468	100000	1059
BnaWGS-1007	BnaA02G0156900ZS	scaffoldA02	8644469	8744468	100000	1035
BnaWGS-1007	BnaA02G0156900ZS	scaffoldA02	8654469	8754468	100000	939
BnaWGS-1007	BnaA02G0156900ZS	scaffoldA02	8664469	8764468	100000	919
BnaWGS-1007	BnaA02G0156900ZS	scaffoldA02	8674469	8774468	100000	936
BnaWGS-1007	BnaA02G0156900ZS	scaffoldA02	8684469	8784468	100000	1002
BnaWGS-1007	BnaA02G0156900ZS	scaffoldA02	8694469	8794468	100000	1029
BnaWGS-1007	BnaA02G0156900ZS	scaffoldA02	8704469	8804468	100000	1015
BnaWGS-1007	BnaA02G0156900ZS	scaffoldA02	8714469	8814468	100000	909
BnaWGS-1007	BnaA02G0156900ZS	scaffoldA02	8724469	8824468	100000	870
BnaWGS-1007	BnaA02G0156900ZS	scaffoldA02	8734469	8834468	100000	1032
BnaWGS-1007	BnaA02G0156900ZS	scaffoldA02	8744469	8844468	100000	1041
BnaWGS-1007	BnaA02G0156900ZS	scaffoldA02	8754469	8854468	100000	1032
BnaWGS-1007	BnaA02G0156900ZS	scaffoldA02	8764469	8864468	100000	1045
BnaWGS-1007	BnaA02G0156900ZS	scaffoldA02	8774469	8874468	100000	1056
BnaWGS-1007	BnaA02G0156900ZS	scaffoldA02	8784469	8884468	100000	1010
BnaWGS-1007	BnaA02G0156900ZS	scaffoldA02	8794469	8894468	100000	957
BnaWGS-1007	BnaA02G0156900ZS	scaffoldA02	8804469	8904468	100000	995
BnaWGS-1007	BnaA02G0156900ZS	scaffoldA02	8814469	8914468	100000	1191
BnaWGS-1007	BnaA02G0156900ZS	scaffoldA02	8824469	8924468	100000	1134
BnaWGS-1007	BnaA02G0156900ZS	scaffoldA02	8834469	8934468	100000	986
BnaWGS-1007	BnaA02G0156900ZS	scaffoldA02	8844469	8944468	100000	1109
BnaWGS-1007	BnaA02G0156900ZS	scaffoldA02	8854469	8954468	100000	1267
BnaWGS-1007	BnaA02G0156900ZS	scaffoldA02	8864469	8964468	100000	1394
BnaWGS-1007	BnaA02G0156900ZS	scaffoldA02	8874469	8974468	100000	1330
BnaWGS-1007	BnaA02G0156900ZS	scaffoldA02	8884469	8984468	100000	1450
BnaWGS-1007	BnaA02G0156900ZS	scaffoldA02	8894469	8994468	100000	1517
BnaWGS-1007	BnaA02G0156900ZS	scaffoldA02	8904469	9004468	100000	1448
BnaWGS-1007	BnaA02G0156900ZS	scaffoldA02	8914469	9014468	100000	1311
BnaWGS-1007	BnaA02G0156900ZS	scaffoldA02	8924469	9024468	100000	1341
BnaWGS-1007	BnaA02G0156900ZS	scaffoldA02	8934469	9034468	100000	1254
BnaWGS-1007	BnaA02G0156900ZS	scaffoldA02	8944469	9044468	100000	1185
BnaWGS-1007	BnaA02G0156900ZS	scaffoldA02	8954469	9054468	100000	1164
BnaWGS-1007	BnaA02G0156900ZS	scaffoldA02	8964469	9064468	100000	1165
BnaWGS-1007	BnaA02G0156900ZS	scaffoldA02	8974469	9074468	100000	1335
BnaWGS-1007	BnaA02G0156900ZS	scaffoldA02	8984469	9084468	100000	1331
BnaWGS-1007	BnaA02G0156900ZS	scaffoldA02	8994469	9094468	100000	1323
BnaWGS-1007	BnaA02G0156900ZS	scaffoldA02	9004469	9104468	100000	1309
BnaWGS-1007	BnaA02G0156900ZS	scaffoldA02	9014469	9114468	100000	1298
BnaWGS-1007	BnaA02G0156900ZS	scaffoldA02	9024469	9124468	100000	1312
BnaWGS-1007	BnaA02G0156900ZS	scaffoldA02	9034469	9134468	100000	1390

For Review Only

BnaWGS-1007	BnaA02G0156900ZS	scaffoldA02	9044469	9144468	100000	1438
BnaWGS-1007	BnaA02G0156900ZS	scaffoldA02	9054469	9154468	100000	1364
BnaWGS-1007	BnaA02G0156900ZS	scaffoldA02	9064469	9164468	100000	1231
BnaWGS-1007	BnaA02G0156900ZS	scaffoldA02	9074469	9174468	100000	1086
BnaWGS-1007	BnaA02G0156900ZS	scaffoldA02	9084469	9184468	100000	958
BnaWGS-1007	BnaA02G0156900ZS	scaffoldA02	9094469	9194468	100000	915
BnaWGS-1007	BnaA02G0156900ZS	scaffoldA02	9104469	9204468	100000	1040
BnaWGS-1007	BnaA02G0156900ZS	scaffoldA02	9114469	9214468	100000	1079
BnaWGS-1007	BnaA02G0156900ZS	scaffoldA02	9124469	9224468	100000	997
BnaWGS-1007	BnaA02G0156900ZS	scaffoldA02	9134469	9234468	100000	871
BnaWGS-1007	BnaA02G0156900ZS	scaffoldA02	9144469	9244468	100000	851
BnaWGS-1007	BnaA02G0156900ZS	scaffoldA02	9154469	9254468	100000	923
BnaWGS-1007	BnaA02G0156900ZS	scaffoldA02	9164469	9264468	100000	959
BnaWGS-1007	BnaA02G0156900ZS	scaffoldA02	9174469	9274468	100000	937
BnaWGS-1007	BnaA02G0156900ZS	scaffoldA02	9184469	9284468	100000	945
BnaWGS-1007	BnaA02G0156900ZS	scaffoldA02	9194469	9294468	100000	959
BnaWGS-1007	BnaA02G0156900ZS	scaffoldA02	9204469	9304468	100000	922
BnaWGS-1007	BnaA02G0156900ZS	scaffoldA02	9214469	9314468	100000	822
BnaWGS-1007	BnaA02G0156900ZS	scaffoldA02	9224469	9324468	100000	855
BnaWGS-1007	BnaA02G0156900ZS	scaffoldA02	9234469	9334468	100000	933
BnaWGS-1007	BnaA02G0156900ZS	scaffoldA02	9244469	9344468	100000	868
BnaWGS-1007	BnaA02G0156900ZS	scaffoldA02	9254469	9354468	100000	814
BnaWGS-1007	BnaA02G0156900ZS	scaffoldA02	9264469	9364468	100000	800
BnaWGS-1007	BnaA02G0156900ZS	scaffoldA02	9274469	9374468	100000	879
BnaWGS-1007	BnaA02G0156900ZS	scaffoldA02	9284469	9384468	100000	987
BnaWGS-1007	BnaA02G0156900ZS	scaffoldA02	9294469	9394468	100000	979
BnaWGS-1007	BnaA02G0156900ZS	scaffoldA02	9304469	9404468	100000	946
BnaWGS-1007	BnaA02G0156900ZS	scaffoldA02	9314469	9414468	100000	1028
BnaWGS-1007	BnaA02G0156900ZS	scaffoldA02	9324469	9424468	100000	1135
BnaWGS-1007	BnaA02G0156900ZS	scaffoldA02	9334469	9434468	100000	1082
BnaWGS-1007	BnaA02G0156900ZS	scaffoldA02	9344469	9444468	100000	1154
BnaWGS-1007	BnaA02G0156900ZS	scaffoldA02	9354469	9454468	100000	1109
BnaWGS-1007	BnaA02G0156900ZS	scaffoldA02	9364469	9464468	100000	1105
BnaWGS-1007	BnaA02G0156900ZS	scaffoldA02	9374469	9474468	100000	1014
BnaWGS-1007	BnaA02G0156900ZS	scaffoldA02	9384469	9484468	100000	970
BnaWGS-1007	BnaA02G0156900ZS	scaffoldA02	9394469	9494468	100000	1008
BnaWGS-1007	BnaA02G0156900ZS	scaffoldA02	9404469	9504468	100000	1012
BnaWGS-1007	BnaA02G0156900ZS	scaffoldA02	9414469	9514468	100000	902
BnaWGS-1007	BnaA02G0156900ZS	scaffoldA02	9424469	9524468	100000	746
BnaWGS-1007	BnaA02G0156900ZS	scaffoldA02	9434469	9534468	100000	889
BnaWGS-1007	BnaA02G0156900ZS	scaffoldA02	9444469	9544468	100000	856
BnaWGS-1007	BnaA02G0156900ZS	scaffoldA02	9454469	9554468	100000	888
BnaWGS-1007	BnaA02G0156900ZS	scaffoldA02	9464469	9564468	100000	892
BnaWGS-1007	BnaA02G0156900ZS	scaffoldA02	9474469	9574468	100000	999
BnaWGS-1007	BnaA02G0156900ZS	scaffoldA02	9484469	9584468	100000	910
BnaWGS-1007	BnaA02G0156900ZS	scaffoldA02	9494469	9594468	100000	914
BnaWGS-1007	BnaA02G0156900ZS	scaffoldA02	9504469	9604468	100000	953
BnaWGS-1007	BnaA02G0156900ZS	scaffoldA02	9514469	9614468	100000	964
BnaWGS-1007	BnaA02G0156900ZS	scaffoldA02	9524469	9624468	100000	957
BnaWGS-1007	BnaA02G0156900ZS	scaffoldA02	9534469	9634468	100000	804
BnaWGS-1007	BnaA02G0156900ZS	scaffoldA02	9544469	9644468	100000	790
BnaWGS-1007	BnaA02G0156900ZS	scaffoldA02	9554469	9654468	100000	903
BnaWGS-1007	BnaA02G0156900ZS	scaffoldA02	9564469	9664468	100000	913
BnaWGS-1007	BnaA02G0156900ZS	scaffoldA02	9574469	9674468	100000	808
BnaWGS-1007	BnaA02G0156900ZS	scaffoldA02	9584469	9684468	100000	847
BnaWGS-1007	BnaA02G0156900ZS	scaffoldA02	9594469	9694468	100000	796
BnaWGS-1007	BnaA02G0156900ZS	scaffoldA02	9604469	9704468	100000	730
BnaWGS-1007	BnaA02G0156900ZS	scaffoldA02	9614469	9714468	100000	868
BnaWGS-1007	BnaA02G0156900ZS	scaffoldA02	9624469	9724468	100000	906
BnaWGS-1007	BnaA02G0156900ZS	scaffoldA02	9634469	9734468	100000	1026

For Review Only

BnaWGS-1007	BnaA02G0156900ZS	scaffoldA02	9644469	9744468	100000	954
BnaWGS-1007	BnaA02G0156900ZS	scaffoldA02	9654469	9754468	100000	759
BnaWGS-1007	BnaA02G0156900ZS	scaffoldA02	9664469	9764468	100000	687
BnaWGS-1007	BnaA02G0156900ZS	scaffoldA02	9674469	9774468	100000	760
BnaWGS-1007	BnaA02G0156900ZS	scaffoldA02	9684469	9784468	100000	797
BnaWGS-1007	BnaA02G0156900ZS	scaffoldA02	9694469	9794468	100000	863
BnaWGS-1007	BnaA02G0156900ZS	scaffoldA02	9704469	9804468	100000	882
BnaWGS-1007	BnaA02G0156900ZS	scaffoldA02	9714469	9814468	100000	875
BnaWGS-1007	BnaA02G0156900ZS	scaffoldA02	9724469	9824468	100000	935
BnaWGS-1007	BnaA02G0156900ZS	scaffoldA02	9734469	9834468	100000	842
BnaWGS-1007	BnaA02G0156900ZS	scaffoldA02	9744469	9844468	100000	874
BnaWGS-1007	BnaA02G0156900ZS	scaffoldA02	9754469	9854468	100000	978
BnaWGS-1007	BnaA02G0156900ZS	scaffoldA02	9764469	9864468	100000	1116
BnaWGS-1007	BnaA02G0156900ZS	scaffoldA02	9774469	9874468	100000	1197
BnaWGS-1007	BnaA02G0156900ZS	scaffoldA02	9784469	9884468	100000	1299
BnaWGS-1007	BnaA02G0156900ZS	scaffoldA02	9794469	9894468	100000	1316
BnaWGS-1007	BnaA02G0156900ZS	scaffoldA02	9804469	9904468	100000	1301
BnaWGS-1007	BnaA02G0156900ZS	scaffoldA02	9814469	9914468	100000	1328
BnaWGS-1007	BnaA02G0156900ZS	scaffoldA02	9824469	9924468	100000	1346
BnaWGS-1007	BnaA02G0156900ZS	scaffoldA02	9834469	9934468	100000	1303
BnaWGS-1007	BnaA02G0156900ZS	scaffoldA02	9844469	9944468	100000	1366
BnaWGS-1007	BnaA02G0156900ZS	scaffoldA02	9854469	9954468	100000	1299
BnaWGS-1007	BnaA02G0156900ZS	scaffoldA02	9864469	9964468	100000	1210
BnaWGS-1007	BnaA02G0156900ZS	scaffoldA02	9874469	9974468	100000	1185
BnaWGS-1007	BnaA02G0156900ZS	scaffoldA02	9884469	9984468	100000	1061
BnaWGS-1007	BnaA02G0156900ZS	scaffoldA02	9894469	9994468	100000	1110
BnaWGS-1007	BnaA02G0156900ZS	scaffoldA02	9904469	10004468	100000	1145
BnaWGS-1007	BnaA02G0156900ZS	scaffoldA02	9914469	10014468	100000	1181
BnaWGS-1007	BnaA02G0156900ZS	scaffoldA02	9924469	10024468	100000	1077
BnaWGS-1007	BnaA02G0156900ZS	scaffoldA02	9934469	10034468	100000	1104
BnaWGS-1007	BnaA02G0156900ZS	scaffoldA02	9944469	10044468	100000	1152
BnaWGS-1007	BnaA02G0156900ZS	scaffoldA02	9954469	10054468	100000	1225
BnaWGS-1007	BnaA02G0156900ZS	scaffoldA02	9964469	10064468	100000	1247
BnaWGS-1007	BnaA02G0156900ZS	scaffoldA02	9974469	10074468	100000	1139
BnaWGS-1007	BnaA02G0156900ZS	scaffoldA02	9984469	10084468	100000	1125
BnaWGS-1007	BnaA02G0156900ZS	scaffoldA02	9994469	10094468	100000	1046
BnaWGS-1007	BnaA02G0156900ZS	scaffoldA02	10004469	10104468	100000	1001
BnaWGS-1007	BnaA02G0156900ZS	scaffoldA02	10014469	10107241	92773	823
BnaWGS-655	BnaA02G0156900ZS	scaffoldA02	8104469	8204468	100000	1766
BnaWGS-655	BnaA02G0156900ZS	scaffoldA02	8114469	8214468	100000	1813
BnaWGS-655	BnaA02G0156900ZS	scaffoldA02	8124469	8224468	100000	1798
BnaWGS-655	BnaA02G0156900ZS	scaffoldA02	8134469	8234468	100000	1622
BnaWGS-655	BnaA02G0156900ZS	scaffoldA02	8144469	8244468	100000	1713
BnaWGS-655	BnaA02G0156900ZS	scaffoldA02	8154469	8254468	100000	1718
BnaWGS-655	BnaA02G0156900ZS	scaffoldA02	8164469	8264468	100000	1631
BnaWGS-655	BnaA02G0156900ZS	scaffoldA02	8174469	8274468	100000	1578
BnaWGS-655	BnaA02G0156900ZS	scaffoldA02	8184469	8284468	100000	1610
BnaWGS-655	BnaA02G0156900ZS	scaffoldA02	8194469	8294468	100000	1535
BnaWGS-655	BnaA02G0156900ZS	scaffoldA02	8204469	8304468	100000	1529
BnaWGS-655	BnaA02G0156900ZS	scaffoldA02	8214469	8314468	100000	1385
BnaWGS-655	BnaA02G0156900ZS	scaffoldA02	8224469	8324468	100000	1370
BnaWGS-655	BnaA02G0156900ZS	scaffoldA02	8234469	8334468	100000	1396
BnaWGS-655	BnaA02G0156900ZS	scaffoldA02	8244469	8344468	100000	1254
BnaWGS-655	BnaA02G0156900ZS	scaffoldA02	8254469	8354468	100000	1147
BnaWGS-655	BnaA02G0156900ZS	scaffoldA02	8264469	8364468	100000	1214
BnaWGS-655	BnaA02G0156900ZS	scaffoldA02	8274469	8374468	100000	1226
BnaWGS-655	BnaA02G0156900ZS	scaffoldA02	8284469	8384468	100000	1203
BnaWGS-655	BnaA02G0156900ZS	scaffoldA02	8294469	8394468	100000	1158
BnaWGS-655	BnaA02G0156900ZS	scaffoldA02	8304469	8404468	100000	1085
BnaWGS-655	BnaA02G0156900ZS	scaffoldA02	8314469	8414468	100000	1076

For Review Only

BnaWGS-655	BnaA02G0156900ZS	scaffoldA02	8324469	8424468	100000	1113
BnaWGS-655	BnaA02G0156900ZS	scaffoldA02	8334469	8434468	100000	1104
BnaWGS-655	BnaA02G0156900ZS	scaffoldA02	8344469	8444468	100000	1003
BnaWGS-655	BnaA02G0156900ZS	scaffoldA02	8354469	8454468	100000	981
BnaWGS-655	BnaA02G0156900ZS	scaffoldA02	8364469	8464468	100000	793
BnaWGS-655	BnaA02G0156900ZS	scaffoldA02	8374469	8474468	100000	680
BnaWGS-655	BnaA02G0156900ZS	scaffoldA02	8384469	8484468	100000	593
BnaWGS-655	BnaA02G0156900ZS	scaffoldA02	8394469	8494468	100000	557
BnaWGS-655	BnaA02G0156900ZS	scaffoldA02	8404469	8504468	100000	538
BnaWGS-655	BnaA02G0156900ZS	scaffoldA02	8414469	8514468	100000	436
BnaWGS-655	BnaA02G0156900ZS	scaffoldA02	8424469	8524468	100000	258
BnaWGS-655	BnaA02G0156900ZS	scaffoldA02	8434469	8534468	100000	163
BnaWGS-655	BnaA02G0156900ZS	scaffoldA02	8444469	8544468	100000	120
BnaWGS-655	BnaA02G0156900ZS	scaffoldA02	8454469	8554468	100000	72
BnaWGS-655	BnaA02G0156900ZS	scaffoldA02	8464469	8564468	100000	159
BnaWGS-655	BnaA02G0156900ZS	scaffoldA02	8474469	8574468	100000	272
BnaWGS-655	BnaA02G0156900ZS	scaffoldA02	8484469	8584468	100000	317
BnaWGS-655	BnaA02G0156900ZS	scaffoldA02	8494469	8594468	100000	355
BnaWGS-655	BnaA02G0156900ZS	scaffoldA02	8504469	8604468	100000	536
BnaWGS-655	BnaA02G0156900ZS	scaffoldA02	8514469	8614468	100000	763
BnaWGS-655	BnaA02G0156900ZS	scaffoldA02	8524469	8624468	100000	790
BnaWGS-655	BnaA02G0156900ZS	scaffoldA02	8534469	8634468	100000	910
BnaWGS-655	BnaA02G0156900ZS	scaffoldA02	8544469	8644468	100000	996
BnaWGS-655	BnaA02G0156900ZS	scaffoldA02	8554469	8654468	100000	1156
BnaWGS-655	BnaA02G0156900ZS	scaffoldA02	8564469	8664468	100000	1195
BnaWGS-655	BnaA02G0156900ZS	scaffoldA02	8574469	8674468	100000	1210
BnaWGS-655	BnaA02G0156900ZS	scaffoldA02	8584469	8684468	100000	1214
BnaWGS-655	BnaA02G0156900ZS	scaffoldA02	8594469	8694468	100000	1330
BnaWGS-655	BnaA02G0156900ZS	scaffoldA02	8604469	8704468	100000	1343
BnaWGS-655	BnaA02G0156900ZS	scaffoldA02	8614469	8714468	100000	1335
BnaWGS-655	BnaA02G0156900ZS	scaffoldA02	8624469	8724468	100000	1524
BnaWGS-655	BnaA02G0156900ZS	scaffoldA02	8634469	8734468	100000	1587
BnaWGS-655	BnaA02G0156900ZS	scaffoldA02	8644469	8744468	100000	1581
BnaWGS-655	BnaA02G0156900ZS	scaffoldA02	8654469	8754468	100000	1427
BnaWGS-655	BnaA02G0156900ZS	scaffoldA02	8664469	8764468	100000	1337
BnaWGS-655	BnaA02G0156900ZS	scaffoldA02	8674469	8774468	100000	1303
BnaWGS-655	BnaA02G0156900ZS	scaffoldA02	8684469	8784468	100000	1403
BnaWGS-655	BnaA02G0156900ZS	scaffoldA02	8694469	8794468	100000	1395
BnaWGS-655	BnaA02G0156900ZS	scaffoldA02	8704469	8804468	100000	1347
BnaWGS-655	BnaA02G0156900ZS	scaffoldA02	8714469	8814468	100000	1188
BnaWGS-655	BnaA02G0156900ZS	scaffoldA02	8724469	8824468	100000	1162
BnaWGS-655	BnaA02G0156900ZS	scaffoldA02	8734469	8834468	100000	1325
BnaWGS-655	BnaA02G0156900ZS	scaffoldA02	8744469	8844468	100000	1321
BnaWGS-655	BnaA02G0156900ZS	scaffoldA02	8754469	8854468	100000	1301
BnaWGS-655	BnaA02G0156900ZS	scaffoldA02	8764469	8864468	100000	1327
BnaWGS-655	BnaA02G0156900ZS	scaffoldA02	8774469	8874468	100000	1391
BnaWGS-655	BnaA02G0156900ZS	scaffoldA02	8784469	8884468	100000	1300
BnaWGS-655	BnaA02G0156900ZS	scaffoldA02	8794469	8894468	100000	1212
BnaWGS-655	BnaA02G0156900ZS	scaffoldA02	8804469	8904468	100000	1231
BnaWGS-655	BnaA02G0156900ZS	scaffoldA02	8814469	8914468	100000	1460
BnaWGS-655	BnaA02G0156900ZS	scaffoldA02	8824469	8924468	100000	1404
BnaWGS-655	BnaA02G0156900ZS	scaffoldA02	8834469	8934468	100000	1313
BnaWGS-655	BnaA02G0156900ZS	scaffoldA02	8844469	8944468	100000	1532
BnaWGS-655	BnaA02G0156900ZS	scaffoldA02	8854469	8954468	100000	1747
BnaWGS-655	BnaA02G0156900ZS	scaffoldA02	8864469	8964468	100000	1898
BnaWGS-655	BnaA02G0156900ZS	scaffoldA02	8874469	8974468	100000	1792
BnaWGS-655	BnaA02G0156900ZS	scaffoldA02	8884469	8984468	100000	2003
BnaWGS-655	BnaA02G0156900ZS	scaffoldA02	8894469	8994468	100000	2092
BnaWGS-655	BnaA02G0156900ZS	scaffoldA02	8904469	9004468	100000	2026
BnaWGS-655	BnaA02G0156900ZS	scaffoldA02	8914469	9014468	100000	1856

For Review Only

BnaWGS-655	BnaA02G0156900ZS	scaffoldA02	8924469	9024468	100000	1881
BnaWGS-655	BnaA02G0156900ZS	scaffoldA02	8934469	9034468	100000	1748
BnaWGS-655	BnaA02G0156900ZS	scaffoldA02	8944469	9044468	100000	1646
BnaWGS-655	BnaA02G0156900ZS	scaffoldA02	8954469	9054468	100000	1671
BnaWGS-655	BnaA02G0156900ZS	scaffoldA02	8964469	9064468	100000	1730
BnaWGS-655	BnaA02G0156900ZS	scaffoldA02	8974469	9074468	100000	2006
BnaWGS-655	BnaA02G0156900ZS	scaffoldA02	8984469	9084468	100000	1986
BnaWGS-655	BnaA02G0156900ZS	scaffoldA02	8994469	9094468	100000	1989
BnaWGS-655	BnaA02G0156900ZS	scaffoldA02	9004469	9104468	100000	1949
BnaWGS-655	BnaA02G0156900ZS	scaffoldA02	9014469	9114468	100000	1937
BnaWGS-655	BnaA02G0156900ZS	scaffoldA02	9024469	9124468	100000	1991
BnaWGS-655	BnaA02G0156900ZS	scaffoldA02	9034469	9134468	100000	2089
BnaWGS-655	BnaA02G0156900ZS	scaffoldA02	9044469	9144468	100000	2054
BnaWGS-655	BnaA02G0156900ZS	scaffoldA02	9054469	9154468	100000	1917
BnaWGS-655	BnaA02G0156900ZS	scaffoldA02	9064469	9164468	100000	1707
BnaWGS-655	BnaA02G0156900ZS	scaffoldA02	9074469	9174468	100000	1477
BnaWGS-655	BnaA02G0156900ZS	scaffoldA02	9084469	9184468	100000	1282
BnaWGS-655	BnaA02G0156900ZS	scaffoldA02	9094469	9194468	100000	1216
BnaWGS-655	BnaA02G0156900ZS	scaffoldA02	9104469	9204468	100000	1417
BnaWGS-655	BnaA02G0156900ZS	scaffoldA02	9114469	9214468	100000	1490
BnaWGS-655	BnaA02G0156900ZS	scaffoldA02	9124469	9224468	100000	1365
BnaWGS-655	BnaA02G0156900ZS	scaffoldA02	9134469	9234468	100000	1241
BnaWGS-655	BnaA02G0156900ZS	scaffoldA02	9144469	9244468	100000	1247
BnaWGS-655	BnaA02G0156900ZS	scaffoldA02	9154469	9254468	100000	1369
BnaWGS-655	BnaA02G0156900ZS	scaffoldA02	9164469	9264468	100000	1405
BnaWGS-655	BnaA02G0156900ZS	scaffoldA02	9174469	9274468	100000	1368
BnaWGS-655	BnaA02G0156900ZS	scaffoldA02	9184469	9284468	100000	1429
BnaWGS-655	BnaA02G0156900ZS	scaffoldA02	9194469	9294468	100000	1463
BnaWGS-655	BnaA02G0156900ZS	scaffoldA02	9204469	9304468	100000	1410
BnaWGS-655	BnaA02G0156900ZS	scaffoldA02	9214469	9314468	100000	1297
BnaWGS-655	BnaA02G0156900ZS	scaffoldA02	9224469	9324468	100000	1344
BnaWGS-655	BnaA02G0156900ZS	scaffoldA02	9234469	9334468	100000	1413
BnaWGS-655	BnaA02G0156900ZS	scaffoldA02	9244469	9344468	100000	1345
BnaWGS-655	BnaA02G0156900ZS	scaffoldA02	9254469	9354468	100000	1241
BnaWGS-655	BnaA02G0156900ZS	scaffoldA02	9264469	9364468	100000	1233
BnaWGS-655	BnaA02G0156900ZS	scaffoldA02	9274469	9374468	100000	1365
BnaWGS-655	BnaA02G0156900ZS	scaffoldA02	9284469	9384468	100000	1476
BnaWGS-655	BnaA02G0156900ZS	scaffoldA02	9294469	9394468	100000	1458
BnaWGS-655	BnaA02G0156900ZS	scaffoldA02	9304469	9404468	100000	1395
BnaWGS-655	BnaA02G0156900ZS	scaffoldA02	9314469	9414468	100000	1490
BnaWGS-655	BnaA02G0156900ZS	scaffoldA02	9324469	9424468	100000	1625
BnaWGS-655	BnaA02G0156900ZS	scaffoldA02	9334469	9434468	100000	1580
BnaWGS-655	BnaA02G0156900ZS	scaffoldA02	9344469	9444468	100000	1651
BnaWGS-655	BnaA02G0156900ZS	scaffoldA02	9354469	9454468	100000	1614
BnaWGS-655	BnaA02G0156900ZS	scaffoldA02	9364469	9464468	100000	1599
BnaWGS-655	BnaA02G0156900ZS	scaffoldA02	9374469	9474468	100000	1459
BnaWGS-655	BnaA02G0156900ZS	scaffoldA02	9384469	9484468	100000	1435
BnaWGS-655	BnaA02G0156900ZS	scaffoldA02	9394469	9494468	100000	1639
BnaWGS-655	BnaA02G0156900ZS	scaffoldA02	9404469	9504468	100000	1662
BnaWGS-655	BnaA02G0156900ZS	scaffoldA02	9414469	9514468	100000	1519
BnaWGS-655	BnaA02G0156900ZS	scaffoldA02	9424469	9524468	100000	1299
BnaWGS-655	BnaA02G0156900ZS	scaffoldA02	9434469	9534468	100000	1423
BnaWGS-655	BnaA02G0156900ZS	scaffoldA02	9444469	9544468	100000	1399
BnaWGS-655	BnaA02G0156900ZS	scaffoldA02	9454469	9554468	100000	1401
BnaWGS-655	BnaA02G0156900ZS	scaffoldA02	9464469	9564468	100000	1456
BnaWGS-655	BnaA02G0156900ZS	scaffoldA02	9474469	9574468	100000	1561
BnaWGS-655	BnaA02G0156900ZS	scaffoldA02	9484469	9584468	100000	1388
BnaWGS-655	BnaA02G0156900ZS	scaffoldA02	9494469	9594468	100000	1171
BnaWGS-655	BnaA02G0156900ZS	scaffoldA02	9504469	9604468	100000	1169
BnaWGS-655	BnaA02G0156900ZS	scaffoldA02	9514469	9614468	100000	1162

For Review Only

BnaWGS-655	BnaA02G0156900ZS	scaffoldA02	9524469	9624468	100000	1138
BnaWGS-655	BnaA02G0156900ZS	scaffoldA02	9534469	9634468	100000	943
BnaWGS-655	BnaA02G0156900ZS	scaffoldA02	9544469	9644468	100000	897
BnaWGS-655	BnaA02G0156900ZS	scaffoldA02	9554469	9654468	100000	988
BnaWGS-655	BnaA02G0156900ZS	scaffoldA02	9564469	9664468	100000	920
BnaWGS-655	BnaA02G0156900ZS	scaffoldA02	9574469	9674468	100000	801
BnaWGS-655	BnaA02G0156900ZS	scaffoldA02	9584469	9684468	100000	833
BnaWGS-655	BnaA02G0156900ZS	scaffoldA02	9594469	9694468	100000	844
BnaWGS-655	BnaA02G0156900ZS	scaffoldA02	9604469	9704468	100000	811
BnaWGS-655	BnaA02G0156900ZS	scaffoldA02	9614469	9714468	100000	981
BnaWGS-655	BnaA02G0156900ZS	scaffoldA02	9624469	9724468	100000	1032
BnaWGS-655	BnaA02G0156900ZS	scaffoldA02	9634469	9734468	100000	1238
BnaWGS-655	BnaA02G0156900ZS	scaffoldA02	9644469	9744468	100000	1176
BnaWGS-655	BnaA02G0156900ZS	scaffoldA02	9654469	9754468	100000	988
BnaWGS-655	BnaA02G0156900ZS	scaffoldA02	9664469	9764468	100000	943
BnaWGS-655	BnaA02G0156900ZS	scaffoldA02	9674469	9774468	100000	1107
BnaWGS-655	BnaA02G0156900ZS	scaffoldA02	9684469	9784468	100000	1219
BnaWGS-655	BnaA02G0156900ZS	scaffoldA02	9694469	9794468	100000	1279
BnaWGS-655	BnaA02G0156900ZS	scaffoldA02	9704469	9804468	100000	1364
BnaWGS-655	BnaA02G0156900ZS	scaffoldA02	9714469	9814468	100000	1401
BnaWGS-655	BnaA02G0156900ZS	scaffoldA02	9724469	9824468	100000	1485
BnaWGS-655	BnaA02G0156900ZS	scaffoldA02	9734469	9834468	100000	1363
BnaWGS-655	BnaA02G0156900ZS	scaffoldA02	9744469	9844468	100000	1414
BnaWGS-655	BnaA02G0156900ZS	scaffoldA02	9754469	9854468	100000	1536
BnaWGS-655	BnaA02G0156900ZS	scaffoldA02	9764469	9864468	100000	1751
BnaWGS-655	BnaA02G0156900ZS	scaffoldA02	9774469	9874468	100000	1792
BnaWGS-655	BnaA02G0156900ZS	scaffoldA02	9784469	9884468	100000	1849
BnaWGS-655	BnaA02G0156900ZS	scaffoldA02	9794469	9894468	100000	1869
BnaWGS-655	BnaA02G0156900ZS	scaffoldA02	9804469	9904468	100000	1795
BnaWGS-655	BnaA02G0156900ZS	scaffoldA02	9814469	9914468	100000	1801
BnaWGS-655	BnaA02G0156900ZS	scaffoldA02	9824469	9924468	100000	1866
BnaWGS-655	BnaA02G0156900ZS	scaffoldA02	9834469	9934468	100000	1869
BnaWGS-655	BnaA02G0156900ZS	scaffoldA02	9844469	9944468	100000	1948
BnaWGS-655	BnaA02G0156900ZS	scaffoldA02	9854469	9954468	100000	1876
BnaWGS-655	BnaA02G0156900ZS	scaffoldA02	9864469	9964468	100000	1720
BnaWGS-655	BnaA02G0156900ZS	scaffoldA02	9874469	9974468	100000	1747
BnaWGS-655	BnaA02G0156900ZS	scaffoldA02	9884469	9984468	100000	1673
BnaWGS-655	BnaA02G0156900ZS	scaffoldA02	9894469	9994468	100000	1749
BnaWGS-655	BnaA02G0156900ZS	scaffoldA02	9904469	10004468	100000	1745
BnaWGS-655	BnaA02G0156900ZS	scaffoldA02	9914469	10014468	100000	1745
BnaWGS-655	BnaA02G0156900ZS	scaffoldA02	9924469	10024468	100000	1561
BnaWGS-655	BnaA02G0156900ZS	scaffoldA02	9934469	10034468	100000	1521
BnaWGS-655	BnaA02G0156900ZS	scaffoldA02	9944469	10044468	100000	1597
BnaWGS-655	BnaA02G0156900ZS	scaffoldA02	9954469	10054468	100000	1800
BnaWGS-655	BnaA02G0156900ZS	scaffoldA02	9964469	10064468	100000	1875
BnaWGS-655	BnaA02G0156900ZS	scaffoldA02	9974469	10074468	100000	1695
BnaWGS-655	BnaA02G0156900ZS	scaffoldA02	9984469	10084468	100000	1648
BnaWGS-655	BnaA02G0156900ZS	scaffoldA02	9994469	10094468	100000	1536
BnaWGS-655	BnaA02G0156900ZS	scaffoldA02	10004469	10104468	100000	1483
BnaWGS-655	BnaA02G0156900ZS	scaffoldA02	10014469	10107241	92773	1313
BnaWGS-655	BnaA02G0156900ZS	scaffoldA02	8104469	8204468	100000	1766
BnaWGS-655	BnaA02G0156900ZS	scaffoldA02	8114469	8214468	100000	1813
BnaWGS-655	BnaA02G0156900ZS	scaffoldA02	8124469	8224468	100000	1798
BnaWGS-655	BnaA02G0156900ZS	scaffoldA02	8134469	8234468	100000	1622
BnaWGS-655	BnaA02G0156900ZS	scaffoldA02	8144469	8244468	100000	1713
BnaWGS-655	BnaA02G0156900ZS	scaffoldA02	8154469	8254468	100000	1718
BnaWGS-655	BnaA02G0156900ZS	scaffoldA02	8164469	8264468	100000	1631
BnaWGS-655	BnaA02G0156900ZS	scaffoldA02	8174469	8274468	100000	1578
BnaWGS-655	BnaA02G0156900ZS	scaffoldA02	8184469	8284468	100000	1610
BnaWGS-655	BnaA02G0156900ZS	scaffoldA02	8194469	8294468	100000	1535

For Review Only

BnaWGS-655	BnaA02G0156900ZS	scaffoldA02	8204469	8304468	100000	1529
BnaWGS-655	BnaA02G0156900ZS	scaffoldA02	8214469	8314468	100000	1385
BnaWGS-655	BnaA02G0156900ZS	scaffoldA02	8224469	8324468	100000	1370
BnaWGS-655	BnaA02G0156900ZS	scaffoldA02	8234469	8334468	100000	1396
BnaWGS-655	BnaA02G0156900ZS	scaffoldA02	8244469	8344468	100000	1254
BnaWGS-655	BnaA02G0156900ZS	scaffoldA02	8254469	8354468	100000	1147
BnaWGS-655	BnaA02G0156900ZS	scaffoldA02	8264469	8364468	100000	1214
BnaWGS-655	BnaA02G0156900ZS	scaffoldA02	8274469	8374468	100000	1226
BnaWGS-655	BnaA02G0156900ZS	scaffoldA02	8284469	8384468	100000	1203
BnaWGS-655	BnaA02G0156900ZS	scaffoldA02	8294469	8394468	100000	1158
BnaWGS-655	BnaA02G0156900ZS	scaffoldA02	8304469	8404468	100000	1085
BnaWGS-655	BnaA02G0156900ZS	scaffoldA02	8314469	8414468	100000	1076
BnaWGS-655	BnaA02G0156900ZS	scaffoldA02	8324469	8424468	100000	1113
BnaWGS-655	BnaA02G0156900ZS	scaffoldA02	8334469	8434468	100000	1104
BnaWGS-655	BnaA02G0156900ZS	scaffoldA02	8344469	8444468	100000	1003
BnaWGS-655	BnaA02G0156900ZS	scaffoldA02	8354469	8454468	100000	981
BnaWGS-655	BnaA02G0156900ZS	scaffoldA02	8364469	8464468	100000	793
BnaWGS-655	BnaA02G0156900ZS	scaffoldA02	8374469	8474468	100000	680
BnaWGS-655	BnaA02G0156900ZS	scaffoldA02	8384469	8484468	100000	593
BnaWGS-655	BnaA02G0156900ZS	scaffoldA02	8394469	8494468	100000	557
BnaWGS-655	BnaA02G0156900ZS	scaffoldA02	8404469	8504468	100000	538
BnaWGS-655	BnaA02G0156900ZS	scaffoldA02	8414469	8514468	100000	436
BnaWGS-655	BnaA02G0156900ZS	scaffoldA02	8424469	8524468	100000	258
BnaWGS-655	BnaA02G0156900ZS	scaffoldA02	8434469	8534468	100000	163
BnaWGS-655	BnaA02G0156900ZS	scaffoldA02	8444469	8544468	100000	120
BnaWGS-655	BnaA02G0156900ZS	scaffoldA02	8454469	8554468	100000	72
BnaWGS-655	BnaA02G0156900ZS	scaffoldA02	8464469	8564468	100000	159
BnaWGS-655	BnaA02G0156900ZS	scaffoldA02	8474469	8574468	100000	272
BnaWGS-655	BnaA02G0156900ZS	scaffoldA02	8484469	8584468	100000	317
BnaWGS-655	BnaA02G0156900ZS	scaffoldA02	8494469	8594468	100000	355
BnaWGS-655	BnaA02G0156900ZS	scaffoldA02	8504469	8604468	100000	536
BnaWGS-655	BnaA02G0156900ZS	scaffoldA02	8514469	8614468	100000	763
BnaWGS-655	BnaA02G0156900ZS	scaffoldA02	8524469	8624468	100000	790
BnaWGS-655	BnaA02G0156900ZS	scaffoldA02	8534469	8634468	100000	910
BnaWGS-655	BnaA02G0156900ZS	scaffoldA02	8544469	8644468	100000	996
BnaWGS-655	BnaA02G0156900ZS	scaffoldA02	8554469	8654468	100000	1156
BnaWGS-655	BnaA02G0156900ZS	scaffoldA02	8564469	8664468	100000	1195
BnaWGS-655	BnaA02G0156900ZS	scaffoldA02	8574469	8674468	100000	1210
BnaWGS-655	BnaA02G0156900ZS	scaffoldA02	8584469	8684468	100000	1214
BnaWGS-655	BnaA02G0156900ZS	scaffoldA02	8594469	8694468	100000	1330
BnaWGS-655	BnaA02G0156900ZS	scaffoldA02	8604469	8704468	100000	1343
BnaWGS-655	BnaA02G0156900ZS	scaffoldA02	8614469	8714468	100000	1335
BnaWGS-655	BnaA02G0156900ZS	scaffoldA02	8624469	8724468	100000	1524
BnaWGS-655	BnaA02G0156900ZS	scaffoldA02	8634469	8734468	100000	1587
BnaWGS-655	BnaA02G0156900ZS	scaffoldA02	8644469	8744468	100000	1581
BnaWGS-655	BnaA02G0156900ZS	scaffoldA02	8654469	8754468	100000	1427
BnaWGS-655	BnaA02G0156900ZS	scaffoldA02	8664469	8764468	100000	1337
BnaWGS-655	BnaA02G0156900ZS	scaffoldA02	8674469	8774468	100000	1303
BnaWGS-655	BnaA02G0156900ZS	scaffoldA02	8684469	8784468	100000	1403
BnaWGS-655	BnaA02G0156900ZS	scaffoldA02	8694469	8794468	100000	1395
BnaWGS-655	BnaA02G0156900ZS	scaffoldA02	8704469	8804468	100000	1347
BnaWGS-655	BnaA02G0156900ZS	scaffoldA02	8714469	8814468	100000	1188
BnaWGS-655	BnaA02G0156900ZS	scaffoldA02	8724469	8824468	100000	1162
BnaWGS-655	BnaA02G0156900ZS	scaffoldA02	8734469	8834468	100000	1325
BnaWGS-655	BnaA02G0156900ZS	scaffoldA02	8744469	8844468	100000	1321
BnaWGS-655	BnaA02G0156900ZS	scaffoldA02	8754469	8854468	100000	1301
BnaWGS-655	BnaA02G0156900ZS	scaffoldA02	8764469	8864468	100000	1327
BnaWGS-655	BnaA02G0156900ZS	scaffoldA02	8774469	8874468	100000	1391
BnaWGS-655	BnaA02G0156900ZS	scaffoldA02	8784469	8884468	100000	1300
BnaWGS-655	BnaA02G0156900ZS	scaffoldA02	8794469	8894468	100000	1212

For Review Only

BnaWGS-655	BnaA02G0156900ZS	scaffoldA02	8804469	8904468	100000	1231
BnaWGS-655	BnaA02G0156900ZS	scaffoldA02	8814469	8914468	100000	1460
BnaWGS-655	BnaA02G0156900ZS	scaffoldA02	8824469	8924468	100000	1404
BnaWGS-655	BnaA02G0156900ZS	scaffoldA02	8834469	8934468	100000	1313
BnaWGS-655	BnaA02G0156900ZS	scaffoldA02	8844469	8944468	100000	1532
BnaWGS-655	BnaA02G0156900ZS	scaffoldA02	8854469	8954468	100000	1747
BnaWGS-655	BnaA02G0156900ZS	scaffoldA02	8864469	8964468	100000	1898
BnaWGS-655	BnaA02G0156900ZS	scaffoldA02	8874469	8974468	100000	1792
BnaWGS-655	BnaA02G0156900ZS	scaffoldA02	8884469	8984468	100000	2003
BnaWGS-655	BnaA02G0156900ZS	scaffoldA02	8894469	8994468	100000	2092
BnaWGS-655	BnaA02G0156900ZS	scaffoldA02	8904469	9004468	100000	2026
BnaWGS-655	BnaA02G0156900ZS	scaffoldA02	8914469	9014468	100000	1856
BnaWGS-655	BnaA02G0156900ZS	scaffoldA02	8924469	9024468	100000	1881
BnaWGS-655	BnaA02G0156900ZS	scaffoldA02	8934469	9034468	100000	1748
BnaWGS-655	BnaA02G0156900ZS	scaffoldA02	8944469	9044468	100000	1646
BnaWGS-655	BnaA02G0156900ZS	scaffoldA02	8954469	9054468	100000	1671
BnaWGS-655	BnaA02G0156900ZS	scaffoldA02	8964469	9064468	100000	1730
BnaWGS-655	BnaA02G0156900ZS	scaffoldA02	8974469	9074468	100000	2006
BnaWGS-655	BnaA02G0156900ZS	scaffoldA02	8984469	9084468	100000	1986
BnaWGS-655	BnaA02G0156900ZS	scaffoldA02	8994469	9094468	100000	1989
BnaWGS-655	BnaA02G0156900ZS	scaffoldA02	9004469	9104468	100000	1949
BnaWGS-655	BnaA02G0156900ZS	scaffoldA02	9014469	9114468	100000	1937
BnaWGS-655	BnaA02G0156900ZS	scaffoldA02	9024469	9124468	100000	1991
BnaWGS-655	BnaA02G0156900ZS	scaffoldA02	9034469	9134468	100000	2089
BnaWGS-655	BnaA02G0156900ZS	scaffoldA02	9044469	9144468	100000	2054
BnaWGS-655	BnaA02G0156900ZS	scaffoldA02	9054469	9154468	100000	1917
BnaWGS-655	BnaA02G0156900ZS	scaffoldA02	9064469	9164468	100000	1707
BnaWGS-655	BnaA02G0156900ZS	scaffoldA02	9074469	9174468	100000	1477
BnaWGS-655	BnaA02G0156900ZS	scaffoldA02	9084469	9184468	100000	1282
BnaWGS-655	BnaA02G0156900ZS	scaffoldA02	9094469	9194468	100000	1216
BnaWGS-655	BnaA02G0156900ZS	scaffoldA02	9104469	9204468	100000	1417
BnaWGS-655	BnaA02G0156900ZS	scaffoldA02	9114469	9214468	100000	1490
BnaWGS-655	BnaA02G0156900ZS	scaffoldA02	9124469	9224468	100000	1365
BnaWGS-655	BnaA02G0156900ZS	scaffoldA02	9134469	9234468	100000	1241
BnaWGS-655	BnaA02G0156900ZS	scaffoldA02	9144469	9244468	100000	1247
BnaWGS-655	BnaA02G0156900ZS	scaffoldA02	9154469	9254468	100000	1369
BnaWGS-655	BnaA02G0156900ZS	scaffoldA02	9164469	9264468	100000	1405
BnaWGS-655	BnaA02G0156900ZS	scaffoldA02	9174469	9274468	100000	1368
BnaWGS-655	BnaA02G0156900ZS	scaffoldA02	9184469	9284468	100000	1429
BnaWGS-655	BnaA02G0156900ZS	scaffoldA02	9194469	9294468	100000	1463
BnaWGS-655	BnaA02G0156900ZS	scaffoldA02	9204469	9304468	100000	1410
BnaWGS-655	BnaA02G0156900ZS	scaffoldA02	9214469	9314468	100000	1297
BnaWGS-655	BnaA02G0156900ZS	scaffoldA02	9224469	9324468	100000	1344
BnaWGS-655	BnaA02G0156900ZS	scaffoldA02	9234469	9334468	100000	1413
BnaWGS-655	BnaA02G0156900ZS	scaffoldA02	9244469	9344468	100000	1345
BnaWGS-655	BnaA02G0156900ZS	scaffoldA02	9254469	9354468	100000	1241
BnaWGS-655	BnaA02G0156900ZS	scaffoldA02	9264469	9364468	100000	1233
BnaWGS-655	BnaA02G0156900ZS	scaffoldA02	9274469	9374468	100000	1365
BnaWGS-655	BnaA02G0156900ZS	scaffoldA02	9284469	9384468	100000	1476
BnaWGS-655	BnaA02G0156900ZS	scaffoldA02	9294469	9394468	100000	1458
BnaWGS-655	BnaA02G0156900ZS	scaffoldA02	9304469	9404468	100000	1395
BnaWGS-655	BnaA02G0156900ZS	scaffoldA02	9314469	9414468	100000	1490
BnaWGS-655	BnaA02G0156900ZS	scaffoldA02	9324469	9424468	100000	1625
BnaWGS-655	BnaA02G0156900ZS	scaffoldA02	9334469	9434468	100000	1580
BnaWGS-655	BnaA02G0156900ZS	scaffoldA02	9344469	9444468	100000	1651
BnaWGS-655	BnaA02G0156900ZS	scaffoldA02	9354469	9454468	100000	1614
BnaWGS-655	BnaA02G0156900ZS	scaffoldA02	9364469	9464468	100000	1599
BnaWGS-655	BnaA02G0156900ZS	scaffoldA02	9374469	9474468	100000	1459
BnaWGS-655	BnaA02G0156900ZS	scaffoldA02	9384469	9484468	100000	1435
BnaWGS-655	BnaA02G0156900ZS	scaffoldA02	9394469	9494468	100000	1639

For Review Only

BnaWGS-655	BnaA02G0156900ZS	scaffoldA02	9404469	9504468	100000	1662
BnaWGS-655	BnaA02G0156900ZS	scaffoldA02	9414469	9514468	100000	1519
BnaWGS-655	BnaA02G0156900ZS	scaffoldA02	9424469	9524468	100000	1299
BnaWGS-655	BnaA02G0156900ZS	scaffoldA02	9434469	9534468	100000	1423
BnaWGS-655	BnaA02G0156900ZS	scaffoldA02	9444469	9544468	100000	1399
BnaWGS-655	BnaA02G0156900ZS	scaffoldA02	9454469	9554468	100000	1401
BnaWGS-655	BnaA02G0156900ZS	scaffoldA02	9464469	9564468	100000	1456
BnaWGS-655	BnaA02G0156900ZS	scaffoldA02	9474469	9574468	100000	1561
BnaWGS-655	BnaA02G0156900ZS	scaffoldA02	9484469	9584468	100000	1388
BnaWGS-655	BnaA02G0156900ZS	scaffoldA02	9494469	9594468	100000	1171
BnaWGS-655	BnaA02G0156900ZS	scaffoldA02	9504469	9604468	100000	1169
BnaWGS-655	BnaA02G0156900ZS	scaffoldA02	9514469	9614468	100000	1162
BnaWGS-655	BnaA02G0156900ZS	scaffoldA02	9524469	9624468	100000	1138
BnaWGS-655	BnaA02G0156900ZS	scaffoldA02	9534469	9634468	100000	943
BnaWGS-655	BnaA02G0156900ZS	scaffoldA02	9544469	9644468	100000	897
BnaWGS-655	BnaA02G0156900ZS	scaffoldA02	9554469	9654468	100000	988
BnaWGS-655	BnaA02G0156900ZS	scaffoldA02	9564469	9664468	100000	920
BnaWGS-655	BnaA02G0156900ZS	scaffoldA02	9574469	9674468	100000	801
BnaWGS-655	BnaA02G0156900ZS	scaffoldA02	9584469	9684468	100000	833
BnaWGS-655	BnaA02G0156900ZS	scaffoldA02	9594469	9694468	100000	844
BnaWGS-655	BnaA02G0156900ZS	scaffoldA02	9604469	9704468	100000	811
BnaWGS-655	BnaA02G0156900ZS	scaffoldA02	9614469	9714468	100000	981
BnaWGS-655	BnaA02G0156900ZS	scaffoldA02	9624469	9724468	100000	1032
BnaWGS-655	BnaA02G0156900ZS	scaffoldA02	9634469	9734468	100000	1238
BnaWGS-655	BnaA02G0156900ZS	scaffoldA02	9644469	9744468	100000	1176
BnaWGS-655	BnaA02G0156900ZS	scaffoldA02	9654469	9754468	100000	988
BnaWGS-655	BnaA02G0156900ZS	scaffoldA02	9664469	9764468	100000	943
BnaWGS-655	BnaA02G0156900ZS	scaffoldA02	9674469	9774468	100000	1107
BnaWGS-655	BnaA02G0156900ZS	scaffoldA02	9684469	9784468	100000	1219
BnaWGS-655	BnaA02G0156900ZS	scaffoldA02	9694469	9794468	100000	1279
BnaWGS-655	BnaA02G0156900ZS	scaffoldA02	9704469	9804468	100000	1364
BnaWGS-655	BnaA02G0156900ZS	scaffoldA02	9714469	9814468	100000	1401
BnaWGS-655	BnaA02G0156900ZS	scaffoldA02	9724469	9824468	100000	1485
BnaWGS-655	BnaA02G0156900ZS	scaffoldA02	9734469	9834468	100000	1363
BnaWGS-655	BnaA02G0156900ZS	scaffoldA02	9744469	9844468	100000	1414
BnaWGS-655	BnaA02G0156900ZS	scaffoldA02	9754469	9854468	100000	1536
BnaWGS-655	BnaA02G0156900ZS	scaffoldA02	9764469	9864468	100000	1751
BnaWGS-655	BnaA02G0156900ZS	scaffoldA02	9774469	9874468	100000	1792
BnaWGS-655	BnaA02G0156900ZS	scaffoldA02	9784469	9884468	100000	1849
BnaWGS-655	BnaA02G0156900ZS	scaffoldA02	9794469	9894468	100000	1869
BnaWGS-655	BnaA02G0156900ZS	scaffoldA02	9804469	9904468	100000	1795
BnaWGS-655	BnaA02G0156900ZS	scaffoldA02	9814469	9914468	100000	1801
BnaWGS-655	BnaA02G0156900ZS	scaffoldA02	9824469	9924468	100000	1866
BnaWGS-655	BnaA02G0156900ZS	scaffoldA02	9834469	9934468	100000	1869
BnaWGS-655	BnaA02G0156900ZS	scaffoldA02	9844469	9944468	100000	1948
BnaWGS-655	BnaA02G0156900ZS	scaffoldA02	9854469	9954468	100000	1876
BnaWGS-655	BnaA02G0156900ZS	scaffoldA02	9864469	9964468	100000	1720
BnaWGS-655	BnaA02G0156900ZS	scaffoldA02	9874469	9974468	100000	1747
BnaWGS-655	BnaA02G0156900ZS	scaffoldA02	9884469	9984468	100000	1673
BnaWGS-655	BnaA02G0156900ZS	scaffoldA02	9894469	9994468	100000	1749
BnaWGS-655	BnaA02G0156900ZS	scaffoldA02	9904469	10004468	100000	1745
BnaWGS-655	BnaA02G0156900ZS	scaffoldA02	9914469	10014468	100000	1745
BnaWGS-655	BnaA02G0156900ZS	scaffoldA02	9924469	10024468	100000	1561
BnaWGS-655	BnaA02G0156900ZS	scaffoldA02	9934469	10034468	100000	1521
BnaWGS-655	BnaA02G0156900ZS	scaffoldA02	9944469	10044468	100000	1597
BnaWGS-655	BnaA02G0156900ZS	scaffoldA02	9954469	10054468	100000	1800
BnaWGS-655	BnaA02G0156900ZS	scaffoldA02	9964469	10064468	100000	1875
BnaWGS-655	BnaA02G0156900ZS	scaffoldA02	9974469	10074468	100000	1695
BnaWGS-655	BnaA02G0156900ZS	scaffoldA02	9984469	10084468	100000	1648
BnaWGS-655	BnaA02G0156900ZS	scaffoldA02	9994469	10094468	100000	1536

For Review Only

BnaWGS-655	BnaA02G0156900ZS	scaffoldA02	10004469	10104468	100000	1483
BnaWGS-655	BnaA02G0156900ZS	scaffoldA02	10014469	10107241	92773	1313
BnaWGS-655	BnaA02G0156900ZS	scaffoldA02	8104469	8204468	100000	1766
BnaWGS-655	BnaA02G0156900ZS	scaffoldA02	8114469	8214468	100000	1813
BnaWGS-655	BnaA02G0156900ZS	scaffoldA02	8124469	8224468	100000	1798
BnaWGS-655	BnaA02G0156900ZS	scaffoldA02	8134469	8234468	100000	1622
BnaWGS-655	BnaA02G0156900ZS	scaffoldA02	8144469	8244468	100000	1713
BnaWGS-655	BnaA02G0156900ZS	scaffoldA02	8154469	8254468	100000	1718
BnaWGS-655	BnaA02G0156900ZS	scaffoldA02	8164469	8264468	100000	1631
BnaWGS-655	BnaA02G0156900ZS	scaffoldA02	8174469	8274468	100000	1578
BnaWGS-655	BnaA02G0156900ZS	scaffoldA02	8184469	8284468	100000	1610
BnaWGS-655	BnaA02G0156900ZS	scaffoldA02	8194469	8294468	100000	1535
BnaWGS-655	BnaA02G0156900ZS	scaffoldA02	8204469	8304468	100000	1529
BnaWGS-655	BnaA02G0156900ZS	scaffoldA02	8214469	8314468	100000	1385
BnaWGS-655	BnaA02G0156900ZS	scaffoldA02	8224469	8324468	100000	1370
BnaWGS-655	BnaA02G0156900ZS	scaffoldA02	8234469	8334468	100000	1396
BnaWGS-655	BnaA02G0156900ZS	scaffoldA02	8244469	8344468	100000	1254
BnaWGS-655	BnaA02G0156900ZS	scaffoldA02	8254469	8354468	100000	1147
BnaWGS-655	BnaA02G0156900ZS	scaffoldA02	8264469	8364468	100000	1214
BnaWGS-655	BnaA02G0156900ZS	scaffoldA02	8274469	8374468	100000	1226
BnaWGS-655	BnaA02G0156900ZS	scaffoldA02	8284469	8384468	100000	1203
BnaWGS-655	BnaA02G0156900ZS	scaffoldA02	8294469	8394468	100000	1158
BnaWGS-655	BnaA02G0156900ZS	scaffoldA02	8304469	8404468	100000	1085
BnaWGS-655	BnaA02G0156900ZS	scaffoldA02	8314469	8414468	100000	1076
BnaWGS-655	BnaA02G0156900ZS	scaffoldA02	8324469	8424468	100000	1113
BnaWGS-655	BnaA02G0156900ZS	scaffoldA02	8334469	8434468	100000	1104
BnaWGS-655	BnaA02G0156900ZS	scaffoldA02	8344469	8444468	100000	1003
BnaWGS-655	BnaA02G0156900ZS	scaffoldA02	8354469	8454468	100000	981
BnaWGS-655	BnaA02G0156900ZS	scaffoldA02	8364469	8464468	100000	793
BnaWGS-655	BnaA02G0156900ZS	scaffoldA02	8374469	8474468	100000	680
BnaWGS-655	BnaA02G0156900ZS	scaffoldA02	8384469	8484468	100000	593
BnaWGS-655	BnaA02G0156900ZS	scaffoldA02	8394469	8494468	100000	557
BnaWGS-655	BnaA02G0156900ZS	scaffoldA02	8404469	8504468	100000	538
BnaWGS-655	BnaA02G0156900ZS	scaffoldA02	8414469	8514468	100000	436
BnaWGS-655	BnaA02G0156900ZS	scaffoldA02	8424469	8524468	100000	258
BnaWGS-655	BnaA02G0156900ZS	scaffoldA02	8434469	8534468	100000	163
BnaWGS-655	BnaA02G0156900ZS	scaffoldA02	8444469	8544468	100000	120
BnaWGS-655	BnaA02G0156900ZS	scaffoldA02	8454469	8554468	100000	72
BnaWGS-655	BnaA02G0156900ZS	scaffoldA02	8464469	8564468	100000	159
BnaWGS-655	BnaA02G0156900ZS	scaffoldA02	8474469	8574468	100000	272
BnaWGS-655	BnaA02G0156900ZS	scaffoldA02	8484469	8584468	100000	317
BnaWGS-655	BnaA02G0156900ZS	scaffoldA02	8494469	8594468	100000	355
BnaWGS-655	BnaA02G0156900ZS	scaffoldA02	8504469	8604468	100000	536
BnaWGS-655	BnaA02G0156900ZS	scaffoldA02	8514469	8614468	100000	763
BnaWGS-655	BnaA02G0156900ZS	scaffoldA02	8524469	8624468	100000	790
BnaWGS-655	BnaA02G0156900ZS	scaffoldA02	8534469	8634468	100000	910
BnaWGS-655	BnaA02G0156900ZS	scaffoldA02	8544469	8644468	100000	996
BnaWGS-655	BnaA02G0156900ZS	scaffoldA02	8554469	8654468	100000	1156
BnaWGS-655	BnaA02G0156900ZS	scaffoldA02	8564469	8664468	100000	1195
BnaWGS-655	BnaA02G0156900ZS	scaffoldA02	8574469	8674468	100000	1210
BnaWGS-655	BnaA02G0156900ZS	scaffoldA02	8584469	8684468	100000	1214
BnaWGS-655	BnaA02G0156900ZS	scaffoldA02	8594469	8694468	100000	1330
BnaWGS-655	BnaA02G0156900ZS	scaffoldA02	8604469	8704468	100000	1343
BnaWGS-655	BnaA02G0156900ZS	scaffoldA02	8614469	8714468	100000	1335
BnaWGS-655	BnaA02G0156900ZS	scaffoldA02	8624469	8724468	100000	1524
BnaWGS-655	BnaA02G0156900ZS	scaffoldA02	8634469	8734468	100000	1587
BnaWGS-655	BnaA02G0156900ZS	scaffoldA02	8644469	8744468	100000	1581
BnaWGS-655	BnaA02G0156900ZS	scaffoldA02	8654469	8754468	100000	1427
BnaWGS-655	BnaA02G0156900ZS	scaffoldA02	8664469	8764468	100000	1337
BnaWGS-655	BnaA02G0156900ZS	scaffoldA02	8674469	8774468	100000	1303

For Review Only

BnaWGS-655	BnaA02G0156900ZS	scaffoldA02	8684469	8784468	100000	1403
BnaWGS-655	BnaA02G0156900ZS	scaffoldA02	8694469	8794468	100000	1395
BnaWGS-655	BnaA02G0156900ZS	scaffoldA02	8704469	8804468	100000	1347
BnaWGS-655	BnaA02G0156900ZS	scaffoldA02	8714469	8814468	100000	1188
BnaWGS-655	BnaA02G0156900ZS	scaffoldA02	8724469	8824468	100000	1162
BnaWGS-655	BnaA02G0156900ZS	scaffoldA02	8734469	8834468	100000	1325
BnaWGS-655	BnaA02G0156900ZS	scaffoldA02	8744469	8844468	100000	1321
BnaWGS-655	BnaA02G0156900ZS	scaffoldA02	8754469	8854468	100000	1301
BnaWGS-655	BnaA02G0156900ZS	scaffoldA02	8764469	8864468	100000	1327
BnaWGS-655	BnaA02G0156900ZS	scaffoldA02	8774469	8874468	100000	1391
BnaWGS-655	BnaA02G0156900ZS	scaffoldA02	8784469	8884468	100000	1300
BnaWGS-655	BnaA02G0156900ZS	scaffoldA02	8794469	8894468	100000	1212
BnaWGS-655	BnaA02G0156900ZS	scaffoldA02	8804469	8904468	100000	1231
BnaWGS-655	BnaA02G0156900ZS	scaffoldA02	8814469	8914468	100000	1460
BnaWGS-655	BnaA02G0156900ZS	scaffoldA02	8824469	8924468	100000	1404
BnaWGS-655	BnaA02G0156900ZS	scaffoldA02	8834469	8934468	100000	1313
BnaWGS-655	BnaA02G0156900ZS	scaffoldA02	8844469	8944468	100000	1532
BnaWGS-655	BnaA02G0156900ZS	scaffoldA02	8854469	8954468	100000	1747
BnaWGS-655	BnaA02G0156900ZS	scaffoldA02	8864469	8964468	100000	1898
BnaWGS-655	BnaA02G0156900ZS	scaffoldA02	8874469	8974468	100000	1792
BnaWGS-655	BnaA02G0156900ZS	scaffoldA02	8884469	8984468	100000	2003
BnaWGS-655	BnaA02G0156900ZS	scaffoldA02	8894469	8994468	100000	2092
BnaWGS-655	BnaA02G0156900ZS	scaffoldA02	8904469	9004468	100000	2026
BnaWGS-655	BnaA02G0156900ZS	scaffoldA02	8914469	9014468	100000	1856
BnaWGS-655	BnaA02G0156900ZS	scaffoldA02	8924469	9024468	100000	1881
BnaWGS-655	BnaA02G0156900ZS	scaffoldA02	8934469	9034468	100000	1748
BnaWGS-655	BnaA02G0156900ZS	scaffoldA02	8944469	9044468	100000	1646
BnaWGS-655	BnaA02G0156900ZS	scaffoldA02	8954469	9054468	100000	1671
BnaWGS-655	BnaA02G0156900ZS	scaffoldA02	8964469	9064468	100000	1730
BnaWGS-655	BnaA02G0156900ZS	scaffoldA02	8974469	9074468	100000	2006
BnaWGS-655	BnaA02G0156900ZS	scaffoldA02	8984469	9084468	100000	1986
BnaWGS-655	BnaA02G0156900ZS	scaffoldA02	8994469	9094468	100000	1989
BnaWGS-655	BnaA02G0156900ZS	scaffoldA02	9004469	9104468	100000	1949
BnaWGS-655	BnaA02G0156900ZS	scaffoldA02	9014469	9114468	100000	1937
BnaWGS-655	BnaA02G0156900ZS	scaffoldA02	9024469	9124468	100000	1991
BnaWGS-655	BnaA02G0156900ZS	scaffoldA02	9034469	9134468	100000	2089
BnaWGS-655	BnaA02G0156900ZS	scaffoldA02	9044469	9144468	100000	2054
BnaWGS-655	BnaA02G0156900ZS	scaffoldA02	9054469	9154468	100000	1917
BnaWGS-655	BnaA02G0156900ZS	scaffoldA02	9064469	9164468	100000	1707
BnaWGS-655	BnaA02G0156900ZS	scaffoldA02	9074469	9174468	100000	1477
BnaWGS-655	BnaA02G0156900ZS	scaffoldA02	9084469	9184468	100000	1282
BnaWGS-655	BnaA02G0156900ZS	scaffoldA02	9094469	9194468	100000	1216
BnaWGS-655	BnaA02G0156900ZS	scaffoldA02	9104469	9204468	100000	1417
BnaWGS-655	BnaA02G0156900ZS	scaffoldA02	9114469	9214468	100000	1490
BnaWGS-655	BnaA02G0156900ZS	scaffoldA02	9124469	9224468	100000	1365
BnaWGS-655	BnaA02G0156900ZS	scaffoldA02	9134469	9234468	100000	1241
BnaWGS-655	BnaA02G0156900ZS	scaffoldA02	9144469	9244468	100000	1247
BnaWGS-655	BnaA02G0156900ZS	scaffoldA02	9154469	9254468	100000	1369
BnaWGS-655	BnaA02G0156900ZS	scaffoldA02	9164469	9264468	100000	1405
BnaWGS-655	BnaA02G0156900ZS	scaffoldA02	9174469	9274468	100000	1368
BnaWGS-655	BnaA02G0156900ZS	scaffoldA02	9184469	9284468	100000	1429
BnaWGS-655	BnaA02G0156900ZS	scaffoldA02	9194469	9294468	100000	1463
BnaWGS-655	BnaA02G0156900ZS	scaffoldA02	9204469	9304468	100000	1410
BnaWGS-655	BnaA02G0156900ZS	scaffoldA02	9214469	9314468	100000	1297
BnaWGS-655	BnaA02G0156900ZS	scaffoldA02	9224469	9324468	100000	1344
BnaWGS-655	BnaA02G0156900ZS	scaffoldA02	9234469	9334468	100000	1413
BnaWGS-655	BnaA02G0156900ZS	scaffoldA02	9244469	9344468	100000	1345
BnaWGS-655	BnaA02G0156900ZS	scaffoldA02	9254469	9354468	100000	1241
BnaWGS-655	BnaA02G0156900ZS	scaffoldA02	9264469	9364468	100000	1233
BnaWGS-655	BnaA02G0156900ZS	scaffoldA02	9274469	9374468	100000	1365

BnaWGS-655	BnaA02G0156900ZS	scaffoldA02	9284469	9384468	100000	1476
BnaWGS-655	BnaA02G0156900ZS	scaffoldA02	9294469	9394468	100000	1458
BnaWGS-655	BnaA02G0156900ZS	scaffoldA02	9304469	9404468	100000	1395
BnaWGS-655	BnaA02G0156900ZS	scaffoldA02	9314469	9414468	100000	1490
BnaWGS-655	BnaA02G0156900ZS	scaffoldA02	9324469	9424468	100000	1625
BnaWGS-655	BnaA02G0156900ZS	scaffoldA02	9334469	9434468	100000	1580
BnaWGS-655	BnaA02G0156900ZS	scaffoldA02	9344469	9444468	100000	1651
BnaWGS-655	BnaA02G0156900ZS	scaffoldA02	9354469	9454468	100000	1614
BnaWGS-655	BnaA02G0156900ZS	scaffoldA02	9364469	9464468	100000	1599
BnaWGS-655	BnaA02G0156900ZS	scaffoldA02	9374469	9474468	100000	1459
BnaWGS-655	BnaA02G0156900ZS	scaffoldA02	9384469	9484468	100000	1435
BnaWGS-655	BnaA02G0156900ZS	scaffoldA02	9394469	9494468	100000	1639
BnaWGS-655	BnaA02G0156900ZS	scaffoldA02	9404469	9504468	100000	1662
BnaWGS-655	BnaA02G0156900ZS	scaffoldA02	9414469	9514468	100000	1519
BnaWGS-655	BnaA02G0156900ZS	scaffoldA02	9424469	9524468	100000	1299
BnaWGS-655	BnaA02G0156900ZS	scaffoldA02	9434469	9534468	100000	1423
BnaWGS-655	BnaA02G0156900ZS	scaffoldA02	9444469	9544468	100000	1399
BnaWGS-655	BnaA02G0156900ZS	scaffoldA02	9454469	9554468	100000	1401
BnaWGS-655	BnaA02G0156900ZS	scaffoldA02	9464469	9564468	100000	1456
BnaWGS-655	BnaA02G0156900ZS	scaffoldA02	9474469	9574468	100000	1561
BnaWGS-655	BnaA02G0156900ZS	scaffoldA02	9484469	9584468	100000	1388
BnaWGS-655	BnaA02G0156900ZS	scaffoldA02	9494469	9594468	100000	1171
BnaWGS-655	BnaA02G0156900ZS	scaffoldA02	9504469	9604468	100000	1169
BnaWGS-655	BnaA02G0156900ZS	scaffoldA02	9514469	9614468	100000	1162
BnaWGS-655	BnaA02G0156900ZS	scaffoldA02	9524469	9624468	100000	1138
BnaWGS-655	BnaA02G0156900ZS	scaffoldA02	9534469	9634468	100000	943
BnaWGS-655	BnaA02G0156900ZS	scaffoldA02	9544469	9644468	100000	897
BnaWGS-655	BnaA02G0156900ZS	scaffoldA02	9554469	9654468	100000	988
BnaWGS-655	BnaA02G0156900ZS	scaffoldA02	9564469	9664468	100000	920
BnaWGS-655	BnaA02G0156900ZS	scaffoldA02	9574469	9674468	100000	801
BnaWGS-655	BnaA02G0156900ZS	scaffoldA02	9584469	9684468	100000	833
BnaWGS-655	BnaA02G0156900ZS	scaffoldA02	9594469	9694468	100000	844
BnaWGS-655	BnaA02G0156900ZS	scaffoldA02	9604469	9704468	100000	811
BnaWGS-655	BnaA02G0156900ZS	scaffoldA02	9614469	9714468	100000	981
BnaWGS-655	BnaA02G0156900ZS	scaffoldA02	9624469	9724468	100000	1032
BnaWGS-655	BnaA02G0156900ZS	scaffoldA02	9634469	9734468	100000	1238
BnaWGS-655	BnaA02G0156900ZS	scaffoldA02	9644469	9744468	100000	1176
BnaWGS-655	BnaA02G0156900ZS	scaffoldA02	9654469	9754468	100000	988
BnaWGS-655	BnaA02G0156900ZS	scaffoldA02	9664469	9764468	100000	943
BnaWGS-655	BnaA02G0156900ZS	scaffoldA02	9674469	9774468	100000	1107
BnaWGS-655	BnaA02G0156900ZS	scaffoldA02	9684469	9784468	100000	1219
BnaWGS-655	BnaA02G0156900ZS	scaffoldA02	9694469	9794468	100000	1279
BnaWGS-655	BnaA02G0156900ZS	scaffoldA02	9704469	9804468	100000	1364
BnaWGS-655	BnaA02G0156900ZS	scaffoldA02	9714469	9814468	100000	1401
BnaWGS-655	BnaA02G0156900ZS	scaffoldA02	9724469	9824468	100000	1485
BnaWGS-655	BnaA02G0156900ZS	scaffoldA02	9734469	9834468	100000	1363
BnaWGS-655	BnaA02G0156900ZS	scaffoldA02	9744469	9844468	100000	1414
BnaWGS-655	BnaA02G0156900ZS	scaffoldA02	9754469	9854468	100000	1536
BnaWGS-655	BnaA02G0156900ZS	scaffoldA02	9764469	9864468	100000	1751
BnaWGS-655	BnaA02G0156900ZS	scaffoldA02	9774469	9874468	100000	1792
BnaWGS-655	BnaA02G0156900ZS	scaffoldA02	9784469	9884468	100000	1849
BnaWGS-655	BnaA02G0156900ZS	scaffoldA02	9794469	9894468	100000	1869
BnaWGS-655	BnaA02G0156900ZS	scaffoldA02	9804469	9904468	100000	1795
BnaWGS-655	BnaA02G0156900ZS	scaffoldA02	9814469	9914468	100000	1801
BnaWGS-655	BnaA02G0156900ZS	scaffoldA02	9824469	9924468	100000	1866
BnaWGS-655	BnaA02G0156900ZS	scaffoldA02	9834469	9934468	100000	1869
BnaWGS-655	BnaA02G0156900ZS	scaffoldA02	9844469	9944468	100000	1948
BnaWGS-655	BnaA02G0156900ZS	scaffoldA02	9854469	9954468	100000	1876
BnaWGS-655	BnaA02G0156900ZS	scaffoldA02	9864469	9964468	100000	1720
BnaWGS-655	BnaA02G0156900ZS	scaffoldA02	9874469	9974468	100000	1747

For Review Only

BnaWGS-655	BnaA02G0156900ZS	scaffoldA02	9884469	9984468	100000	1673
BnaWGS-655	BnaA02G0156900ZS	scaffoldA02	9894469	9994468	100000	1749
BnaWGS-655	BnaA02G0156900ZS	scaffoldA02	9904469	10004468	100000	1745
BnaWGS-655	BnaA02G0156900ZS	scaffoldA02	9914469	10014468	100000	1745
BnaWGS-655	BnaA02G0156900ZS	scaffoldA02	9924469	10024468	100000	1561
BnaWGS-655	BnaA02G0156900ZS	scaffoldA02	9934469	10034468	100000	1521
BnaWGS-655	BnaA02G0156900ZS	scaffoldA02	9944469	10044468	100000	1597
BnaWGS-655	BnaA02G0156900ZS	scaffoldA02	9954469	10054468	100000	1800
BnaWGS-655	BnaA02G0156900ZS	scaffoldA02	9964469	10064468	100000	1875
BnaWGS-655	BnaA02G0156900ZS	scaffoldA02	9974469	10074468	100000	1695
BnaWGS-655	BnaA02G0156900ZS	scaffoldA02	9984469	10084468	100000	1648
BnaWGS-655	BnaA02G0156900ZS	scaffoldA02	9994469	10094468	100000	1536
BnaWGS-655	BnaA02G0156900ZS	scaffoldA02	10004469	10104468	100000	1483
BnaWGS-655	BnaA02G0156900ZS	scaffoldA02	10014469	10107241	92773	1313
BnaWGS-1007	BnaA10G0244800ZS	scaffoldA10	22943882	23043881	100000	511
BnaWGS-1007	BnaA10G0244800ZS	scaffoldA10	22953882	23053881	100000	488
BnaWGS-1007	BnaA10G0244800ZS	scaffoldA10	22963882	23063881	100000	489
BnaWGS-1007	BnaA10G0244800ZS	scaffoldA10	22973882	23073881	100000	512
BnaWGS-1007	BnaA10G0244800ZS	scaffoldA10	22983882	23083881	100000	485
BnaWGS-1007	BnaA10G0244800ZS	scaffoldA10	22993882	23093881	100000	513
BnaWGS-1007	BnaA10G0244800ZS	scaffoldA10	23003882	23103881	100000	536
BnaWGS-1007	BnaA10G0244800ZS	scaffoldA10	23013882	23113881	100000	569
BnaWGS-1007	BnaA10G0244800ZS	scaffoldA10	23023882	23123881	100000	568
BnaWGS-1007	BnaA10G0244800ZS	scaffoldA10	23033882	23133881	100000	655
BnaWGS-1007	BnaA10G0244800ZS	scaffoldA10	23043882	23143881	100000	746
BnaWGS-1007	BnaA10G0244800ZS	scaffoldA10	23053882	23153881	100000	833
BnaWGS-1007	BnaA10G0244800ZS	scaffoldA10	23063882	23163881	100000	942
BnaWGS-1007	BnaA10G0244800ZS	scaffoldA10	23073882	23173881	100000	981
BnaWGS-1007	BnaA10G0244800ZS	scaffoldA10	23083882	23183881	100000	1068
BnaWGS-1007	BnaA10G0244800ZS	scaffoldA10	23093882	23193881	100000	1086
BnaWGS-1007	BnaA10G0244800ZS	scaffoldA10	23103882	23203881	100000	1103
BnaWGS-1007	BnaA10G0244800ZS	scaffoldA10	23113882	23213881	100000	1212
BnaWGS-1007	BnaA10G0244800ZS	scaffoldA10	23123882	23223881	100000	1293
BnaWGS-1007	BnaA10G0244800ZS	scaffoldA10	23133882	23233881	100000	1177
BnaWGS-1007	BnaA10G0244800ZS	scaffoldA10	23143882	23243881	100000	1046
BnaWGS-1007	BnaA10G0244800ZS	scaffoldA10	23153882	23253881	100000	953
BnaWGS-1007	BnaA10G0244800ZS	scaffoldA10	23163882	23263881	100000	900
BnaWGS-1007	BnaA10G0244800ZS	scaffoldA10	23173882	23273881	100000	837
BnaWGS-1007	BnaA10G0244800ZS	scaffoldA10	23183882	23283881	100000	773
BnaWGS-1007	BnaA10G0244800ZS	scaffoldA10	23193882	23293881	100000	739
BnaWGS-1007	BnaA10G0244800ZS	scaffoldA10	23203882	23303881	100000	713
BnaWGS-1007	BnaA10G0244800ZS	scaffoldA10	23213882	23313881	100000	594
BnaWGS-1007	BnaA10G0244800ZS	scaffoldA10	23223882	23323881	100000	618
BnaWGS-1007	BnaA10G0244800ZS	scaffoldA10	23233882	23333881	100000	680
BnaWGS-1007	BnaA10G0244800ZS	scaffoldA10	23243882	23343881	100000	825
BnaWGS-1007	BnaA10G0244800ZS	scaffoldA10	23253882	23353881	100000	878
BnaWGS-1007	BnaA10G0244800ZS	scaffoldA10	23263882	23363881	100000	800
BnaWGS-1007	BnaA10G0244800ZS	scaffoldA10	23273882	23373881	100000	785
BnaWGS-1007	BnaA10G0244800ZS	scaffoldA10	23283882	23383881	100000	747
BnaWGS-1007	BnaA10G0244800ZS	scaffoldA10	23293882	23393881	100000	670
BnaWGS-1007	BnaA10G0244800ZS	scaffoldA10	23303882	23403881	100000	665
BnaWGS-1007	BnaA10G0244800ZS	scaffoldA10	23313882	23413881	100000	668
BnaWGS-1007	BnaA10G0244800ZS	scaffoldA10	23323882	23423881	100000	605
BnaWGS-1007	BnaA10G0244800ZS	scaffoldA10	23333882	23433881	100000	587
BnaWGS-1007	BnaA10G0244800ZS	scaffoldA10	23343882	23443881	100000	494
BnaWGS-1007	BnaA10G0244800ZS	scaffoldA10	23353882	23453881	100000	461
BnaWGS-1007	BnaA10G0244800ZS	scaffoldA10	23363882	23463881	100000	497
BnaWGS-1007	BnaA10G0244800ZS	scaffoldA10	23373882	23473881	100000	521
BnaWGS-1007	BnaA10G0244800ZS	scaffoldA10	23383882	23483881	100000	603
BnaWGS-1007	BnaA10G0244800ZS	scaffoldA10	23393882	23493881	100000	685

For Review Only

BnaWGS-1007	BnaA10G0244800ZS	scaffoldA10	23403882	23503881	100000	733
BnaWGS-1007	BnaA10G0244800ZS	scaffoldA10	23413882	23513881	100000	872
BnaWGS-1007	BnaA10G0244800ZS	scaffoldA10	23423882	23523881	100000	894
BnaWGS-1007	BnaA10G0244800ZS	scaffoldA10	23433882	23533881	100000	872
BnaWGS-1007	BnaA10G0244800ZS	scaffoldA10	23443882	23543881	100000	1015
BnaWGS-1007	BnaA10G0244800ZS	scaffoldA10	23453882	23553881	100000	1068
BnaWGS-1007	BnaA10G0244800ZS	scaffoldA10	23463882	23563881	100000	1090
BnaWGS-1007	BnaA10G0244800ZS	scaffoldA10	23473882	23573881	100000	1074
BnaWGS-1007	BnaA10G0244800ZS	scaffoldA10	23483882	23583881	100000	1002
BnaWGS-1007	BnaA10G0244800ZS	scaffoldA10	23493882	23593881	100000	978
BnaWGS-1007	BnaA10G0244800ZS	scaffoldA10	23503882	23603881	100000	999
BnaWGS-1007	BnaA10G0244800ZS	scaffoldA10	23513882	23613881	100000	831
BnaWGS-1007	BnaA10G0244800ZS	scaffoldA10	23523882	23623881	100000	851
BnaWGS-1007	BnaA10G0244800ZS	scaffoldA10	23533882	23633881	100000	934
BnaWGS-1007	BnaA10G0244800ZS	scaffoldA10	23543882	23643881	100000	830
BnaWGS-1007	BnaA10G0244800ZS	scaffoldA10	23553882	23653881	100000	856
BnaWGS-1007	BnaA10G0244800ZS	scaffoldA10	23563882	23663881	100000	890
BnaWGS-1007	BnaA10G0244800ZS	scaffoldA10	23573882	23673881	100000	1076
BnaWGS-1007	BnaA10G0244800ZS	scaffoldA10	23583882	23683881	100000	1220
BnaWGS-1007	BnaA10G0244800ZS	scaffoldA10	23593882	23693881	100000	1194
BnaWGS-1007	BnaA10G0244800ZS	scaffoldA10	23603882	23703881	100000	1129
BnaWGS-1007	BnaA10G0244800ZS	scaffoldA10	23613882	23713881	100000	1189
BnaWGS-1007	BnaA10G0244800ZS	scaffoldA10	23623882	23723881	100000	1142
BnaWGS-1007	BnaA10G0244800ZS	scaffoldA10	23633882	23733881	100000	1113
BnaWGS-1007	BnaA10G0244800ZS	scaffoldA10	23643882	23743881	100000	1172
BnaWGS-1007	BnaA10G0244800ZS	scaffoldA10	23653882	23753881	100000	1237
BnaWGS-1007	BnaA10G0244800ZS	scaffoldA10	23663882	23763881	100000	1262
BnaWGS-1007	BnaA10G0244800ZS	scaffoldA10	23673882	23773881	100000	1134
BnaWGS-1007	BnaA10G0244800ZS	scaffoldA10	23683882	23783881	100000	1076
BnaWGS-1007	BnaA10G0244800ZS	scaffoldA10	23693882	23793881	100000	1115
BnaWGS-1007	BnaA10G0244800ZS	scaffoldA10	23703882	23803881	100000	1167
BnaWGS-1007	BnaA10G0244800ZS	scaffoldA10	23713882	23813881	100000	1176
BnaWGS-1007	BnaA10G0244800ZS	scaffoldA10	23723882	23823881	100000	1178
BnaWGS-1007	BnaA10G0244800ZS	scaffoldA10	23733882	23833881	100000	1122
BnaWGS-1007	BnaA10G0244800ZS	scaffoldA10	23743882	23843881	100000	998
BnaWGS-1007	BnaA10G0244800ZS	scaffoldA10	23753882	23853881	100000	913
BnaWGS-1007	BnaA10G0244800ZS	scaffoldA10	23763882	23863881	100000	916
BnaWGS-1007	BnaA10G0244800ZS	scaffoldA10	23773882	23873881	100000	848
BnaWGS-1007	BnaA10G0244800ZS	scaffoldA10	23783882	23883881	100000	793
BnaWGS-1007	BnaA10G0244800ZS	scaffoldA10	23793882	23893881	100000	744
BnaWGS-1007	BnaA10G0244800ZS	scaffoldA10	23803882	23903881	100000	684
BnaWGS-1007	BnaA10G0244800ZS	scaffoldA10	23813882	23913881	100000	623
BnaWGS-1007	BnaA10G0244800ZS	scaffoldA10	23823882	23923881	100000	567
BnaWGS-1007	BnaA10G0244800ZS	scaffoldA10	23833882	23933881	100000	546
BnaWGS-1007	BnaA10G0244800ZS	scaffoldA10	23843882	23943881	100000	580
BnaWGS-1007	BnaA10G0244800ZS	scaffoldA10	23853882	23953881	100000	513
BnaWGS-1007	BnaA10G0244800ZS	scaffoldA10	23863882	23963881	100000	409
BnaWGS-1007	BnaA10G0244800ZS	scaffoldA10	23873882	23973881	100000	432
BnaWGS-1007	BnaA10G0244800ZS	scaffoldA10	23883882	23983881	100000	588
BnaWGS-1007	BnaA10G0244800ZS	scaffoldA10	23893882	23993881	100000	585
BnaWGS-1007	BnaA10G0244800ZS	scaffoldA10	23903882	24003881	100000	620
BnaWGS-1007	BnaA10G0244800ZS	scaffoldA10	23913882	24013881	100000	659
BnaWGS-1007	BnaA10G0244800ZS	scaffoldA10	23923882	24023881	100000	750
BnaWGS-1007	BnaA10G0244800ZS	scaffoldA10	23933882	24033881	100000	747
BnaWGS-1007	BnaA10G0244800ZS	scaffoldA10	23943882	24043881	100000	823
BnaWGS-1007	BnaA10G0244800ZS	scaffoldA10	23953882	24053881	100000	831
BnaWGS-1007	BnaA10G0244800ZS	scaffoldA10	23963882	24063881	100000	919
BnaWGS-1007	BnaA10G0244800ZS	scaffoldA10	23973882	24073881	100000	1039
BnaWGS-1007	BnaA10G0244800ZS	scaffoldA10	23983882	24083881	100000	1020
BnaWGS-1007	BnaA10G0244800ZS	scaffoldA10	23993882	24093881	100000	1197

For Review Only

BnaWGS-1007	BnaA10G0244800ZS	scaffoldA10	24003882	24103881	100000	1342
BnaWGS-1007	BnaA10G0244800ZS	scaffoldA10	24013882	24113881	100000	1400
BnaWGS-1007	BnaA10G0244800ZS	scaffoldA10	24023882	24123881	100000	1506
BnaWGS-1007	BnaA10G0244800ZS	scaffoldA10	24033882	24133881	100000	1553
BnaWGS-1007	BnaA10G0244800ZS	scaffoldA10	24043882	24143881	100000	1523
BnaWGS-1007	BnaA10G0244800ZS	scaffoldA10	24053882	24153881	100000	1592
BnaWGS-1007	BnaA10G0244800ZS	scaffoldA10	24063882	24163881	100000	1607
BnaWGS-1007	BnaA10G0244800ZS	scaffoldA10	24073882	24173881	100000	1645
BnaWGS-1007	BnaA10G0244800ZS	scaffoldA10	24083882	24183881	100000	1712
BnaWGS-1007	BnaA10G0244800ZS	scaffoldA10	24093882	24193881	100000	1794
BnaWGS-1007	BnaA10G0244800ZS	scaffoldA10	24103882	24203881	100000	1636
BnaWGS-1007	BnaA10G0244800ZS	scaffoldA10	24113882	24213881	100000	1549
BnaWGS-1007	BnaA10G0244800ZS	scaffoldA10	24123882	24223881	100000	1535
BnaWGS-1007	BnaA10G0244800ZS	scaffoldA10	24133882	24233881	100000	1641
BnaWGS-1007	BnaA10G0244800ZS	scaffoldA10	24143882	24243881	100000	1663
BnaWGS-1007	BnaA10G0244800ZS	scaffoldA10	24153882	24253881	100000	1761
BnaWGS-1007	BnaA10G0244800ZS	scaffoldA10	24163882	24263881	100000	1778
BnaWGS-1007	BnaA10G0244800ZS	scaffoldA10	24173882	24273881	100000	1804
BnaWGS-1007	BnaA10G0244800ZS	scaffoldA10	24183882	24283881	100000	1655
BnaWGS-1007	BnaA10G0244800ZS	scaffoldA10	24193882	24293881	100000	1439
BnaWGS-1007	BnaA10G0244800ZS	scaffoldA10	24203882	24303881	100000	1554
BnaWGS-1007	BnaA10G0244800ZS	scaffoldA10	24213882	24313881	100000	1699
BnaWGS-1007	BnaA10G0244800ZS	scaffoldA10	24223882	24323881	100000	1713
BnaWGS-1007	BnaA10G0244800ZS	scaffoldA10	24233882	24333881	100000	1662
BnaWGS-1007	BnaA10G0244800ZS	scaffoldA10	24243882	24343881	100000	1729
BnaWGS-1007	BnaA10G0244800ZS	scaffoldA10	24253882	24353881	100000	1748
BnaWGS-1007	BnaA10G0244800ZS	scaffoldA10	24263882	24363881	100000	1674
BnaWGS-1007	BnaA10G0244800ZS	scaffoldA10	24273882	24373881	100000	1603
BnaWGS-1007	BnaA10G0244800ZS	scaffoldA10	24283882	24383881	100000	1647
BnaWGS-1007	BnaA10G0244800ZS	scaffoldA10	24293882	24393881	100000	1623
BnaWGS-1007	BnaA10G0244800ZS	scaffoldA10	24303882	24403881	100000	1448
BnaWGS-1007	BnaA10G0244800ZS	scaffoldA10	24313882	24413881	100000	1427
BnaWGS-1007	BnaA10G0244800ZS	scaffoldA10	24323882	24423881	100000	1434
BnaWGS-1007	BnaA10G0244800ZS	scaffoldA10	24333882	24433881	100000	1449
BnaWGS-1007	BnaA10G0244800ZS	scaffoldA10	24343882	24443881	100000	1324
BnaWGS-1007	BnaA10G0244800ZS	scaffoldA10	24353882	24453881	100000	1165
BnaWGS-1007	BnaA10G0244800ZS	scaffoldA10	24363882	24463881	100000	1306
BnaWGS-1007	BnaA10G0244800ZS	scaffoldA10	24373882	24473881	100000	1314
BnaWGS-1007	BnaA10G0244800ZS	scaffoldA10	24383882	24483881	100000	1244
BnaWGS-1007	BnaA10G0244800ZS	scaffoldA10	24393882	24493881	100000	1326
BnaWGS-1007	BnaA10G0244800ZS	scaffoldA10	24403882	24503881	100000	1382
BnaWGS-1007	BnaA10G0244800ZS	scaffoldA10	24413882	24513881	100000	1286
BnaWGS-1007	BnaA10G0244800ZS	scaffoldA10	24423882	24523881	100000	1126
BnaWGS-1007	BnaA10G0244800ZS	scaffoldA10	24433882	24533881	100000	1033
BnaWGS-1007	BnaA10G0244800ZS	scaffoldA10	24443882	24543881	100000	1049
BnaWGS-1007	BnaA10G0244800ZS	scaffoldA10	24453882	24553881	100000	1114
BnaWGS-1007	BnaA10G0244800ZS	scaffoldA10	24463882	24563881	100000	1052
BnaWGS-1007	BnaA10G0244800ZS	scaffoldA10	24473882	24573881	100000	1023
BnaWGS-1007	BnaA10G0244800ZS	scaffoldA10	24483882	24583881	100000	1056
BnaWGS-1007	BnaA10G0244800ZS	scaffoldA10	24493882	24593881	100000	980
BnaWGS-1007	BnaA10G0244800ZS	scaffoldA10	24503882	24603881	100000	1008
BnaWGS-1007	BnaA10G0244800ZS	scaffoldA10	24513882	24613881	100000	1025
BnaWGS-1007	BnaA10G0244800ZS	scaffoldA10	24523882	24623881	100000	1086
BnaWGS-1007	BnaA10G0244800ZS	scaffoldA10	24533882	24633881	100000	1145
BnaWGS-1007	BnaA10G0244800ZS	scaffoldA10	24543882	24643881	100000	1182
BnaWGS-1007	BnaA10G0244800ZS	scaffoldA10	24553882	24653881	100000	1172
BnaWGS-1007	BnaA10G0244800ZS	scaffoldA10	24563882	24663881	100000	1138
BnaWGS-1007	BnaA10G0244800ZS	scaffoldA10	24573882	24673881	100000	1024
BnaWGS-1007	BnaA10G0244800ZS	scaffoldA10	24583882	24683881	100000	1010
BnaWGS-1007	BnaA10G0244800ZS	scaffoldA10	24593882	24693881	100000	1040

For Review Only

BnaWGS-1007	BnaA10G0244800ZS	scaffoldA10	24603882	24703881	100000	1011
BnaWGS-1007	BnaA10G0244800ZS	scaffoldA10	24613882	24713881	100000	980
BnaWGS-1007	BnaA10G0244800ZS	scaffoldA10	24623882	24723881	100000	910
BnaWGS-1007	BnaA10G0244800ZS	scaffoldA10	24633882	24733881	100000	915
BnaWGS-1007	BnaA10G0244800ZS	scaffoldA10	24643882	24743881	100000	913
BnaWGS-1007	BnaA10G0244800ZS	scaffoldA10	24653882	24753881	100000	932
BnaWGS-1007	BnaA10G0244800ZS	scaffoldA10	24663882	24763881	100000	838
BnaWGS-1007	BnaA10G0244800ZS	scaffoldA10	24673882	24773881	100000	802
BnaWGS-1007	BnaA10G0244800ZS	scaffoldA10	24683882	24783881	100000	849
BnaWGS-1007	BnaA10G0244800ZS	scaffoldA10	24693882	24793881	100000	845
BnaWGS-1007	BnaA10G0244800ZS	scaffoldA10	24703882	24803881	100000	783
BnaWGS-1007	BnaA10G0244800ZS	scaffoldA10	24713882	24813881	100000	778
BnaWGS-1007	BnaA10G0244800ZS	scaffoldA10	24723882	24823881	100000	926
BnaWGS-1007	BnaA10G0244800ZS	scaffoldA10	24733882	24833881	100000	935
BnaWGS-1007	BnaA10G0244800ZS	scaffoldA10	24743882	24843881	100000	961
BnaWGS-1007	BnaA10G0244800ZS	scaffoldA10	24753882	24853881	100000	910
BnaWGS-1007	BnaA10G0244800ZS	scaffoldA10	24763882	24863881	100000	923
BnaWGS-1007	BnaA10G0244800ZS	scaffoldA10	24773882	24873881	100000	1033
BnaWGS-1007	BnaA10G0244800ZS	scaffoldA10	24783882	24883881	100000	932
BnaWGS-1007	BnaA10G0244800ZS	scaffoldA10	24793882	24893881	100000	899
BnaWGS-1007	BnaA10G0244800ZS	scaffoldA10	24803882	24903881	100000	1034
BnaWGS-1007	BnaA10G0244800ZS	scaffoldA10	24813882	24913881	100000	1090
BnaWGS-1007	BnaA10G0244800ZS	scaffoldA10	24823882	24923881	100000	1222
BnaWGS-1007	BnaA10G0244800ZS	scaffoldA10	24833882	24933881	100000	1312
BnaWGS-1007	BnaA10G0244800ZS	scaffoldA10	24843882	24943881	100000	1248
BnaWGS-1007	BnaA10G0244800ZS	scaffoldA10	24853882	24947904	94023	1187
BnaWGS-1007	BnaA10G0244800ZS	scaffoldA10	22943882	23043881	100000	511
BnaWGS-1007	BnaA10G0244800ZS	scaffoldA10	22953882	23053881	100000	488
BnaWGS-1007	BnaA10G0244800ZS	scaffoldA10	22963882	23063881	100000	489
BnaWGS-1007	BnaA10G0244800ZS	scaffoldA10	22973882	23073881	100000	512
BnaWGS-1007	BnaA10G0244800ZS	scaffoldA10	22983882	23083881	100000	485
BnaWGS-1007	BnaA10G0244800ZS	scaffoldA10	22993882	23093881	100000	513
BnaWGS-1007	BnaA10G0244800ZS	scaffoldA10	23003882	23103881	100000	536
BnaWGS-1007	BnaA10G0244800ZS	scaffoldA10	23013882	23113881	100000	569
BnaWGS-1007	BnaA10G0244800ZS	scaffoldA10	23023882	23123881	100000	568
BnaWGS-1007	BnaA10G0244800ZS	scaffoldA10	23033882	23133881	100000	655
BnaWGS-1007	BnaA10G0244800ZS	scaffoldA10	23043882	23143881	100000	746
BnaWGS-1007	BnaA10G0244800ZS	scaffoldA10	23053882	23153881	100000	833
BnaWGS-1007	BnaA10G0244800ZS	scaffoldA10	23063882	23163881	100000	942
BnaWGS-1007	BnaA10G0244800ZS	scaffoldA10	23073882	23173881	100000	981
BnaWGS-1007	BnaA10G0244800ZS	scaffoldA10	23083882	23183881	100000	1068
BnaWGS-1007	BnaA10G0244800ZS	scaffoldA10	23093882	23193881	100000	1086
BnaWGS-1007	BnaA10G0244800ZS	scaffoldA10	23103882	23203881	100000	1103
BnaWGS-1007	BnaA10G0244800ZS	scaffoldA10	23113882	23213881	100000	1212
BnaWGS-1007	BnaA10G0244800ZS	scaffoldA10	23123882	23223881	100000	1293
BnaWGS-1007	BnaA10G0244800ZS	scaffoldA10	23133882	23233881	100000	1177
BnaWGS-1007	BnaA10G0244800ZS	scaffoldA10	23143882	23243881	100000	1046
BnaWGS-1007	BnaA10G0244800ZS	scaffoldA10	23153882	23253881	100000	953
BnaWGS-1007	BnaA10G0244800ZS	scaffoldA10	23163882	23263881	100000	900
BnaWGS-1007	BnaA10G0244800ZS	scaffoldA10	23173882	23273881	100000	837
BnaWGS-1007	BnaA10G0244800ZS	scaffoldA10	23183882	23283881	100000	773
BnaWGS-1007	BnaA10G0244800ZS	scaffoldA10	23193882	23293881	100000	739
BnaWGS-1007	BnaA10G0244800ZS	scaffoldA10	23203882	23303881	100000	713
BnaWGS-1007	BnaA10G0244800ZS	scaffoldA10	23213882	23313881	100000	594
BnaWGS-1007	BnaA10G0244800ZS	scaffoldA10	23223882	23323881	100000	618
BnaWGS-1007	BnaA10G0244800ZS	scaffoldA10	23233882	23333881	100000	680
BnaWGS-1007	BnaA10G0244800ZS	scaffoldA10	23243882	23343881	100000	825
BnaWGS-1007	BnaA10G0244800ZS	scaffoldA10	23253882	23353881	100000	878
BnaWGS-1007	BnaA10G0244800ZS	scaffoldA10	23263882	23363881	100000	800
BnaWGS-1007	BnaA10G0244800ZS	scaffoldA10	23273882	23373881	100000	785

For Review Only

BnaWGS-1007	BnaA10G0244800ZS	scaffoldA10	23283882	23383881	100000	747
BnaWGS-1007	BnaA10G0244800ZS	scaffoldA10	23293882	23393881	100000	670
BnaWGS-1007	BnaA10G0244800ZS	scaffoldA10	23303882	23403881	100000	665
BnaWGS-1007	BnaA10G0244800ZS	scaffoldA10	23313882	23413881	100000	668
BnaWGS-1007	BnaA10G0244800ZS	scaffoldA10	23323882	23423881	100000	605
BnaWGS-1007	BnaA10G0244800ZS	scaffoldA10	23333882	23433881	100000	587
BnaWGS-1007	BnaA10G0244800ZS	scaffoldA10	23343882	23443881	100000	494
BnaWGS-1007	BnaA10G0244800ZS	scaffoldA10	23353882	23453881	100000	461
BnaWGS-1007	BnaA10G0244800ZS	scaffoldA10	23363882	23463881	100000	497
BnaWGS-1007	BnaA10G0244800ZS	scaffoldA10	23373882	23473881	100000	521
BnaWGS-1007	BnaA10G0244800ZS	scaffoldA10	23383882	23483881	100000	603
BnaWGS-1007	BnaA10G0244800ZS	scaffoldA10	23393882	23493881	100000	685
BnaWGS-1007	BnaA10G0244800ZS	scaffoldA10	23403882	23503881	100000	733
BnaWGS-1007	BnaA10G0244800ZS	scaffoldA10	23413882	23513881	100000	872
BnaWGS-1007	BnaA10G0244800ZS	scaffoldA10	23423882	23523881	100000	894
BnaWGS-1007	BnaA10G0244800ZS	scaffoldA10	23433882	23533881	100000	872
BnaWGS-1007	BnaA10G0244800ZS	scaffoldA10	23443882	23543881	100000	1015
BnaWGS-1007	BnaA10G0244800ZS	scaffoldA10	23453882	23553881	100000	1068
BnaWGS-1007	BnaA10G0244800ZS	scaffoldA10	23463882	23563881	100000	1090
BnaWGS-1007	BnaA10G0244800ZS	scaffoldA10	23473882	23573881	100000	1074
BnaWGS-1007	BnaA10G0244800ZS	scaffoldA10	23483882	23583881	100000	1002
BnaWGS-1007	BnaA10G0244800ZS	scaffoldA10	23493882	23593881	100000	978
BnaWGS-1007	BnaA10G0244800ZS	scaffoldA10	23503882	23603881	100000	999
BnaWGS-1007	BnaA10G0244800ZS	scaffoldA10	23513882	23613881	100000	831
BnaWGS-1007	BnaA10G0244800ZS	scaffoldA10	23523882	23623881	100000	851
BnaWGS-1007	BnaA10G0244800ZS	scaffoldA10	23533882	23633881	100000	934
BnaWGS-1007	BnaA10G0244800ZS	scaffoldA10	23543882	23643881	100000	830
BnaWGS-1007	BnaA10G0244800ZS	scaffoldA10	23553882	23653881	100000	856
BnaWGS-1007	BnaA10G0244800ZS	scaffoldA10	23563882	23663881	100000	890
BnaWGS-1007	BnaA10G0244800ZS	scaffoldA10	23573882	23673881	100000	1076
BnaWGS-1007	BnaA10G0244800ZS	scaffoldA10	23583882	23683881	100000	1220
BnaWGS-1007	BnaA10G0244800ZS	scaffoldA10	23593882	23693881	100000	1194
BnaWGS-1007	BnaA10G0244800ZS	scaffoldA10	23603882	23703881	100000	1129
BnaWGS-1007	BnaA10G0244800ZS	scaffoldA10	23613882	23713881	100000	1189
BnaWGS-1007	BnaA10G0244800ZS	scaffoldA10	23623882	23723881	100000	1142
BnaWGS-1007	BnaA10G0244800ZS	scaffoldA10	23633882	23733881	100000	1113
BnaWGS-1007	BnaA10G0244800ZS	scaffoldA10	23643882	23743881	100000	1172
BnaWGS-1007	BnaA10G0244800ZS	scaffoldA10	23653882	23753881	100000	1237
BnaWGS-1007	BnaA10G0244800ZS	scaffoldA10	23663882	23763881	100000	1262
BnaWGS-1007	BnaA10G0244800ZS	scaffoldA10	23673882	23773881	100000	1134
BnaWGS-1007	BnaA10G0244800ZS	scaffoldA10	23683882	23783881	100000	1076
BnaWGS-1007	BnaA10G0244800ZS	scaffoldA10	23693882	23793881	100000	1115
BnaWGS-1007	BnaA10G0244800ZS	scaffoldA10	23703882	23803881	100000	1167
BnaWGS-1007	BnaA10G0244800ZS	scaffoldA10	23713882	23813881	100000	1176
BnaWGS-1007	BnaA10G0244800ZS	scaffoldA10	23723882	23823881	100000	1178
BnaWGS-1007	BnaA10G0244800ZS	scaffoldA10	23733882	23833881	100000	1122
BnaWGS-1007	BnaA10G0244800ZS	scaffoldA10	23743882	23843881	100000	998
BnaWGS-1007	BnaA10G0244800ZS	scaffoldA10	23753882	23853881	100000	913
BnaWGS-1007	BnaA10G0244800ZS	scaffoldA10	23763882	23863881	100000	916
BnaWGS-1007	BnaA10G0244800ZS	scaffoldA10	23773882	23873881	100000	848
BnaWGS-1007	BnaA10G0244800ZS	scaffoldA10	23783882	23883881	100000	793
BnaWGS-1007	BnaA10G0244800ZS	scaffoldA10	23793882	23893881	100000	744
BnaWGS-1007	BnaA10G0244800ZS	scaffoldA10	23803882	23903881	100000	684
BnaWGS-1007	BnaA10G0244800ZS	scaffoldA10	23813882	23913881	100000	623
BnaWGS-1007	BnaA10G0244800ZS	scaffoldA10	23823882	23923881	100000	567
BnaWGS-1007	BnaA10G0244800ZS	scaffoldA10	23833882	23933881	100000	546
BnaWGS-1007	BnaA10G0244800ZS	scaffoldA10	23843882	23943881	100000	580
BnaWGS-1007	BnaA10G0244800ZS	scaffoldA10	23853882	23953881	100000	513
BnaWGS-1007	BnaA10G0244800ZS	scaffoldA10	23863882	23963881	100000	409
BnaWGS-1007	BnaA10G0244800ZS	scaffoldA10	23873882	23973881	100000	432

For Review Only

BnaWGS-1007	BnaA10G0244800ZS	scaffoldA10	23883882	23983881	100000	588
BnaWGS-1007	BnaA10G0244800ZS	scaffoldA10	23893882	23993881	100000	585
BnaWGS-1007	BnaA10G0244800ZS	scaffoldA10	23903882	24003881	100000	620
BnaWGS-1007	BnaA10G0244800ZS	scaffoldA10	23913882	24013881	100000	659
BnaWGS-1007	BnaA10G0244800ZS	scaffoldA10	23923882	24023881	100000	750
BnaWGS-1007	BnaA10G0244800ZS	scaffoldA10	23933882	24033881	100000	747
BnaWGS-1007	BnaA10G0244800ZS	scaffoldA10	23943882	24043881	100000	823
BnaWGS-1007	BnaA10G0244800ZS	scaffoldA10	23953882	24053881	100000	831
BnaWGS-1007	BnaA10G0244800ZS	scaffoldA10	23963882	24063881	100000	919
BnaWGS-1007	BnaA10G0244800ZS	scaffoldA10	23973882	24073881	100000	1039
BnaWGS-1007	BnaA10G0244800ZS	scaffoldA10	23983882	24083881	100000	1020
BnaWGS-1007	BnaA10G0244800ZS	scaffoldA10	23993882	24093881	100000	1197
BnaWGS-1007	BnaA10G0244800ZS	scaffoldA10	24003882	24103881	100000	1342
BnaWGS-1007	BnaA10G0244800ZS	scaffoldA10	24013882	24113881	100000	1400
BnaWGS-1007	BnaA10G0244800ZS	scaffoldA10	24023882	24123881	100000	1506
BnaWGS-1007	BnaA10G0244800ZS	scaffoldA10	24033882	24133881	100000	1553
BnaWGS-1007	BnaA10G0244800ZS	scaffoldA10	24043882	24143881	100000	1523
BnaWGS-1007	BnaA10G0244800ZS	scaffoldA10	24053882	24153881	100000	1592
BnaWGS-1007	BnaA10G0244800ZS	scaffoldA10	24063882	24163881	100000	1607
BnaWGS-1007	BnaA10G0244800ZS	scaffoldA10	24073882	24173881	100000	1645
BnaWGS-1007	BnaA10G0244800ZS	scaffoldA10	24083882	24183881	100000	1712
BnaWGS-1007	BnaA10G0244800ZS	scaffoldA10	24093882	24193881	100000	1794
BnaWGS-1007	BnaA10G0244800ZS	scaffoldA10	24103882	24203881	100000	1636
BnaWGS-1007	BnaA10G0244800ZS	scaffoldA10	24113882	24213881	100000	1549
BnaWGS-1007	BnaA10G0244800ZS	scaffoldA10	24123882	24223881	100000	1535
BnaWGS-1007	BnaA10G0244800ZS	scaffoldA10	24133882	24233881	100000	1641
BnaWGS-1007	BnaA10G0244800ZS	scaffoldA10	24143882	24243881	100000	1663
BnaWGS-1007	BnaA10G0244800ZS	scaffoldA10	24153882	24253881	100000	1761
BnaWGS-1007	BnaA10G0244800ZS	scaffoldA10	24163882	24263881	100000	1778
BnaWGS-1007	BnaA10G0244800ZS	scaffoldA10	24173882	24273881	100000	1804
BnaWGS-1007	BnaA10G0244800ZS	scaffoldA10	24183882	24283881	100000	1655
BnaWGS-1007	BnaA10G0244800ZS	scaffoldA10	24193882	24293881	100000	1439
BnaWGS-1007	BnaA10G0244800ZS	scaffoldA10	24203882	24303881	100000	1554
BnaWGS-1007	BnaA10G0244800ZS	scaffoldA10	24213882	24313881	100000	1699
BnaWGS-1007	BnaA10G0244800ZS	scaffoldA10	24223882	24323881	100000	1713
BnaWGS-1007	BnaA10G0244800ZS	scaffoldA10	24233882	24333881	100000	1662
BnaWGS-1007	BnaA10G0244800ZS	scaffoldA10	24243882	24343881	100000	1729
BnaWGS-1007	BnaA10G0244800ZS	scaffoldA10	24253882	24353881	100000	1748
BnaWGS-1007	BnaA10G0244800ZS	scaffoldA10	24263882	24363881	100000	1674
BnaWGS-1007	BnaA10G0244800ZS	scaffoldA10	24273882	24373881	100000	1603
BnaWGS-1007	BnaA10G0244800ZS	scaffoldA10	24283882	24383881	100000	1647
BnaWGS-1007	BnaA10G0244800ZS	scaffoldA10	24293882	24393881	100000	1623
BnaWGS-1007	BnaA10G0244800ZS	scaffoldA10	24303882	24403881	100000	1448
BnaWGS-1007	BnaA10G0244800ZS	scaffoldA10	24313882	24413881	100000	1427
BnaWGS-1007	BnaA10G0244800ZS	scaffoldA10	24323882	24423881	100000	1434
BnaWGS-1007	BnaA10G0244800ZS	scaffoldA10	24333882	24433881	100000	1449
BnaWGS-1007	BnaA10G0244800ZS	scaffoldA10	24343882	24443881	100000	1324
BnaWGS-1007	BnaA10G0244800ZS	scaffoldA10	24353882	24453881	100000	1165
BnaWGS-1007	BnaA10G0244800ZS	scaffoldA10	24363882	24463881	100000	1306
BnaWGS-1007	BnaA10G0244800ZS	scaffoldA10	24373882	24473881	100000	1314
BnaWGS-1007	BnaA10G0244800ZS	scaffoldA10	24383882	24483881	100000	1244
BnaWGS-1007	BnaA10G0244800ZS	scaffoldA10	24393882	24493881	100000	1326
BnaWGS-1007	BnaA10G0244800ZS	scaffoldA10	24403882	24503881	100000	1382
BnaWGS-1007	BnaA10G0244800ZS	scaffoldA10	24413882	24513881	100000	1286
BnaWGS-1007	BnaA10G0244800ZS	scaffoldA10	24423882	24523881	100000	1126
BnaWGS-1007	BnaA10G0244800ZS	scaffoldA10	24433882	24533881	100000	1033
BnaWGS-1007	BnaA10G0244800ZS	scaffoldA10	24443882	24543881	100000	1049
BnaWGS-1007	BnaA10G0244800ZS	scaffoldA10	24453882	24553881	100000	1114
BnaWGS-1007	BnaA10G0244800ZS	scaffoldA10	24463882	24563881	100000	1052
BnaWGS-1007	BnaA10G0244800ZS	scaffoldA10	24473882	24573881	100000	1023

For Review Only

BnaWGS-1007	BnaA10G0244800ZS	scaffoldA10	24483882	24583881	100000	1056
BnaWGS-1007	BnaA10G0244800ZS	scaffoldA10	24493882	24593881	100000	980
BnaWGS-1007	BnaA10G0244800ZS	scaffoldA10	24503882	24603881	100000	1008
BnaWGS-1007	BnaA10G0244800ZS	scaffoldA10	24513882	24613881	100000	1025
BnaWGS-1007	BnaA10G0244800ZS	scaffoldA10	24523882	24623881	100000	1086
BnaWGS-1007	BnaA10G0244800ZS	scaffoldA10	24533882	24633881	100000	1145
BnaWGS-1007	BnaA10G0244800ZS	scaffoldA10	24543882	24643881	100000	1182
BnaWGS-1007	BnaA10G0244800ZS	scaffoldA10	24553882	24653881	100000	1172
BnaWGS-1007	BnaA10G0244800ZS	scaffoldA10	24563882	24663881	100000	1138
BnaWGS-1007	BnaA10G0244800ZS	scaffoldA10	24573882	24673881	100000	1024
BnaWGS-1007	BnaA10G0244800ZS	scaffoldA10	24583882	24683881	100000	1010
BnaWGS-1007	BnaA10G0244800ZS	scaffoldA10	24593882	24693881	100000	1040
BnaWGS-1007	BnaA10G0244800ZS	scaffoldA10	24603882	24703881	100000	1011
BnaWGS-1007	BnaA10G0244800ZS	scaffoldA10	24613882	24713881	100000	980
BnaWGS-1007	BnaA10G0244800ZS	scaffoldA10	24623882	24723881	100000	910
BnaWGS-1007	BnaA10G0244800ZS	scaffoldA10	24633882	24733881	100000	915
BnaWGS-1007	BnaA10G0244800ZS	scaffoldA10	24643882	24743881	100000	913
BnaWGS-1007	BnaA10G0244800ZS	scaffoldA10	24653882	24753881	100000	932
BnaWGS-1007	BnaA10G0244800ZS	scaffoldA10	24663882	24763881	100000	838
BnaWGS-1007	BnaA10G0244800ZS	scaffoldA10	24673882	24773881	100000	802
BnaWGS-1007	BnaA10G0244800ZS	scaffoldA10	24683882	24783881	100000	849
BnaWGS-1007	BnaA10G0244800ZS	scaffoldA10	24693882	24793881	100000	845
BnaWGS-1007	BnaA10G0244800ZS	scaffoldA10	24703882	24803881	100000	783
BnaWGS-1007	BnaA10G0244800ZS	scaffoldA10	24713882	24813881	100000	778
BnaWGS-1007	BnaA10G0244800ZS	scaffoldA10	24723882	24823881	100000	926
BnaWGS-1007	BnaA10G0244800ZS	scaffoldA10	24733882	24833881	100000	935
BnaWGS-1007	BnaA10G0244800ZS	scaffoldA10	24743882	24843881	100000	961
BnaWGS-1007	BnaA10G0244800ZS	scaffoldA10	24753882	24853881	100000	910
BnaWGS-1007	BnaA10G0244800ZS	scaffoldA10	24763882	24863881	100000	923
BnaWGS-1007	BnaA10G0244800ZS	scaffoldA10	24773882	24873881	100000	1033
BnaWGS-1007	BnaA10G0244800ZS	scaffoldA10	24783882	24883881	100000	932
BnaWGS-1007	BnaA10G0244800ZS	scaffoldA10	24793882	24893881	100000	899
BnaWGS-1007	BnaA10G0244800ZS	scaffoldA10	24803882	24903881	100000	1034
BnaWGS-1007	BnaA10G0244800ZS	scaffoldA10	24813882	24913881	100000	1090
BnaWGS-1007	BnaA10G0244800ZS	scaffoldA10	24823882	24923881	100000	1222
BnaWGS-1007	BnaA10G0244800ZS	scaffoldA10	24833882	24933881	100000	1312
BnaWGS-1007	BnaA10G0244800ZS	scaffoldA10	24843882	24943881	100000	1248
BnaWGS-1007	BnaA10G0244800ZS	scaffoldA10	24853882	24947904	94023	1187
BnaWGS-1007	BnaA10G0244800ZS	scaffoldA10	22943882	23043881	100000	511
BnaWGS-1007	BnaA10G0244800ZS	scaffoldA10	22953882	23053881	100000	488
BnaWGS-1007	BnaA10G0244800ZS	scaffoldA10	22963882	23063881	100000	489
BnaWGS-1007	BnaA10G0244800ZS	scaffoldA10	22973882	23073881	100000	512
BnaWGS-1007	BnaA10G0244800ZS	scaffoldA10	22983882	23083881	100000	485
BnaWGS-1007	BnaA10G0244800ZS	scaffoldA10	22993882	23093881	100000	513
BnaWGS-1007	BnaA10G0244800ZS	scaffoldA10	23003882	23103881	100000	536
BnaWGS-1007	BnaA10G0244800ZS	scaffoldA10	23013882	23113881	100000	569
BnaWGS-1007	BnaA10G0244800ZS	scaffoldA10	23023882	23123881	100000	568
BnaWGS-1007	BnaA10G0244800ZS	scaffoldA10	23033882	23133881	100000	655
BnaWGS-1007	BnaA10G0244800ZS	scaffoldA10	23043882	23143881	100000	746
BnaWGS-1007	BnaA10G0244800ZS	scaffoldA10	23053882	23153881	100000	833
BnaWGS-1007	BnaA10G0244800ZS	scaffoldA10	23063882	23163881	100000	942
BnaWGS-1007	BnaA10G0244800ZS	scaffoldA10	23073882	23173881	100000	981
BnaWGS-1007	BnaA10G0244800ZS	scaffoldA10	23083882	23183881	100000	1068
BnaWGS-1007	BnaA10G0244800ZS	scaffoldA10	23093882	23193881	100000	1086
BnaWGS-1007	BnaA10G0244800ZS	scaffoldA10	23103882	23203881	100000	1103
BnaWGS-1007	BnaA10G0244800ZS	scaffoldA10	23113882	23213881	100000	1212
BnaWGS-1007	BnaA10G0244800ZS	scaffoldA10	23123882	23223881	100000	1293
BnaWGS-1007	BnaA10G0244800ZS	scaffoldA10	23133882	23233881	100000	1177
BnaWGS-1007	BnaA10G0244800ZS	scaffoldA10	23143882	23243881	100000	1046
BnaWGS-1007	BnaA10G0244800ZS	scaffoldA10	23153882	23253881	100000	953

BnaWGS-1007	BnaA10G0244800ZS	scaffoldA10	23163882	23263881	100000	900
BnaWGS-1007	BnaA10G0244800ZS	scaffoldA10	23173882	23273881	100000	837
BnaWGS-1007	BnaA10G0244800ZS	scaffoldA10	23183882	23283881	100000	773
BnaWGS-1007	BnaA10G0244800ZS	scaffoldA10	23193882	23293881	100000	739
BnaWGS-1007	BnaA10G0244800ZS	scaffoldA10	23203882	23303881	100000	713
BnaWGS-1007	BnaA10G0244800ZS	scaffoldA10	23213882	23313881	100000	594
BnaWGS-1007	BnaA10G0244800ZS	scaffoldA10	23223882	23323881	100000	618
BnaWGS-1007	BnaA10G0244800ZS	scaffoldA10	23233882	23333881	100000	680
BnaWGS-1007	BnaA10G0244800ZS	scaffoldA10	23243882	23343881	100000	825
BnaWGS-1007	BnaA10G0244800ZS	scaffoldA10	23253882	23353881	100000	878
BnaWGS-1007	BnaA10G0244800ZS	scaffoldA10	23263882	23363881	100000	800
BnaWGS-1007	BnaA10G0244800ZS	scaffoldA10	23273882	23373881	100000	785
BnaWGS-1007	BnaA10G0244800ZS	scaffoldA10	23283882	23383881	100000	747
BnaWGS-1007	BnaA10G0244800ZS	scaffoldA10	23293882	23393881	100000	670
BnaWGS-1007	BnaA10G0244800ZS	scaffoldA10	23303882	23403881	100000	665
BnaWGS-1007	BnaA10G0244800ZS	scaffoldA10	23313882	23413881	100000	668
BnaWGS-1007	BnaA10G0244800ZS	scaffoldA10	23323882	23423881	100000	605
BnaWGS-1007	BnaA10G0244800ZS	scaffoldA10	23333882	23433881	100000	587
BnaWGS-1007	BnaA10G0244800ZS	scaffoldA10	23343882	23443881	100000	494
BnaWGS-1007	BnaA10G0244800ZS	scaffoldA10	23353882	23453881	100000	461
BnaWGS-1007	BnaA10G0244800ZS	scaffoldA10	23363882	23463881	100000	497
BnaWGS-1007	BnaA10G0244800ZS	scaffoldA10	23373882	23473881	100000	521
BnaWGS-1007	BnaA10G0244800ZS	scaffoldA10	23383882	23483881	100000	603
BnaWGS-1007	BnaA10G0244800ZS	scaffoldA10	23393882	23493881	100000	685
BnaWGS-1007	BnaA10G0244800ZS	scaffoldA10	23403882	23503881	100000	733
BnaWGS-1007	BnaA10G0244800ZS	scaffoldA10	23413882	23513881	100000	872
BnaWGS-1007	BnaA10G0244800ZS	scaffoldA10	23423882	23523881	100000	894
BnaWGS-1007	BnaA10G0244800ZS	scaffoldA10	23433882	23533881	100000	872
BnaWGS-1007	BnaA10G0244800ZS	scaffoldA10	23443882	23543881	100000	1015
BnaWGS-1007	BnaA10G0244800ZS	scaffoldA10	23453882	23553881	100000	1068
BnaWGS-1007	BnaA10G0244800ZS	scaffoldA10	23463882	23563881	100000	1090
BnaWGS-1007	BnaA10G0244800ZS	scaffoldA10	23473882	23573881	100000	1074
BnaWGS-1007	BnaA10G0244800ZS	scaffoldA10	23483882	23583881	100000	1002
BnaWGS-1007	BnaA10G0244800ZS	scaffoldA10	23493882	23593881	100000	978
BnaWGS-1007	BnaA10G0244800ZS	scaffoldA10	23503882	23603881	100000	999
BnaWGS-1007	BnaA10G0244800ZS	scaffoldA10	23513882	23613881	100000	831
BnaWGS-1007	BnaA10G0244800ZS	scaffoldA10	23523882	23623881	100000	851
BnaWGS-1007	BnaA10G0244800ZS	scaffoldA10	23533882	23633881	100000	934
BnaWGS-1007	BnaA10G0244800ZS	scaffoldA10	23543882	23643881	100000	830
BnaWGS-1007	BnaA10G0244800ZS	scaffoldA10	23553882	23653881	100000	856
BnaWGS-1007	BnaA10G0244800ZS	scaffoldA10	23563882	23663881	100000	890
BnaWGS-1007	BnaA10G0244800ZS	scaffoldA10	23573882	23673881	100000	1076
BnaWGS-1007	BnaA10G0244800ZS	scaffoldA10	23583882	23683881	100000	1220
BnaWGS-1007	BnaA10G0244800ZS	scaffoldA10	23593882	23693881	100000	1194
BnaWGS-1007	BnaA10G0244800ZS	scaffoldA10	23603882	23703881	100000	1129
BnaWGS-1007	BnaA10G0244800ZS	scaffoldA10	23613882	23713881	100000	1189
BnaWGS-1007	BnaA10G0244800ZS	scaffoldA10	23623882	23723881	100000	1142
BnaWGS-1007	BnaA10G0244800ZS	scaffoldA10	23633882	23733881	100000	1113
BnaWGS-1007	BnaA10G0244800ZS	scaffoldA10	23643882	23743881	100000	1172
BnaWGS-1007	BnaA10G0244800ZS	scaffoldA10	23653882	23753881	100000	1237
BnaWGS-1007	BnaA10G0244800ZS	scaffoldA10	23663882	23763881	100000	1262
BnaWGS-1007	BnaA10G0244800ZS	scaffoldA10	23673882	23773881	100000	1134
BnaWGS-1007	BnaA10G0244800ZS	scaffoldA10	23683882	23783881	100000	1076
BnaWGS-1007	BnaA10G0244800ZS	scaffoldA10	23693882	23793881	100000	1115
BnaWGS-1007	BnaA10G0244800ZS	scaffoldA10	23703882	23803881	100000	1167
BnaWGS-1007	BnaA10G0244800ZS	scaffoldA10	23713882	23813881	100000	1176
BnaWGS-1007	BnaA10G0244800ZS	scaffoldA10	23723882	23823881	100000	1178
BnaWGS-1007	BnaA10G0244800ZS	scaffoldA10	23733882	23833881	100000	1122
BnaWGS-1007	BnaA10G0244800ZS	scaffoldA10	23743882	23843881	100000	998
BnaWGS-1007	BnaA10G0244800ZS	scaffoldA10	23753882	23853881	100000	913

For Review Only

BnaWGS-1007	BnaA10G0244800ZS	scaffoldA10	23763882	23863881	100000	916
BnaWGS-1007	BnaA10G0244800ZS	scaffoldA10	23773882	23873881	100000	848
BnaWGS-1007	BnaA10G0244800ZS	scaffoldA10	23783882	23883881	100000	793
BnaWGS-1007	BnaA10G0244800ZS	scaffoldA10	23793882	23893881	100000	744
BnaWGS-1007	BnaA10G0244800ZS	scaffoldA10	23803882	23903881	100000	684
BnaWGS-1007	BnaA10G0244800ZS	scaffoldA10	23813882	23913881	100000	623
BnaWGS-1007	BnaA10G0244800ZS	scaffoldA10	23823882	23923881	100000	567
BnaWGS-1007	BnaA10G0244800ZS	scaffoldA10	23833882	23933881	100000	546
BnaWGS-1007	BnaA10G0244800ZS	scaffoldA10	23843882	23943881	100000	580
BnaWGS-1007	BnaA10G0244800ZS	scaffoldA10	23853882	23953881	100000	513
BnaWGS-1007	BnaA10G0244800ZS	scaffoldA10	23863882	23963881	100000	409
BnaWGS-1007	BnaA10G0244800ZS	scaffoldA10	23873882	23973881	100000	432
BnaWGS-1007	BnaA10G0244800ZS	scaffoldA10	23883882	23983881	100000	588
BnaWGS-1007	BnaA10G0244800ZS	scaffoldA10	23893882	23993881	100000	585
BnaWGS-1007	BnaA10G0244800ZS	scaffoldA10	23903882	24003881	100000	620
BnaWGS-1007	BnaA10G0244800ZS	scaffoldA10	23913882	24013881	100000	659
BnaWGS-1007	BnaA10G0244800ZS	scaffoldA10	23923882	24023881	100000	750
BnaWGS-1007	BnaA10G0244800ZS	scaffoldA10	23933882	24033881	100000	747
BnaWGS-1007	BnaA10G0244800ZS	scaffoldA10	23943882	24043881	100000	823
BnaWGS-1007	BnaA10G0244800ZS	scaffoldA10	23953882	24053881	100000	831
BnaWGS-1007	BnaA10G0244800ZS	scaffoldA10	23963882	24063881	100000	919
BnaWGS-1007	BnaA10G0244800ZS	scaffoldA10	23973882	24073881	100000	1039
BnaWGS-1007	BnaA10G0244800ZS	scaffoldA10	23983882	24083881	100000	1020
BnaWGS-1007	BnaA10G0244800ZS	scaffoldA10	23993882	24093881	100000	1197
BnaWGS-1007	BnaA10G0244800ZS	scaffoldA10	24003882	24103881	100000	1342
BnaWGS-1007	BnaA10G0244800ZS	scaffoldA10	24013882	24113881	100000	1400
BnaWGS-1007	BnaA10G0244800ZS	scaffoldA10	24023882	24123881	100000	1506
BnaWGS-1007	BnaA10G0244800ZS	scaffoldA10	24033882	24133881	100000	1553
BnaWGS-1007	BnaA10G0244800ZS	scaffoldA10	24043882	24143881	100000	1523
BnaWGS-1007	BnaA10G0244800ZS	scaffoldA10	24053882	24153881	100000	1592
BnaWGS-1007	BnaA10G0244800ZS	scaffoldA10	24063882	24163881	100000	1607
BnaWGS-1007	BnaA10G0244800ZS	scaffoldA10	24073882	24173881	100000	1645
BnaWGS-1007	BnaA10G0244800ZS	scaffoldA10	24083882	24183881	100000	1712
BnaWGS-1007	BnaA10G0244800ZS	scaffoldA10	24093882	24193881	100000	1794
BnaWGS-1007	BnaA10G0244800ZS	scaffoldA10	24103882	24203881	100000	1636
BnaWGS-1007	BnaA10G0244800ZS	scaffoldA10	24113882	24213881	100000	1549
BnaWGS-1007	BnaA10G0244800ZS	scaffoldA10	24123882	24223881	100000	1535
BnaWGS-1007	BnaA10G0244800ZS	scaffoldA10	24133882	24233881	100000	1641
BnaWGS-1007	BnaA10G0244800ZS	scaffoldA10	24143882	24243881	100000	1663
BnaWGS-1007	BnaA10G0244800ZS	scaffoldA10	24153882	24253881	100000	1761
BnaWGS-1007	BnaA10G0244800ZS	scaffoldA10	24163882	24263881	100000	1778
BnaWGS-1007	BnaA10G0244800ZS	scaffoldA10	24173882	24273881	100000	1804
BnaWGS-1007	BnaA10G0244800ZS	scaffoldA10	24183882	24283881	100000	1655
BnaWGS-1007	BnaA10G0244800ZS	scaffoldA10	24193882	24293881	100000	1439
BnaWGS-1007	BnaA10G0244800ZS	scaffoldA10	24203882	24303881	100000	1554
BnaWGS-1007	BnaA10G0244800ZS	scaffoldA10	24213882	24313881	100000	1699
BnaWGS-1007	BnaA10G0244800ZS	scaffoldA10	24223882	24323881	100000	1713
BnaWGS-1007	BnaA10G0244800ZS	scaffoldA10	24233882	24333881	100000	1662
BnaWGS-1007	BnaA10G0244800ZS	scaffoldA10	24243882	24343881	100000	1729
BnaWGS-1007	BnaA10G0244800ZS	scaffoldA10	24253882	24353881	100000	1748
BnaWGS-1007	BnaA10G0244800ZS	scaffoldA10	24263882	24363881	100000	1674
BnaWGS-1007	BnaA10G0244800ZS	scaffoldA10	24273882	24373881	100000	1603
BnaWGS-1007	BnaA10G0244800ZS	scaffoldA10	24283882	24383881	100000	1647
BnaWGS-1007	BnaA10G0244800ZS	scaffoldA10	24293882	24393881	100000	1623
BnaWGS-1007	BnaA10G0244800ZS	scaffoldA10	24303882	24403881	100000	1448
BnaWGS-1007	BnaA10G0244800ZS	scaffoldA10	24313882	24413881	100000	1427
BnaWGS-1007	BnaA10G0244800ZS	scaffoldA10	24323882	24423881	100000	1434
BnaWGS-1007	BnaA10G0244800ZS	scaffoldA10	24333882	24433881	100000	1449
BnaWGS-1007	BnaA10G0244800ZS	scaffoldA10	24343882	24443881	100000	1324
BnaWGS-1007	BnaA10G0244800ZS	scaffoldA10	24353882	24453881	100000	1165

For Review Only

BnaWGS-1007	BnaA10G0244800ZS	scaffoldA10	24363882	24463881	100000	1306
BnaWGS-1007	BnaA10G0244800ZS	scaffoldA10	24373882	24473881	100000	1314
BnaWGS-1007	BnaA10G0244800ZS	scaffoldA10	24383882	24483881	100000	1244
BnaWGS-1007	BnaA10G0244800ZS	scaffoldA10	24393882	24493881	100000	1326
BnaWGS-1007	BnaA10G0244800ZS	scaffoldA10	24403882	24503881	100000	1382
BnaWGS-1007	BnaA10G0244800ZS	scaffoldA10	24413882	24513881	100000	1286
BnaWGS-1007	BnaA10G0244800ZS	scaffoldA10	24423882	24523881	100000	1126
BnaWGS-1007	BnaA10G0244800ZS	scaffoldA10	24433882	24533881	100000	1033
BnaWGS-1007	BnaA10G0244800ZS	scaffoldA10	24443882	24543881	100000	1049
BnaWGS-1007	BnaA10G0244800ZS	scaffoldA10	24453882	24553881	100000	1114
BnaWGS-1007	BnaA10G0244800ZS	scaffoldA10	24463882	24563881	100000	1052
BnaWGS-1007	BnaA10G0244800ZS	scaffoldA10	24473882	24573881	100000	1023
BnaWGS-1007	BnaA10G0244800ZS	scaffoldA10	24483882	24583881	100000	1056
BnaWGS-1007	BnaA10G0244800ZS	scaffoldA10	24493882	24593881	100000	980
BnaWGS-1007	BnaA10G0244800ZS	scaffoldA10	24503882	24603881	100000	1008
BnaWGS-1007	BnaA10G0244800ZS	scaffoldA10	24513882	24613881	100000	1025
BnaWGS-1007	BnaA10G0244800ZS	scaffoldA10	24523882	24623881	100000	1086
BnaWGS-1007	BnaA10G0244800ZS	scaffoldA10	24533882	24633881	100000	1145
BnaWGS-1007	BnaA10G0244800ZS	scaffoldA10	24543882	24643881	100000	1182
BnaWGS-1007	BnaA10G0244800ZS	scaffoldA10	24553882	24653881	100000	1172
BnaWGS-1007	BnaA10G0244800ZS	scaffoldA10	24563882	24663881	100000	1138
BnaWGS-1007	BnaA10G0244800ZS	scaffoldA10	24573882	24673881	100000	1024
BnaWGS-1007	BnaA10G0244800ZS	scaffoldA10	24583882	24683881	100000	1010
BnaWGS-1007	BnaA10G0244800ZS	scaffoldA10	24593882	24693881	100000	1040
BnaWGS-1007	BnaA10G0244800ZS	scaffoldA10	24603882	24703881	100000	1011
BnaWGS-1007	BnaA10G0244800ZS	scaffoldA10	24613882	24713881	100000	980
BnaWGS-1007	BnaA10G0244800ZS	scaffoldA10	24623882	24723881	100000	910
BnaWGS-1007	BnaA10G0244800ZS	scaffoldA10	24633882	24733881	100000	915
BnaWGS-1007	BnaA10G0244800ZS	scaffoldA10	24643882	24743881	100000	913
BnaWGS-1007	BnaA10G0244800ZS	scaffoldA10	24653882	24753881	100000	932
BnaWGS-1007	BnaA10G0244800ZS	scaffoldA10	24663882	24763881	100000	838
BnaWGS-1007	BnaA10G0244800ZS	scaffoldA10	24673882	24773881	100000	802
BnaWGS-1007	BnaA10G0244800ZS	scaffoldA10	24683882	24783881	100000	849
BnaWGS-1007	BnaA10G0244800ZS	scaffoldA10	24693882	24793881	100000	845
BnaWGS-1007	BnaA10G0244800ZS	scaffoldA10	24703882	24803881	100000	783
BnaWGS-1007	BnaA10G0244800ZS	scaffoldA10	24713882	24813881	100000	778
BnaWGS-1007	BnaA10G0244800ZS	scaffoldA10	24723882	24823881	100000	926
BnaWGS-1007	BnaA10G0244800ZS	scaffoldA10	24733882	24833881	100000	935
BnaWGS-1007	BnaA10G0244800ZS	scaffoldA10	24743882	24843881	100000	961
BnaWGS-1007	BnaA10G0244800ZS	scaffoldA10	24753882	24853881	100000	910
BnaWGS-1007	BnaA10G0244800ZS	scaffoldA10	24763882	24863881	100000	923
BnaWGS-1007	BnaA10G0244800ZS	scaffoldA10	24773882	24873881	100000	1033
BnaWGS-1007	BnaA10G0244800ZS	scaffoldA10	24783882	24883881	100000	932
BnaWGS-1007	BnaA10G0244800ZS	scaffoldA10	24793882	24893881	100000	899
BnaWGS-1007	BnaA10G0244800ZS	scaffoldA10	24803882	24903881	100000	1034
BnaWGS-1007	BnaA10G0244800ZS	scaffoldA10	24813882	24913881	100000	1090
BnaWGS-1007	BnaA10G0244800ZS	scaffoldA10	24823882	24923881	100000	1222
BnaWGS-1007	BnaA10G0244800ZS	scaffoldA10	24833882	24933881	100000	1312
BnaWGS-1007	BnaA10G0244800ZS	scaffoldA10	24843882	24943881	100000	1248
BnaWGS-1007	BnaA10G0244800ZS	scaffoldA10	24853882	24947904	94023	1187
BnaWGS-655	BnaA10G0244800ZS	scaffoldA10	22943882	23043881	100000	663
BnaWGS-655	BnaA10G0244800ZS	scaffoldA10	22953882	23053881	100000	624
BnaWGS-655	BnaA10G0244800ZS	scaffoldA10	22963882	23063881	100000	607
BnaWGS-655	BnaA10G0244800ZS	scaffoldA10	22973882	23073881	100000	600
BnaWGS-655	BnaA10G0244800ZS	scaffoldA10	22983882	23083881	100000	564
BnaWGS-655	BnaA10G0244800ZS	scaffoldA10	22993882	23093881	100000	591
BnaWGS-655	BnaA10G0244800ZS	scaffoldA10	23003882	23103881	100000	625
BnaWGS-655	BnaA10G0244800ZS	scaffoldA10	23013882	23113881	100000	625
BnaWGS-655	BnaA10G0244800ZS	scaffoldA10	23023882	23123881	100000	616
BnaWGS-655	BnaA10G0244800ZS	scaffoldA10	23033882	23133881	100000	718

For Review Only

BnaWGS-655	BnaA10G0244800ZS	scaffoldA10	23043882	23143881	100000	856
BnaWGS-655	BnaA10G0244800ZS	scaffoldA10	23053882	23153881	100000	1085
BnaWGS-655	BnaA10G0244800ZS	scaffoldA10	23063882	23163881	100000	1274
BnaWGS-655	BnaA10G0244800ZS	scaffoldA10	23073882	23173881	100000	1354
BnaWGS-655	BnaA10G0244800ZS	scaffoldA10	23083882	23183881	100000	1433
BnaWGS-655	BnaA10G0244800ZS	scaffoldA10	23093882	23193881	100000	1528
BnaWGS-655	BnaA10G0244800ZS	scaffoldA10	23103882	23203881	100000	1613
BnaWGS-655	BnaA10G0244800ZS	scaffoldA10	23113882	23213881	100000	1821
BnaWGS-655	BnaA10G0244800ZS	scaffoldA10	23123882	23223881	100000	1914
BnaWGS-655	BnaA10G0244800ZS	scaffoldA10	23133882	23233881	100000	1906
BnaWGS-655	BnaA10G0244800ZS	scaffoldA10	23143882	23243881	100000	1740
BnaWGS-655	BnaA10G0244800ZS	scaffoldA10	23153882	23253881	100000	1621
BnaWGS-655	BnaA10G0244800ZS	scaffoldA10	23163882	23263881	100000	1493
BnaWGS-655	BnaA10G0244800ZS	scaffoldA10	23173882	23273881	100000	1412
BnaWGS-655	BnaA10G0244800ZS	scaffoldA10	23183882	23283881	100000	1338
BnaWGS-655	BnaA10G0244800ZS	scaffoldA10	23193882	23293881	100000	1150
BnaWGS-655	BnaA10G0244800ZS	scaffoldA10	23203882	23303881	100000	1074
BnaWGS-655	BnaA10G0244800ZS	scaffoldA10	23213882	23313881	100000	900
BnaWGS-655	BnaA10G0244800ZS	scaffoldA10	23223882	23323881	100000	928
BnaWGS-655	BnaA10G0244800ZS	scaffoldA10	23233882	23333881	100000	897
BnaWGS-655	BnaA10G0244800ZS	scaffoldA10	23243882	23343881	100000	1111
BnaWGS-655	BnaA10G0244800ZS	scaffoldA10	23253882	23353881	100000	1092
BnaWGS-655	BnaA10G0244800ZS	scaffoldA10	23263882	23363881	100000	1068
BnaWGS-655	BnaA10G0244800ZS	scaffoldA10	23273882	23373881	100000	1060
BnaWGS-655	BnaA10G0244800ZS	scaffoldA10	23283882	23383881	100000	1028
BnaWGS-655	BnaA10G0244800ZS	scaffoldA10	23293882	23393881	100000	1068
BnaWGS-655	BnaA10G0244800ZS	scaffoldA10	23303882	23403881	100000	1068
BnaWGS-655	BnaA10G0244800ZS	scaffoldA10	23313882	23413881	100000	1032
BnaWGS-655	BnaA10G0244800ZS	scaffoldA10	23323882	23423881	100000	957
BnaWGS-655	BnaA10G0244800ZS	scaffoldA10	23333882	23433881	100000	901
BnaWGS-655	BnaA10G0244800ZS	scaffoldA10	23343882	23443881	100000	726
BnaWGS-655	BnaA10G0244800ZS	scaffoldA10	23353882	23453881	100000	661
BnaWGS-655	BnaA10G0244800ZS	scaffoldA10	23363882	23463881	100000	689
BnaWGS-655	BnaA10G0244800ZS	scaffoldA10	23373882	23473881	100000	763
BnaWGS-655	BnaA10G0244800ZS	scaffoldA10	23383882	23483881	100000	866
BnaWGS-655	BnaA10G0244800ZS	scaffoldA10	23393882	23493881	100000	880
BnaWGS-655	BnaA10G0244800ZS	scaffoldA10	23403882	23503881	100000	901
BnaWGS-655	BnaA10G0244800ZS	scaffoldA10	23413882	23513881	100000	1082
BnaWGS-655	BnaA10G0244800ZS	scaffoldA10	23423882	23523881	100000	1122
BnaWGS-655	BnaA10G0244800ZS	scaffoldA10	23433882	23533881	100000	1098
BnaWGS-655	BnaA10G0244800ZS	scaffoldA10	23443882	23543881	100000	1317
BnaWGS-655	BnaA10G0244800ZS	scaffoldA10	23453882	23553881	100000	1372
BnaWGS-655	BnaA10G0244800ZS	scaffoldA10	23463882	23563881	100000	1385
BnaWGS-655	BnaA10G0244800ZS	scaffoldA10	23473882	23573881	100000	1360
BnaWGS-655	BnaA10G0244800ZS	scaffoldA10	23483882	23583881	100000	1289
BnaWGS-655	BnaA10G0244800ZS	scaffoldA10	23493882	23593881	100000	1326
BnaWGS-655	BnaA10G0244800ZS	scaffoldA10	23503882	23603881	100000	1368
BnaWGS-655	BnaA10G0244800ZS	scaffoldA10	23513882	23613881	100000	1161
BnaWGS-655	BnaA10G0244800ZS	scaffoldA10	23523882	23623881	100000	1220
BnaWGS-655	BnaA10G0244800ZS	scaffoldA10	23533882	23633881	100000	1326
BnaWGS-655	BnaA10G0244800ZS	scaffoldA10	23543882	23643881	100000	1146
BnaWGS-655	BnaA10G0244800ZS	scaffoldA10	23553882	23653881	100000	1143
BnaWGS-655	BnaA10G0244800ZS	scaffoldA10	23563882	23663881	100000	1193
BnaWGS-655	BnaA10G0244800ZS	scaffoldA10	23573882	23673881	100000	1432
BnaWGS-655	BnaA10G0244800ZS	scaffoldA10	23583882	23683881	100000	1621
BnaWGS-655	BnaA10G0244800ZS	scaffoldA10	23593882	23693881	100000	1556
BnaWGS-655	BnaA10G0244800ZS	scaffoldA10	23603882	23703881	100000	1568
BnaWGS-655	BnaA10G0244800ZS	scaffoldA10	23613882	23713881	100000	1681
BnaWGS-655	BnaA10G0244800ZS	scaffoldA10	23623882	23723881	100000	1593
BnaWGS-655	BnaA10G0244800ZS	scaffoldA10	23633882	23733881	100000	1557

BnaWGS-655	BnaA10G0244800ZS	scaffoldA10	23643882	23743881	100000	1566
BnaWGS-655	BnaA10G0244800ZS	scaffoldA10	23653882	23753881	100000	1693
BnaWGS-655	BnaA10G0244800ZS	scaffoldA10	23663882	23763881	100000	1712
BnaWGS-655	BnaA10G0244800ZS	scaffoldA10	23673882	23773881	100000	1548
BnaWGS-655	BnaA10G0244800ZS	scaffoldA10	23683882	23783881	100000	1467
BnaWGS-655	BnaA10G0244800ZS	scaffoldA10	23693882	23793881	100000	1524
BnaWGS-655	BnaA10G0244800ZS	scaffoldA10	23703882	23803881	100000	1550
BnaWGS-655	BnaA10G0244800ZS	scaffoldA10	23713882	23813881	100000	1510
BnaWGS-655	BnaA10G0244800ZS	scaffoldA10	23723882	23823881	100000	1516
BnaWGS-655	BnaA10G0244800ZS	scaffoldA10	23733882	23833881	100000	1453
BnaWGS-655	BnaA10G0244800ZS	scaffoldA10	23743882	23843881	100000	1423
BnaWGS-655	BnaA10G0244800ZS	scaffoldA10	23753882	23853881	100000	1328
BnaWGS-655	BnaA10G0244800ZS	scaffoldA10	23763882	23863881	100000	1360
BnaWGS-655	BnaA10G0244800ZS	scaffoldA10	23773882	23873881	100000	1230
BnaWGS-655	BnaA10G0244800ZS	scaffoldA10	23783882	23883881	100000	1158
BnaWGS-655	BnaA10G0244800ZS	scaffoldA10	23793882	23893881	100000	1126
BnaWGS-655	BnaA10G0244800ZS	scaffoldA10	23803882	23903881	100000	1005
BnaWGS-655	BnaA10G0244800ZS	scaffoldA10	23813882	23913881	100000	931
BnaWGS-655	BnaA10G0244800ZS	scaffoldA10	23823882	23923881	100000	861
BnaWGS-655	BnaA10G0244800ZS	scaffoldA10	23833882	23933881	100000	831
BnaWGS-655	BnaA10G0244800ZS	scaffoldA10	23843882	23943881	100000	823
BnaWGS-655	BnaA10G0244800ZS	scaffoldA10	23853882	23953881	100000	728
BnaWGS-655	BnaA10G0244800ZS	scaffoldA10	23863882	23963881	100000	597
BnaWGS-655	BnaA10G0244800ZS	scaffoldA10	23873882	23973881	100000	663
BnaWGS-655	BnaA10G0244800ZS	scaffoldA10	23883882	23983881	100000	787
BnaWGS-655	BnaA10G0244800ZS	scaffoldA10	23893882	23993881	100000	776
BnaWGS-655	BnaA10G0244800ZS	scaffoldA10	23903882	24003881	100000	794
BnaWGS-655	BnaA10G0244800ZS	scaffoldA10	23913882	24013881	100000	862
BnaWGS-655	BnaA10G0244800ZS	scaffoldA10	23923882	24023881	100000	993
BnaWGS-655	BnaA10G0244800ZS	scaffoldA10	23933882	24033881	100000	1010
BnaWGS-655	BnaA10G0244800ZS	scaffoldA10	23943882	24043881	100000	1121
BnaWGS-655	BnaA10G0244800ZS	scaffoldA10	23953882	24053881	100000	1138
BnaWGS-655	BnaA10G0244800ZS	scaffoldA10	23963882	24063881	100000	1234
BnaWGS-655	BnaA10G0244800ZS	scaffoldA10	23973882	24073881	100000	1325
BnaWGS-655	BnaA10G0244800ZS	scaffoldA10	23983882	24083881	100000	1322
BnaWGS-655	BnaA10G0244800ZS	scaffoldA10	23993882	24093881	100000	1427
BnaWGS-655	BnaA10G0244800ZS	scaffoldA10	24003882	24103881	100000	1572
BnaWGS-655	BnaA10G0244800ZS	scaffoldA10	24013882	24113881	100000	1616
BnaWGS-655	BnaA10G0244800ZS	scaffoldA10	24023882	24123881	100000	1711
BnaWGS-655	BnaA10G0244800ZS	scaffoldA10	24033882	24133881	100000	1737
BnaWGS-655	BnaA10G0244800ZS	scaffoldA10	24043882	24143881	100000	1725
BnaWGS-655	BnaA10G0244800ZS	scaffoldA10	24053882	24153881	100000	1796
BnaWGS-655	BnaA10G0244800ZS	scaffoldA10	24063882	24163881	100000	1814
BnaWGS-655	BnaA10G0244800ZS	scaffoldA10	24073882	24173881	100000	1836
BnaWGS-655	BnaA10G0244800ZS	scaffoldA10	24083882	24183881	100000	1993
BnaWGS-655	BnaA10G0244800ZS	scaffoldA10	24093882	24193881	100000	2122
BnaWGS-655	BnaA10G0244800ZS	scaffoldA10	24103882	24203881	100000	1993
BnaWGS-655	BnaA10G0244800ZS	scaffoldA10	24113882	24213881	100000	1887
BnaWGS-655	BnaA10G0244800ZS	scaffoldA10	24123882	24223881	100000	1926
BnaWGS-655	BnaA10G0244800ZS	scaffoldA10	24133882	24233881	100000	2032
BnaWGS-655	BnaA10G0244800ZS	scaffoldA10	24143882	24243881	100000	2093
BnaWGS-655	BnaA10G0244800ZS	scaffoldA10	24153882	24253881	100000	2152
BnaWGS-655	BnaA10G0244800ZS	scaffoldA10	24163882	24263881	100000	2159
BnaWGS-655	BnaA10G0244800ZS	scaffoldA10	24173882	24273881	100000	2190
BnaWGS-655	BnaA10G0244800ZS	scaffoldA10	24183882	24283881	100000	2032
BnaWGS-655	BnaA10G0244800ZS	scaffoldA10	24193882	24293881	100000	1863
BnaWGS-655	BnaA10G0244800ZS	scaffoldA10	24203882	24303881	100000	1936
BnaWGS-655	BnaA10G0244800ZS	scaffoldA10	24213882	24313881	100000	2055
BnaWGS-655	BnaA10G0244800ZS	scaffoldA10	24223882	24323881	100000	1956
BnaWGS-655	BnaA10G0244800ZS	scaffoldA10	24233882	24333881	100000	1961

For Review Only

BnaWGS-655	BnaA10G0244800ZS	scaffoldA10	24243882	24343881	100000	2003
BnaWGS-655	BnaA10G0244800ZS	scaffoldA10	24253882	24353881	100000	2142
BnaWGS-655	BnaA10G0244800ZS	scaffoldA10	24263882	24363881	100000	2073
BnaWGS-655	BnaA10G0244800ZS	scaffoldA10	24273882	24373881	100000	2050
BnaWGS-655	BnaA10G0244800ZS	scaffoldA10	24283882	24383881	100000	2054
BnaWGS-655	BnaA10G0244800ZS	scaffoldA10	24293882	24393881	100000	1969
BnaWGS-655	BnaA10G0244800ZS	scaffoldA10	24303882	24403881	100000	1811
BnaWGS-655	BnaA10G0244800ZS	scaffoldA10	24313882	24413881	100000	1845
BnaWGS-655	BnaA10G0244800ZS	scaffoldA10	24323882	24423881	100000	1931
BnaWGS-655	BnaA10G0244800ZS	scaffoldA10	24333882	24433881	100000	1929
BnaWGS-655	BnaA10G0244800ZS	scaffoldA10	24343882	24443881	100000	1795
BnaWGS-655	BnaA10G0244800ZS	scaffoldA10	24353882	24453881	100000	1546
BnaWGS-655	BnaA10G0244800ZS	scaffoldA10	24363882	24463881	100000	1749
BnaWGS-655	BnaA10G0244800ZS	scaffoldA10	24373882	24473881	100000	1713
BnaWGS-655	BnaA10G0244800ZS	scaffoldA10	24383882	24483881	100000	1610
BnaWGS-655	BnaA10G0244800ZS	scaffoldA10	24393882	24493881	100000	1706
BnaWGS-655	BnaA10G0244800ZS	scaffoldA10	24403882	24503881	100000	1799
BnaWGS-655	BnaA10G0244800ZS	scaffoldA10	24413882	24513881	100000	1675
BnaWGS-655	BnaA10G0244800ZS	scaffoldA10	24423882	24523881	100000	1463
BnaWGS-655	BnaA10G0244800ZS	scaffoldA10	24433882	24533881	100000	1338
BnaWGS-655	BnaA10G0244800ZS	scaffoldA10	24443882	24543881	100000	1326
BnaWGS-655	BnaA10G0244800ZS	scaffoldA10	24453882	24553881	100000	1472
BnaWGS-655	BnaA10G0244800ZS	scaffoldA10	24463882	24563881	100000	1372
BnaWGS-655	BnaA10G0244800ZS	scaffoldA10	24473882	24573881	100000	1397
BnaWGS-655	BnaA10G0244800ZS	scaffoldA10	24483882	24583881	100000	1425
BnaWGS-655	BnaA10G0244800ZS	scaffoldA10	24493882	24593881	100000	1317
BnaWGS-655	BnaA10G0244800ZS	scaffoldA10	24503882	24603881	100000	1311
BnaWGS-655	BnaA10G0244800ZS	scaffoldA10	24513882	24613881	100000	1361
BnaWGS-655	BnaA10G0244800ZS	scaffoldA10	24523882	24623881	100000	1473
BnaWGS-655	BnaA10G0244800ZS	scaffoldA10	24533882	24633881	100000	1514
BnaWGS-655	BnaA10G0244800ZS	scaffoldA10	24543882	24643881	100000	1529
BnaWGS-655	BnaA10G0244800ZS	scaffoldA10	24553882	24653881	100000	1509
BnaWGS-655	BnaA10G0244800ZS	scaffoldA10	24563882	24663881	100000	1412
BnaWGS-655	BnaA10G0244800ZS	scaffoldA10	24573882	24673881	100000	1274
BnaWGS-655	BnaA10G0244800ZS	scaffoldA10	24583882	24683881	100000	1340
BnaWGS-655	BnaA10G0244800ZS	scaffoldA10	24593882	24693881	100000	1438
BnaWGS-655	BnaA10G0244800ZS	scaffoldA10	24603882	24703881	100000	1443
BnaWGS-655	BnaA10G0244800ZS	scaffoldA10	24613882	24713881	100000	1383
BnaWGS-655	BnaA10G0244800ZS	scaffoldA10	24623882	24723881	100000	1273
BnaWGS-655	BnaA10G0244800ZS	scaffoldA10	24633882	24733881	100000	1302
BnaWGS-655	BnaA10G0244800ZS	scaffoldA10	24643882	24743881	100000	1330
BnaWGS-655	BnaA10G0244800ZS	scaffoldA10	24653882	24753881	100000	1341
BnaWGS-655	BnaA10G0244800ZS	scaffoldA10	24663882	24763881	100000	1253
BnaWGS-655	BnaA10G0244800ZS	scaffoldA10	24673882	24773881	100000	1181
BnaWGS-655	BnaA10G0244800ZS	scaffoldA10	24683882	24783881	100000	1170
BnaWGS-655	BnaA10G0244800ZS	scaffoldA10	24693882	24793881	100000	1246
BnaWGS-655	BnaA10G0244800ZS	scaffoldA10	24703882	24803881	100000	1228
BnaWGS-655	BnaA10G0244800ZS	scaffoldA10	24713882	24813881	100000	1307
BnaWGS-655	BnaA10G0244800ZS	scaffoldA10	24723882	24823881	100000	1466
BnaWGS-655	BnaA10G0244800ZS	scaffoldA10	24733882	24833881	100000	1448
BnaWGS-655	BnaA10G0244800ZS	scaffoldA10	24743882	24843881	100000	1519
BnaWGS-655	BnaA10G0244800ZS	scaffoldA10	24753882	24853881	100000	1428
BnaWGS-655	BnaA10G0244800ZS	scaffoldA10	24763882	24863881	100000	1451
BnaWGS-655	BnaA10G0244800ZS	scaffoldA10	24773882	24873881	100000	1607
BnaWGS-655	BnaA10G0244800ZS	scaffoldA10	24783882	24883881	100000	1467
BnaWGS-655	BnaA10G0244800ZS	scaffoldA10	24793882	24893881	100000	1285
BnaWGS-655	BnaA10G0244800ZS	scaffoldA10	24803882	24903881	100000	1392
BnaWGS-655	BnaA10G0244800ZS	scaffoldA10	24813882	24913881	100000	1477
BnaWGS-655	BnaA10G0244800ZS	scaffoldA10	24823882	24923881	100000	1636
BnaWGS-655	BnaA10G0244800ZS	scaffoldA10	24833882	24933881	100000	1790

For Review Only

BnaWGS-655	BnaA10G0244800ZS	scaffoldA10	24843882	24943881	100000	1654
BnaWGS-655	BnaA10G0244800ZS	scaffoldA10	24853882	24947904	94023	1580
BnaWGS-655	BnaA10G0244800ZS	scaffoldA10	22943882	23043881	100000	663
BnaWGS-655	BnaA10G0244800ZS	scaffoldA10	22953882	23053881	100000	624
BnaWGS-655	BnaA10G0244800ZS	scaffoldA10	22963882	23063881	100000	607
BnaWGS-655	BnaA10G0244800ZS	scaffoldA10	22973882	23073881	100000	600
BnaWGS-655	BnaA10G0244800ZS	scaffoldA10	22983882	23083881	100000	564
BnaWGS-655	BnaA10G0244800ZS	scaffoldA10	22993882	23093881	100000	591
BnaWGS-655	BnaA10G0244800ZS	scaffoldA10	23003882	23103881	100000	625
BnaWGS-655	BnaA10G0244800ZS	scaffoldA10	23013882	23113881	100000	625
BnaWGS-655	BnaA10G0244800ZS	scaffoldA10	23023882	23123881	100000	616
BnaWGS-655	BnaA10G0244800ZS	scaffoldA10	23033882	23133881	100000	718
BnaWGS-655	BnaA10G0244800ZS	scaffoldA10	23043882	23143881	100000	856
BnaWGS-655	BnaA10G0244800ZS	scaffoldA10	23053882	23153881	100000	1085
BnaWGS-655	BnaA10G0244800ZS	scaffoldA10	23063882	23163881	100000	1274
BnaWGS-655	BnaA10G0244800ZS	scaffoldA10	23073882	23173881	100000	1354
BnaWGS-655	BnaA10G0244800ZS	scaffoldA10	23083882	23183881	100000	1433
BnaWGS-655	BnaA10G0244800ZS	scaffoldA10	23093882	23193881	100000	1528
BnaWGS-655	BnaA10G0244800ZS	scaffoldA10	23103882	23203881	100000	1613
BnaWGS-655	BnaA10G0244800ZS	scaffoldA10	23113882	23213881	100000	1821
BnaWGS-655	BnaA10G0244800ZS	scaffoldA10	23123882	23223881	100000	1914
BnaWGS-655	BnaA10G0244800ZS	scaffoldA10	23133882	23233881	100000	1906
BnaWGS-655	BnaA10G0244800ZS	scaffoldA10	23143882	23243881	100000	1740
BnaWGS-655	BnaA10G0244800ZS	scaffoldA10	23153882	23253881	100000	1621
BnaWGS-655	BnaA10G0244800ZS	scaffoldA10	23163882	23263881	100000	1493
BnaWGS-655	BnaA10G0244800ZS	scaffoldA10	23173882	23273881	100000	1412
BnaWGS-655	BnaA10G0244800ZS	scaffoldA10	23183882	23283881	100000	1338
BnaWGS-655	BnaA10G0244800ZS	scaffoldA10	23193882	23293881	100000	1150
BnaWGS-655	BnaA10G0244800ZS	scaffoldA10	23203882	23303881	100000	1074
BnaWGS-655	BnaA10G0244800ZS	scaffoldA10	23213882	23313881	100000	900
BnaWGS-655	BnaA10G0244800ZS	scaffoldA10	23223882	23323881	100000	928
BnaWGS-655	BnaA10G0244800ZS	scaffoldA10	23233882	23333881	100000	897
BnaWGS-655	BnaA10G0244800ZS	scaffoldA10	23243882	23343881	100000	1111
BnaWGS-655	BnaA10G0244800ZS	scaffoldA10	23253882	23353881	100000	1092
BnaWGS-655	BnaA10G0244800ZS	scaffoldA10	23263882	23363881	100000	1068
BnaWGS-655	BnaA10G0244800ZS	scaffoldA10	23273882	23373881	100000	1060
BnaWGS-655	BnaA10G0244800ZS	scaffoldA10	23283882	23383881	100000	1028
BnaWGS-655	BnaA10G0244800ZS	scaffoldA10	23293882	23393881	100000	1068
BnaWGS-655	BnaA10G0244800ZS	scaffoldA10	23303882	23403881	100000	1068
BnaWGS-655	BnaA10G0244800ZS	scaffoldA10	23313882	23413881	100000	1032
BnaWGS-655	BnaA10G0244800ZS	scaffoldA10	23323882	23423881	100000	957
BnaWGS-655	BnaA10G0244800ZS	scaffoldA10	23333882	23433881	100000	901
BnaWGS-655	BnaA10G0244800ZS	scaffoldA10	23343882	23443881	100000	726
BnaWGS-655	BnaA10G0244800ZS	scaffoldA10	23353882	23453881	100000	661
BnaWGS-655	BnaA10G0244800ZS	scaffoldA10	23363882	23463881	100000	689
BnaWGS-655	BnaA10G0244800ZS	scaffoldA10	23373882	23473881	100000	763
BnaWGS-655	BnaA10G0244800ZS	scaffoldA10	23383882	23483881	100000	866
BnaWGS-655	BnaA10G0244800ZS	scaffoldA10	23393882	23493881	100000	880
BnaWGS-655	BnaA10G0244800ZS	scaffoldA10	23403882	23503881	100000	901
BnaWGS-655	BnaA10G0244800ZS	scaffoldA10	23413882	23513881	100000	1082
BnaWGS-655	BnaA10G0244800ZS	scaffoldA10	23423882	23523881	100000	1122
BnaWGS-655	BnaA10G0244800ZS	scaffoldA10	23433882	23533881	100000	1098
BnaWGS-655	BnaA10G0244800ZS	scaffoldA10	23443882	23543881	100000	1317
BnaWGS-655	BnaA10G0244800ZS	scaffoldA10	23453882	23553881	100000	1372
BnaWGS-655	BnaA10G0244800ZS	scaffoldA10	23463882	23563881	100000	1385
BnaWGS-655	BnaA10G0244800ZS	scaffoldA10	23473882	23573881	100000	1360
BnaWGS-655	BnaA10G0244800ZS	scaffoldA10	23483882	23583881	100000	1289
BnaWGS-655	BnaA10G0244800ZS	scaffoldA10	23493882	23593881	100000	1326
BnaWGS-655	BnaA10G0244800ZS	scaffoldA10	23503882	23603881	100000	1368
BnaWGS-655	BnaA10G0244800ZS	scaffoldA10	23513882	23613881	100000	1161

For Review Only

BnaWGS-655	BnaA10G0244800ZS	scaffoldA10	23523882	23623881	100000	1220
BnaWGS-655	BnaA10G0244800ZS	scaffoldA10	23533882	23633881	100000	1326
BnaWGS-655	BnaA10G0244800ZS	scaffoldA10	23543882	23643881	100000	1146
BnaWGS-655	BnaA10G0244800ZS	scaffoldA10	23553882	23653881	100000	1143
BnaWGS-655	BnaA10G0244800ZS	scaffoldA10	23563882	23663881	100000	1193
BnaWGS-655	BnaA10G0244800ZS	scaffoldA10	23573882	23673881	100000	1432
BnaWGS-655	BnaA10G0244800ZS	scaffoldA10	23583882	23683881	100000	1621
BnaWGS-655	BnaA10G0244800ZS	scaffoldA10	23593882	23693881	100000	1556
BnaWGS-655	BnaA10G0244800ZS	scaffoldA10	23603882	23703881	100000	1568
BnaWGS-655	BnaA10G0244800ZS	scaffoldA10	23613882	23713881	100000	1681
BnaWGS-655	BnaA10G0244800ZS	scaffoldA10	23623882	23723881	100000	1593
BnaWGS-655	BnaA10G0244800ZS	scaffoldA10	23633882	23733881	100000	1557
BnaWGS-655	BnaA10G0244800ZS	scaffoldA10	23643882	23743881	100000	1566
BnaWGS-655	BnaA10G0244800ZS	scaffoldA10	23653882	23753881	100000	1693
BnaWGS-655	BnaA10G0244800ZS	scaffoldA10	23663882	23763881	100000	1712
BnaWGS-655	BnaA10G0244800ZS	scaffoldA10	23673882	23773881	100000	1548
BnaWGS-655	BnaA10G0244800ZS	scaffoldA10	23683882	23783881	100000	1467
BnaWGS-655	BnaA10G0244800ZS	scaffoldA10	23693882	23793881	100000	1524
BnaWGS-655	BnaA10G0244800ZS	scaffoldA10	23703882	23803881	100000	1550
BnaWGS-655	BnaA10G0244800ZS	scaffoldA10	23713882	23813881	100000	1510
BnaWGS-655	BnaA10G0244800ZS	scaffoldA10	23723882	23823881	100000	1516
BnaWGS-655	BnaA10G0244800ZS	scaffoldA10	23733882	23833881	100000	1453
BnaWGS-655	BnaA10G0244800ZS	scaffoldA10	23743882	23843881	100000	1423
BnaWGS-655	BnaA10G0244800ZS	scaffoldA10	23753882	23853881	100000	1328
BnaWGS-655	BnaA10G0244800ZS	scaffoldA10	23763882	23863881	100000	1360
BnaWGS-655	BnaA10G0244800ZS	scaffoldA10	23773882	23873881	100000	1230
BnaWGS-655	BnaA10G0244800ZS	scaffoldA10	23783882	23883881	100000	1158
BnaWGS-655	BnaA10G0244800ZS	scaffoldA10	23793882	23893881	100000	1126
BnaWGS-655	BnaA10G0244800ZS	scaffoldA10	23803882	23903881	100000	1005
BnaWGS-655	BnaA10G0244800ZS	scaffoldA10	23813882	23913881	100000	931
BnaWGS-655	BnaA10G0244800ZS	scaffoldA10	23823882	23923881	100000	861
BnaWGS-655	BnaA10G0244800ZS	scaffoldA10	23833882	23933881	100000	831
BnaWGS-655	BnaA10G0244800ZS	scaffoldA10	23843882	23943881	100000	823
BnaWGS-655	BnaA10G0244800ZS	scaffoldA10	23853882	23953881	100000	728
BnaWGS-655	BnaA10G0244800ZS	scaffoldA10	23863882	23963881	100000	597
BnaWGS-655	BnaA10G0244800ZS	scaffoldA10	23873882	23973881	100000	663
BnaWGS-655	BnaA10G0244800ZS	scaffoldA10	23883882	23983881	100000	787
BnaWGS-655	BnaA10G0244800ZS	scaffoldA10	23893882	23993881	100000	776
BnaWGS-655	BnaA10G0244800ZS	scaffoldA10	23903882	24003881	100000	794
BnaWGS-655	BnaA10G0244800ZS	scaffoldA10	23913882	24013881	100000	862
BnaWGS-655	BnaA10G0244800ZS	scaffoldA10	23923882	24023881	100000	993
BnaWGS-655	BnaA10G0244800ZS	scaffoldA10	23933882	24033881	100000	1010
BnaWGS-655	BnaA10G0244800ZS	scaffoldA10	23943882	24043881	100000	1121
BnaWGS-655	BnaA10G0244800ZS	scaffoldA10	23953882	24053881	100000	1138
BnaWGS-655	BnaA10G0244800ZS	scaffoldA10	23963882	24063881	100000	1234
BnaWGS-655	BnaA10G0244800ZS	scaffoldA10	23973882	24073881	100000	1325
BnaWGS-655	BnaA10G0244800ZS	scaffoldA10	23983882	24083881	100000	1322
BnaWGS-655	BnaA10G0244800ZS	scaffoldA10	23993882	24093881	100000	1427
BnaWGS-655	BnaA10G0244800ZS	scaffoldA10	24003882	24103881	100000	1572
BnaWGS-655	BnaA10G0244800ZS	scaffoldA10	24013882	24113881	100000	1616
BnaWGS-655	BnaA10G0244800ZS	scaffoldA10	24023882	24123881	100000	1711
BnaWGS-655	BnaA10G0244800ZS	scaffoldA10	24033882	24133881	100000	1737
BnaWGS-655	BnaA10G0244800ZS	scaffoldA10	24043882	24143881	100000	1725
BnaWGS-655	BnaA10G0244800ZS	scaffoldA10	24053882	24153881	100000	1796
BnaWGS-655	BnaA10G0244800ZS	scaffoldA10	24063882	24163881	100000	1814
BnaWGS-655	BnaA10G0244800ZS	scaffoldA10	24073882	24173881	100000	1836
BnaWGS-655	BnaA10G0244800ZS	scaffoldA10	24083882	24183881	100000	1993
BnaWGS-655	BnaA10G0244800ZS	scaffoldA10	24093882	24193881	100000	2122
BnaWGS-655	BnaA10G0244800ZS	scaffoldA10	24103882	24203881	100000	1993
BnaWGS-655	BnaA10G0244800ZS	scaffoldA10	24113882	24213881	100000	1887

BnaWGS-655	BnaA10G0244800ZS	scaffoldA10	24123882	24223881	100000	1926
BnaWGS-655	BnaA10G0244800ZS	scaffoldA10	24133882	24233881	100000	2032
BnaWGS-655	BnaA10G0244800ZS	scaffoldA10	24143882	24243881	100000	2093
BnaWGS-655	BnaA10G0244800ZS	scaffoldA10	24153882	24253881	100000	2152
BnaWGS-655	BnaA10G0244800ZS	scaffoldA10	24163882	24263881	100000	2159
BnaWGS-655	BnaA10G0244800ZS	scaffoldA10	24173882	24273881	100000	2190
BnaWGS-655	BnaA10G0244800ZS	scaffoldA10	24183882	24283881	100000	2032
BnaWGS-655	BnaA10G0244800ZS	scaffoldA10	24193882	24293881	100000	1863
BnaWGS-655	BnaA10G0244800ZS	scaffoldA10	24203882	24303881	100000	1936
BnaWGS-655	BnaA10G0244800ZS	scaffoldA10	24213882	24313881	100000	2055
BnaWGS-655	BnaA10G0244800ZS	scaffoldA10	24223882	24323881	100000	1956
BnaWGS-655	BnaA10G0244800ZS	scaffoldA10	24233882	24333881	100000	1961
BnaWGS-655	BnaA10G0244800ZS	scaffoldA10	24243882	24343881	100000	2003
BnaWGS-655	BnaA10G0244800ZS	scaffoldA10	24253882	24353881	100000	2142
BnaWGS-655	BnaA10G0244800ZS	scaffoldA10	24263882	24363881	100000	2073
BnaWGS-655	BnaA10G0244800ZS	scaffoldA10	24273882	24373881	100000	2050
BnaWGS-655	BnaA10G0244800ZS	scaffoldA10	24283882	24383881	100000	2054
BnaWGS-655	BnaA10G0244800ZS	scaffoldA10	24293882	24393881	100000	1969
BnaWGS-655	BnaA10G0244800ZS	scaffoldA10	24303882	24403881	100000	1811
BnaWGS-655	BnaA10G0244800ZS	scaffoldA10	24313882	24413881	100000	1845
BnaWGS-655	BnaA10G0244800ZS	scaffoldA10	24323882	24423881	100000	1931
BnaWGS-655	BnaA10G0244800ZS	scaffoldA10	24333882	24433881	100000	1929
BnaWGS-655	BnaA10G0244800ZS	scaffoldA10	24343882	24443881	100000	1795
BnaWGS-655	BnaA10G0244800ZS	scaffoldA10	24353882	24453881	100000	1546
BnaWGS-655	BnaA10G0244800ZS	scaffoldA10	24363882	24463881	100000	1749
BnaWGS-655	BnaA10G0244800ZS	scaffoldA10	24373882	24473881	100000	1713
BnaWGS-655	BnaA10G0244800ZS	scaffoldA10	24383882	24483881	100000	1610
BnaWGS-655	BnaA10G0244800ZS	scaffoldA10	24393882	24493881	100000	1706
BnaWGS-655	BnaA10G0244800ZS	scaffoldA10	24403882	24503881	100000	1799
BnaWGS-655	BnaA10G0244800ZS	scaffoldA10	24413882	24513881	100000	1675
BnaWGS-655	BnaA10G0244800ZS	scaffoldA10	24423882	24523881	100000	1463
BnaWGS-655	BnaA10G0244800ZS	scaffoldA10	24433882	24533881	100000	1338
BnaWGS-655	BnaA10G0244800ZS	scaffoldA10	24443882	24543881	100000	1326
BnaWGS-655	BnaA10G0244800ZS	scaffoldA10	24453882	24553881	100000	1472
BnaWGS-655	BnaA10G0244800ZS	scaffoldA10	24463882	24563881	100000	1372
BnaWGS-655	BnaA10G0244800ZS	scaffoldA10	24473882	24573881	100000	1397
BnaWGS-655	BnaA10G0244800ZS	scaffoldA10	24483882	24583881	100000	1425
BnaWGS-655	BnaA10G0244800ZS	scaffoldA10	24493882	24593881	100000	1317
BnaWGS-655	BnaA10G0244800ZS	scaffoldA10	24503882	24603881	100000	1311
BnaWGS-655	BnaA10G0244800ZS	scaffoldA10	24513882	24613881	100000	1361
BnaWGS-655	BnaA10G0244800ZS	scaffoldA10	24523882	24623881	100000	1473
BnaWGS-655	BnaA10G0244800ZS	scaffoldA10	24533882	24633881	100000	1514
BnaWGS-655	BnaA10G0244800ZS	scaffoldA10	24543882	24643881	100000	1529
BnaWGS-655	BnaA10G0244800ZS	scaffoldA10	24553882	24653881	100000	1509
BnaWGS-655	BnaA10G0244800ZS	scaffoldA10	24563882	24663881	100000	1412
BnaWGS-655	BnaA10G0244800ZS	scaffoldA10	24573882	24673881	100000	1274
BnaWGS-655	BnaA10G0244800ZS	scaffoldA10	24583882	24683881	100000	1340
BnaWGS-655	BnaA10G0244800ZS	scaffoldA10	24593882	24693881	100000	1438
BnaWGS-655	BnaA10G0244800ZS	scaffoldA10	24603882	24703881	100000	1443
BnaWGS-655	BnaA10G0244800ZS	scaffoldA10	24613882	24713881	100000	1383
BnaWGS-655	BnaA10G0244800ZS	scaffoldA10	24623882	24723881	100000	1273
BnaWGS-655	BnaA10G0244800ZS	scaffoldA10	24633882	24733881	100000	1302
BnaWGS-655	BnaA10G0244800ZS	scaffoldA10	24643882	24743881	100000	1330
BnaWGS-655	BnaA10G0244800ZS	scaffoldA10	24653882	24753881	100000	1341
BnaWGS-655	BnaA10G0244800ZS	scaffoldA10	24663882	24763881	100000	1253
BnaWGS-655	BnaA10G0244800ZS	scaffoldA10	24673882	24773881	100000	1181
BnaWGS-655	BnaA10G0244800ZS	scaffoldA10	24683882	24783881	100000	1170
BnaWGS-655	BnaA10G0244800ZS	scaffoldA10	24693882	24793881	100000	1246
BnaWGS-655	BnaA10G0244800ZS	scaffoldA10	24703882	24803881	100000	1228
BnaWGS-655	BnaA10G0244800ZS	scaffoldA10	24713882	24813881	100000	1307

For Review Only

BnaWGS-655	BnaA10G0244800ZS	scaffoldA10	24723882	24823881	100000	1466
BnaWGS-655	BnaA10G0244800ZS	scaffoldA10	24733882	24833881	100000	1448
BnaWGS-655	BnaA10G0244800ZS	scaffoldA10	24743882	24843881	100000	1519
BnaWGS-655	BnaA10G0244800ZS	scaffoldA10	24753882	24853881	100000	1428
BnaWGS-655	BnaA10G0244800ZS	scaffoldA10	24763882	24863881	100000	1451
BnaWGS-655	BnaA10G0244800ZS	scaffoldA10	24773882	24873881	100000	1607
BnaWGS-655	BnaA10G0244800ZS	scaffoldA10	24783882	24883881	100000	1467
BnaWGS-655	BnaA10G0244800ZS	scaffoldA10	24793882	24893881	100000	1285
BnaWGS-655	BnaA10G0244800ZS	scaffoldA10	24803882	24903881	100000	1392
BnaWGS-655	BnaA10G0244800ZS	scaffoldA10	24813882	24913881	100000	1477
BnaWGS-655	BnaA10G0244800ZS	scaffoldA10	24823882	24923881	100000	1636
BnaWGS-655	BnaA10G0244800ZS	scaffoldA10	24833882	24933881	100000	1790
BnaWGS-655	BnaA10G0244800ZS	scaffoldA10	24843882	24943881	100000	1654
BnaWGS-655	BnaA10G0244800ZS	scaffoldA10	24853882	24947904	94023	1580
BnaWGS-655	BnaA10G0244800ZS	scaffoldA10	22943882	23043881	100000	663
BnaWGS-655	BnaA10G0244800ZS	scaffoldA10	22953882	23053881	100000	624
BnaWGS-655	BnaA10G0244800ZS	scaffoldA10	22963882	23063881	100000	607
BnaWGS-655	BnaA10G0244800ZS	scaffoldA10	22973882	23073881	100000	600
BnaWGS-655	BnaA10G0244800ZS	scaffoldA10	22983882	23083881	100000	564
BnaWGS-655	BnaA10G0244800ZS	scaffoldA10	22993882	23093881	100000	591
BnaWGS-655	BnaA10G0244800ZS	scaffoldA10	23003882	23103881	100000	625
BnaWGS-655	BnaA10G0244800ZS	scaffoldA10	23013882	23113881	100000	625
BnaWGS-655	BnaA10G0244800ZS	scaffoldA10	23023882	23123881	100000	616
BnaWGS-655	BnaA10G0244800ZS	scaffoldA10	23033882	23133881	100000	718
BnaWGS-655	BnaA10G0244800ZS	scaffoldA10	23043882	23143881	100000	856
BnaWGS-655	BnaA10G0244800ZS	scaffoldA10	23053882	23153881	100000	1085
BnaWGS-655	BnaA10G0244800ZS	scaffoldA10	23063882	23163881	100000	1274
BnaWGS-655	BnaA10G0244800ZS	scaffoldA10	23073882	23173881	100000	1354
BnaWGS-655	BnaA10G0244800ZS	scaffoldA10	23083882	23183881	100000	1433
BnaWGS-655	BnaA10G0244800ZS	scaffoldA10	23093882	23193881	100000	1528
BnaWGS-655	BnaA10G0244800ZS	scaffoldA10	23103882	23203881	100000	1613
BnaWGS-655	BnaA10G0244800ZS	scaffoldA10	23113882	23213881	100000	1821
BnaWGS-655	BnaA10G0244800ZS	scaffoldA10	23123882	23223881	100000	1914
BnaWGS-655	BnaA10G0244800ZS	scaffoldA10	23133882	23233881	100000	1906
BnaWGS-655	BnaA10G0244800ZS	scaffoldA10	23143882	23243881	100000	1740
BnaWGS-655	BnaA10G0244800ZS	scaffoldA10	23153882	23253881	100000	1621
BnaWGS-655	BnaA10G0244800ZS	scaffoldA10	23163882	23263881	100000	1493
BnaWGS-655	BnaA10G0244800ZS	scaffoldA10	23173882	23273881	100000	1412
BnaWGS-655	BnaA10G0244800ZS	scaffoldA10	23183882	23283881	100000	1338
BnaWGS-655	BnaA10G0244800ZS	scaffoldA10	23193882	23293881	100000	1150
BnaWGS-655	BnaA10G0244800ZS	scaffoldA10	23203882	23303881	100000	1074
BnaWGS-655	BnaA10G0244800ZS	scaffoldA10	23213882	23313881	100000	900
BnaWGS-655	BnaA10G0244800ZS	scaffoldA10	23223882	23323881	100000	928
BnaWGS-655	BnaA10G0244800ZS	scaffoldA10	23233882	23333881	100000	897
BnaWGS-655	BnaA10G0244800ZS	scaffoldA10	23243882	23343881	100000	1111
BnaWGS-655	BnaA10G0244800ZS	scaffoldA10	23253882	23353881	100000	1092
BnaWGS-655	BnaA10G0244800ZS	scaffoldA10	23263882	23363881	100000	1068
BnaWGS-655	BnaA10G0244800ZS	scaffoldA10	23273882	23373881	100000	1060
BnaWGS-655	BnaA10G0244800ZS	scaffoldA10	23283882	23383881	100000	1028
BnaWGS-655	BnaA10G0244800ZS	scaffoldA10	23293882	23393881	100000	1068
BnaWGS-655	BnaA10G0244800ZS	scaffoldA10	23303882	23403881	100000	1068
BnaWGS-655	BnaA10G0244800ZS	scaffoldA10	23313882	23413881	100000	1032
BnaWGS-655	BnaA10G0244800ZS	scaffoldA10	23323882	23423881	100000	957
BnaWGS-655	BnaA10G0244800ZS	scaffoldA10	23333882	23433881	100000	901
BnaWGS-655	BnaA10G0244800ZS	scaffoldA10	23343882	23443881	100000	726
BnaWGS-655	BnaA10G0244800ZS	scaffoldA10	23353882	23453881	100000	661
BnaWGS-655	BnaA10G0244800ZS	scaffoldA10	23363882	23463881	100000	689
BnaWGS-655	BnaA10G0244800ZS	scaffoldA10	23373882	23473881	100000	763
BnaWGS-655	BnaA10G0244800ZS	scaffoldA10	23383882	23483881	100000	866
BnaWGS-655	BnaA10G0244800ZS	scaffoldA10	23393882	23493881	100000	880

For Review Only

BnaWGS-655	BnaA10G0244800ZS	scaffoldA10	23403882	23503881	100000	901
BnaWGS-655	BnaA10G0244800ZS	scaffoldA10	23413882	23513881	100000	1082
BnaWGS-655	BnaA10G0244800ZS	scaffoldA10	23423882	23523881	100000	1122
BnaWGS-655	BnaA10G0244800ZS	scaffoldA10	23433882	23533881	100000	1098
BnaWGS-655	BnaA10G0244800ZS	scaffoldA10	23443882	23543881	100000	1317
BnaWGS-655	BnaA10G0244800ZS	scaffoldA10	23453882	23553881	100000	1372
BnaWGS-655	BnaA10G0244800ZS	scaffoldA10	23463882	23563881	100000	1385
BnaWGS-655	BnaA10G0244800ZS	scaffoldA10	23473882	23573881	100000	1360
BnaWGS-655	BnaA10G0244800ZS	scaffoldA10	23483882	23583881	100000	1289
BnaWGS-655	BnaA10G0244800ZS	scaffoldA10	23493882	23593881	100000	1326
BnaWGS-655	BnaA10G0244800ZS	scaffoldA10	23503882	23603881	100000	1368
BnaWGS-655	BnaA10G0244800ZS	scaffoldA10	23513882	23613881	100000	1161
BnaWGS-655	BnaA10G0244800ZS	scaffoldA10	23523882	23623881	100000	1220
BnaWGS-655	BnaA10G0244800ZS	scaffoldA10	23533882	23633881	100000	1326
BnaWGS-655	BnaA10G0244800ZS	scaffoldA10	23543882	23643881	100000	1146
BnaWGS-655	BnaA10G0244800ZS	scaffoldA10	23553882	23653881	100000	1143
BnaWGS-655	BnaA10G0244800ZS	scaffoldA10	23563882	23663881	100000	1193
BnaWGS-655	BnaA10G0244800ZS	scaffoldA10	23573882	23673881	100000	1432
BnaWGS-655	BnaA10G0244800ZS	scaffoldA10	23583882	23683881	100000	1621
BnaWGS-655	BnaA10G0244800ZS	scaffoldA10	23593882	23693881	100000	1556
BnaWGS-655	BnaA10G0244800ZS	scaffoldA10	23603882	23703881	100000	1568
BnaWGS-655	BnaA10G0244800ZS	scaffoldA10	23613882	23713881	100000	1681
BnaWGS-655	BnaA10G0244800ZS	scaffoldA10	23623882	23723881	100000	1593
BnaWGS-655	BnaA10G0244800ZS	scaffoldA10	23633882	23733881	100000	1557
BnaWGS-655	BnaA10G0244800ZS	scaffoldA10	23643882	23743881	100000	1566
BnaWGS-655	BnaA10G0244800ZS	scaffoldA10	23653882	23753881	100000	1693
BnaWGS-655	BnaA10G0244800ZS	scaffoldA10	23663882	23763881	100000	1712
BnaWGS-655	BnaA10G0244800ZS	scaffoldA10	23673882	23773881	100000	1548
BnaWGS-655	BnaA10G0244800ZS	scaffoldA10	23683882	23783881	100000	1467
BnaWGS-655	BnaA10G0244800ZS	scaffoldA10	23693882	23793881	100000	1524
BnaWGS-655	BnaA10G0244800ZS	scaffoldA10	23703882	23803881	100000	1550
BnaWGS-655	BnaA10G0244800ZS	scaffoldA10	23713882	23813881	100000	1510
BnaWGS-655	BnaA10G0244800ZS	scaffoldA10	23723882	23823881	100000	1516
BnaWGS-655	BnaA10G0244800ZS	scaffoldA10	23733882	23833881	100000	1453
BnaWGS-655	BnaA10G0244800ZS	scaffoldA10	23743882	23843881	100000	1423
BnaWGS-655	BnaA10G0244800ZS	scaffoldA10	23753882	23853881	100000	1328
BnaWGS-655	BnaA10G0244800ZS	scaffoldA10	23763882	23863881	100000	1360
BnaWGS-655	BnaA10G0244800ZS	scaffoldA10	23773882	23873881	100000	1230
BnaWGS-655	BnaA10G0244800ZS	scaffoldA10	23783882	23883881	100000	1158
BnaWGS-655	BnaA10G0244800ZS	scaffoldA10	23793882	23893881	100000	1126
BnaWGS-655	BnaA10G0244800ZS	scaffoldA10	23803882	23903881	100000	1005
BnaWGS-655	BnaA10G0244800ZS	scaffoldA10	23813882	23913881	100000	931
BnaWGS-655	BnaA10G0244800ZS	scaffoldA10	23823882	23923881	100000	861
BnaWGS-655	BnaA10G0244800ZS	scaffoldA10	23833882	23933881	100000	831
BnaWGS-655	BnaA10G0244800ZS	scaffoldA10	23843882	23943881	100000	823
BnaWGS-655	BnaA10G0244800ZS	scaffoldA10	23853882	23953881	100000	728
BnaWGS-655	BnaA10G0244800ZS	scaffoldA10	23863882	23963881	100000	597
BnaWGS-655	BnaA10G0244800ZS	scaffoldA10	23873882	23973881	100000	663
BnaWGS-655	BnaA10G0244800ZS	scaffoldA10	23883882	23983881	100000	787
BnaWGS-655	BnaA10G0244800ZS	scaffoldA10	23893882	23993881	100000	776
BnaWGS-655	BnaA10G0244800ZS	scaffoldA10	23903882	24003881	100000	794
BnaWGS-655	BnaA10G0244800ZS	scaffoldA10	23913882	24013881	100000	862
BnaWGS-655	BnaA10G0244800ZS	scaffoldA10	23923882	24023881	100000	993
BnaWGS-655	BnaA10G0244800ZS	scaffoldA10	23933882	24033881	100000	1010
BnaWGS-655	BnaA10G0244800ZS	scaffoldA10	23943882	24043881	100000	1121
BnaWGS-655	BnaA10G0244800ZS	scaffoldA10	23953882	24053881	100000	1138
BnaWGS-655	BnaA10G0244800ZS	scaffoldA10	23963882	24063881	100000	1234
BnaWGS-655	BnaA10G0244800ZS	scaffoldA10	23973882	24073881	100000	1325
BnaWGS-655	BnaA10G0244800ZS	scaffoldA10	23983882	24083881	100000	1322
BnaWGS-655	BnaA10G0244800ZS	scaffoldA10	23993882	24093881	100000	1427

For Review Only

BnaWGS-655	BnaA10G0244800ZS	scaffoldA10	24003882	24103881	100000	1572
BnaWGS-655	BnaA10G0244800ZS	scaffoldA10	24013882	24113881	100000	1616
BnaWGS-655	BnaA10G0244800ZS	scaffoldA10	24023882	24123881	100000	1711
BnaWGS-655	BnaA10G0244800ZS	scaffoldA10	24033882	24133881	100000	1737
BnaWGS-655	BnaA10G0244800ZS	scaffoldA10	24043882	24143881	100000	1725
BnaWGS-655	BnaA10G0244800ZS	scaffoldA10	24053882	24153881	100000	1796
BnaWGS-655	BnaA10G0244800ZS	scaffoldA10	24063882	24163881	100000	1814
BnaWGS-655	BnaA10G0244800ZS	scaffoldA10	24073882	24173881	100000	1836
BnaWGS-655	BnaA10G0244800ZS	scaffoldA10	24083882	24183881	100000	1993
BnaWGS-655	BnaA10G0244800ZS	scaffoldA10	24093882	24193881	100000	2122
BnaWGS-655	BnaA10G0244800ZS	scaffoldA10	24103882	24203881	100000	1993
BnaWGS-655	BnaA10G0244800ZS	scaffoldA10	24113882	24213881	100000	1887
BnaWGS-655	BnaA10G0244800ZS	scaffoldA10	24123882	24223881	100000	1926
BnaWGS-655	BnaA10G0244800ZS	scaffoldA10	24133882	24233881	100000	2032
BnaWGS-655	BnaA10G0244800ZS	scaffoldA10	24143882	24243881	100000	2093
BnaWGS-655	BnaA10G0244800ZS	scaffoldA10	24153882	24253881	100000	2152
BnaWGS-655	BnaA10G0244800ZS	scaffoldA10	24163882	24263881	100000	2159
BnaWGS-655	BnaA10G0244800ZS	scaffoldA10	24173882	24273881	100000	2190
BnaWGS-655	BnaA10G0244800ZS	scaffoldA10	24183882	24283881	100000	2032
BnaWGS-655	BnaA10G0244800ZS	scaffoldA10	24193882	24293881	100000	1863
BnaWGS-655	BnaA10G0244800ZS	scaffoldA10	24203882	24303881	100000	1936
BnaWGS-655	BnaA10G0244800ZS	scaffoldA10	24213882	24313881	100000	2055
BnaWGS-655	BnaA10G0244800ZS	scaffoldA10	24223882	24323881	100000	1956
BnaWGS-655	BnaA10G0244800ZS	scaffoldA10	24233882	24333881	100000	1961
BnaWGS-655	BnaA10G0244800ZS	scaffoldA10	24243882	24343881	100000	2003
BnaWGS-655	BnaA10G0244800ZS	scaffoldA10	24253882	24353881	100000	2142
BnaWGS-655	BnaA10G0244800ZS	scaffoldA10	24263882	24363881	100000	2073
BnaWGS-655	BnaA10G0244800ZS	scaffoldA10	24273882	24373881	100000	2050
BnaWGS-655	BnaA10G0244800ZS	scaffoldA10	24283882	24383881	100000	2054
BnaWGS-655	BnaA10G0244800ZS	scaffoldA10	24293882	24393881	100000	1969
BnaWGS-655	BnaA10G0244800ZS	scaffoldA10	24303882	24403881	100000	1811
BnaWGS-655	BnaA10G0244800ZS	scaffoldA10	24313882	24413881	100000	1845
BnaWGS-655	BnaA10G0244800ZS	scaffoldA10	24323882	24423881	100000	1931
BnaWGS-655	BnaA10G0244800ZS	scaffoldA10	24333882	24433881	100000	1929
BnaWGS-655	BnaA10G0244800ZS	scaffoldA10	24343882	24443881	100000	1795
BnaWGS-655	BnaA10G0244800ZS	scaffoldA10	24353882	24453881	100000	1546
BnaWGS-655	BnaA10G0244800ZS	scaffoldA10	24363882	24463881	100000	1749
BnaWGS-655	BnaA10G0244800ZS	scaffoldA10	24373882	24473881	100000	1713
BnaWGS-655	BnaA10G0244800ZS	scaffoldA10	24383882	24483881	100000	1610
BnaWGS-655	BnaA10G0244800ZS	scaffoldA10	24393882	24493881	100000	1706
BnaWGS-655	BnaA10G0244800ZS	scaffoldA10	24403882	24503881	100000	1799
BnaWGS-655	BnaA10G0244800ZS	scaffoldA10	24413882	24513881	100000	1675
BnaWGS-655	BnaA10G0244800ZS	scaffoldA10	24423882	24523881	100000	1463
BnaWGS-655	BnaA10G0244800ZS	scaffoldA10	24433882	24533881	100000	1338
BnaWGS-655	BnaA10G0244800ZS	scaffoldA10	24443882	24543881	100000	1326
BnaWGS-655	BnaA10G0244800ZS	scaffoldA10	24453882	24553881	100000	1472
BnaWGS-655	BnaA10G0244800ZS	scaffoldA10	24463882	24563881	100000	1372
BnaWGS-655	BnaA10G0244800ZS	scaffoldA10	24473882	24573881	100000	1397
BnaWGS-655	BnaA10G0244800ZS	scaffoldA10	24483882	24583881	100000	1425
BnaWGS-655	BnaA10G0244800ZS	scaffoldA10	24493882	24593881	100000	1317
BnaWGS-655	BnaA10G0244800ZS	scaffoldA10	24503882	24603881	100000	1311
BnaWGS-655	BnaA10G0244800ZS	scaffoldA10	24513882	24613881	100000	1361
BnaWGS-655	BnaA10G0244800ZS	scaffoldA10	24523882	24623881	100000	1473
BnaWGS-655	BnaA10G0244800ZS	scaffoldA10	24533882	24633881	100000	1514
BnaWGS-655	BnaA10G0244800ZS	scaffoldA10	24543882	24643881	100000	1529
BnaWGS-655	BnaA10G0244800ZS	scaffoldA10	24553882	24653881	100000	1509
BnaWGS-655	BnaA10G0244800ZS	scaffoldA10	24563882	24663881	100000	1412
BnaWGS-655	BnaA10G0244800ZS	scaffoldA10	24573882	24673881	100000	1274
BnaWGS-655	BnaA10G0244800ZS	scaffoldA10	24583882	24683881	100000	1340
BnaWGS-655	BnaA10G0244800ZS	scaffoldA10	24593882	24693881	100000	1438

For Review Only

BnaWGS-655	BnaA10G0244800ZS	scaffoldA10	24603882	24703881	100000	1443
BnaWGS-655	BnaA10G0244800ZS	scaffoldA10	24613882	24713881	100000	1383
BnaWGS-655	BnaA10G0244800ZS	scaffoldA10	24623882	24723881	100000	1273
BnaWGS-655	BnaA10G0244800ZS	scaffoldA10	24633882	24733881	100000	1302
BnaWGS-655	BnaA10G0244800ZS	scaffoldA10	24643882	24743881	100000	1330
BnaWGS-655	BnaA10G0244800ZS	scaffoldA10	24653882	24753881	100000	1341
BnaWGS-655	BnaA10G0244800ZS	scaffoldA10	24663882	24763881	100000	1253
BnaWGS-655	BnaA10G0244800ZS	scaffoldA10	24673882	24773881	100000	1181
BnaWGS-655	BnaA10G0244800ZS	scaffoldA10	24683882	24783881	100000	1170
BnaWGS-655	BnaA10G0244800ZS	scaffoldA10	24693882	24793881	100000	1246
BnaWGS-655	BnaA10G0244800ZS	scaffoldA10	24703882	24803881	100000	1228
BnaWGS-655	BnaA10G0244800ZS	scaffoldA10	24713882	24813881	100000	1307
BnaWGS-655	BnaA10G0244800ZS	scaffoldA10	24723882	24823881	100000	1466
BnaWGS-655	BnaA10G0244800ZS	scaffoldA10	24733882	24833881	100000	1448
BnaWGS-655	BnaA10G0244800ZS	scaffoldA10	24743882	24843881	100000	1519
BnaWGS-655	BnaA10G0244800ZS	scaffoldA10	24753882	24853881	100000	1428
BnaWGS-655	BnaA10G0244800ZS	scaffoldA10	24763882	24863881	100000	1451
BnaWGS-655	BnaA10G0244800ZS	scaffoldA10	24773882	24873881	100000	1607
BnaWGS-655	BnaA10G0244800ZS	scaffoldA10	24783882	24883881	100000	1467
BnaWGS-655	BnaA10G0244800ZS	scaffoldA10	24793882	24893881	100000	1285
BnaWGS-655	BnaA10G0244800ZS	scaffoldA10	24803882	24903881	100000	1392
BnaWGS-655	BnaA10G0244800ZS	scaffoldA10	24813882	24913881	100000	1477
BnaWGS-655	BnaA10G0244800ZS	scaffoldA10	24823882	24923881	100000	1636
BnaWGS-655	BnaA10G0244800ZS	scaffoldA10	24833882	24933881	100000	1790
BnaWGS-655	BnaA10G0244800ZS	scaffoldA10	24843882	24943881	100000	1654
BnaWGS-655	BnaA10G0244800ZS	scaffoldA10	24853882	24947904	94023	1580

For Review Only

ıs surrounding gene

pi	groups
0.005055	Winter
0.005095	Winter
0.005085	Winter
0.004352	Winter
0.004458	Winter
0.0043	Winter
0.003942	Winter
0.00385	Winter
0.003803	Winter
0.003633	Winter
0.003631	Winter
0.003299	Winter
0.002994	Winter
0.003097	Winter
0.002756	Winter
0.002437	Winter
0.002744	Winter
0.002743	Winter
0.002746	Winter
0.002569	Winter
0.002411	Winter
0.002404	Winter
0.002684	Winter
0.002703	Winter
0.00251	Winter
0.002255	Winter
0.001654	Winter
0.001342	Winter
0.00112	Winter
0.001074	Winter
0.001055	Winter
0.000903	Winter
0.000476	Winter
0.000243	Winter
0.000132	Winter
8.98E-05	Winter
0.000129	Winter
0.000162	Winter
0.000169	Winter
0.000178	Winter
0.000665	Winter
0.001195	Winter
0.001248	Winter
0.001604	Winter
0.001833	Winter
0.002288	Winter
0.002362	Winter
0.002606	Winter
0.002683	Winter
0.002952	Winter
0.002779	Winter
0.002655	Winter
0.003356	Winter
0.003696	Winter
0.003641	Winter
0.003341	Winter
0.003418	Winter
0.003601	Winter

For Review Only

0.003823	Winter
0.003886	Winter
0.003806	Winter
0.003472	Winter
0.003249	Winter
0.003842	Winter
0.003826	Winter
0.003727	Winter
0.003718	Winter
0.003522	Winter
0.003398	Winter
0.003126	Winter
0.003364	Winter
0.00408	Winter
0.003651	Winter
0.002796	Winter
0.003331	Winter
0.003692	Winter
0.003972	Winter
0.003793	Winter
0.004092	Winter
0.004592	Winter
0.004238	Winter
0.003583	Winter
0.003788	Winter
0.003632	Winter
0.00322	Winter
0.003268	Winter
0.003287	Winter
0.003722	Winter
0.003894	Winter
0.003651	Winter
0.00366	Winter
0.003816	Winter
0.003929	Winter
0.00427	Winter
0.004554	Winter
0.004354	Winter
0.004018	Winter
0.003687	Winter
0.003128	Winter
0.002962	Winter
0.003394	Winter
0.003478	Winter
0.00322	Winter
0.002729	Winter
0.002479	Winter
0.002732	Winter
0.002679	Winter
0.002565	Winter
0.002531	Winter
0.002539	Winter
0.002117	Winter
0.001783	Winter
0.001734	Winter
0.001819	Winter
0.001632	Winter
0.001345	Winter
0.001433	Winter
0.001696	Winter

For Review Only

0.001856	Winter
0.001791	Winter
0.001806	Winter
0.002067	Winter
0.002754	Winter
0.00287	Winter
0.003251	Winter
0.003195	Winter
0.003186	Winter
0.002938	Winter
0.003007	Winter
0.003197	Winter
0.003359	Winter
0.003062	Winter
0.002398	Winter
0.002929	Winter
0.002846	Winter
0.002944	Winter
0.00297	Winter
0.003384	Winter
0.003155	Winter
0.003334	Winter
0.003752	Winter
0.003796	Winter
0.003831	Winter
0.003302	Winter
0.003292	Winter
0.003899	Winter
0.004003	Winter
0.003666	Winter
0.003855	Winter
0.003654	Winter
0.003303	Winter
0.004018	Winter
0.004189	Winter
0.00504	Winter
0.004759	Winter
0.003943	Winter
0.003668	Winter
0.004068	Winter
0.004356	Winter
0.004537	Winter
0.004506	Winter
0.004239	Winter
0.004338	Winter
0.003431	Winter
0.003383	Winter
0.003619	Winter
0.004038	Winter
0.004131	Winter
0.004305	Winter
0.004022	Winter
0.003819	Winter
0.00381	Winter
0.003837	Winter
0.003856	Winter
0.004334	Winter
0.004167	Winter
0.003879	Winter
0.003643	Winter

For Review Only

0.003168	Winter
0.003599	Winter
0.003887	Winter
0.004075	Winter
0.003772	Winter
0.003808	Winter
0.003822	Winter
0.004335	Winter
0.004548	Winter
0.004266	Winter
0.004251	Winter
0.00415	Winter
0.003934	Winter
0.003583	Winter
0.005084	Spring
0.005174	Spring
0.005151	Spring
0.004495	Spring
0.004962	Spring
0.00496	Spring
0.004476	Spring
0.004273	Spring
0.004495	Spring
0.004291	Spring
0.004357	Spring
0.004047	Spring
0.003878	Spring
0.003992	Spring
0.003545	Spring
0.0035	Spring
0.00442	Spring
0.00472	Spring
0.00465	Spring
0.004451	Spring
0.004173	Spring
0.004187	Spring
0.004532	Spring
0.004584	Spring
0.004313	Spring
0.00393	Spring
0.00281	Spring
0.002244	Spring
0.001943	Spring
0.001852	Spring
0.001816	Spring
0.001522	Spring
0.0008	Spring
0.000456	Spring
0.000282	Spring
0.000186	Spring
0.000384	Spring
0.000698	Spring
0.000754	Spring
0.000834	Spring
0.001579	Spring
0.002378	Spring
0.002511	Spring
0.003003	Spring
0.003244	Spring
0.003625	Spring

For Review Only

0.003647	Spring
0.003634	Spring
0.003718	Spring
0.003988	Spring
0.003642	Spring
0.003367	Spring
0.004088	Spring
0.004358	Spring
0.004265	Spring
0.0039	Spring
0.003869	Spring
0.004007	Spring
0.004237	Spring
0.00438	Spring
0.004319	Spring
0.003914	Spring
0.003702	Spring
0.00473	Spring
0.004814	Spring
0.004833	Spring
0.004903	Spring
0.004962	Spring
0.004815	Spring
0.004584	Spring
0.004817	Spring
0.005787	Spring
0.005348	Spring
0.004184	Spring
0.004753	Spring
0.005105	Spring
0.005289	Spring
0.004878	Spring
0.005247	Spring
0.00527	Spring
0.004891	Spring
0.004105	Spring
0.004443	Spring
0.004323	Spring
0.004	Spring
0.004206	Spring
0.004571	Spring
0.005477	Spring
0.005701	Spring
0.005859	Spring
0.005911	Spring
0.006024	Spring
0.006068	Spring
0.006424	Spring
0.00674	Spring
0.006452	Spring
0.00587	Spring
0.005172	Spring
0.004498	Spring
0.004299	Spring
0.00483	Spring
0.004918	Spring
0.004533	Spring
0.003852	Spring
0.003617	Spring
0.003617	Spring

For Review Only

0.003734	Spring
0.003556	Spring
0.003504	Spring
0.003486	Spring
0.002936	Spring
0.002482	Spring
0.002515	Spring
0.002588	Spring
0.002188	Spring
0.002099	Spring
0.002046	Spring
0.002315	Spring
0.002573	Spring
0.002494	Spring
0.002511	Spring
0.002771	Spring
0.003201	Spring
0.003214	Spring
0.003605	Spring
0.003453	Spring
0.00333	Spring
0.003032	Spring
0.002981	Spring
0.00312	Spring
0.003203	Spring
0.002895	Spring
0.002372	Spring
0.002945	Spring
0.002648	Spring
0.002722	Spring
0.002796	Spring
0.003243	Spring
0.002983	Spring
0.002946	Spring
0.003058	Spring
0.003084	Spring
0.002995	Spring
0.002317	Spring
0.00229	Spring
0.002511	Spring
0.002442	Spring
0.002006	Spring
0.002076	Spring
0.001913	Spring
0.001577	Spring
0.001817	Spring
0.001889	Spring
0.001959	Spring
0.001769	Spring
0.001266	Spring
0.001106	Spring
0.00123	Spring
0.001408	Spring
0.001559	Spring
0.001798	Spring
0.002114	Spring
0.002452	Spring
0.002506	Spring
0.002577	Spring
0.00299	Spring

For Review Only

0.00348	Spring
0.003873	Spring
0.004279	Spring
0.004282	Spring
0.004138	Spring
0.004149	Spring
0.00431	Spring
0.004146	Spring
0.004325	Spring
0.004122	Spring
0.003855	Spring
0.003848	Spring
0.003388	Spring
0.0038	Spring
0.004016	Spring
0.004151	Spring
0.003703	Spring
0.003838	Spring
0.004198	Spring
0.004245	Spring
0.00421	Spring
0.003771	Spring
0.003772	Spring
0.003411	Spring
0.003181	Spring
0.002755	Spring
0.006186	Semi-winter
0.006391	Semi-winter
0.006215	Semi-winter
0.00542	Semi-winter
0.005955	Semi-winter
0.005804	Semi-winter
0.005307	Semi-winter
0.005182	Semi-winter
0.005453	Semi-winter
0.005043	Semi-winter
0.005084	Semi-winter
0.004709	Semi-winter
0.004879	Semi-winter
0.005275	Semi-winter
0.004847	Semi-winter
0.004772	Semi-winter
0.005706	Semi-winter
0.005956	Semi-winter
0.005731	Semi-winter
0.005484	Semi-winter
0.005147	Semi-winter
0.004987	Semi-winter
0.005191	Semi-winter
0.004943	Semi-winter
0.004514	Semi-winter
0.004327	Semi-winter
0.003143	Semi-winter
0.002546	Semi-winter
0.002259	Semi-winter
0.002144	Semi-winter
0.002078	Semi-winter
0.001767	Semi-winter
0.00103	Semi-winter
0.00068	Semi-winter

For Review Only

0.000494	Semi-winter
0.000163	Semi-winter
0.000333	Semi-winter
0.000622	Semi-winter
0.000671	Semi-winter
0.000863	Semi-winter
0.00149	Semi-winter
0.002195	Semi-winter
0.002269	Semi-winter
0.002791	Semi-winter
0.003146	Semi-winter
0.003607	Semi-winter
0.003662	Semi-winter
0.003745	Semi-winter
0.003801	Semi-winter
0.003915	Semi-winter
0.003771	Semi-winter
0.003735	Semi-winter
0.004421	Semi-winter
0.004627	Semi-winter
0.004445	Semi-winter
0.004036	Semi-winter
0.003988	Semi-winter
0.003997	Semi-winter
0.004262	Semi-winter
0.004343	Semi-winter
0.004333	Semi-winter
0.003867	Semi-winter
0.003806	Semi-winter
0.004755	Semi-winter
0.00485	Semi-winter
0.00482	Semi-winter
0.004885	Semi-winter
0.004966	Semi-winter
0.004792	Semi-winter
0.004584	Semi-winter
0.004807	Semi-winter
0.005703	Semi-winter
0.005549	Semi-winter
0.004836	Semi-winter
0.00534	Semi-winter
0.006087	Semi-winter
0.006691	Semi-winter
0.00645	Semi-winter
0.007085	Semi-winter
0.007411	Semi-winter
0.007039	Semi-winter
0.006477	Semi-winter
0.006432	Semi-winter
0.005973	Semi-winter
0.005625	Semi-winter
0.005488	Semi-winter
0.005386	Semi-winter
0.006117	Semi-winter
0.00592	Semi-winter
0.005931	Semi-winter
0.00585	Semi-winter
0.005761	Semi-winter
0.005777	Semi-winter
0.005814	Semi-winter

For Review Only

0.005814	Semi-winter
0.005311	Semi-winter
0.004794	Semi-winter
0.00409	Semi-winter
0.003572	Semi-winter
0.003237	Semi-winter
0.003876	Semi-winter
0.004149	Semi-winter
0.003903	Semi-winter
0.003654	Semi-winter
0.003855	Semi-winter
0.004426	Semi-winter
0.004469	Semi-winter
0.004451	Semi-winter
0.00454	Semi-winter
0.004692	Semi-winter
0.004557	Semi-winter
0.003996	Semi-winter
0.004176	Semi-winter
0.004607	Semi-winter
0.004358	Semi-winter
0.00416	Semi-winter
0.004184	Semi-winter
0.004487	Semi-winter
0.005085	Semi-winter
0.005116	Semi-winter
0.004865	Semi-winter
0.005296	Semi-winter
0.00587	Semi-winter
0.005457	Semi-winter
0.005623	Semi-winter
0.005391	Semi-winter
0.005348	Semi-winter
0.004955	Semi-winter
0.00455	Semi-winter
0.004528	Semi-winter
0.004587	Semi-winter
0.003999	Semi-winter
0.003163	Semi-winter
0.003996	Semi-winter
0.003905	Semi-winter
0.003888	Semi-winter
0.003893	Semi-winter
0.004388	Semi-winter
0.004046	Semi-winter
0.003942	Semi-winter
0.003879	Semi-winter
0.003922	Semi-winter
0.003799	Semi-winter
0.002859	Semi-winter
0.002975	Semi-winter
0.003938	Semi-winter
0.004046	Semi-winter
0.003618	Semi-winter
0.003767	Semi-winter
0.003668	Semi-winter
0.003329	Semi-winter
0.003691	Semi-winter
0.003804	Semi-winter
0.003905	Semi-winter

For Review Only

0.003392	Semi-winter
0.002087	Semi-winter
0.001729	Semi-winter
0.001848	Semi-winter
0.002104	Semi-winter
0.002551	Semi-winter
0.002782	Semi-winter
0.002931	Semi-winter
0.003162	Semi-winter
0.003257	Semi-winter
0.003505	Semi-winter
0.004104	Semi-winter
0.00464	Semi-winter
0.005198	Semi-winter
0.005757	Semi-winter
0.006003	Semi-winter
0.006058	Semi-winter
0.006236	Semi-winter
0.006546	Semi-winter
0.006357	Semi-winter
0.006535	Semi-winter
0.006117	Semi-winter
0.005812	Semi-winter
0.005693	Semi-winter
0.005008	Semi-winter
0.005009	Semi-winter
0.005109	Semi-winter
0.005285	Semi-winter
0.004766	Semi-winter
0.00492	Semi-winter
0.005126	Semi-winter
0.005338	Semi-winter
0.005386	Semi-winter
0.004861	Semi-winter
0.004733	Semi-winter
0.004426	Semi-winter
0.004211	Semi-winter
0.003703	Semi-winter
0.007142	Semi-winter
0.007519	Semi-winter
0.007505	Semi-winter
0.006835	Semi-winter
0.007381	Semi-winter
0.007205	Semi-winter
0.00669	Semi-winter
0.006406	Semi-winter
0.006644	Semi-winter
0.006223	Semi-winter
0.006156	Semi-winter
0.005402	Semi-winter
0.005431	Semi-winter
0.005625	Semi-winter
0.005065	Semi-winter
0.004846	Semi-winter
0.005466	Semi-winter
0.005625	Semi-winter
0.005398	Semi-winter
0.005203	Semi-winter
0.004879	Semi-winter
0.004785	Semi-winter

For Review Only

0.004841	Semi-winter
0.004709	Semi-winter
0.004197	Semi-winter
0.00404	Semi-winter
0.003025	Semi-winter
0.002495	Semi-winter
0.002238	Semi-winter
0.002114	Semi-winter
0.002043	Semi-winter
0.001685	Semi-winter
0.001051	Semi-winter
0.000677	Semi-winter
0.000515	Semi-winter
0.000197	Semi-winter
0.000383	Semi-winter
0.000643	Semi-winter
0.000742	Semi-winter
0.0009	Semi-winter
0.001543	Semi-winter
0.002275	Semi-winter
0.002362	Semi-winter
0.002857	Semi-winter
0.003191	Semi-winter
0.003808	Semi-winter
0.004023	Semi-winter
0.004227	Semi-winter
0.004273	Semi-winter
0.004511	Semi-winter
0.004555	Semi-winter
0.004581	Semi-winter
0.005181	Semi-winter
0.005342	Semi-winter
0.005292	Semi-winter
0.004696	Semi-winter
0.004418	Semi-winter
0.004278	Semi-winter
0.004652	Semi-winter
0.00467	Semi-winter
0.004526	Semi-winter
0.003989	Semi-winter
0.004067	Semi-winter
0.004592	Semi-winter
0.004594	Semi-winter
0.004574	Semi-winter
0.004662	Semi-winter
0.004861	Semi-winter
0.004572	Semi-winter
0.004376	Semi-winter
0.004491	Semi-winter
0.005394	Semi-winter
0.005216	Semi-winter
0.005095	Semi-winter
0.005955	Semi-winter
0.006889	Semi-winter
0.0076	Semi-winter
0.007313	Semi-winter
0.008185	Semi-winter
0.008562	Semi-winter
0.008327	Semi-winter
0.007755	Semi-winter

For Review Only

0.007816	Semi-winter
0.007274	Semi-winter
0.006793	Semi-winter
0.006733	Semi-winter
0.006809	Semi-winter
0.007991	Semi-winter
0.007825	Semi-winter
0.007848	Semi-winter
0.007647	Semi-winter
0.007534	Semi-winter
0.007737	Semi-winter
0.00799	Semi-winter
0.007814	Semi-winter
0.007242	Semi-winter
0.006433	Semi-winter
0.005273	Semi-winter
0.004499	Semi-winter
0.004091	Semi-winter
0.004927	Semi-winter
0.005251	Semi-winter
0.004745	Semi-winter
0.004334	Semi-winter
0.004515	Semi-winter
0.005215	Semi-winter
0.005377	Semi-winter
0.005399	Semi-winter
0.005736	Semi-winter
0.006008	Semi-winter
0.005925	Semi-winter
0.005483	Semi-winter
0.005765	Semi-winter
0.006134	Semi-winter
0.005877	Semi-winter
0.005476	Semi-winter
0.005512	Semi-winter
0.006009	Semi-winter
0.006528	Semi-winter
0.006515	Semi-winter
0.006106	Semi-winter
0.006506	Semi-winter
0.00715	Semi-winter
0.006886	Semi-winter
0.007099	Semi-winter
0.006908	Semi-winter
0.006781	Semi-winter
0.006213	Semi-winter
0.00595	Semi-winter
0.006594	Semi-winter
0.006738	Semi-winter
0.006099	Semi-winter
0.005062	Semi-winter
0.005672	Semi-winter
0.005535	Semi-winter
0.005451	Semi-winter
0.005647	Semi-winter
0.006115	Semi-winter
0.005474	Semi-winter
0.004672	Semi-winter
0.004587	Semi-winter
0.004557	Semi-winter

For Review Only

0.004381	Semi-winter
0.003476	Semi-winter
0.003466	Semi-winter
0.004131	Semi-winter
0.003952	Semi-winter
0.00348	Semi-winter
0.003609	Semi-winter
0.003647	Semi-winter
0.003467	Semi-winter
0.00408	Semi-winter
0.004307	Semi-winter
0.004825	Semi-winter
0.004469	Semi-winter
0.003424	Semi-winter
0.003203	Semi-winter
0.003861	Semi-winter
0.004424	Semi-winter
0.004659	Semi-winter
0.005006	Semi-winter
0.005013	Semi-winter
0.005214	Semi-winter
0.005009	Semi-winter
0.005241	Semi-winter
0.005802	Semi-winter
0.006579	Semi-winter
0.006812	Semi-winter
0.007179	Semi-winter
0.007455	Semi-winter
0.007329	Semi-winter
0.00754	Semi-winter
0.007923	Semi-winter
0.007963	Semi-winter
0.008211	Semi-winter
0.007896	Semi-winter
0.007365	Semi-winter
0.007291	Semi-winter
0.006725	Semi-winter
0.00688	Semi-winter
0.006764	Semi-winter
0.006822	Semi-winter
0.006133	Semi-winter
0.006027	Semi-winter
0.006238	Semi-winter
0.006886	Semi-winter
0.007033	Semi-winter
0.006317	Semi-winter
0.006065	Semi-winter
0.005585	Semi-winter
0.005381	Semi-winter
0.005056	Semi-winter
0.006112	Spring
0.006076	Spring
0.006077	Spring
0.005459	Spring
0.005979	Spring
0.006221	Spring
0.005803	Spring
0.005519	Spring
0.005705	Spring
0.005487	Spring

For Review Only

0.005454	Spring
0.00505	Spring
0.004875	Spring
0.004757	Spring
0.004052	Spring
0.003743	Spring
0.004397	Spring
0.004587	Spring
0.004465	Spring
0.004272	Spring
0.004001	Spring
0.004113	Spring
0.004375	Spring
0.004553	Spring
0.004291	Spring
0.004007	Spring
0.002956	Spring
0.002422	Spring
0.002183	Spring
0.002087	Spring
0.002047	Spring
0.001666	Spring
0.00095	Spring
0.00059	Spring
0.000404	Spring
0.000319	Spring
0.000761	Spring
0.001313	Spring
0.001529	Spring
0.001652	Spring
0.002658	Spring
0.003846	Spring
0.003989	Spring
0.004481	Spring
0.004778	Spring
0.005229	Spring
0.005286	Spring
0.005218	Spring
0.005261	Spring
0.005931	Spring
0.005614	Spring
0.005119	Spring
0.005716	Spring
0.005964	Spring
0.005834	Spring
0.005298	Spring
0.004964	Spring
0.004896	Spring
0.005106	Spring
0.004916	Spring
0.004819	Spring
0.004275	Spring
0.004264	Spring
0.00508	Spring
0.005157	Spring
0.005149	Spring
0.005195	Spring
0.005308	Spring
0.005052	Spring
0.00479	Spring

For Review Only

0.004783	Spring
0.005543	Spring
0.005	Spring
0.004091	Spring
0.004699	Spring
0.004956	Spring
0.005199	Spring
0.00476	Spring
0.005236	Spring
0.005123	Spring
0.00471	Spring
0.004114	Spring
0.004458	Spring
0.004239	Spring
0.004012	Spring
0.004359	Spring
0.004816	Spring
0.00608	Spring
0.006458	Spring
0.006622	Spring
0.006631	Spring
0.006652	Spring
0.006673	Spring
0.006966	Spring
0.007014	Spring
0.006712	Spring
0.005998	Spring
0.004879	Spring
0.003948	Spring
0.003753	Spring
0.004175	Spring
0.004137	Spring
0.003765	Spring
0.003152	Spring
0.003	Spring
0.003097	Spring
0.0033	Spring
0.003204	Spring
0.003358	Spring
0.00353	Spring
0.003284	Spring
0.003081	Spring
0.003256	Spring
0.003505	Spring
0.003184	Spring
0.003102	Spring
0.003042	Spring
0.0035	Spring
0.003692	Spring
0.003531	Spring
0.003491	Spring
0.003788	Spring
0.004208	Spring
0.004207	Spring
0.00455	Spring
0.004347	Spring
0.004132	Spring
0.003666	Spring
0.003432	Spring
0.003537	Spring

For Review Only

0.003486	Spring
0.003126	Spring
0.0025	Spring
0.002829	Spring
0.002552	Spring
0.002506	Spring
0.002798	Spring
0.003092	Spring
0.002901	Spring
0.002791	Spring
0.002921	Spring
0.002911	Spring
0.002778	Spring
0.002192	Spring
0.002105	Spring
0.002278	Spring
0.001985	Spring
0.001647	Spring
0.001681	Spring
0.001512	Spring
0.001169	Spring
0.001253	Spring
0.001269	Spring
0.00128	Spring
0.001128	Spring
0.000702	Spring
0.000593	Spring
0.000791	Spring
0.000953	Spring
0.001118	Spring
0.001437	Spring
0.001995	Spring
0.002438	Spring
0.002583	Spring
0.002691	Spring
0.003085	Spring
0.003732	Spring
0.004167	Spring
0.00472	Spring
0.004863	Spring
0.004856	Spring
0.004889	Spring
0.004974	Spring
0.004845	Spring
0.004968	Spring
0.004781	Spring
0.004334	Spring
0.004632	Spring
0.004185	Spring
0.004513	Spring
0.004494	Spring
0.004548	Spring
0.004109	Spring
0.004209	Spring
0.004686	Spring
0.004773	Spring
0.004731	Spring
0.003913	Spring
0.003909	Spring
0.003454	Spring

For Review Only

0.003275	Spring
0.002955	Spring
0.006709	Winter
0.006801	Winter
0.006882	Winter
0.006244	Winter
0.006512	Winter
0.006512	Winter
0.006287	Winter
0.006052	Winter
0.005977	Winter
0.005637	Winter
0.005497	Winter
0.004801	Winter
0.004446	Winter
0.004456	Winter
0.004007	Winter
0.003594	Winter
0.003826	Winter
0.00387	Winter
0.003881	Winter
0.003706	Winter
0.003513	Winter
0.003489	Winter
0.003715	Winter
0.00376	Winter
0.003386	Winter
0.003205	Winter
0.002363	Winter
0.00193	Winter
0.001678	Winter
0.001603	Winter
0.001557	Winter
0.001354	Winter
0.000864	Winter
0.000545	Winter
0.000402	Winter
0.000177	Winter
0.000231	Winter
0.000277	Winter
0.000308	Winter
0.000319	Winter
0.001235	Winter
0.002263	Winter
0.002309	Winter
0.002691	Winter
0.002928	Winter
0.003419	Winter
0.00358	Winter
0.003993	Winter
0.00418	Winter
0.004702	Winter
0.004541	Winter
0.004344	Winter
0.005133	Winter
0.005392	Winter
0.005368	Winter
0.004975	Winter
0.004946	Winter
0.004971	Winter

For Review Only

0.005125	Winter
0.005018	Winter
0.004832	Winter
0.004175	Winter
0.003988	Winter
0.004307	Winter
0.004239	Winter
0.004106	Winter
0.004053	Winter
0.003814	Winter
0.003614	Winter
0.003268	Winter
0.003228	Winter
0.003739	Winter
0.00325	Winter
0.002843	Winter
0.003607	Winter
0.00415	Winter
0.004622	Winter
0.004477	Winter
0.005037	Winter
0.005512	Winter
0.005129	Winter
0.004591	Winter
0.004758	Winter
0.004456	Winter
0.003832	Winter
0.003795	Winter
0.003858	Winter
0.004695	Winter
0.004839	Winter
0.004719	Winter
0.004676	Winter
0.004802	Winter
0.005034	Winter
0.005391	Winter
0.005621	Winter
0.005438	Winter
0.004903	Winter
0.004194	Winter
0.003437	Winter
0.003191	Winter
0.003751	Winter
0.003904	Winter
0.003553	Winter
0.003134	Winter
0.002936	Winter
0.003234	Winter
0.003209	Winter
0.003144	Winter
0.003291	Winter
0.003463	Winter
0.003201	Winter
0.00289	Winter
0.003066	Winter
0.003208	Winter
0.003105	Winter
0.002943	Winter
0.003107	Winter
0.003538	Winter

For Review Only

0.003708	Winter
0.003561	Winter
0.003415	Winter
0.003748	Winter
0.004303	Winter
0.004437	Winter
0.004704	Winter
0.004474	Winter
0.004374	Winter
0.003936	Winter
0.003969	Winter
0.004402	Winter
0.004684	Winter
0.00427	Winter
0.003501	Winter
0.003967	Winter
0.003992	Winter
0.004135	Winter
0.004393	Winter
0.004797	Winter
0.004401	Winter
0.004097	Winter
0.004199	Winter
0.004196	Winter
0.004156	Winter
0.003444	Winter
0.003296	Winter
0.003679	Winter
0.003473	Winter
0.003094	Winter
0.00325	Winter
0.003254	Winter
0.003043	Winter
0.003792	Winter
0.004019	Winter
0.004926	Winter
0.004714	Winter
0.00401	Winter
0.003847	Winter
0.004395	Winter
0.004909	Winter
0.005063	Winter
0.005268	Winter
0.005256	Winter
0.005485	Winter
0.004711	Winter
0.004716	Winter
0.005004	Winter
0.005734	Winter
0.005858	Winter
0.005911	Winter
0.005785	Winter
0.005554	Winter
0.005653	Winter
0.005698	Winter
0.005817	Winter
0.0063	Winter
0.006112	Winter
0.005497	Winter
0.005685	Winter

For Review Only

0.005374	Winter
0.005813	Winter
0.005983	Winter
0.005973	Winter
0.005512	Winter
0.005403	Winter
0.005566	Winter
0.006326	Winter
0.006579	Winter
0.005747	Winter
0.005487	Winter
0.005299	Winter
0.005012	Winter
0.004675	Winter
0.002187	Winter
0.00195	Winter
0.001799	Winter
0.001609	Winter
0.001477	Winter
0.001324	Winter
0.001415	Winter
0.001521	Winter
0.001561	Winter
0.001915	Winter
0.002317	Winter
0.002723	Winter
0.003257	Winter
0.003658	Winter
0.00418	Winter
0.004226	Winter
0.004378	Winter
0.00479	Winter
0.005031	Winter
0.004589	Winter
0.004062	Winter
0.003652	Winter
0.00341	Winter
0.003049	Winter
0.002669	Winter
0.002641	Winter
0.002501	Winter
0.002148	Winter
0.002241	Winter
0.002637	Winter
0.003397	Winter
0.003725	Winter
0.003436	Winter
0.003474	Winter
0.003325	Winter
0.00314	Winter
0.003243	Winter
0.003184	Winter
0.002979	Winter
0.002742	Winter
0.002185	Winter
0.001957	Winter
0.002045	Winter
0.001954	Winter
0.002141	Winter
0.002215	Winter

For Review Only

0.002142	Winter
0.00244	Winter
0.002402	Winter
0.002328	Winter
0.002881	Winter
0.00307	Winter
0.003117	Winter
0.003077	Winter
0.002903	Winter
0.002823	Winter
0.002922	Winter
0.002572	Winter
0.002823	Winter
0.003127	Winter
0.002786	Winter
0.002923	Winter
0.003187	Winter
0.004003	Winter
0.004398	Winter
0.004407	Winter
0.004235	Winter
0.004445	Winter
0.004251	Winter
0.004067	Winter
0.004076	Winter
0.004258	Winter
0.00415	Winter
0.003579	Winter
0.00329	Winter
0.003388	Winter
0.003685	Winter
0.003777	Winter
0.003826	Winter
0.003719	Winter
0.003396	Winter
0.002975	Winter
0.002996	Winter
0.002761	Winter
0.002696	Winter
0.002509	Winter
0.002172	Winter
0.001842	Winter
0.001575	Winter
0.001431	Winter
0.001474	Winter
0.001274	Winter
0.000953	Winter
0.000911	Winter
0.001723	Winter
0.001678	Winter
0.001813	Winter
0.002113	Winter
0.002415	Winter
0.002465	Winter
0.002651	Winter
0.002752	Winter
0.003044	Winter
0.003837	Winter
0.003784	Winter
0.004796	Winter

For Review Only

0.005411	Winter
0.005525	Winter
0.006105	Winter
0.006289	Winter
0.006278	Winter
0.006456	Winter
0.006659	Winter
0.006651	Winter
0.006915	Winter
0.007251	Winter
0.006691	Winter
0.006453	Winter
0.006268	Winter
0.006829	Winter
0.007142	Winter
0.007675	Winter
0.007666	Winter
0.007696	Winter
0.006881	Winter
0.005732	Winter
0.006143	Winter
0.006551	Winter
0.006625	Winter
0.006258	Winter
0.006479	Winter
0.006471	Winter
0.006148	Winter
0.005658	Winter
0.005774	Winter
0.005802	Winter
0.005121	Winter
0.005001	Winter
0.005072	Winter
0.005106	Winter
0.004519	Winter
0.003845	Winter
0.004374	Winter
0.004735	Winter
0.004726	Winter
0.005157	Winter
0.005434	Winter
0.005235	Winter
0.004595	Winter
0.004268	Winter
0.004509	Winter
0.004937	Winter
0.00483	Winter
0.004649	Winter
0.004724	Winter
0.004338	Winter
0.004468	Winter
0.004496	Winter
0.004793	Winter
0.005045	Winter
0.005084	Winter
0.004982	Winter
0.004711	Winter
0.004309	Winter
0.004303	Winter
0.004487	Winter

For Review Only

0.004374	Winter
0.004256	Winter
0.00393	Winter
0.003943	Winter
0.003774	Winter
0.003794	Winter
0.003511	Winter
0.003331	Winter
0.003605	Winter
0.003413	Winter
0.003052	Winter
0.00297	Winter
0.00359	Winter
0.003714	Winter
0.004087	Winter
0.003976	Winter
0.004035	Winter
0.004548	Winter
0.003906	Winter
0.00374	Winter
0.004235	Winter
0.004481	Winter
0.004781	Winter
0.00502	Winter
0.004551	Winter
0.004528	Winter
0.001642	Spring
0.001583	Spring
0.001586	Spring
0.001822	Spring
0.001785	Spring
0.002193	Spring
0.002345	Spring
0.002541	Spring
0.002558	Spring
0.003108	Spring
0.003597	Spring
0.004066	Spring
0.004717	Spring
0.004901	Spring
0.005275	Spring
0.00536	Spring
0.005495	Spring
0.006095	Spring
0.006604	Spring
0.005995	Spring
0.00533	Spring
0.004841	Spring
0.004354	Spring
0.00389	Spring
0.00358	Spring
0.003096	Spring
0.002715	Spring
0.001861	Spring
0.001552	Spring
0.001719	Spring
0.002215	Spring
0.002431	Spring
0.002263	Spring
0.002358	Spring

For Review Only

0.002221	Spring
0.002047	Spring
0.002129	Spring
0.002394	Spring
0.002446	Spring
0.002578	Spring
0.002355	Spring
0.002284	Spring
0.002501	Spring
0.002709	Spring
0.003169	Spring
0.003712	Spring
0.004138	Spring
0.004941	Spring
0.005068	Spring
0.004892	Spring
0.005612	Spring
0.005867	Spring
0.005964	Spring
0.005877	Spring
0.005474	Spring
0.005178	Spring
0.005267	Spring
0.004257	Spring
0.004196	Spring
0.004632	Spring
0.003999	Spring
0.003828	Spring
0.003961	Spring
0.004592	Spring
0.005294	Spring
0.005189	Spring
0.004752	Spring
0.00511	Spring
0.004947	Spring
0.00455	Spring
0.004507	Spring
0.00502	Spring
0.005049	Spring
0.004638	Spring
0.004215	Spring
0.004285	Spring
0.004313	Spring
0.004181	Spring
0.004074	Spring
0.003971	Spring
0.003763	Spring
0.003305	Spring
0.003257	Spring
0.002881	Spring
0.002696	Spring
0.002577	Spring
0.00247	Spring
0.002266	Spring
0.00211	Spring
0.001998	Spring
0.002103	Spring
0.001878	Spring
0.0015	Spring
0.001639	Spring

For Review Only

0.002204	Spring
0.00219	Spring
0.002353	Spring
0.002456	Spring
0.002901	Spring
0.002911	Spring
0.003126	Spring
0.00315	Spring
0.003404	Spring
0.003578	Spring
0.003526	Spring
0.003931	Spring
0.004432	Spring
0.00465	Spring
0.004833	Spring
0.004945	Spring
0.004853	Spring
0.005105	Spring
0.005211	Spring
0.005806	Spring
0.006143	Spring
0.006515	Spring
0.005918	Spring
0.005635	Spring
0.005806	Spring
0.006129	Spring
0.006265	Spring
0.006576	Spring
0.006741	Spring
0.006595	Spring
0.005898	Spring
0.005312	Spring
0.005779	Spring
0.006408	Spring
0.006512	Spring
0.006552	Spring
0.006915	Spring
0.007336	Spring
0.007026	Spring
0.007012	Spring
0.007177	Spring
0.006978	Spring
0.006332	Spring
0.006441	Spring
0.006539	Spring
0.006632	Spring
0.006154	Spring
0.005328	Spring
0.006081	Spring
0.006159	Spring
0.005886	Spring
0.006264	Spring
0.006472	Spring
0.005879	Spring
0.005022	Spring
0.004553	Spring
0.00456	Spring
0.004903	Spring
0.004506	Spring
0.004113	Spring

For Review Only

0.004166	Spring
0.003754	Spring
0.003858	Spring
0.003932	Spring
0.004295	Spring
0.00463	Spring
0.004784	Spring
0.00461	Spring
0.004296	Spring
0.00386	Spring
0.003987	Spring
0.0042	Spring
0.004171	Spring
0.004134	Spring
0.003857	Spring
0.003922	Spring
0.004159	Spring
0.004443	Spring
0.004176	Spring
0.00405	Spring
0.004134	Spring
0.004086	Spring
0.003763	Spring
0.003502	Spring
0.003736	Spring
0.003499	Spring
0.003122	Spring
0.00265	Spring
0.002657	Spring
0.00307	Spring
0.002704	Spring
0.002614	Spring
0.003151	Spring
0.003415	Spring
0.004402	Spring
0.0049	Spring
0.00484	Spring
0.004986	Spring
0.00133	Semi-winter
0.001269	Semi-winter
0.001317	Semi-winter
0.001467	Semi-winter
0.001413	Semi-winter
0.001668	Semi-winter
0.001841	Semi-winter
0.001981	Semi-winter
0.001998	Semi-winter
0.002289	Semi-winter
0.002625	Semi-winter
0.002907	Semi-winter
0.003268	Semi-winter
0.003344	Semi-winter
0.003597	Semi-winter
0.003548	Semi-winter
0.003562	Semi-winter
0.003992	Semi-winter
0.004299	Semi-winter
0.003897	Semi-winter
0.00343	Semi-winter
0.003132	Semi-winter

For Review Only

0.002875	Semi-winter
0.002657	Semi-winter
0.00249	Semi-winter
0.002338	Semi-winter
0.00217	Semi-winter
0.001614	Semi-winter
0.001575	Semi-winter
0.001766	Semi-winter
0.002133	Semi-winter
0.002268	Semi-winter
0.002091	Semi-winter
0.002083	Semi-winter
0.00195	Semi-winter
0.001778	Semi-winter
0.001787	Semi-winter
0.00188	Semi-winter
0.001729	Semi-winter
0.001693	Semi-winter
0.001522	Semi-winter
0.001458	Semi-winter
0.001594	Semi-winter
0.001708	Semi-winter
0.001972	Semi-winter
0.002268	Semi-winter
0.00259	Semi-winter
0.003186	Semi-winter
0.003276	Semi-winter
0.003204	Semi-winter
0.003668	Semi-winter
0.003889	Semi-winter
0.004036	Semi-winter
0.003976	Semi-winter
0.003761	Semi-winter
0.003759	Semi-winter
0.003829	Semi-winter
0.003182	Semi-winter
0.003425	Semi-winter
0.003812	Semi-winter
0.003571	Semi-winter
0.003657	Semi-winter
0.003867	Semi-winter
0.004578	Semi-winter
0.005154	Semi-winter
0.005012	Semi-winter
0.004712	Semi-winter
0.004941	Semi-winter
0.004656	Semi-winter
0.004553	Semi-winter
0.004634	Semi-winter
0.004957	Semi-winter
0.004929	Semi-winter
0.004536	Semi-winter
0.00441	Semi-winter
0.004654	Semi-winter
0.004918	Semi-winter
0.00488	Semi-winter
0.004836	Semi-winter
0.004601	Semi-winter
0.004217	Semi-winter
0.003932	Semi-winter

For Review Only

0.004052	Semi-winter
0.003773	Semi-winter
0.003474	Semi-winter
0.003262	Semi-winter
0.002998	Semi-winter
0.002885	Semi-winter
0.002718	Semi-winter
0.002631	Semi-winter
0.002799	Semi-winter
0.002465	Semi-winter
0.001971	Semi-winter
0.002086	Semi-winter
0.002702	Semi-winter
0.00268	Semi-winter
0.002806	Semi-winter
0.002868	Semi-winter
0.003256	Semi-winter
0.003228	Semi-winter
0.003667	Semi-winter
0.003718	Semi-winter
0.00415	Semi-winter
0.00454	Semi-winter
0.004573	Semi-winter
0.005152	Semi-winter
0.00579	Semi-winter
0.006088	Semi-winter
0.006588	Semi-winter
0.006793	Semi-winter
0.006505	Semi-winter
0.006775	Semi-winter
0.006731	Semi-winter
0.007106	Semi-winter
0.007391	Semi-winter
0.007575	Semi-winter
0.006773	Semi-winter
0.006389	Semi-winter
0.006218	Semi-winter
0.006549	Semi-winter
0.006572	Semi-winter
0.006859	Semi-winter
0.006953	Semi-winter
0.006822	Semi-winter
0.006185	Semi-winter
0.005565	Semi-winter
0.006083	Semi-winter
0.00679	Semi-winter
0.006781	Semi-winter
0.006598	Semi-winter
0.006863	Semi-winter
0.006963	Semi-winter
0.006689	Semi-winter
0.006365	Semi-winter
0.006464	Semi-winter
0.006385	Semi-winter
0.005783	Semi-winter
0.005434	Semi-winter
0.005557	Semi-winter
0.005666	Semi-winter
0.005236	Semi-winter
0.004625	Semi-winter

For Review Only

0.005033	Semi-winter
0.004843	Semi-winter
0.004503	Semi-winter
0.004766	Semi-winter
0.004933	Semi-winter
0.004665	Semi-winter
0.003997	Semi-winter
0.00368	Semi-winter
0.003779	Semi-winter
0.004067	Semi-winter
0.004033	Semi-winter
0.004282	Semi-winter
0.004419	Semi-winter
0.004133	Semi-winter
0.00421	Semi-winter
0.004208	Semi-winter
0.004531	Semi-winter
0.004709	Semi-winter
0.004865	Semi-winter
0.004893	Semi-winter
0.004679	Semi-winter
0.004146	Semi-winter
0.00417	Semi-winter
0.004354	Semi-winter
0.004324	Semi-winter
0.004332	Semi-winter
0.004018	Semi-winter
0.004123	Semi-winter
0.004163	Semi-winter
0.004104	Semi-winter
0.003734	Semi-winter
0.003593	Semi-winter
0.00378	Semi-winter
0.003823	Semi-winter
0.003533	Semi-winter
0.003537	Semi-winter
0.004037	Semi-winter
0.004009	Semi-winter
0.004001	Semi-winter
0.003848	Semi-winter
0.003896	Semi-winter
0.004308	Semi-winter
0.0039	Semi-winter
0.003668	Semi-winter
0.004226	Semi-winter
0.00447	Semi-winter
0.004667	Semi-winter
0.005024	Semi-winter
0.004849	Semi-winter
0.004908	Semi-winter
0.001254	Semi-winter
0.001134	Semi-winter
0.00112	Semi-winter
0.001128	Semi-winter
0.001065	Semi-winter
0.001338	Semi-winter
0.00156	Semi-winter
0.001659	Semi-winter
0.001688	Semi-winter
0.002089	Semi-winter

For Review Only

0.002616	Semi-winter
0.003338	Semi-winter
0.003939	Semi-winter
0.004227	Semi-winter
0.004489	Semi-winter
0.004754	Semi-winter
0.005029	Semi-winter
0.005794	Semi-winter
0.006133	Semi-winter
0.005988	Semi-winter
0.005412	Semi-winter
0.00495	Semi-winter
0.004457	Semi-winter
0.004164	Semi-winter
0.003944	Semi-winter
0.003326	Semi-winter
0.002953	Semi-winter
0.002227	Semi-winter
0.002154	Semi-winter
0.002063	Semi-winter
0.002557	Semi-winter
0.002518	Semi-winter
0.002494	Semi-winter
0.002507	Semi-winter
0.00241	Semi-winter
0.002481	Semi-winter
0.002467	Semi-winter
0.00242	Semi-winter
0.002291	Semi-winter
0.002245	Semi-winter
0.001908	Semi-winter
0.001781	Semi-winter
0.00188	Semi-winter
0.002112	Semi-winter
0.002383	Semi-winter
0.002552	Semi-winter
0.002848	Semi-winter
0.003412	Semi-winter
0.00347	Semi-winter
0.003392	Semi-winter
0.00399	Semi-winter
0.004195	Semi-winter
0.004301	Semi-winter
0.004278	Semi-winter
0.004099	Semi-winter
0.004196	Semi-winter
0.004323	Semi-winter
0.003736	Semi-winter
0.004162	Semi-winter
0.004621	Semi-winter
0.004183	Semi-winter
0.004215	Semi-winter
0.00442	Semi-winter
0.005146	Semi-winter
0.005887	Semi-winter
0.005713	Semi-winter
0.005704	Semi-winter
0.006103	Semi-winter
0.005668	Semi-winter
0.005455	Semi-winter

For Review Only

0.005568	Semi-winter
0.006047	Semi-winter
0.006041	Semi-winter
0.005579	Semi-winter
0.005299	Semi-winter
0.005555	Semi-winter
0.005535	Semi-winter
0.00534	Semi-winter
0.00535	Semi-winter
0.005168	Semi-winter
0.004928	Semi-winter
0.004564	Semi-winter
0.004737	Semi-winter
0.004365	Semi-winter
0.00404	Semi-winter
0.003916	Semi-winter
0.003591	Semi-winter
0.003402	Semi-winter
0.003187	Semi-winter
0.003063	Semi-winter
0.003111	Semi-winter
0.002743	Semi-winter
0.002276	Semi-winter
0.00248	Semi-winter
0.003083	Semi-winter
0.002981	Semi-winter
0.003143	Semi-winter
0.003227	Semi-winter
0.003712	Semi-winter
0.003724	Semi-winter
0.004182	Semi-winter
0.004279	Semi-winter
0.004714	Semi-winter
0.004954	Semi-winter
0.005037	Semi-winter
0.005554	Semi-winter
0.006261	Semi-winter
0.006639	Semi-winter
0.007128	Semi-winter
0.007314	Semi-winter
0.00718	Semi-winter
0.007475	Semi-winter
0.007454	Semi-winter
0.0077	Semi-winter
0.008191	Semi-winter
0.008669	Semi-winter
0.007897	Semi-winter
0.00746	Semi-winter
0.007593	Semi-winter
0.007937	Semi-winter
0.008157	Semi-winter
0.008372	Semi-winter
0.008482	Semi-winter
0.008421	Semi-winter
0.007864	Semi-winter
0.007168	Semi-winter
0.00754	Semi-winter
0.008093	Semi-winter
0.007584	Semi-winter
0.007603	Semi-winter

For Review Only

0.007747	Semi-winter
0.00818	Semi-winter
0.007897	Semi-winter
0.00784	Semi-winter
0.007806	Semi-winter
0.007515	Semi-winter
0.00693	Semi-winter
0.006899	Semi-winter
0.007343	Semi-winter
0.007437	Semi-winter
0.007035	Semi-winter
0.006187	Semi-winter
0.007007	Semi-winter
0.006791	Semi-winter
0.006329	Semi-winter
0.006678	Semi-winter
0.006973	Semi-winter
0.006535	Semi-winter
0.005652	Semi-winter
0.00516	Semi-winter
0.005072	Semi-winter
0.005602	Semi-winter
0.005266	Semi-winter
0.005613	Semi-winter
0.005747	Semi-winter
0.005338	Semi-winter
0.00532	Semi-winter
0.005473	Semi-winter
0.006057	Semi-winter
0.006201	Semi-winter
0.006325	Semi-winter
0.00634	Semi-winter
0.005971	Semi-winter
0.005304	Semi-winter
0.005437	Semi-winter
0.005814	Semi-winter
0.005892	Semi-winter
0.005776	Semi-winter
0.005248	Semi-winter
0.005402	Semi-winter
0.005558	Semi-winter
0.005478	Semi-winter
0.005019	Semi-winter
0.004802	Semi-winter
0.004944	Semi-winter
0.005235	Semi-winter
0.005029	Semi-winter
0.005241	Semi-winter
0.005875	Semi-winter
0.005756	Semi-winter
0.005906	Semi-winter
0.0056	Semi-winter
0.005697	Semi-winter
0.00631	Semi-winter
0.005738	Semi-winter
0.005023	Semi-winter
0.005538	Semi-winter
0.005976	Semi-winter
0.006457	Semi-winter
0.007137	Semi-winter

For Review Only

0.006692	Semi-winter
0.006792	Semi-winter
0.00178	Spring
0.001664	Spring
0.001631	Spring
0.001732	Spring
0.001688	Spring
0.002111	Spring
0.002291	Spring
0.002379	Spring
0.002377	Spring
0.002942	Spring
0.003569	Spring
0.004347	Spring
0.00518	Spring
0.005596	Spring
0.005931	Spring
0.006135	Spring
0.00639	Spring
0.007207	Spring
0.007732	Spring
0.00734	Spring
0.00659	Spring
0.006131	Spring
0.005439	Spring
0.004884	Spring
0.004586	Spring
0.003857	Spring
0.003445	Spring
0.002679	Spring
0.002373	Spring
0.002334	Spring
0.002842	Spring
0.002727	Spring
0.002682	Spring
0.002767	Spring
0.002649	Spring
0.002617	Spring
0.00263	Spring
0.002647	Spring
0.002668	Spring
0.002741	Spring
0.002475	Spring
0.002436	Spring
0.002671	Spring
0.003013	Spring
0.003439	Spring
0.003839	Spring
0.004173	Spring
0.005007	Spring
0.005188	Spring
0.005011	Spring
0.00583	Spring
0.006053	Spring
0.006094	Spring
0.006027	Spring
0.005689	Spring
0.005672	Spring
0.005851	Spring
0.004865	Spring

For Review Only

0.005005	Spring
0.005489	Spring
0.004706	Spring
0.004557	Spring
0.004849	Spring
0.005701	Spring
0.006614	Spring
0.006476	Spring
0.006573	Spring
0.007099	Spring
0.006689	Spring
0.006214	Spring
0.006192	Spring
0.006759	Spring
0.006684	Spring
0.005876	Spring
0.00532	Spring
0.00524	Spring
0.004789	Spring
0.004473	Spring
0.004434	Spring
0.004392	Spring
0.004234	Spring
0.003802	Spring
0.003734	Spring
0.003394	Spring
0.003171	Spring
0.003263	Spring
0.003106	Spring
0.002872	Spring
0.002672	Spring
0.002558	Spring
0.002664	Spring
0.002349	Spring
0.00204	Spring
0.002367	Spring
0.002902	Spring
0.002703	Spring
0.002845	Spring
0.002862	Spring
0.003417	Spring
0.003435	Spring
0.00367	Spring
0.00372	Spring
0.003819	Spring
0.003738	Spring
0.003693	Spring
0.003899	Spring
0.004413	Spring
0.004665	Spring
0.004601	Spring
0.004722	Spring
0.004713	Spring
0.004926	Spring
0.005103	Spring
0.005463	Spring
0.005748	Spring
0.006336	Spring
0.005806	Spring
0.0055	Spring

For Review Only

0.006012	Spring
0.006191	Spring
0.006524	Spring
0.00678	Spring
0.006894	Spring
0.006804	Spring
0.006146	Spring
0.005503	Spring
0.005785	Spring
0.006344	Spring
0.006122	Spring
0.006327	Spring
0.006578	Spring
0.007328	Spring
0.007215	Spring
0.007643	Spring
0.007783	Spring
0.007531	Spring
0.006995	Spring
0.007221	Spring
0.007417	Spring
0.007555	Spring
0.00698	Spring
0.005861	Spring
0.006754	Spring
0.006551	Spring
0.006293	Spring
0.006661	Spring
0.006888	Spring
0.006264	Spring
0.005391	Spring
0.004867	Spring
0.004775	Spring
0.005268	Spring
0.004621	Spring
0.004439	Spring
0.004486	Spring
0.004131	Spring
0.004223	Spring
0.004486	Spring
0.004958	Spring
0.005222	Spring
0.005383	Spring
0.005194	Spring
0.00475	Spring
0.00419	Spring
0.004309	Spring
0.004558	Spring
0.004573	Spring
0.004336	Spring
0.003965	Spring
0.004005	Spring
0.004196	Spring
0.004486	Spring
0.004296	Spring
0.004175	Spring
0.004251	Spring
0.004363	Spring
0.004078	Spring
0.003926	Spring

For Review Only

0.004214	Spring
0.003915	Spring
0.003667	Spring
0.003142	Spring
0.003099	Spring
0.003677	Spring
0.00328	Spring
0.002926	Spring
0.003422	Spring
0.003791	Spring
0.0048	Spring
0.005433	Spring
0.005217	Spring
0.005396	Spring
0.001943	Winter
0.001654	Winter
0.001478	Winter
0.001191	Winter
0.001096	Winter
0.000764	Winter
0.000787	Winter
0.000839	Winter
0.000864	Winter
0.001204	Winter
0.001696	Winter
0.00222	Winter
0.002863	Winter
0.003286	Winter
0.00371	Winter
0.003963	Winter
0.004264	Winter
0.004726	Winter
0.004826	Winter
0.004475	Winter
0.003947	Winter
0.003482	Winter
0.003129	Winter
0.002833	Winter
0.00253	Winter
0.002217	Winter
0.001984	Winter
0.00176	Winter
0.001863	Winter
0.002241	Winter
0.003178	Winter
0.003406	Winter
0.003196	Winter
0.003283	Winter
0.003179	Winter
0.003205	Winter
0.003313	Winter
0.003054	Winter
0.002889	Winter
0.002538	Winter
0.001747	Winter
0.001594	Winter
0.001584	Winter
0.001438	Winter
0.001531	Winter
0.001456	Winter

For Review Only

0.001247	Winter
0.001457	Winter
0.001455	Winter
0.001436	Winter
0.002043	Winter
0.002133	Winter
0.002133	Winter
0.002055	Winter
0.001958	Winter
0.001963	Winter
0.002065	Winter
0.001951	Winter
0.002534	Winter
0.002837	Winter
0.002455	Winter
0.002576	Winter
0.003085	Winter
0.004371	Winter
0.004977	Winter
0.005158	Winter
0.005654	Winter
0.006046	Winter
0.005623	Winter
0.005584	Winter
0.005546	Winter
0.006011	Winter
0.005894	Winter
0.004986	Winter
0.004666	Winter
0.0047	Winter
0.004675	Winter
0.004685	Winter
0.004821	Winter
0.004608	Winter
0.004402	Winter
0.003885	Winter
0.003951	Winter
0.00366	Winter
0.003556	Winter
0.003622	Winter
0.003172	Winter
0.002749	Winter
0.002396	Winter
0.002305	Winter
0.002391	Winter
0.002138	Winter
0.001814	Winter
0.002072	Winter
0.002733	Winter
0.002497	Winter
0.002509	Winter
0.002814	Winter
0.00332	Winter
0.003406	Winter
0.003626	Winter
0.003748	Winter
0.003945	Winter
0.004354	Winter
0.004259	Winter
0.004909	Winter

For Review Only

0.005372	Winter
0.005513	Winter
0.005801	Winter
0.005921	Winter
0.005972	Winter
0.006157	Winter
0.006418	Winter
0.006429	Winter
0.006894	Winter
0.007441	Winter
0.007175	Winter
0.006859	Winter
0.007053	Winter
0.00746	Winter
0.00794	Winter
0.008235	Winter
0.008128	Winter
0.008266	Winter
0.007382	Winter
0.006329	Winter
0.006575	Winter
0.006934	Winter
0.006576	Winter
0.006414	Winter
0.006424	Winter
0.006763	Winter
0.006491	Winter
0.006187	Winter
0.006325	Winter
0.006293	Winter
0.005725	Winter
0.005791	Winter
0.006041	Winter
0.006198	Winter
0.005659	Winter
0.004867	Winter
0.00573	Winter
0.005682	Winter
0.005658	Winter
0.0061	Winter
0.0064	Winter
0.006057	Winter
0.005375	Winter
0.004897	Winter
0.005039	Winter
0.00568	Winter
0.005298	Winter
0.005525	Winter
0.005531	Winter
0.005098	Winter
0.005192	Winter
0.005353	Winter
0.005756	Winter
0.005856	Winter
0.005796	Winter
0.005637	Winter
0.005208	Winter
0.004667	Winter
0.004723	Winter
0.005075	Winter

For Review Only

0.005019	Winter
0.004636	Winter
0.004143	Winter
0.004084	Winter
0.003872	Winter
0.00373	Winter
0.003498	Winter
0.00326	Winter
0.00341	Winter
0.003354	Winter
0.003046	Winter
0.003249	Winter
0.003956	Winter
0.004188	Winter
0.004915	Winter
0.004817	Winter
0.00487	Winter
0.005508	Winter
0.004924	Winter
0.004458	Winter
0.004938	Winter
0.005313	Winter
0.005364	Winter
0.005629	Winter
0.004798	Winter
0.004775	Winter

For Review Only

Table S4. The haplotyes information of *BnFT.A02* in BnaWGS-1007 panel

chrom	pos	ref	alt	Hap1	Hap2	Hap3	Hap4	Hap5
scaffoldA02	9104606	A	C	0	0	0	2	0
scaffoldA02	9104747	C	T	0	0	0	2	0
scaffoldA02	9104831	A	G	0	0	0	2	0
scaffoldA02	9104965	A	T	0	0	0	2	0
scaffoldA02	9105073	T	A	0	0	0	0	0
scaffoldA02	9105131	A	T	0	0	0	0	0
scaffoldA02	9105659	A	T	0	0	0	0	0
scaffoldA02	9105719	T	C	0	0	0	0	0
scaffoldA02	9105902	A	G	0	0	0	0	1
scaffoldA02	9105930	A	G	0	0	0	0	1
scaffoldA02	9105996	G	A	0	0	0	0	0
scaffoldA02	9106045	A	T	0	0	0	0	1
scaffoldA02	9106080	T	C	0	0	0	0	1
scaffoldA02	9106142	T	C	0	0	0	0	0
scaffoldA02	9106175	G	T	0	0	0	0	1
scaffoldA02	9106789	A	C	0	2	1	2	2
scaffoldA02	9106850	A	G	0	0	0	0	0
scaffoldA02	9106904	A	G	0	0	0	0	1
scaffoldA02	9106911	A	G	0	0	0	0	1
scaffoldA02	9106919	A	T	0	0	0	0	1

For Review Only

Hap6	Hap7	Hap8	Hap9	Hap10
2	0	0	0	2
0	0	0	0	0
0	0	0	0	0
0	0	0	0	2
2	0	0	0	2
2	0	0	0	2
2	0	0	0	2
2	0	0	0	2
0	1	1	1	0
0	1	1	1	0
2	0	0	0	2
0	1	1	1	0
0	1	1	0	0
2	0	0	0	2
0	1	1	0	0
2	0	1	1	2
2	0	0	0	2
0	1	1	1	0
0	1	1	1	0
0	1	1	1	0

For Review Only

Table S5. The haplotyes information of *BnFT.A02* in BnaWGS-655 panel

chrom	pos	ref	alt	Hap1	Hap2	Hap3	Hap4	Hap5
scaffoldA02	9104027	C	A	0	0	2	1	0
scaffoldA02	9104362	C	T	0	0	0	0	2
scaffoldA02	9104606	A	C	0	2	0	0	0
scaffoldA02	9104747	C	T	0	2	0	0	0
scaffoldA02	9104831	A	G	0	2	0	0	0
scaffoldA02	9104965	A	T	0	2	0	0	0
scaffoldA02	9105073	T	A	0	0	0	0	0
scaffoldA02	9105131	A	T	0	0	0	0	0
scaffoldA02	9105659	A	T	0	0	0	0	0
scaffoldA02	9105719	T	C	0	0	0	0	0
scaffoldA02	9105833	G	A	0	0	0	0	0
scaffoldA02	9105930	A	G	0	0	0	0	0
scaffoldA02	9105996	G	A	0	0	0	0	0
scaffoldA02	9106045	A	T	0	0	0	0	0
scaffoldA02	9106079	G	A	0	0	0	0	0
scaffoldA02	9106080	T	C	0	0	0	0	0
scaffoldA02	9106142	T	C	0	0	0	0	0
scaffoldA02	9106175	G	T	0	0	0	0	0
scaffoldA02	9106789	A	C	0	2	0	0	2
scaffoldA02	9106850	A	G	0	0	0	0	0

For Review Only

Hap6	Hap7	Hap8	Hap9	Hap10
0	0	0	0	0
0	0	0	0	0
2	2	2	2	2
0	0	0	2	2
0	0	0	1	2
2	0	2	2	0
0	2	0	0	0
0	2	0	0	0
0	2	0	0	0
0	2	0	0	0
0	0	0	0	0
0	0	1	0	0
0	1	0	0	0
0	0	0	0	0
0	0	0	0	0
0	0	0	0	0
0	1	0	0	0
0	0	0	0	0
2	2	2	2	2
0	2	0	0	0

For Review Only

Table S6. The haplotyes information of *BnFLC.A10* in BnaWGS-1007 panel

chrom	pos	ref	alt	Hap1	Hap2	Hap3	Hap4	Hap5
scaffoldA10	23941998	G	C	0	0	1	0	1
scaffoldA10	23942077	C	A	2	2	2	0	2
scaffoldA10	23942097	A	C	0	0	1	0	1
scaffoldA10	23942248	A	G	0	1	0	0	0
scaffoldA10	23942642	A	G	0	0	1	0	0
scaffoldA10	23942852	A	G	2	2	2	0	2
scaffoldA10	23943181	A	C	2	2	2	0	2
scaffoldA10	23943692	C	A	2	2	2	0	2
scaffoldA10	23943700	C	T	2	2	2	0	2
scaffoldA10	23943940	T	G	2	2	2	0	2
scaffoldA10	23944120	C	T	2	2	2	0	2
scaffoldA10	23944231	T	A	2	2	2	0	2
scaffoldA10	23944644	A	G	2	2	2	0	2
scaffoldA10	23944996	C	T	2	2	2	0	2
scaffoldA10	23945470	G	C	2	2	2	0	2
scaffoldA10	23945829	G	A	2	2	2	0	2
scaffoldA10	23946475	G	T	2	2	2	0	2
scaffoldA10	23947242	T	C	2	2	2	0	2
scaffoldA10	23947405	A	G	2	2	2	0	2

For Review Only

**Hap6**	**Hap7**	**Hap8**	**Hap9**	**Hap10**
0	0	0	0	0
1	2	2	2	2
0	0	0	0	0
0	0	0	0	0
0	0	0	1	0
0	2	2	2	2
0	2	2	2	2
0	2	0	2	2
0	0	0	2	2
0	2	2	2	2
0	2	2	2	2
0	2	2	2	2
0	2	2	2	2
0	2	2	2	2
0	2	2	2	2
0	2	2	2	2
0	2	2	2	1
0	2	2	2	2
0	2	2	2	2

For Review Only

Table S7. The haplotyes information of *BnFLC.A10* in BnaWGS-655 panel

chrom	pos	ref	alt	Hap1	Hap2	Hap3	Hap4	Hap5
scaffoldA10	23942511	G	A	0	0	2	1	0
scaffoldA10	23942852	A	G	0	2	0	0	2
scaffoldA10	23943181	A	C	0	2	0	0	2
scaffoldA10	23943692	C	A	0	2	2	2	0
scaffoldA10	23943700	C	T	0	2	0	0	0
scaffoldA10	23943940	T	G	0	2	0	0	2
scaffoldA10	23944120	C	T	0	2	0	0	2
scaffoldA10	23944231	T	A	0	2	0	0	2
scaffoldA10	23944644	A	G	0	2	0	0	2
scaffoldA10	23944996	C	T	0	2	0	0	2
scaffoldA10	23945470	G	C	0	2	0	0	2
scaffoldA10	23945829	G	A	0	2	0	0	2
scaffoldA10	23946475	G	T	0	2	0	0	2
scaffoldA10	23947242	T	C	0	2	0	0	2
scaffoldA10	23947405	A	G	0	2	2	2	2

For Review Only

Hap6	Hap7	Hap8	Hap9	Hap10
0	0	0	0	0
2	2	2	2	2
2	2	0	2	2
2	2	2	0	1
0	1	0	1	0
2	2	0	2	2
2	2	0	2	2
2	2	0	2	2
2	2	2	2	2
2	2	2	2	2
2	2	2	2	2
2	2	0	2	2
2	2	0	2	2
2	2	0	2	2
2	2	2	2	2

For Review Only

Table S8. The one-way ANOVA statistics results for *BnFT.A02* and *BnFLC.A10*

panel	group1	group2	meandiff	p-adj	lower	upper	reject
BnaWGS-1007	Hap1	Hap2	2.939	0.0109	0.6801	5.1979	TRUE
BnaWGS-655	Hap1	Hap2	-13.4003	0	-19.0192	-7.7813	TRUE
BnaWGS-655	Hap1	Hap3	-14.7166	0	-20.6573	-8.776	TRUE
BnaWGS-655	Hap1	Hap4	-16.5551	0	-23.7801	-9.3301	TRUE
BnaWGS-655	Hap1	Hap5	-13.2096	0.0001	-21.3787	-5.0406	TRUE
BnaWGS-655	Hap1	Hap6	-15.4551	0	-23.9512	-6.959	TRUE
BnaWGS-655	Hap1	Hap7	-14.898	0.0003	-24.7912	-5.0048	TRUE
BnaWGS-655	Hap2	Hap3	-1.3164	0.9966	-7.8276	5.1948	FALSE
BnaWGS-655	Hap2	Hap4	-3.1548	0.8832	-10.8558	4.5461	FALSE
BnaWGS-655	Hap2	Hap5	0.1906	1	-8.4022	8.7835	FALSE
BnaWGS-655	Hap2	Hap6	-2.0548	0.993	-10.9592	6.8495	FALSE
BnaWGS-655	Hap2	Hap7	-1.4977	0.9995	-11.7436	8.7482	FALSE
BnaWGS-655	Hap3	Hap4	-1.8385	0.9928	-9.7772	6.1002	FALSE
BnaWGS-655	Hap3	Hap5	1.507	0.9987	-7.2996	10.3136	FALSE
BnaWGS-655	Hap3	Hap6	-0.7385	1	-9.8492	8.3723	FALSE
BnaWGS-655	Hap3	Hap7	-0.1813	1	-10.6072	10.2445	FALSE
BnaWGS-655	Hap4	Hap5	3.3455	0.9464	-6.3738	13.0647	FALSE
BnaWGS-655	Hap4	Hap6	1.1	0.9999	-8.8957	11.0957	FALSE
BnaWGS-655	Hap4	Hap7	1.6571	0.9994	-9.5503	12.8646	FALSE
BnaWGS-655	Hap5	Hap6	-2.2455	0.9958	-12.9435	8.4526	FALSE
BnaWGS-655	Hap5	Hap7	-1.6883	0.9995	-13.5264	10.1498	FALSE
BnaWGS-655	Hap6	Hap7	0.5571	1	-11.5089	12.6232	FALSE
BnaWGS-1007	Hap1	Hap10	-1.1555	1	-7.3962	5.0852	FALSE
BnaWGS-1007	Hap1	Hap11	-15.5722	0	-23.1543	-7.99	TRUE
BnaWGS-1007	Hap1	Hap12	5.0778	0.3742	-1.7313	11.8869	FALSE
BnaWGS-1007	Hap1	Hap2	-0.1666	1	-3.5946	3.2614	FALSE
BnaWGS-1007	Hap1	Hap3	-0.2507	1	-4.4645	3.963	FALSE
BnaWGS-1007	Hap1	Hap4	-14.0064	0	-19.0347	-8.9781	TRUE
BnaWGS-1007	Hap1	Hap5	-2.0222	0.9713	-6.9327	2.8883	FALSE
BnaWGS-1007	Hap1	Hap6	-16.7222	0	-22.3373	-11.107	TRUE
BnaWGS-1007	Hap1	Hap7	-6.0145	0.0495	-12.0223	-0.0067	TRUE
BnaWGS-1007	Hap1	Hap8	-1.0222	1	-7.8313	5.7869	FALSE
BnaWGS-1007	Hap1	Hap9	-1.1222	1	-7.9313	5.6869	FALSE
BnaWGS-1007	Hap10	Hap11	-14.4167	0.0001	-24.0454	-4.788	TRUE
BnaWGS-1007	Hap10	Hap12	6.2333	0.5011	-2.7992	15.2658	FALSE
BnaWGS-1007	Hap10	Hap2	0.9889	1	-5.8649	7.8426	FALSE
BnaWGS-1007	Hap10	Hap3	0.9048	1	-6.3738	8.1834	FALSE
BnaWGS-1007	Hap10	Hap4	-12.8509	0	-20.6295	-5.0723	TRUE
BnaWGS-1007	Hap10	Hap5	-0.8667	1	-8.5696	6.8363	FALSE
BnaWGS-1007	Hap10	Hap6	-15.5667	0	-23.7369	-7.3965	TRUE
BnaWGS-1007	Hap10	Hap7	-4.859	0.7643	-13.3039	3.5859	FALSE
BnaWGS-1007	Hap10	Hap8	0.1333	1	-8.8992	9.1658	FALSE
BnaWGS-1007	Hap10	Hap9	0.0333	1	-8.9992	9.0658	FALSE
BnaWGS-1007	Hap11	Hap12	20.65	0	10.6436	30.6564	TRUE
BnaWGS-1007	Hap11	Hap2	15.4056	0	7.3113	23.4998	TRUE
BnaWGS-1007	Hap11	Hap3	15.3214	0	6.8645	23.7784	TRUE
BnaWGS-1007	Hap11	Hap4	1.5658	1	-7.3251	10.4567	FALSE
BnaWGS-1007	Hap11	Hap5	13.55	0	4.7252	22.3748	TRUE
BnaWGS-1007	Hap11	Hap6	-1.15	1	-10.3855	8.0855	FALSE
BnaWGS-1007	Hap11	Hap7	9.5577	0.046	0.0783	19.0371	TRUE
BnaWGS-1007	Hap11	Hap8	14.55	0.0002	4.5436	24.5564	TRUE
BnaWGS-1007	Hap11	Hap9	14.45	0.0002	4.4436	24.4564	TRUE
BnaWGS-1007	Hap12	Hap2	-5.2444	0.4518	-12.6195	2.1306	FALSE
BnaWGS-1007	Hap12	Hap3	-5.3286	0.5116	-13.1	2.4428	FALSE
BnaWGS-1007	Hap12	Hap4	-19.0842	0	-27.3258	-10.8426	TRUE
BnaWGS-1007	Hap12	Hap5	-7.1	0.161	-15.2702	1.0702	FALSE
BnaWGS-1007	Hap12	Hap6	-21.8	0	-30.4122	-13.1878	TRUE
BnaWGS-1007	Hap12	Hap7	-11.0923	0.0028	-19.9655	-2.2191	TRUE

For Review Only

BnaWGS-1007	Hap12	Hap8	-6.1	0.6049	-15.5341	3.3341	FALSE
BnaWGS-1007	Hap12	Hap9	-6.2	0.5797	-15.6341	3.2341	FALSE
BnaWGS-1007	Hap2	Hap3	-0.0841	1	-5.1618	4.9935	FALSE
BnaWGS-1007	Hap2	Hap4	-13.8398	0	-19.6113	-8.0682	TRUE
BnaWGS-1007	Hap2	Hap5	-1.8556	0.9955	-7.5248	3.8137	FALSE
BnaWGS-1007	Hap2	Hap6	-16.5556	0	-22.845	-10.2661	TRUE
BnaWGS-1007	Hap2	Hap7	-5.8479	0.147	-12.4902	0.7945	FALSE
BnaWGS-1007	Hap2	Hap8	-0.8556	1	-8.2306	6.5195	FALSE
BnaWGS-1007	Hap2	Hap9	-0.9556	1	-8.3306	6.4195	FALSE
BnaWGS-1007	Hap3	Hap4	-13.7556	0	-20.0258	-7.4854	TRUE
BnaWGS-1007	Hap3	Hap5	-1.7714	0.9986	-7.9475	4.4047	FALSE
BnaWGS-1007	Hap3	Hap6	-16.4714	0	-23.2213	-9.7215	TRUE
BnaWGS-1007	Hap3	Hap7	-5.7637	0.2422	-12.8437	1.3162	FALSE
BnaWGS-1007	Hap3	Hap8	-0.7714	1	-8.5428	7	FALSE
BnaWGS-1007	Hap3	Hap9	-0.8714	1	-8.6428	6.9	FALSE
BnaWGS-1007	Hap4	Hap5	11.9842	0	5.2261	18.7424	TRUE
BnaWGS-1007	Hap4	Hap6	-2.7158	0.9867	-10.002	4.5705	FALSE
BnaWGS-1007	Hap4	Hap7	7.9919	0.0291	0.3989	15.5849	TRUE
BnaWGS-1007	Hap4	Hap8	12.9842	0	4.7426	21.2258	TRUE
BnaWGS-1007	Hap4	Hap9	12.8842	0	4.6426	21.1258	TRUE
BnaWGS-1007	Hap5	Hap6	-14.7	0	-21.9054	-7.4946	TRUE
BnaWGS-1007	Hap5	Hap7	-3.9923	0.8456	-11.5078	3.5232	FALSE
BnaWGS-1007	Hap5	Hap8	1	1	-7.1702	9.1702	FALSE
BnaWGS-1007	Hap5	Hap9	0.9	1	-7.2702	9.0702	FALSE
BnaWGS-1007	Hap6	Hap7	10.7077	0.0008	2.714	18.7014	TRUE
BnaWGS-1007	Hap6	Hap8	15.7	0	7.0878	24.3122	TRUE
BnaWGS-1007	Hap6	Hap9	15.6	0	6.9878	24.2122	TRUE
BnaWGS-1007	Hap7	Hap8	4.9923	0.7895	-3.8809	13.8655	FALSE
BnaWGS-1007	Hap7	Hap9	4.8923	0.8107	-3.9809	13.7655	FALSE
BnaWGS-1007	Hap8	Hap9	-0.1	1	-9.5341	9.3341	FALSE
BnaWGS-655	Hap1	Hap2	9.6399	0	6.8482	12.4316	TRUE
BnaWGS-655	Hap1	Hap3	1.8035	0.8044	-2.2889	5.896	FALSE
BnaWGS-655	Hap1	Hap4	2.7581	0.3841	-1.3344	6.8505	FALSE
BnaWGS-655	Hap1	Hap5	3.5819	0.2534	-1.1451	8.3089	FALSE
BnaWGS-655	Hap1	Hap6	1.5194	0.9407	-3.2076	6.2464	FALSE
BnaWGS-655	Hap2	Hap3	-7.8364	0	-12.4073	-3.2654	TRUE
BnaWGS-655	Hap2	Hap4	-6.8818	0.0003	-11.4528	-2.3109	TRUE
BnaWGS-655	Hap2	Hap5	-6.058	0.0107	-11.2048	-0.9111	TRUE
BnaWGS-655	Hap2	Hap6	-8.1205	0.0001	-13.2673	-2.9736	TRUE
BnaWGS-655	Hap3	Hap4	0.9545	0.9961	-4.5088	6.4179	FALSE
BnaWGS-655	Hap3	Hap5	1.7784	0.9563	-4.1751	7.7319	FALSE
BnaWGS-655	Hap3	Hap6	-0.2841	1	-6.2376	5.6694	FALSE
BnaWGS-655	Hap4	Hap5	0.8239	0.9987	-5.1297	6.7774	FALSE
BnaWGS-655	Hap4	Hap6	-1.2386	0.9912	-7.1922	4.7149	FALSE
BnaWGS-655	Hap5	Hap6	-2.0625	0.9403	-8.4688	4.3438	FALSE

For Review Only

pheno	gene	anova	count1	count2	mean1	mean2
Flowering Time	BnaA02G0156900ZS	0.301005615	356	28	181.2753	184.2143
Flowering Time	BnaA02G0156900ZS	3.82926E-09	49	31	179.7551	166.3548
Flowering Time	BnaA02G0156900ZS	3.82926E-09	49	26	179.7551	165.0385
Flowering Time	BnaA02G0156900ZS	3.82926E-09	49	15	179.7551	163.2
Flowering Time	BnaA02G0156900ZS	3.82926E-09	49	11	179.7551	166.5455
Flowering Time	BnaA02G0156900ZS	3.82926E-09	49	10	179.7551	164.3
Flowering Time	BnaA02G0156900ZS	3.82926E-09	49	7	179.7551	164.8571
Flowering Time	BnaA02G0156900ZS	3.82926E-09	31	26	166.3548	165.0385
Flowering Time	BnaA02G0156900ZS	3.82926E-09	31	15	166.3548	163.2
Flowering Time	BnaA02G0156900ZS	3.82926E-09	31	11	166.3548	166.5455
Flowering Time	BnaA02G0156900ZS	3.82926E-09	31	10	166.3548	164.3
Flowering Time	BnaA02G0156900ZS	3.82926E-09	31	7	166.3548	164.8571
Flowering Time	BnaA02G0156900ZS	3.82926E-09	26	15	165.0385	163.2
Flowering Time	BnaA02G0156900ZS	3.82926E-09	26	11	165.0385	166.5455
Flowering Time	BnaA02G0156900ZS	3.82926E-09	26	10	165.0385	164.3
Flowering Time	BnaA02G0156900ZS	3.82926E-09	26	7	165.0385	164.8571
Flowering Time	BnaA02G0156900ZS	3.82926E-09	15	11	163.2	166.5455
Flowering Time	BnaA02G0156900ZS	3.82926E-09	15	10	163.2	164.3
Flowering Time	BnaA02G0156900ZS	3.82926E-09	15	7	163.2	164.8571
Flowering Time	BnaA02G0156900ZS	3.82926E-09	11	10	166.5455	164.3
Flowering Time	BnaA02G0156900ZS	3.82926E-09	11	7	166.5455	164.8571
Flowering Time	BnaA02G0156900ZS	3.82926E-09	10	7	164.3	164.8571
Flowering Time	BnaA10G0244800ZS	2.41E-16	239	12	181.3222	180.1667
Flowering Time	BnaA10G0244800ZS	2.41E-16	239	8	181.3222	165.75
Flowering Time	BnaA10G0244800ZS	2.41E-16	239	10	181.3222	186.4
Flowering Time	BnaA10G0244800ZS	2.41E-16	239	45	181.3222	181.1556
Flowering Time	BnaA10G0244800ZS	2.41E-16	239	28	181.3222	181.0714
Flowering Time	BnaA10G0244800ZS	2.41E-16	239	19	181.3222	167.3158
Flowering Time	BnaA10G0244800ZS	2.41E-16	239	20	181.3222	179.3
Flowering Time	BnaA10G0244800ZS	2.41E-16	239	15	181.3222	164.6
Flowering Time	BnaA10G0244800ZS	2.41E-16	239	13	181.3222	175.3077
Flowering Time	BnaA10G0244800ZS	2.41E-16	239	10	181.3222	180.3
Flowering Time	BnaA10G0244800ZS	2.41E-16	239	10	181.3222	180.2
Flowering Time	BnaA10G0244800ZS	2.41E-16	12	8	180.1667	165.75
Flowering Time	BnaA10G0244800ZS	2.41E-16	12	10	180.1667	186.4
Flowering Time	BnaA10G0244800ZS	2.41E-16	12	45	180.1667	181.1556
Flowering Time	BnaA10G0244800ZS	2.41E-16	12	28	180.1667	181.0714
Flowering Time	BnaA10G0244800ZS	2.41E-16	12	19	180.1667	167.3158
Flowering Time	BnaA10G0244800ZS	2.41E-16	12	20	180.1667	179.3
Flowering Time	BnaA10G0244800ZS	2.41E-16	12	15	180.1667	164.6
Flowering Time	BnaA10G0244800ZS	2.41E-16	12	13	180.1667	175.3077
Flowering Time	BnaA10G0244800ZS	2.41E-16	12	10	180.1667	180.3
Flowering Time	BnaA10G0244800ZS	2.41E-16	12	10	180.1667	180.2
Flowering Time	BnaA10G0244800ZS	2.41E-16	8	10	165.75	186.4
Flowering Time	BnaA10G0244800ZS	2.41E-16	8	45	165.75	181.1556
Flowering Time	BnaA10G0244800ZS	2.41E-16	8	28	165.75	181.0714
Flowering Time	BnaA10G0244800ZS	2.41E-16	8	19	165.75	167.3158
Flowering Time	BnaA10G0244800ZS	2.41E-16	8	20	165.75	179.3
Flowering Time	BnaA10G0244800ZS	2.41E-16	8	15	165.75	164.6
Flowering Time	BnaA10G0244800ZS	2.41E-16	8	13	165.75	175.3077
Flowering Time	BnaA10G0244800ZS	2.41E-16	8	10	165.75	180.3
Flowering Time	BnaA10G0244800ZS	2.41E-16	8	10	165.75	180.2
Flowering Time	BnaA10G0244800ZS	2.41E-16	10	45	186.4	181.1556
Flowering Time	BnaA10G0244800ZS	2.41E-16	10	28	186.4	181.0714
Flowering Time	BnaA10G0244800ZS	2.41E-16	10	19	186.4	167.3158
Flowering Time	BnaA10G0244800ZS	2.41E-16	10	20	186.4	179.3
Flowering Time	BnaA10G0244800ZS	2.41E-16	10	15	186.4	164.6
Flowering Time	BnaA10G0244800ZS	2.41E-16	10	13	186.4	175.3077

For Review Only

Flowering Time	BnaA10G0244800ZS	2.41E-16	10	10	186.4	180.3
Flowering Time	BnaA10G0244800ZS	2.41E-16	10	10	186.4	180.2
Flowering Time	BnaA10G0244800ZS	2.41E-16	45	28	181.1556	181.0714
Flowering Time	BnaA10G0244800ZS	2.41E-16	45	19	181.1556	167.3158
Flowering Time	BnaA10G0244800ZS	2.41E-16	45	20	181.1556	179.3
Flowering Time	BnaA10G0244800ZS	2.41E-16	45	15	181.1556	164.6
Flowering Time	BnaA10G0244800ZS	2.41E-16	45	13	181.1556	175.3077
Flowering Time	BnaA10G0244800ZS	2.41E-16	45	10	181.1556	180.3
Flowering Time	BnaA10G0244800ZS	2.41E-16	45	10	181.1556	180.2
Flowering Time	BnaA10G0244800ZS	2.41E-16	28	19	181.0714	167.3158
Flowering Time	BnaA10G0244800ZS	2.41E-16	28	20	181.0714	179.3
Flowering Time	BnaA10G0244800ZS	2.41E-16	28	15	181.0714	164.6
Flowering Time	BnaA10G0244800ZS	2.41E-16	28	13	181.0714	175.3077
Flowering Time	BnaA10G0244800ZS	2.41E-16	28	10	181.0714	180.3
Flowering Time	BnaA10G0244800ZS	2.41E-16	28	10	181.0714	180.2
Flowering Time	BnaA10G0244800ZS	2.41E-16	19	20	167.3158	179.3
Flowering Time	BnaA10G0244800ZS	2.41E-16	19	15	167.3158	164.6
Flowering Time	BnaA10G0244800ZS	2.41E-16	19	13	167.3158	175.3077
Flowering Time	BnaA10G0244800ZS	2.41E-16	19	10	167.3158	180.3
Flowering Time	BnaA10G0244800ZS	2.41E-16	19	10	167.3158	180.2
Flowering Time	BnaA10G0244800ZS	2.41E-16	20	15	179.3	164.6
Flowering Time	BnaA10G0244800ZS	2.41E-16	20	13	179.3	175.3077
Flowering Time	BnaA10G0244800ZS	2.41E-16	20	10	179.3	180.3
Flowering Time	BnaA10G0244800ZS	2.41E-16	20	10	179.3	180.2
Flowering Time	BnaA10G0244800ZS	2.41E-16	15	13	164.6	175.3077
Flowering Time	BnaA10G0244800ZS	2.41E-16	15	10	164.6	180.3
Flowering Time	BnaA10G0244800ZS	2.41E-16	15	10	164.6	180.2
Flowering Time	BnaA10G0244800ZS	2.41E-16	13	10	175.3077	180.3
Flowering Time	BnaA10G0244800ZS	2.41E-16	13	10	175.3077	180.2
Flowering Time	BnaA10G0244800ZS	2.41E-16	10	10	180.3	180.2
Flowering Time	BnaA10G0244800ZS	2.79E-06	180	55	164.1056	173.7455
Flowering Time	BnaA10G0244800ZS	2.79E-06	180	22	164.1056	165.9091
Flowering Time	BnaA10G0244800ZS	2.79E-06	180	22	164.1056	166.8636
Flowering Time	BnaA10G0244800ZS	2.79E-06	180	16	164.1056	167.6875
Flowering Time	BnaA10G0244800ZS	2.79E-06	180	16	164.1056	165.625
Flowering Time	BnaA10G0244800ZS	2.79E-06	55	22	173.7455	165.9091
Flowering Time	BnaA10G0244800ZS	2.79E-06	55	22	173.7455	166.8636
Flowering Time	BnaA10G0244800ZS	2.79E-06	55	16	173.7455	167.6875
Flowering Time	BnaA10G0244800ZS	2.79E-06	55	16	173.7455	165.625
Flowering Time	BnaA10G0244800ZS	2.79E-06	22	22	165.9091	166.8636
Flowering Time	BnaA10G0244800ZS	2.79E-06	22	16	165.9091	167.6875
Flowering Time	BnaA10G0244800ZS	2.79E-06	22	16	165.9091	165.625
Flowering Time	BnaA10G0244800ZS	2.79E-06	22	16	166.8636	167.6875
Flowering Time	BnaA10G0244800ZS	2.79E-06	22	16	166.8636	165.625
Flowering Time	BnaA10G0244800ZS	2.79E-06	16	16	167.6875	165.625

For Review Only

std1	std2
4.52069497	14.69784
10.4573152	7.842399
10.4573152	6.94827
10.4573152	2.677952
10.4573152	9.469568
10.4573152	3.368151
10.4573152	4.375255
7.84239924	6.94827
7.84239924	2.677952
7.84239924	9.469568
7.84239924	3.368151
7.84239924	4.375255
6.9482704	2.677952
6.9482704	9.469568
6.9482704	3.368151
6.9482704	4.375255
2.67795231	9.469568
2.67795231	3.368151
2.67795231	4.375255
9.46956848	3.368151
9.46956848	4.375255
3.36815149	4.375255
5.41868556	6.991337
5.41868556	7.759786
5.41868556	10.37304
5.41868556	4.651273
5.41868556	10.83864
5.41868556	4.534468
5.41868556	8.079213
5.41868556	7.199206
5.41868556	10.09443
5.41868556	5.657836
5.41868556	5.6921
6.99133663	7.759786
6.99133663	10.37304
6.99133663	4.651273
6.99133663	10.83864
6.99133663	4.534468
6.99133663	8.079213
6.99133663	7.199206
6.99133663	10.09443
6.99133663	5.657836
6.99133663	5.6921
7.75978645	10.37304
7.75978645	4.651273
7.75978645	10.83864
7.75978645	4.534468
7.75978645	8.079213
7.75978645	7.199206
7.75978645	10.09443
7.75978645	5.657836
7.75978645	5.6921
10.373042	4.651273
10.373042	10.83864
10.373042	4.534468
10.373042	8.079213
10.373042	7.199206
10.373042	10.09443

For Review Only

10.373042	5.657836
10.373042	5.6921
4.65127331	10.83864
4.65127331	4.534468
4.65127331	8.079213
4.65127331	7.199206
4.65127331	10.09443
4.65127331	5.657836
4.65127331	5.6921
10.8386434	4.534468
10.8386434	8.079213
10.8386434	7.199206
10.8386434	10.09443
10.8386434	5.657836
10.8386434	5.6921
4.53446838	8.079213
4.53446838	7.199206
4.53446838	10.09443
4.53446838	5.657836
4.53446838	5.6921
8.07921309	7.199206
8.07921309	10.09443
8.07921309	5.657836
8.07921309	5.6921
7.19920631	10.09443
7.19920631	5.657836
7.19920631	5.6921
10.094426	5.657836
10.094426	5.6921
5.65783626	5.6921
3.66242772	11.1807
3.66242772	4.65893
3.66242772	4.97896
3.66242772	8.498774
3.66242772	8.01561
11.1807013	4.65893
11.1807013	4.97896
11.1807013	8.498774
11.1807013	8.01561
4.65892989	4.97896
4.65892989	8.498774
4.65892989	8.01561
4.97896006	8.498774
4.97896006	8.01561
8.49877442	8.01561

For Review Only

Table S9. The haplotyes network information of *BnFT.A02* in BnaWGS-1007 panel

source	target	distance
Hap1	Hap2	1
Hap1	Hap3	1
Hap2	Hap4	4
Hap2	Hap6	8
Hap3	Hap12	2
Hap3	Hap18	2
Hap3	Hap9	6
Hap5	Hap7	1
Hap5	Hap8	1
Hap5	Hap16	8
Hap6	Hap10	1
Hap6	Hap19	2
Hap8	Hap9	2
Hap10	Hap13	1
Hap11	Hap20	2
Hap12	Hap15	2
Hap14	Hap17	1
Hap14	Hap18	7
Hap16	Hap20	1

Note: Distance indicates the Hamming distance between two haplotypes

For Review Only

Table S10. The haplotyes network information of *BnFT.A02* in BnaWGS-655 panel

source	target	distance
Hap1	Hap3	1
Hap1	Hap4	1
Hap1	Hap5	2
Hap1	Hap6	3
Hap2	Hap9	1
Hap2	Hap10	1
Hap2	Hap16	1
Hap2	Hap18	1
Hap2	Hap6	2
Hap3	Hap19	2
Hap5	Hap12	1
Hap6	Hap8	1
Hap6	Hap17	6
Hap7	Hap13	2
Hap8	Hap20	1
Hap9	Hap15	2
Hap11	Hap13	1
Hap11	Hap17	1
Hap14	Hap16	1

Note: Distance indicates the Hamming distance between two haplotypes

For Review Only

Table S11. The haplotyes network information of *BnFLC.A10* in BnaWGS-1007 panel

source	target	distance
Hap1	Hap2	1
Hap1	Hap7	1
Hap1	Hap9	1
Hap1	Hap10	1
Hap1	Hap12	1
Hap1	Hap14	1
Hap1	Hap15	1
Hap1	Hap16	1
Hap1	Hap18	1
Hap1	Hap20	1
Hap3	Hap5	1
Hap3	Hap13	1
Hap3	Hap19	1
Hap4	Hap6	1
Hap4	Hap8	13
Hap5	Hap15	1
Hap5	Hap17	1
Hap6	Hap11	3
Hap7	Hap8	1

Note: Distance indicates the Hamming distance between two haplotypes

For Review Only

Table S12. The haplotyes network information of *BnFLC.A10* in BnaWGS-655 panel

source	target	distance
Hap1	Hap11	1
Hap1	Hap12	1
Hap1	Hap18	1
Hap1	Hap19	2
Hap2	Hap6	1
Hap2	Hap7	1
Hap2	Hap14	1
Hap2	Hap15	1
Hap2	Hap17	1
Hap3	Hap4	1
Hap3	Hap19	1
Hap3	Hap8	5
Hap4	Hap16	2
Hap5	Hap6	1
Hap5	Hap9	1
Hap5	Hap10	1
Hap5	Hap20	1
Hap6	Hap8	7
Hap12	Hap13	1

Note: Distance indicates the Hamming distance between two haplotypes

For Review Only
